# IMRAS—A clinical trial of mosquito-bite immunization with live, radiation-attenuated *P. falciparum* sporozoites: Impact of immunization parameters on protective efficacy and generation of a repository of immunologic reagents

**DOI:** 10.1371/journal.pone.0233840

**Published:** 2020-06-17

**Authors:** Bradley Hickey, Nimfa Teneza-Mora, Joanne Lumsden, Sharina Reyes, Martha Sedegah, Lindsey Garver, Michael R. Hollingdale, Jo Glenna Banania, Harini Ganeshan, Megan Dowler, Anatalio Reyes, Cindy Tamminga, Alexandra Singer, Alicia Simmons, Maria Belmonte, Arnel Belmonte, Jun Huang, Sandra Inoue, Rachel Velasco, Steve Abot, Carlos S. Vasquez, Ivelese Guzman, Mimi Wong, Patrick Twomey, Mariusz Wojnarski, James Moon, Yolanda Alcorta, Santina Maiolatesi, Michele Spring, Silas Davidson, Sidhartha Chaudhury, Eileen Villasante, Thomas L. Richie, Judith E. Epstein

**Affiliations:** 1 Malaria Department, Naval Medical Research Center, Silver Spring, MD, United States of America; 2 Henry M. Jackson Foundation, Bethesda, MD, United States of America; 3 Walter Reed Army Institute of Research, Silver Spring, MD, United States of America; 4 Biotechnology HPC Software Applications Institute, Telemedicine and Advanced Technology Research Center, U.S. Army Medical Research and Development Command, Frederick, MD, United States of America; 5 Malaria Department, Naval Medical Research Center, Silver Spring, MD, United States of America; University of Tübingen, GERMANY

## Abstract

**Background:**

Immunization with radiation-attenuated sporozoites (RAS) by mosquito bite provides >90% sterile protection against *Plasmodium falciparum* (Pf) malaria in humans. RAS invade hepatocytes but do not replicate. CD8+ T cells recognizing parasite-derived peptides on the surface of infected hepatocytes are likely the primary protective mechanism. We conducted a randomized clinical trial of RAS immunization to assess safety, to achieve 50% vaccine efficacy (VE) against controlled human malaria infection (CHMI), and to generate reagents from protected and non-protected subjects for future identification of protective immune mechanisms and antigens.

**Methods:**

Two cohorts (Cohort 1 and Cohort 2) of healthy, malaria-naïve, non-pregnant adults age 18–50 received five monthly immunizations with infected (true-immunized, n = 21) or non-infected (mock-immunized, n = 5) mosquito bites and underwent homologous CHMI at 3 weeks. Immunization parameters were selected for 50% protection based on prior clinical data. Leukapheresis was done to collect plasma and peripheral blood mononuclear cells.

**Results:**

Adverse event rates were similar in true- and mock-immunized subjects. Two true- and two mock-immunized subjects developed large local reactions likely caused by mosquito salivary gland antigens. In Cohort 1, 11 subjects received 810–1235 infected bites; 6/11 (55%) were protected against CHMI vs. 0/3 mock-immunized and 0/6 infectivity controls (VE 55%). In Cohort 2, 10 subjects received 839–1131 infected bites with a higher first dose and a reduced fifth dose; 9/10 (90%) were protected vs. 0/2 mock-immunized and 0/6 controls (VE 90%). Three/3 (100%) protected subjects administered three booster immunizations were protected against repeat CHMI vs. 0/6 controls (VE 100%). Cohort 2 uniquely showed a significant rise in IFN-γ responses after the third and fifth immunizations and higher antibody responses to CSP.

**Conclusions:**

PfRAS were generally safe and well tolerated. Cohort 2 had a higher first dose, reduced final dose, higher antibody responses to CSP and significant rise of IFN-γ responses after the third and fifth immunizations. Whether any of these factors contributed to increased protection in Cohort 2 requires further investigation. A cryobank of sera and cells from protected and non-protected individuals was generated for future immunological studies and antigen discovery.

**Trial registration:**

ClinicalTrials.gov NCT01994525.

## Introduction

In clinical trials conducted at the Naval Medical Research Center (NMRC) in 1989–1999, immunization with *Plasmodium falciparum* (Pf) radiation-attenuated sporozoites (RAS), administered by greater than 1000 bites of irradiated mosquitoes, elicited up to 93% sterile protection against controlled human malaria infection (CHMI) conducted within 10 weeks of immunization [1]. Moreover, 6/6 subjects previously protected and receiving booster immunizations were protected against repeat CHMI within 10 weeks and 5/6 were protected against repeat CHMI 23–42 weeks after last immunization. These studies indicated that a malaria vaccine inducing durable immunity to the pre-erythrocytic stages of malaria life cycle was feasible.

The RAS model has proven valuable to characterize immune responses that confer sterile protection as well as identifying protective sporozoite and liver stage antigens. CD8+ T lymphocyte responses targeting peptides expressed on the surface of infected hepatocytes derived from parasite antigens carried into the hepatocyte during SPZ invasion, or expressed during early liver stage development in association with MHC class I, have been linked with protection [2]. The most advanced malaria sub-unit vaccine, RTS,S/AS01, is based on the circumsporozoite protein (CSP), and reduces the incidence of clinical malaria by about 30% in young children [3] but has not been shown to prevent parasitemia in adults in endemic areas [4]. There remains a need to identify additional protective sporozoite and/or liver antigens to replace or combine with CSP to enhance VE.

RAS immunization can induce sterile protection in the absence of responses to CSP, establishing that multiple SPZ and/or liver stage antigens are likely involved [5, 6]. Examination of the humoral [7] and cell mediated immune [5] responses revealed significant differences between protected and non-protected subjects and led to the discovery of new Pf antigens [8, 9]. For example, CelTOS (cell-traversal protein for ookinetes and sporozoites) [10, 11] recalled significantly higher cellular responses from protected than non-protected subjects [5]. A robust repository of samples from protected and non-protected subjects immunized with PfRAS, in a trial conducted under well controlled circumstances, could contribute to a better understanding of protective immune responses.

Additional studies of PfRAS were conducted between 1999 and 2002 by the Naval Medical Research Center (NMRC) [12]. However, only 5 of ten subjects immunized with > 1,000 bites of irradiated, infected mosquitoes were protected against CHMI [12]. As with earlier studies, immunization was performed according to the availability of PfRAS-infected mosquitoes, resulting in varying immunization schedules and times to CHMI for each subject, raising the question whether differences in the various immunization parameters may have affected VE. Given the large number of variables, when data from all 20 research subjects in both NMRC trials were combined, the only positive finding was a shorter median interval between last immunization and CHMI in protected (20 days) than in non-protected subjects (36 days) [12].

This new trial was designed to replicate the 50% protection against CHMI achieved in the 1999–2002 trial. It was reasoned that comparing the immune responses to candidate antigens in protected and non-protected volunteers could identify antigens or immune mechanisms contributing to protection, and that roughly equal numbers of each would enhance discriminatory power. We hypothesized that, based on previous trials, a range of 800–1200 infected bites and an interval of 22–24 days before CHMI might lead to ~50% VE (S1 Fig: Association between number of infectious mosquito bites and time to CHMI with efficacy). This was to be achieved in two cohorts: Cohort 1 would receive five immunizations totaling 800–1200 infected bites followed by CHMI 22–24 days later, and some protected subjects (Hyperimmunity Continuation Phase Cohort) would receive three further immunizations and a second CHMI approximately 3 months later. If 50% protective efficacy was achieved with the first cohort, the same immunization regimen would be administered to the second cohort. However, if the VE in the first cohort was significantly different from 50%, the number of immunization sessions for the second cohort would be adjusted up or down (to 3 or to 7) to target 50% efficacy.

We report the results of this trial, called IMRAS (Immunization by Mosquito bite with RAS), focusing on safety, tolerability, VE, and, as a result of the unexpected difference in protection observed in the two cohorts, an analysis of the vaccination parameters potentially affecting VE. We have included selected antibody and cellular immune responses, primarily to explore significant differences between Cohort 1 and Cohort 2 and whether these are associated with the unexpected increased VE in Cohort 2. However, generation of a repository of cryopreserved samples will allow further investigation of these responses.

## Materials and methods

### Objectives

The study was designed to induce protective immunity against CHMI in approximately 50% of study subjects, in malaria-naïve adults, by immunization with radiation-attenuated *P*. *falciparum* sporozoites (PfRAS) administered via mosquito bites. The objectives were to assess safety and tolerability, compared with mock-immunization via non-infected mosquito bites, and to create a repository of samples from these subjects. The repository was intended for use in future studies for the identification of biomarkers of protection, including host response and antigenic targets, by comparing protected, non-protected, and mock-immunized subjects, and identifying immune correlates of high-grade, durable protection in hyperimmunized subjects.

### Ethics

The study was conducted at the Naval Medical Research Center (NMRC) Clinical Trials Center from 2014 to 2016; the CHMIs were conducted at the Walter Reed Army Institute of Research (WRAIR) secure insectary. The study protocol was reviewed and approved by the NMRC Institutional Review Board in compliance with all federal regulations governing the protection of human subjects. WRAIR holds a Federal-wide Assurance from the Office of Human Research Protections (OHRP) under the Department of Health and Human Services as does NMRC. NMRC also holds a Department of Defense/Department of the Navy Federal-wide Assurance for human subject protections. All key personnel were certified as having completed mandatory human subjects’ protection curricula and training under the direction of the WRAIR Institutional Review Board or the NMRC Office of Research Administration (ORA) and Human Subjects Protections Branch (HSPB). All potential study subjects provided written, informed consent before screening and enrollment and had to pass an assessment of understanding. This study was conducted according to the Declaration of Helsinki as well as principles of Good Clinical Practices under the United States Food and Drug Administration Investigational New Drug (IND) application BB-15767. This trial was performed under an IND allowance by the Food and Drug Administration (FDA) and was registered on ClinicalTrials.gov (NCT01994525).

### Study design

This was an open-label clinical study for safety and identification of biomarkers of protection in two cohorts of healthy malaria-naïve adults, who received five immunization sessions with bites from *Anopheles stephensi* mosquitoes either infected with PfRAS (true-immunization) or non-infected (mock-immunization). The study design is summarized in S2 Fig.

To accommodate the capacity of the entomology facility and enable flexibility towards achieving the goal of 50% sterile protection, there were 2 cohorts (S2 Fig). The planned sample size for each cohort was 12–14 true-immunized subjects who were immunized by the bites of *An*. *stephensi* mosquitoes infected with irradiated Pf sporozoites, followed by CHMI; four mock-immunized subjects who were immunized by the bites of non-infected mosquitoes, followed by CHMI; and six infectivity controls who were not immunized but were exposed to CHMI at the same time as the true-immunized subjects. The inclusion of mock-immunized subjects was deemed critical, since mosquito salivary gland antigens (SGAs) are major immunogens inoculated during mosquito bite immunization. Without these controls, it would not be possible to differentiate immune responses generated solely by SGAs and not by RAS.

The first cohort was completed prior to initiating the second cohort. The target dose was 960 (range 800 to 1200) infected bites for the group median. All immunized subjects (true-immunized and mock-immunized) plus 6 infectivity controls underwent CHMI approximately 3 weeks after the fifth immunization session. For Cohort 2, the dosing schedule was to be adjusted based on the rate of protection observed in Cohort 1 to achieve approximately 50% protection; both cohorts were to receive identical immunization regimens if protection in the first cohort was 40–60%; alternatively, the second cohort would receive more or fewer immunizations if protection in the first cohort was <40% or >60%, respectively.

Hyperimmunity Continuation Phase: protected subjects from Cohort 1 were offered the option to enroll in a continuation phase of the trial to explore the presumed high-grade, durable immunity generated following primary CHMI and boosting immunizations. Three subjects electing this option received 3 secondary immunizations at 47, 51 and 55 weeks and a secondary CHMI at 67 weeks after the first immunization in conjunction with Cohort 2.

### Study population

Healthy, malaria-naïve, non-pregnant adults between the ages of 18 and 50 were included in this study. Subjects were excluded if they had a history of malaria infection, travel to a malaria endemic region within 6 months of the first immunization, history of long-term residence (> 5 years) in an area known to have significant transmission of *P*. *falciparum*, or reactivity by CSP or AMA1 ELISpot assay or ELISA. All study subjects underwent a screening evaluation of medical history, physical examination, electrocardiogram, complete blood count, clinical biochemistries, urinalysis, sickle cell testing and serological studies for previous exposure to or infection with human immunodeficiency virus (HIV), hepatitis B, or hepatitis C. Subjects were excluded if they had any significant medical condition (cardiovascular, hepatic, renal, pulmonary, or hematological), history of anaphylactic or other severe response to mosquito bites, splenectomy, or evidence of increased cardiovascular risk (defined as >5–10%, 5-year risk) [13]. All females had urine pregnancy test at screening, immediately before each immunization and before CHMI; they were to be excluded from further immunization or CHMI if this was positive. All female subjects agreed to use effective means of birth control for the duration of the trial. Prior to first immunization, subjects eligible to receive immunization via mosquito bites were randomized (block randomization, block size of 4) to the true-immunized or mock-immunized groups using a 3:1 ratio.

### True- and mock-immunization procedures

#### Mosquito production

Female *An*. *stephensi* were used for immunizations and CHMI. For each immunization, 4,000–6,000 mosquitoes were infected by membrane feeding on *in vitro* cultures of the NF54 strain of *P*. *falciparum* 18–22 days prior to the day of immunization. Mosquitoes were kept in a secure insectary at 26°C [78.8°F] + 5°C with relative humidity at 75% ± 15%.

#### Sporozoite production and grading

*P*. *falciparum* asexual and sexual erythrocytic stage parasites were grown in normal human erythrocytes using standard culture medium containing 10%-15% normal human serum. All erythrocytes and serum were obtained from donors at low risk for both hepatitis and HIV infection and whose serum was non-reactive to syphilis or HIV. Blood and serum for parasite culture were purchased from a commercial source and each shipment carried a certificate of analysis certifying that the blood products were negative or non-reactive for these pathogens.

Approximately 14 days after membrane feeding, salivary glands of 10 mosquitoes were dissected and scored for the presence of sporozoites: 1–10 sporozoites = gland score 1; 11–100 sporozoites = gland score 2; 101–1,000 sporozoites = gland score 3; and >1,000 sporozoites = gland score 4. A gland score of 2 or higher was recorded as ‘infected’ for study use, although those with ten or fewer sporozoites could still be infected and inject sporozoites during feeding if utilized.

#### Irradiation of sporozoites

Batches of infected mosquitoes with an infectivity rate of 70% or more and a gland score of 2 or higher were exposed to 15,000 rad (cGy) of gamma radiation using a Model 109–68 Cobalt^60^ irradiator prior to immunizing subjects. This dose is sufficient to attenuate the sporozoites, preventing development of a patent blood-stage malaria infection, while permitting the development of a protective anti-hepatic stage immune response [12, 14, 15].

#### Immunization procedures

True- and mock-immunizations were conducted in the secure WRAIR insectary by placing a container containing approximately 200–400 mosquitoes in contact with the volar surface of one forearm for 5 minutes, followed 2 minutes later by a second 5-min feed with the same mosquitoes at the same site. After feeding, mosquitoes were examined to determine the proportion having taken a blood meal, and of the mosquitoes taking a blood meal, approximately 30 (7.5%-15% total mosquitoes in each container) were dissected to determine the percentage having a sporozoite gland score >2 [16]. This percentage was multiplied by the total number of mosquitoes taking a blood meal to calculate the number of immunizing bites [1]. Subjects were not additionally immunized at that time point but, as discussed below (see Fig 3), numbers of infected bites were fairly consistent at each immunization. Mosquitoes used for mock-immunization were raised, handled and irradiated in the same fashion as those for true-immunization except they were fed on blood cultures not infected with *P*. *falciparum*. Both true- and mock-immunized subjects were observed on site for at least 30 min after each immunization.

#### Controlled human malaria infection (CHMI)

Five non-irradiated mosquitoes, infected with the same NF54 strain of *P*. *falciparum* used for immunization, were allowed to feed once for 5 minutes on each subject. All fed mosquitoes were dissected to determine the infectivity rate. Replacement mosquitoes for those of the initial five mosquitoes either not feeding or feeding and found to have gland grades of 1 or less (ten sporozoites or fewer) were then allowed to feed and this process was repeated until five infectious bites had been achieved. Beginning seven days after CHMI, subjects were housed each night for close clinical monitoring by study staff. Each morning, thick blood smears were made for microscopic examination under high-power objective such that approximately 0.55 μL of blood were examined. The presence of two parasites was required for a positive diagnosis, leading to immediate antimalarial treatment with a standard dose of chloroquine or Malarone^®^ (atovaquone/proguanil). The treatment regimen was directly observed and included 1,500 mg chloroquine given orally in divided doses (600 mg initially, followed by 300 mg given at approximately 6, 24, and 48 hours after the first dose) or Malarone^®^ (250 mg atovaquone/100 mg proguanil tablets) 4 tablets taken orally as a single dose once per day for 3 days. Subjects who were diagnosed with parasitemia by thick smear were monitored daily by symptom checks and blood smears until three consecutive negative smears were documented. Subjects who remained negative for parasitemia were similarly monitored daily until day 18 post CHMI, then approximately every other day until day 28. Those remaining negative on day 28 were considered sterilely protected and did not receive antimalarial therapy.

#### Leukapheresis

Leukapheresis was performed to collect a large amount of peripheral blood mononuclear cells (PBMCs) to identify biomarkers of protection, as available evidence suggests the importance of PBMCs in mediating protection [17]. The volume processed for leukapheresis was approximately two blood volumes, about 10–12 liters. In most subjects this was to result in collection of a volume of 200–300 mL of plasma mixed with citrate and 1 ×10^9^ WBCs per liter processed with a target PBMC total content in the bag of > 7.5 × 10^8^. The lost plasma volume was replaced with normal saline solution. There was a net loss of packed hemoglobin equivalent to approximately 20 mL of whole blood. Subjects underwent leukapheresis before the first immunization (all enrolled subjects), after the third immunization (optional, no more than 50% immunized subjects allowed to undergo the procedure as the effects of removing so many PBMCs on protective efficacy are unknown), approximately 5–6 days post-CHMI of immunized subjects (based on the same concern that removing many PBMCs prior to CHMI might adversely affect protection) and at approximately 4–6 month post-CHMI (optional, no limit to how many immunized subjects). Subjects participating in the Hyperimmunity Continuation Phase Cohort underwent 2 additional leukapheresis procedures following the first and third boosting immunizations.

### Assessment of tolerability and safety

#### Immunizations

Solicited adverse events (AEs) were assessed through day 7 after immunization; unsolicited AEs were assessed through day 14; and laboratory abnormalities were assessed through day 7. Serious adverse events (SAEs) were assessed from first immunization to the end of the trial.

#### CHMI

Solicited local signs and symptoms were collected through day 7 post-CHMI and solicited systemic signs and symptoms were collected through day 6. Unsolicited signs and symptoms were collected through day 7 post-CHMI. Starting at day 7 post-CHMI, subjects were monitored for signs and symptoms consistent with malaria infection that were documented but not categorized as AEs because they were expected as a result of malaria infection.

#### Assessment of relatedness and severity of adverse events

AEs were assessed as definitely related, probably related, possibly related, unlikely related or unrelated to PfRAS-infected or non-infected mosquito immunizations. All AEs were assessed for severity including the medical and clinical consideration of all information surrounding the event including any medical intervention required. Each event was assigned one of the following categories: Grade 1 (mild): Does not interfere with routine activities, minimal level of discomfort; Grade 2 (moderate): Interferes with routine activities, moderate level of discomfort; Grade 3: Unable to perform routine activities, significant level of discomfort; Grade 4: Hospitalization for potentially life-threatening event. Any grade 4 AE was reported as a SAE.

#### FluoroSpot assay

PBMCs were obtained via withdrawal of whole blood from subjects in both Cohorts (sampling separate from leukapheresis): pre-immunization; 7 days (Cohort 1 only) and 28 days after the first immunizations (both cohorts); 28 days after the second and third immunizations; 35 days after the fourth immunization; 22–24 days after the fifth immunization (the day of CHMI); and 39–41 days after CHMI. Antigen-specific circulating PBMCs secreting single or multiple cytokines were evaluated using pre-coated FluoroSpot plates and kits purchased from Mabtech (Mabtech AB, Nacka Strand, Sweden) and used according to the manufacturer’s instructions. The previously described *ex vivo* ELISpot was modified [18]; briefly, PBMCs were incubated in the FluoroSpot plates with 2.5 x 10^4^ irradiated (150 Gy), aseptic, purified cryopreserved NF54 PfSPZ (Sanaria Inc., Rockville, MD) suspended in 100 μL complete medium. CTL-CEF-Class I Peptide Pool Plus (Cellular Technology Ltd, Cleveland, OH) consisting of 32 peptides corresponding to defined HLA class I-restricted T cell epitopes from cytomegalovirus, Epstein-Barr virus and influenza virus was used as an internal control for each subject. PHA, a mitogen, was used as a positive control for cell viability. Negative control unstimulated PBMCs received medium only. Cultures were incubated for 36 h at 37°C in 5% CO_2_. Each PBMC sample was assayed in triplicate and the number of single-staining IFNγ- and IL2-secreting cells and double-staining IFNγ- and IL2-secreting cells were recognized as spot-forming cells (sfcs) and enumerated using an automated FluoroSpot reader (AID iSpot, Autoimmun Diagnostika GmbH, Strasberg, Germany). After subtraction of the mean number of sfcs in negative control wells (no antigen), the mean sfcs of the test sample was expressed as sfcs/10^6^ PBMCs.

#### Immunofluorescence antibody assay (IFA) using sporozoites

Antibody responses to whole sporozoites were measured by IFA activities to sporozoites pre-immunization and 22 days after the fifth immunization (day of CHMI). Serum antibody levels were assessed by IFA against air-dried *P*. *falciparum* 3D7 clone of NF54 [19] sporozoites as previously described [20].

#### Enzyme-linked immunosorbent assay (ELISA)

Antibodies to CSP, AMA1 and CelTOS were measured by ELISA pre-immunization, 14 days after the third immunization, the day of CHMI and 28 days after CHMI. ELISA assays were performed in the WRAIR Serology Laboratory that had previously validated these antigens: 20 ng/mL *Pf*CSP with the amino acid sequence CS(NANP)_6_C, 0.25 μg/mL *Pf*CSP Full Length [21], 50 ng/mL PfAMA-1, or 0.5 μg/mL recombinant CelTOS [11].

### Sample size and statistical assessment

The number of subjects enrolled in this study was limited by the complexity of the mosquito bite immunization procedures, leukapheresis procedures, and the capacity to rear and maintain infected mosquitoes at the secure WRAIR insectary. Twenty-four subjects per cohort (14 true-immunized, 4 mock-immunized, 6 infectivity controls), along with a maximum of 6 Hyperimmunity Continuation Phase subjects was the limit achievable based on these constraints. The high percentage of study completion in this complex clinical trial was a testament to the commitment of the study subjects.

#### Statistical analysis

Solicited adverse events, unsolicited adverse events, and laboratory abnormalities are presented in tabular form (cohort, true- or mock-immunized status, and AE grade). The cohort samples sizes were too small to reliably perform statistical comparison for mosquito bites and gland scores; therefore, the data are presented as median and range data. Kaplan-Meier survival curves were used to display parasitemia-free survival times for true-, mock-immunized, and infectivity controls. The log rank test was used to compare time to parasitemia between infectivity control and non-protected immunized subjects. The Mann Whitney U test was used to compare the interval between leukapheresis and CHMI for protected and non-protected subjects, comparison of mosquito bites and gland scores of true-immunized subjects between Cohort 1 and Cohort 2, and differences in ELISA and IFA responses (between cohorts and between protected and non-protected subjects in each cohort). Statistical significance was defined as a two-tailed P value of ≤0.05.

In order to assess the effect of time point and cohort on the immune data, we fit linear mixed models to the FluoroSpot, ELISA, and IFA data using the *lmr* function in the *lme4* package using the *R* statistical software. We assessed fixed effects (by time point, cohort, and the interaction of time point and cohort) and random effects (by subject). We reported any effects with a significance of p < 0.05. The *R* script used for the analysis can be found at https://github.com/BHSAI/IMRAS/lmm_script.R and the data is available upon request.

## Results

### Study flow

Enrollment took place from April 2014 until September 2015. The demographics of both cohorts were approximately balanced in gender, age and ethnic background (Table 1). All subjects within each cohort were randomized to true- or mock-immunization in a 3:1 allocation. Because the target of ~50% protection was achieved after the first cohort, the second cohort followed the same immunization schedule (five immunizations).

**Table 1:** Demographic composition of Cohort 1 and Cohort 2.

	Cohort 1	Cohort 2	Total
**Number of Subjects**			
**Gender**			
Male	20 (71%)	20 (77%)	40 (74%)
Female	8 (29%)	6 (23%)	14 (26%)
**Total**	28	26	54
**Age (Years)**			
18–19	2 (7%)	0	2 (4%)
20–29	14 (50%)	21 (81%)	35 (64%)
30–39	12 (43%)	3 (12%)	15 (28%)
40–49	0	2 (7%)	2 (4%)
50	0	0	0
**Average**	28.0	27.5	27.8
**Race**			
Am. Indian/Alaska N.	0	0	0
Asian	1 (4%)	1 (4%)	2 (4%)
Black or African Am.	2 (7%)	9 (35%)	11 (20%)
Nat. Hawaiian/Other Pacific Is.	0	0	0
White	20 (71%)	16 (61%)	36 (67%)
Other	3 (11%)	0	3 (5%)
Multiple^a^	2 (7%)	0	2 (4%)
**Total**	28	26	54
**Ethnicity**			
Hispanic/Latino	4 (14%)	2 (8%)	6 (11%)
Not Hispanic/Latino	21 (75%)	21 (80%)	42 (78%)
Not reported	2 (7%)	2 (8%)	4 (7%)
Unknown	1 (4%)	1 (4%)	2 (4%)
**Total**	28	26	54
**Military Member**			
No	21 (75%)	17 (65%)	38 (70%)
Yes	7 (25%)	9 (35%)	16 (30%)
**Total**	28	26	54

^a^ “Multiple” refers to subjects who identify with 2 or more race groups.

#### Cohort 1 (Fig 1)

True-immunization group. Before the first true-immunization, one subject was withdrawn for suspected steroid use; 13 subjects received the first true-immunization, and one subject then relocated; 12 subjects received the second true-immunization after which one subject was withdrawn because of an allergic reaction (S1 Appendix), and 11 subjects received the third true-immunization. Six of these 11 subjects then underwent leukapheresis and these six subjects and the remaining five subjects received the fourth and fifth true- immunizations. All 11 subjects then underwent CHMI followed by leukapheresis at day five or six post-CHMI; eight subjects underwent leukapheresis at four months post-CHMI, and all 11 subjects completed follow-up. Mock-immunization group: four subjects received the first, second, and third mock-immunizations. After the third mock-immunization, one subject was withdrawn due to an allergic reaction (S1 Appendix) and two underwent leukapheresis; three subjects then received the fourth and fifth mock-immunizations and all three subjects underwent CHMI. After CHMI, all three subjects underwent leukapheresis at day five or six post-CHMI and one subject underwent leukapheresis at four months post-CHMI. All three subjects completed follow-up. Infectivity controls: six non-immunized infectivity controls underwent CHMI and completed follow-up.

**Fig 1:**
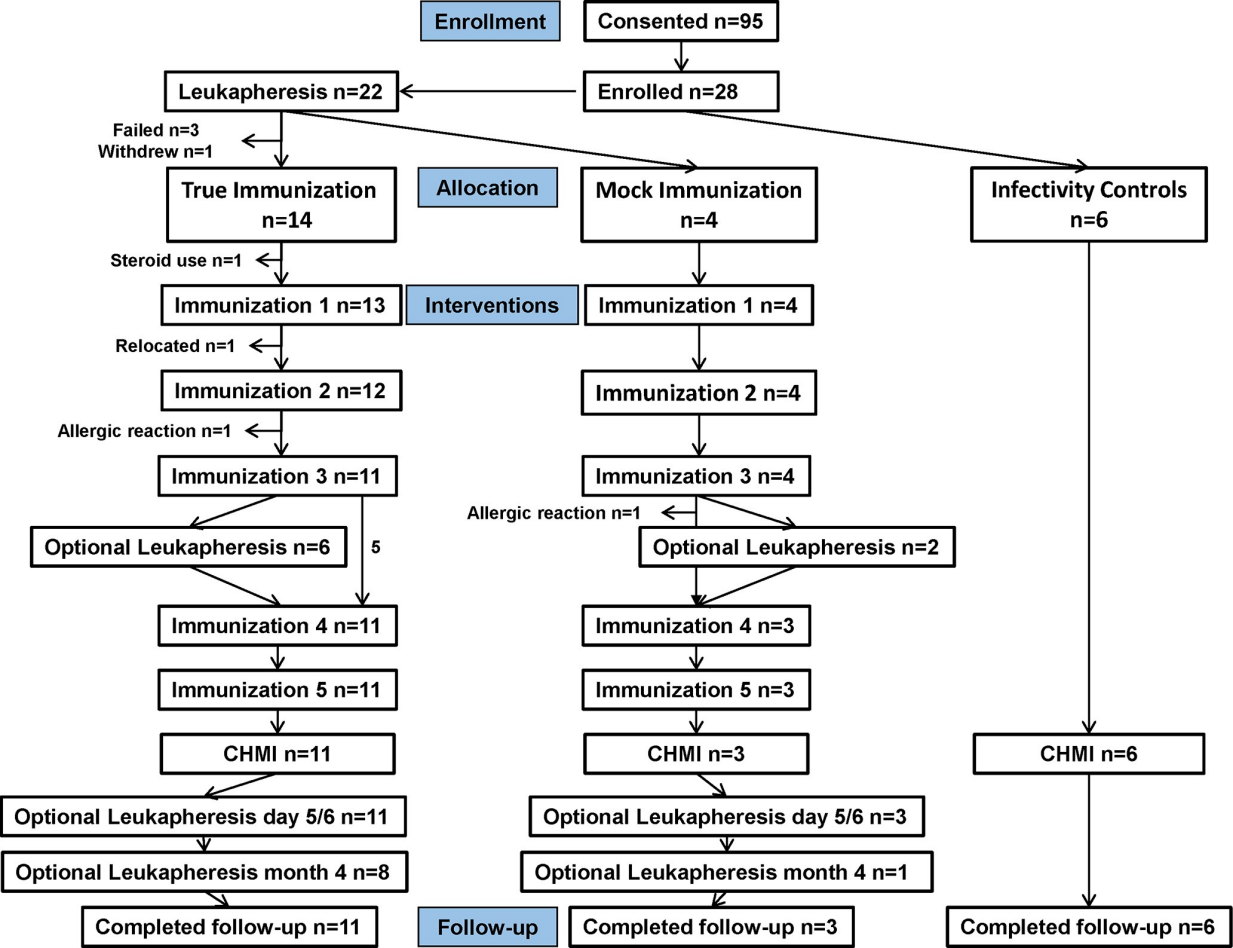
Cohort 1: Flow diagram of immunized and control subjects. Twenty-four subjects met all eligibility criteria and 14 were assigned to the true-immunization group, four were assigned to the mock-immunization group and the remaining six subjects were assigned as infectivity controls.

#### Cohort 2 (Fig 2)

True-immunization group: twelve subjects received the first true-immunization, and one subject was then withdrawn for an unrelated medical condition. Eleven subjects received the second and third true-immunizations, and after the third immunization one subject was withdrawn for an allergic reaction (S1 Appendix) and six subjects underwent leukapheresis. 10 subjects received the fourth and fifth true-immunizations and underwent CHMI. Nine subjects then underwent leukapheresis at days five or six post-CHMI, seven subjects underwent leukapheresis at four months post-CHMI, and all 10 subjects completed follow-up. Mock-immunization group: four subjects in the mock-immunization group received the first mock-immunization, one subject was then withdrawn for an unrelated medical condition (S1 Appendix) and one subject did not attend the second mock-immunization. Two subjects received the second mock-immunization; these two subjects and the subject who did not receive the second mock-immunization then received the third mock-immunization. After the third mock-immunization, one subject was withdrawn due to an allergic reaction (S1 Appendix), and one subject underwent leukapheresis and received the fourth mock-immunization. One subject who received the fourth mock-immunization and one subject who did not receive the fourth mock-immunization then received the fifth mock-immunization, and these two subjects underwent CHMI. Two subjects underwent leukapheresis five or six days post-CHMI, one subject underwent leukapheresis four months post-CHMI, and both subjects completed follow-up. Infectivity controls: six infectivity controls underwent CHMI and all completed follow-up.

**Fig 2:**
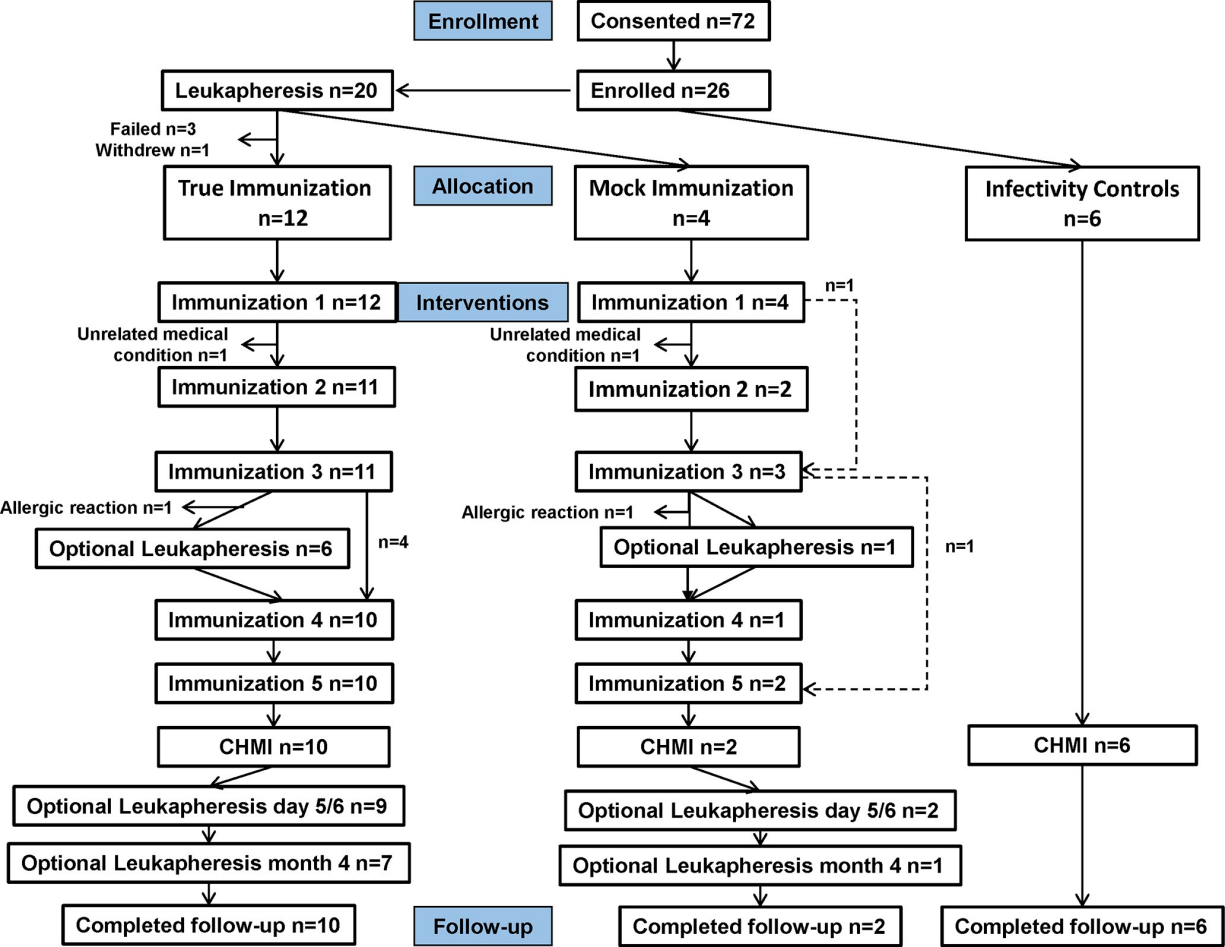
Cohort 2: Flow diagram of immunized and control subjects. Twenty-two subjects met all eligibility criteria and 12 were assigned to the true-immunization group, four were assigned to the mock-immunization group and the remaining six subjects were assigned as infectivity controls.

### Safety and tolerability

#### Solicited local and systemic adverse events (AEs)

The majority of immunizations were well tolerated and AEs were similar to those observed in previous PfRAS studies [12, 22]. All 58 true- and 18 mock-immunizations in Cohort 1, and 54 true and 12 mock-immunizations in Cohort 2, were included in the safety analysis. During the seven days following each immunization, 68 solicited local and systemic adverse events (AE) were recorded in both cohorts (Table 2); 33 AEs were recorded in Cohort 1 and 35 AEs were recorded in Cohort 2, and were generally similar among true- and mock-immunizations. The most frequent local AEs were vaccination site pruritus followed by erythema. Headache was the most common systemic AE.

**Table 2:** Solicited adverse events occurring within 7 days of any immunization for all subjects receiving at least one immunization.

	Cohort 1						Cohort 2					
	True-immunized n = 13			Mock-immunized n = 4			True-immunized n = 12			Mock-immunized n = 4		
	Grade 1	Grade 2	Grade 3	Grade 1	Grade 2	Grade 3	Grade 1	Grade 2	Grade 3	Grade 1	Grade 2	Grade 3
**Local AE**												
Lymphadenopathy	0	0	0	0	0	0	1 (8%)	0	0	0	0	0
Vaccination site erythema	1 (8%)	1 (8%)	2 (15%)	2 (50%)	0	0	2 (18%)	0	1 (8%)	3 (75%)	1 (25%)	0
Vaccination site induration	0	0	0	1 (25%)	0	0	1 (8%)	1 (8%)	0	0	1 (25%)	0
Vaccination site pain	0	0	1 (8%)	1 (25%)	0	0	0	0	0	0	0	0
Vaccination site pruritus	3 (23%)	1 (8%)	0	0	0	0	6 (50%)	1 (8%)	0	1 (25%)	0	0
Vaccination site swelling	1 (8%)	0	0	1 (25%)	0	1 (25%)	1 (8%)	0	1 (8%)	1 (25%)	1 (25%)	0
Vaccination site urticaria	0	0	0	1 (25%)	0	0	0	0	0	0	0	0
**Systemic AE**												
Back pain	0	0	0	0	0	0	0	1 (8%)	0	0	0	0
Dermatitis allergic	0	0	0	0	0	0	0	1 (8%)	0	0	0	0
Diarrhea	1 (8%)	0	0	0	0	0	0	0	0	0	0	0
Fatigue	1 (8%)	0	0	0	0	0	0	0	0	0	0	0
Headache	2 (15%)	0	0	0	0	0	1 (8%)	0	0	0	0	0
Hypersensitivity	0	0	1 (8%)	0	0	0	0	0	0	0	0	0
Malaise	0	0	0	0	0	0	1 (8%)	0	0	0	0	0
Myalgia	1 (8%)	0	0	0	0	0	1 (8%)	0	0	0	0	0
Nausea	0	0	0	0	0	0	0	0	0	1 (25%)	0	0
Pyrexia	0	0	0	0	0	0	1 (8%)	0	0	0	0	0
Urticaria	0	0	0	0	0	1 (25%)	00	0	0	0	0	1 (25%)
^**1**^ **Number of Subjects with at Least One Event**												
	3 (13%)	2 (15%)	2 (15%)	2 (50%)	0	1 (25%)	7 (58%)	1 (8%)	1 (8%)	3 (75%)	1 (25%)	1 (25%)
**Total Number of Events by Severity**												
	**Grade 1**	**Grade 2**	**Grade 3**	**Grade 1**	**Grade 2**	**Grade 3**	**Grade 1**	**Grade 2**	**Grade 3**	**Grade 1**	**Grade 2**	**Grade 3**
	17 (71%)	3 (13%)	4 (17%)	7 (78%)	0	2 (22%)	19 (76%)	4 (16%)	2 (8%)	6 (60%)	3 (30%)	1 (10%)

Summary is limited to subjects who received at least one immunization. Only those solicited adverse events which followed non-boost vaccinations are included. At each level of subject summarization, a subject who reported one or more such events was counted once for the most severe event.

^1^Number (%) of subjects who had an AE.

In Cohort 1 among the true-immunizations (Table 2), 17 (71%) were mild (Grade 1), three (13%) were moderate (Grade 2), and four (17%) were severe (Grade 3). The four Grade 3 events were reported in two subjects: vaccination site erythema (two cases), vaccination site pain, and systemic allergic type reaction. Among the mock-immunizations seven (78%) were Grade 1; there were no Grade 2 AEs and two (22%) were Grade 3, vaccination site swelling and systemic urticaria reported in one subject. This distribution was similar in Cohort 2 (Table 2); among true-immunizations 19 (76%) were Grade 1, four (16%) were Grade 2, and two (8%) were Grade 3, vaccination site erythema and swelling reported in one subject; among mock-immunizations six (60%) were Grade 1, three (30%) were Grade 2 and one (10%) was Grade 3, systemic urticaria.

One serious adverse event was reported: one subject in the mock-immunized group suffered an exacerbation of asthma with hyperglycemia 4 weeks after a single mock-immunization, believed secondary to prescribed steroid use (Subject #126, S1 Appendix) and was withdrawn from the study.

Two true and two mock immunized subjects were withdrawn due to large local reactions at the bite site on the forearm (S1 Appendix). All four also had transient systemic reactions consisting of pruritis, hives and/or erythematous rash, three of which were Grade 3 in severity as noted above. More information is provided on these four reactions in the S1 Appendix. A consultant in allergy and immunology posited IgE-mediated allergic reactions to mosquito antigens (not to parasite antigens), as the reactions were similar in all four subjects and the two mock immunized subjects had no exposure to malaria parasites. This distribution is similar to our previous study using RAS-immunization [12], which reported that 1 of 16 true-immunized subjects and 1 of 9 mock-immunized subjects who received at least 2 immunizations developed large local reactions which a consultant noted were consistent were IgE-mediated reactions to mosquito salivary antigens. As part of a first-in-humans trial with immunization by the bites of mosquitoes carrying genetically attenuated sporozoites, 6 subjects received an immunization with approximately 200 mosquitoes per subject and there were no large (Grade 3) local reactions with that exposure [23].

Solicited adverse events (local and systemic) were compared after each true- and mock-immunization (S1 Table in S1 Appendix). Numbers of AEs after true immunizations declined progressively with sequential immunizations and were predominantly Grade 1, whereas numbers of AEs after mock-immunizations, though fewer, remained unchanged during the regimen except after the 4^th^ immunization when none were recorded.

#### Unsolicited adverse events (AEs)

During the 14 days following any immunization, 71 unsolicited AEs were recorded (S3 Table in S1 Appendix) in both cohorts after true- and mock-immunizations (S1 Appendix).

#### Laboratory adverse events (AEs)

There were no Grade 3 or Grade 4 lab abnormalities reported within 7 days of any immunization (Table 3). In Cohort 1, 3/13 subjects (23%) had Grade 2 decreased hemoglobin and 3/13 subjects (23%) had Grade 2 decreased lymphocytes. In Cohort 2, Grade 2 lab abnormalities were reported in one subject with a Grade 2 elevated alanine transaminase (ALT) and one subject with a Grade 2 elevated aspartate transaminase (AST). Eleven of the 25 true-immunized subjects (44%) had at least one elevated ALT result (with a single subject in Cohort 2 having a Grade 2 ALT elevation) as compared to 4 of the 8 (50%) mock-immunized subjects having at least one abnormal ALT. Nine of 25 (36%) true-immunized subjects experienced at least one elevated AST (with two subjects in Cohort 1 and one subject in Cohort 2 having a Grade 2 result). In comparison there were two of eight (25%) mock-immunized subjects (both in Cohort 1) having an elevated AST (one of these being a Grade 2 result).

**Table 3:** Summary of post-immunization laboratory abnormalities by parameter and grade for subjects in both cohorts by true and mock groups over all five immunizations.

	Cohort 1				Cohort 2			
	True Immunized *N = 13*		Mock Immunized *N = 4*		True Immunized *N = 12*		Mock Immunized *N = 4*	
*Lab test (units)*	*Grade 1 n (%)*	*Grade 2 n (%)*	*Grade 1 n (%)*	*Grade 2 n (%)*	*Grade 1 n (%)*	*Grade 2 n (%)*	*Grade 1 n (%)*	*Grade 2 n (%)*
**Any immunization**								
ALP (U/L)	1 (7.7)	0	1 (25)	0	0	0	0	0
ALT (U/L)	6 (46.2)	0	2 (50)	1 (25)	4 (33.3)	1 (8.3)	1 (25)	0
AST (U/L)	3 (23.1)	2 (15.4)	1 (25)	1 (25)	3 (25)	1 (8.3)	0	0
BUN (mg/dL)	4 (30.8)	2 (50)	2 (50)	0	1 (8.3)	0	2 (50)	0
Creatinine (mg/dL)	3 (23.1)	1 (7.7)	2 (50)	0	3 (25)	0	2 (50)	0
Eosinophils (cells/uL)	1 (7.7)	1 (7.7)	0	0	2 (16.7)	0	0	0
Hemoglobin (g/dL)	0	3 (23.1)	2 (50)	0	0	0	0	0
Lymphocytes (cells/uL)	3 (23.1)	3 (23.1)	1 (25)	3 (75)	2 (16.7)	0	0	0
Neutrophils (cells/uL)	3 (23.1)	2 (15.4)	1 (25)	1 (25)	0	0	0	0
Platelets (10x3/uL)	1 (7.7)	2 (15.4)	1 (25)	0	0	0	0	0
Total bilirubin (mg/dL)	2 (15.4)	0	0	0	1 (8.3)	0	0	0
WBC (10x3/uL)	5 (38.5)	1 (7.7)	1 (25)	1 (25)	3 (25)	0	0	0

Column header counts and denominators are the number of subjects who received at least one immunization.

Subjects with at least one abnormal lab within 7 days of any immunization are presented.

A subject is counted once per row.

No Grade 3 or 4 severity lab abnormalities were reported.

### Immunization procedures–Cumulative infected bites

**Fig 3:**
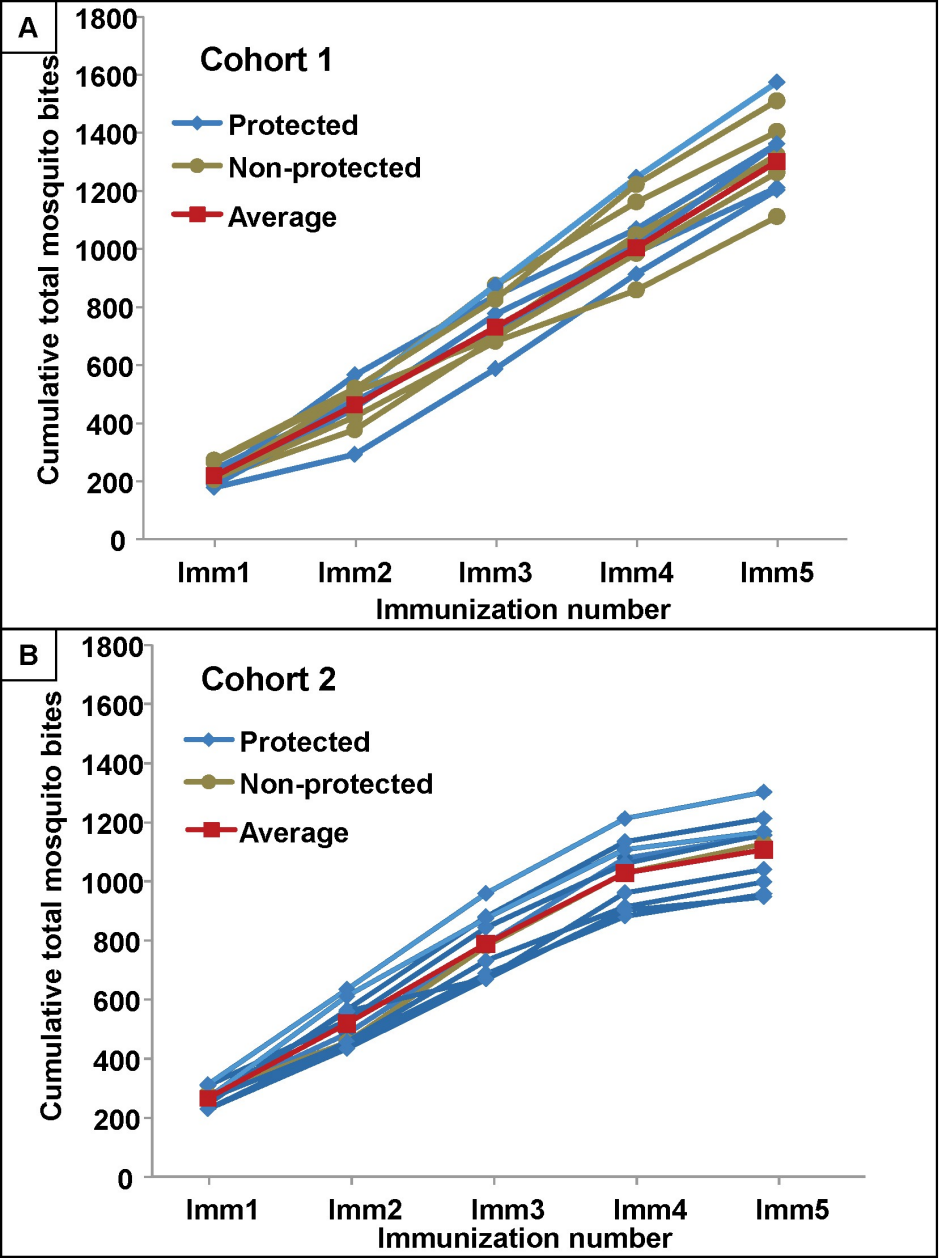
Cumulative numbers of bites in Cohort 1 and Cohort 2. The cumulative numbers of *P*. *falciparum* RAS-infected mosquitoes administered at immunizations 1–5 for each protected and non-protected subjects and the average cumulative total for all subjects. **Panel A:** Cohort 1. **Panel B:** Cohort 2.

Cohort 1 received a mean of 1027 infected bites (Fig 3A) and Cohort 2 received a mean of 936 infected bites (Fig 3B) administered in five immunizations. However, in Cohort 2, the cumulative total after four immunizations (913 bites) exceeded that of Cohort 1 after four immunizations (790 bites) and already met the 800–1200 target established before the start of this study; therefore the fifth and final immunization was reduced to a mean of 73 bites per subject compared to 237 for Cohort 1.

### Protective efficacy and time to parasitemia

**Fig 4:**
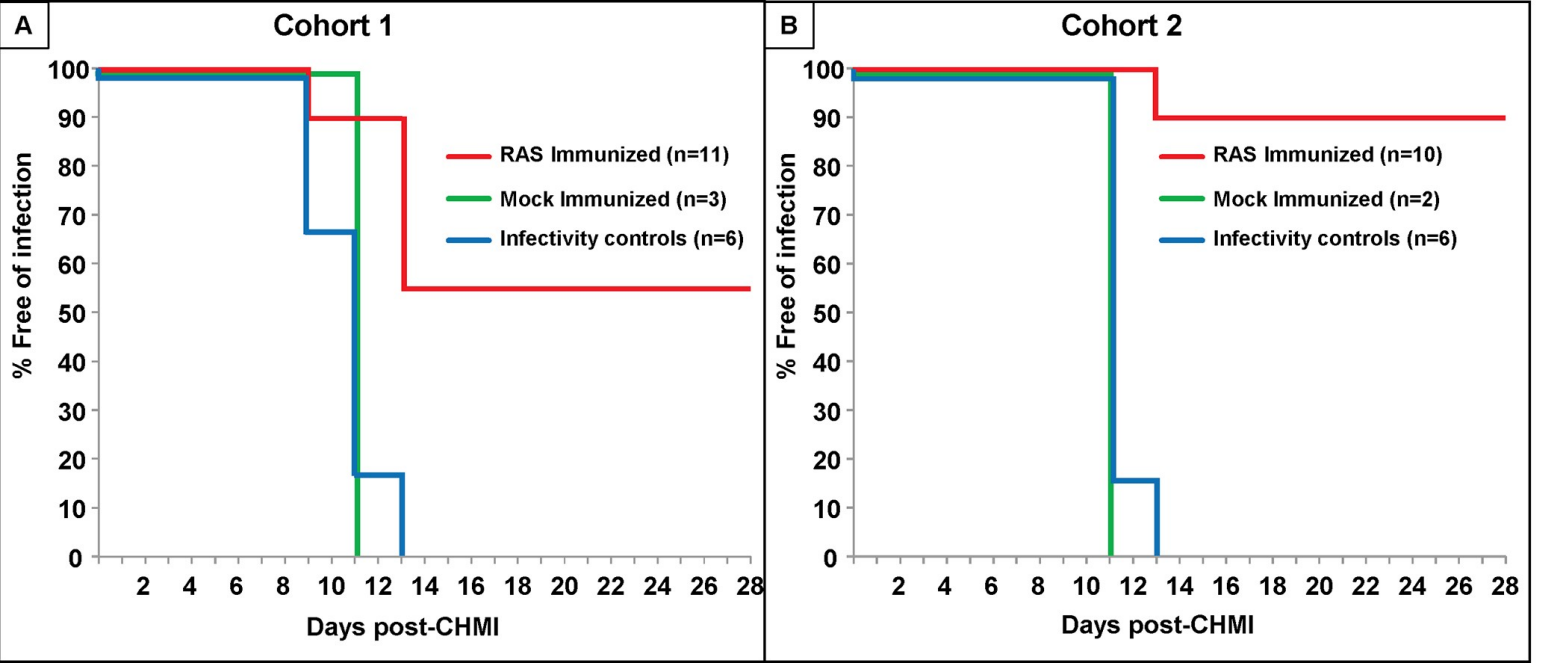
Development of parasitemia in the true- and mock-immunized and infectivity control subjects. Parasitemia-free survival curves (Kaplan-Meier) for true- and mock-immunized subjects and infectivity controls based on microscopic examination of peripheral blood smears. **Panel A:** Cohort 1 where 11 true-immunized, 3 mock-immunized and 6 infectivity controls received CHMI. **Panel B:** Cohort 1 where 10 true-immunized, 2 mock-immunized and 6 infectivity controls received CHMI.

**Cohort 1**: eleven subjects completed all five true-immunizations, three subjects completed all five mock-immunizations, and all 14 subjects received CHMI together with six infectivity controls 3 weeks (Fig 4A). Six of eleven true-immunized subjects were sterilely protected after CHMI (55%), and time to parasitemia of the remaining five subjects was between 9–13 days after CHMI (9, 11, 11, 11, 13 days, mean 10.67 days). The established definition of significant delay of time to parasitemia [16] is where the time to parasitemia of subjects in the vaccine group is greater than the mean of controls + 2 standard deviations (SD). Because mock-immunized and infectivity controls showed similar curves to parasitemia (Fig 4A) and there were only two mock-immunized subjects, we combined both controls. The combined mock-immunized and infectivity controls became parasitemic between 9–13 days after CHMI (9, 9, 11, 11, 11 11,11, 13 days, mean 10.75 days; SD = 1.28 days; mean+2SD = 13.31 days). Therefore, the times to parasitemia of the non-protected subjects were not significantly delayed compared to the combined mock immunized and infectivity controls.

**Cohort 2**: ten subjects completed all five true-immunizations, two subjects completed all five mock-immunizations, and all 12 subjects received CHMI together with six infectivity controls 3 weeks after immunization (Fig 4B). Nine of 10 true immunized subjects were sterilely protected after CHMI (90%) and the time to parasitemia of the non-protected subject was 13 days. The combined mock-immunized and infectivity controls became parasitemic between 11–13 days after CHMI (11, 11, 11 11, 11, 11, 11, 13 days, mean 11.25 days; SD = 0.71 days; mean+2SD = 12.66 days). Therefore, the time to parasitemia of the non-protected subject was not significantly delayed compared to the combined mock immunized and infectivity controls.

### Comparison of immunization procedures in cohort 1 and cohort 2

**Fig 5:**
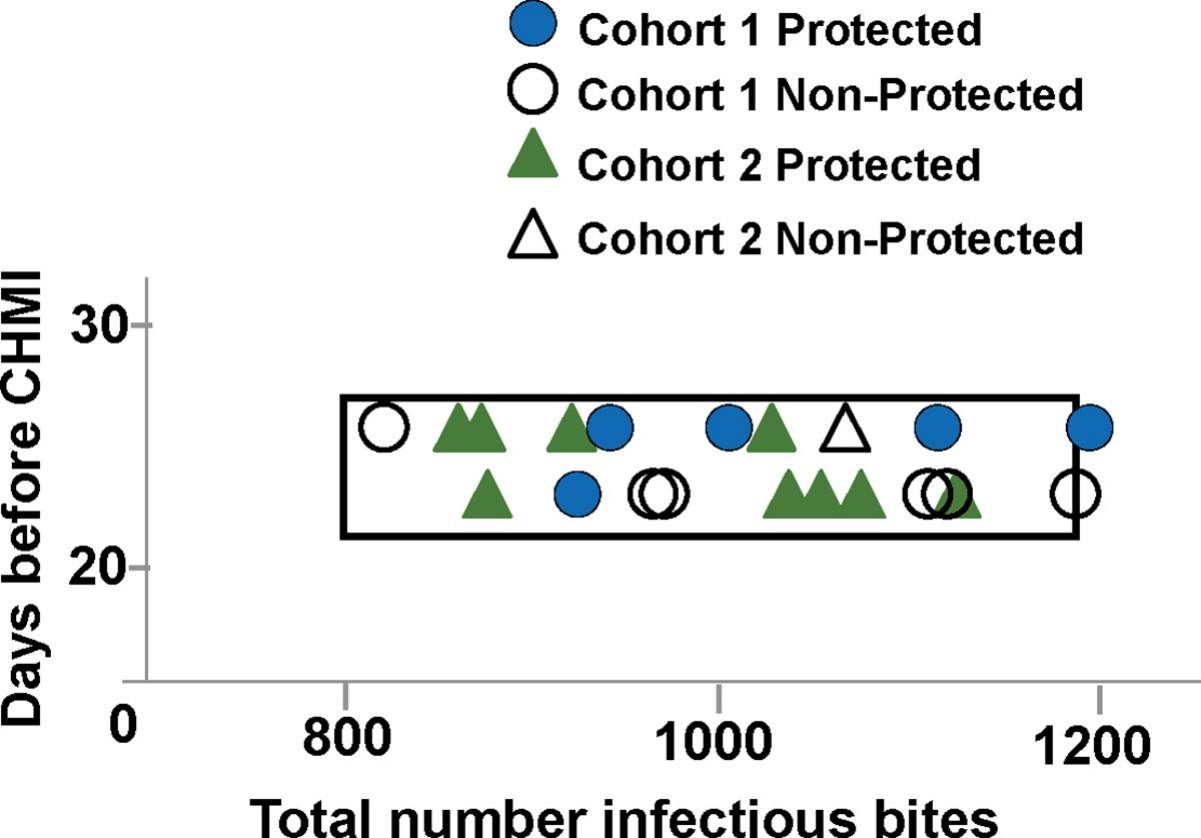
Comparison of total number of infectious bites and time to CHMI in Cohort 1 and Cohort 2 with the target range of infectious bites. The total number of infectious bites and days between final immunization and CHMI are compared for Cohort 1 (protected: blue circles; non-protected: open circles) and Cohort 2 (protected: green triangles; non-protected: open triangles). All Cohort 1 and Cohort 2 subjects fell within the target box except one protected subject and one non-protected subject in Cohort 1 that fell just outside the target box. The distribution of subjects in Cohort 1 and Cohort 2 showed no association of protection with total numbers of infectious bites or time (days) before CHMI.

The distributions of sterilely protected and non-protected subjects in Cohorts 1 and 2 are consistent with previous protective efficacy results and met our experimental objectives of 800–1200 infected bites and an interval of 22–24 days before CHMI (Fig 5).

Cohort 1: Although the study was not designed to determine whether protection was associated with any single immunization parameter, differences between the sterilely protected and non-protected true immunized subjects were examined, given the apparent difference between the two cohorts. There were no differences in total number of bites, total number of infected bites, median bites per immunization, number of bites in the 1^st^, 2^nd^– 5^th^ immunizations, duration of immunization, or gland scores between the six protected and five non-protected subjects (Table 4, S4 Table–S9 Table in S1 Appendix and Fig 3A).

**Table 4:** Cohort 1—Summary of true-immunizations: Mosquito bites, gland scores and duration of immunizations.

	Cohort 1					
	All		Protected		Non-protected	
Number of Subjects	11		6		5	
	Median	Range	Median	Range	Median	Range
Total all bites^1^	1323	1111–1574	1297	1203–1574	1323	1111–1510
Total infected bites^1^	1007	810–1235	1015	917–1235	962	810–1221
Median bites per immunization^1^	209	83–309	203	83–309	211	107–303
Bites 1^st^ immunization^2^	175	148–264	172	148–187	180	158–264
Bites 5^th^ immunization^2^	239	186–272	266	186–272	227	211–248
Bites 2^nd^-4^th^ immunization^2^	209	83–309	212	83–309	195	107–303
Duration immunizations (days)	119	116–119	119	116–119	119	119–119
Interval before CHMI (days)	22	22–24	24	22–24	22	22–24
Gland grade score during immun.^4^	3.2	3.0–3.3	3.2	2.9–3.3	3.3	3.0–3.3
Gland score^3^ 1^st^ immun.	3.3	3.1–3.6	3.3	3.1–3.5	3.3	3.1–3.6
Gland score 5^th^ immun.	3.2	3.0–3.4	3.2	3.0–3.4	3.3	3.2–3.3
Gland score 2^nd^-4^th^ immun.^4^	3.1	3.0–3.3	3.1	2.9–3.3	3.2	3.0–3.3
Gland score CHMI	3.6	2.8–3.8	3.4	2.8–3.8	3.6	3.6–3.8

The median and range of the immunizations are shown of all subjects in Cohort 1 who underwent CHMI. Comparisons between protected and non-protected subjects revealed no statistically differences (p = >0.05).

^1^Median of the numbers of infected bites at all immunizations.

^2^Median of the numbers of infected bites for each subject at the first, fifth, and second-fourth immunizations.

^3^Gland score: as defined in Methods, salivary infection rates were defined as gland scores: 1–10 sporozoites = gland score 1; 11–100 sporozoites = gland score 2; 101–1,000 sporozoites = gland score 3; and > 1,000 = gland score 4.

^4^Median gland scores during immunization are the medians of the mean gland scores of all subjects at each immunization; ranges are means for that immunization.

Cohort 2: Since nine of ten subjects were sterilely protected, it was not possible to do a meaningful comparative analysis of the protected and non-protected subjects (Table 5, Fig 3B). However, the cumulative numbers of bites were similar to Cohort 1, except the numbers of bites in the first true immunization were larger and in the fifth true immunization were smaller than in Cohort 1 (Table 6, S4 Table-S9 Table in S1 Appendix and Fig 3). When the immunization procedures for all subjects in Cohort 1 and 2 were compared, there were statistically significant differences in several procedures between each Cohort (Table 6).

**Table 5:** Cohort 2—Summary of true-immunizations: mosquito bites, gland scores and duration of immunizations.

	Cohort 2				
	All		Protected		Non-Protected
Number of Subjects	10		9		1
	Median	Range	Median	Range	Actual^4^
Total all bites^1^	1136	976–1333	1186	976–1333	1158
Total infected bites^1^	1026	839–1131	1022	839–1131	1038
Median bites per immunization^1^	220	77–270	221	77–251	218
Bites 1^st^ immunization^2^	227	194–249	225	194–249	242
Bites 5^th^ immunization^2^	77	41–96	76	41–96	86
Bites 2^nd^-4^th^ immunization^2^	221	191–3271	218	192–266	237
Duration immunizations (days)	119	119–119	119	119–119	119
Interval before CHMI (days)	23	22–24	22	22–24	24
Gland score^3^ during immun.	3.6	2.9–3.9	3.6	2.9–3.9	3.7
Gland score 1^st^ immun.	3.4	2.9–3.6	3.4	2.9–3.6	3.5
Gland score 5^th^ immun.	3.7	3.5–3.9	3.7	3.5–3.9	3.7
Gland score 2^nd^-4^th^ immun.	3.6	3.4–3.9	3.6	3.3–3.9	3.8
Gland score CHMI	3.5	3.0–3.8	3.4	3.0–3.8	3.6

The median and range of the immunizations are shown of all subjects in Cohort 2 who underwent CHMI. Since only one subject was non-protected, it was not possible to reliably do a statistical comparison.

^1^Median of the numbers of bites at each immunization.

^2^Median of the numbers of bites for each subject at the first, fifth, and second-fourth immunizations.

^3^Gland score: as defined in Methods, salivary infection rates were defined as gland scores: 1–10 sporozoites = gland score 1; 11–100 sporozoites = gland score 2; 101–1,000 sporozoites = gland score 3; and > 1,000 = gland score 4. Median gland scores during immunization are the medians of median gland scores for each subject; ranges are values for each subject at each immunization.

^4^Median gland scores during 1^st^ immunization and 5^th^ immunizations and CHMI are the medians of gland scores for each subject; ranges are values for each subject at that immunization or CHMI.

^5^Actual values for one non-protected subject.

**Table 6:** Cohorts 1 and 2—Comparison of key parameters of true-immunizations.

	Cohort 1		Cohort 2		
Number of Subjects	11		10		p
	Median	Range	Median	Range	
Total all bites	1323	1111–1574	1136	976–1333	**0.004**
% mosquitoes infected	80	70–87	88	83–93	**0.001**
Total infected bites	1007	810–1235	1026	839–1131	0.65
Median bites per immun.	210	175–239	220	77–270	0.4
Bites 1^st^ immun.	175	148–264	227	194–249	**0.005**
Bites 5^th^ immun.	239	186–272	77	41–96	**0.0001**
Bites 2^nd^-4^th^ immun.	210	175–210	221	191–3271	0.2
Duration immun. (days)	119	119–119	119	119–119	1
Interval before CHMI (days)	22	22–24	23	22–24	1
Gland score during immun.	3.2	2.7–3.7	3.6	2.9–3.9	**0.02**
Gland score 1^st^ immun.	3.3	3.1–3.6	3.4	2.9–3.6	0.75
Gland score 5^th^ immun.	3.2	3.0–3.4	3.7	3.5–3.9	**0.0001**
Gland score 2^nd^-4^th^ immun.	3.2	2.7–3.7	3.6	3.4–3.9	**0.0001**
Gland score CHMI	3.6	2.8–3.8	3.5	3.0–3.8	0.65

Summaries (mean, range) of immunizations in Cohorts 1 and 2 were compared using the Mann Whitney U test, where significance is p = ≤0.05.

Number of bites: since the infection rate of mosquitoes used in Cohort 1 was significantly lower than Cohort 2, significantly more bites (infected and non-infected) were administered in Cohort 1 than Cohort 2 (Table 6, Fig 3). However, in Cohort 2, the median number of infected bites in the first immunization was significantly higher (p = 0.005) and in the fifth immunization was significantly lower (p = 0.0001) compared to Cohort 1. The median numbers of bites were similar for the second to fourth immunizations for both cohorts. This may suggest that the increased efficacy in Cohort 2 is associated with the higher numbers of first immunization bites and/or the reduced numbers of fifth immunization bites.

Salivary gland scores (Table 6): the median salivary gland scores of all immunizations in Cohort 2 were significantly higher (p = 0.02) than Cohort 1. The salivary gland scores of Cohort 1 and Cohort 2 during the first immunization were not statistically different, but were higher in Cohort 2 were than Cohort 1 for immunizations two through five (p = 0.0001). Others have suggested that mosquitoes with higher salivary gland loads are more infectious, reasoning that more sporozoites are injected during each bite [24]. It is therefore possible that infected mosquitoes with higher gland loads used in Cohort 2 may have delivered more sporozoites resulting in higher protective efficacy. The salivary gland scores were similar for the CHMI mosquitoes in Cohort 1 and Cohort 2.

### Effect of leukapheresis and protection

An optional leukapheresis was performed in both Cohorts at 14 days after the third immunization (Figs 1 and 2) but did not appear to affect protection. Of the six subjects that were leukapheresed in Cohort 1, three were later protected and three were non-protected; of the six subjects that were leukapheresed in Cohort 2, five were later protected and one was non-protected.

### Comparison of cohort 1 and cohort 2 immunization procedures with prior studies

The variation in VE from 55% in Cohort 1 to 90% in Cohort 2 was similar to that seen in two prior RAS trials conducted by NMRC, which had protected 50% of subjects (1999–2002 trial) and 90% of subjects (1989–1999 trial) respectively [12]. The immunization parameters for the three trials were compared to look for associations with protection (Table 7). The time to CHMI after last immunization was less than 28 days in most cases, and did not appear to play a role in the different outcomes. Similarly, the total number of infectious bites did not appear to be associated with protection, with both protected and non-protected subjects predominantly receiving more than 975 infected bites. However, there were differences among the trials in the number of infected bites in the fifth (or final) immunizations: 77 in Cohort 2 (90% VE) and 131 in the 1989–1999 trial (90% VE), compared to 239 in Cohort 1 (VE 55%) and 186 in the 1999–2002 trials (50% VE), suggesting that the higher efficacy in Cohort 2 and the 1989–1999 trials was associated with lower numbers of infected bites in the final immunizations.

**Table 7:** Comparison of key parameters of Cohorts 1 and 2 with 1989–1999 and 1999–2002 trials.

Trial	1989–1999	1999–2002	Cohort 1	Cohort 2
Vaccine Efficacy %	90%	50%	55%	90%
Median total infected bites	1092 (1001–1163)	1247 (1005–1561)	1007 (810–1235)	1026 (839–1131)
Median bites per immun.	125 (109–210)	214 (175–260)	210 (175–238)	220 (77–270)
Median bites 1^st^ immun.	148 (130–210)	224 (138–334)	175 (148–264)	227 (194–249)
Median bites 5^th^ or final immun.	131 (67–147)	186 (127–252)	239 (186–272)	77 (41–96)
Number of immun.	8.5 (5–10)^1^	6 (5–6)^1^	5	5
Duration immun. (days)	242 (99–547)^1^	206 (175–239)^1^	119 (119–119)	119 (119–119)
Median immunization interval (days)	31 (23–77)	44 (35–48)	28 (28–34)	28 (28–34)
Median interval before CHMI (days)	16 (14–71)	35 (15–42)	22 (22–24)	23 (22–24)
Median gland score during immun.	3.2 (3.0–3.7)	3.5 (3.2–3.9)	3.2 (2.7–3.7)	3.6 (2.9–3.9)
Median gland score CHMI	3.2 (2.8–3.4)	2.7 (2.4–3.2)	3.6 (2.8–3.8)	3.5 (3.0–3.8)

^1^ Studies in 1989–1999 and 1999–2002 used infected mosquitoes when available, and therefore the schedule and numbers of infectious bites varied among subjects.

### Hyperimmunity continuation phase cohort

Three subjects from Cohort 1 who received all five immunizations and were sterilely protected against CHMI received three additional immunizations at 28-day intervals beginning at 158 days after their fifth immunizations. Infected bites were similar (number of bites, gland score/immunization) to their preceding immunizations, and were well tolerated, with all local and systemic solicited AEs and laboratory abnormalities reported within 7 days of immunization were Grade 1 in severity. The three subjects received CHMI 87 days after their final immunization, and each was sterilely protected, suggesting that protection could be extended with boosting immunizations to at least 67 weeks after the first series of immunizations and at least 12 weeks after the final boosting immunization.

### Immunogenicity

Antibody and cellular assays were performed to look for differences between Cohort 1 and Cohort 2.

#### *Ex vivo* fluoroSpot (IFN-γ, IL2, IFN-γ+IL2)

**Fig 6:**
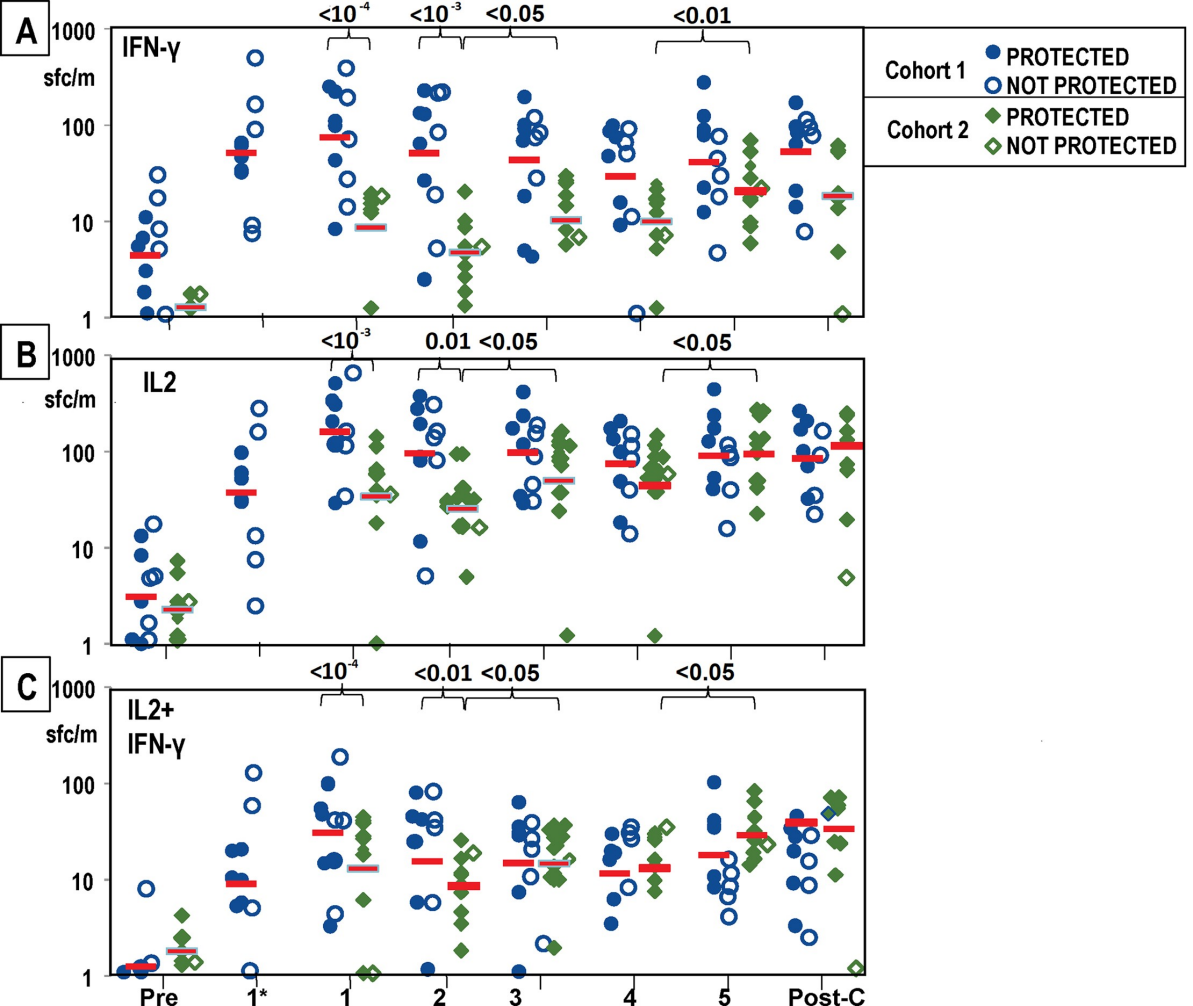
Cohort 1 and Cohort 2: FluoroSpot activities to whole sporozoites after each immunization and CHMI. FluoroSpot IFN-γ, IL2 and IFN-γ+IL2 activities to whole sporozoites were measured pre-immunization (Pre), after each immunization (1, 2, 3, 4, 5) and after CHMI (Post-C); subjects in Cohort 1 only were also measured 7 days after the first immunization (1*). Cohort 1 and Cohort 2 protected and non-protected subjects are shown by indicated symbols. Geometric means are indicated by red bars. **Panel A: IFN-γ:** Geometric mean activities in both Cohorts significantly rose (p = <0.001) after the first immunization and were higher in Cohort 1 than Cohort 2 after the first and second immunizations (see text). In Cohort 2, activities significantly rose after the fifth immunization. **Panel B: IL2:** Geometric mean activities in both Cohorts significantly rose (p = <10^−3^) after the first and second (p = <0.01) immunizations and activities of Cohort 1 were significantly higher than Cohort 2 after the first and second immunizations (see text). In Cohort 2, activities significantly rose after the third and fifth immunizations. **Panel C: IFN-γ+IL2:** Geometric mean activities in both Cohorts 1 were significantly higher after the first immunization, and activities of Cohort 1 were higher than Cohort 2 after the first and second immunizations (see text). In Cohort 2, activities significantly rose after the third and fifth immunizations.

Activities (sfcs/million PBMC) measured after stimulation with cryopreserved sporozoites are shown in Fig 6. The antigens associated with protection induced by RAS have not been identified, justifying the selection of sporozoites as a stimulant. Sporozoites express hundreds of antigens, some of which are carried into hepatocytes during infection and some of which are further expressed in liver stage parasites. We have previously successfully used cryopreserved Pf sporozoites in analyses of RAS responses [12, 17, 22, 25, 26].

**IFN-γ activities:** Using a linear model to analyze time point and cohort effects, we observed that geometric mean activities rose significantly across all immunized subjects (p = < 0.0001) at 28 days after the first immunization (Fig 6A), followed by a modest decline, before increasing again after the third and fifth immunizations in Cohort 2 (p < 0.05 and p < 0.01, respectively). We found that responses in Cohort 1 were significantly higher than in Cohort 2 after the first (p = <10^−4^) and second (p = <10^−3^) immunizations.

**IL2 activities:** We found that IL2 activities were significantly higher (p = < 10^−6^) at 28 days after the first immunization across all immunized subjects (Fig 6B). We found that responses in Cohort 1 were significantly higher than Cohort 2 at 28 days after the first immunization (p < 10^−3^) and 28 days after the second immunization (p < 0.01). In Cohort 1, IL2 activities were higher than IFN-γ activities at all time points, although the differences were not significant, and peaked after the first immunization and declined thereafter. There were no apparent differences between protected and non-protected subjects. In Cohort 2 (Fig 6B): IL2 activities were similar to IFN-γ activities and the difference in IL2 activities between Cohort 1 and 2 was greatest after the first and second immunization and then gradually diminished after the second immunization through the post-challenge time point. As in IFN-γ activities, IL2 activities significantly rose after the third and fifth immunizations in Cohort 2 (p < 0.05 and p < 0.05, respectively).

**IFN-γ+IL2 activities:** We found that IFN-γ+IL2 activities were relatively low across all time points, but were significantly higher at 28 days after the first immunization than at other timepoints. We found that IFN-γ+IL2 activities in Cohort 1 were higher than in Cohort 2 after the first and second immunization (p < 10^−4^ and p < 0.01, respectively), and then gradually decreased over time to the post-challenge time point. As in IFN-γ activities, IFN-γ +IL2 activities significantly rose after the third and fifth immunizations in Cohort 2 (p < 0.05 and p < 0.05, respectively).

#### Antibody responses

*Enzyme-linked immunosorbent assay (ELISA)*. There were no responses to CelTOS in any of the participants.

**Fig 7:**
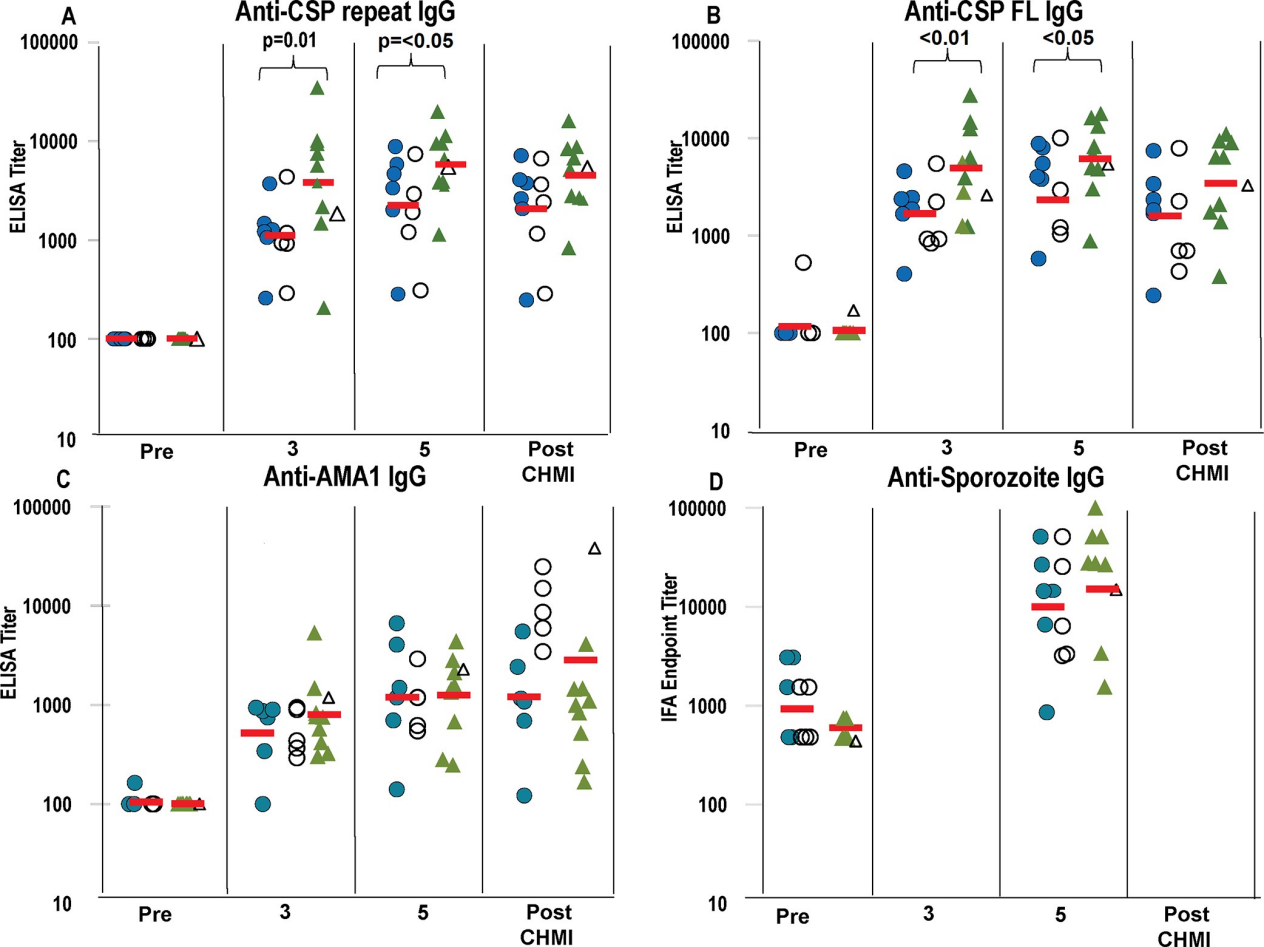
Cohort 1 and Cohort 2: ELISA activities after third and fifth immunizations and CHMI, and IFA activities after fifth immunization and CHMI. ELISA activities to CSP repeat, CSP full length (FL) and AMA1 of Cohort 1 (blue circles) and Cohort 2 (green triangles) were measured pre-immunization (Pre), 14 days after the third (3) and 22 days after fifth (5) immunizations and 28 days after CHMI (Post-CHMI). IFA activities to sporozoites were measured pre-immunization (Pre), and 22 days after fifth (5) immunizations / pre-CHMI. Protected subjects (closed symbols) and non-protected subjects (open symbols). The Geometric mean (red bar) is shown for all subjects in each Cohort. **Panel A: CSP repeat**: Geometric mean activities in both Cohorts significantly rose after the third and fifth immunizations (see text). Activities in Cohort 2 were significantly higher than Cohort 1 after the third and fifth immunizations. **Panel B: CSP Full Length (FL):** activities were similar to those with CSP repeat and activities of Cohort 2 were only significantly higher than Cohort 1 after the third and fifth immunizations. **Panel C: AMA1**: Geometric mean activities of Cohort 1 and Cohort 2 significantly rose after the third and fifth immunization (see text) but were not different between Cohort 1 and Cohort 2. **Panel D IFA**: Activities of Cohort 1 and Cohort 2 significantly rose after the fifth immunizations (see text) but were similar in Cohort 1 and Cohort 2.

CSP (Fig 7A and 7B). Across all immunized subjects, the geometric mean activities to CSP repeat and full length of all immunized subjects rose significantly (p < 10^−5^) after the third immunization, rose significantly after the fifth immunization (p < 0.05) and were unchanged after CHMI. There was no apparent difference between protected and non-protected subjects. Using a linear model, we found that CSP responses in Cohort 2 were significantly higher than in Cohort 1 for both the repeat region and full-length CSP across after both the third and fifth immunization (p < 0.01 and p < 0.05, respectively).

AMA (Fig 7C). We found that AMA1 responses of all immunized subjects rose significantly (p = <0.001) after the third immunization, and rose again significantly (p = 10^−2^) after the fifth immunization. We observed no significant differences with respect to cohort across the post-immunization time points. In Cohort 1, after CHMI, activities of the protected subjects were unchanged but were much higher in the five non-protected subjects, consistent with development of blood stage parasitemia in these subjects, as AMA1 is expressed in blood as well as pre-erythrocytic stages. In Cohort 2, after CHMI, activities of the protected subjects were unchanged although activity of the one non-protected subject was much higher.

*Immunofluorescence Antibody Assay (IFA)*. IFA activities of Cohort 1 and Cohort 2 (Fig 7D) rose significantly after the third and fifth immunization compared to pre-immunization (p < 10^−3^ and p < 10^−4^, respectively), but there were no significant differences between activities of Cohort 1 and Cohort 2.

There were no apparent differences at any time point between protected and non-protected subjects as detected by FluoroSpot or antibody assays.

## Discussion

Immunization with RAS by mosquito bite has been critically important to malaria vaccine research. Investigation of immune mechanisms and antigens associated with protection was considerably aided by having protected and non-protected subjects who received similar immunization parameters [5, 7, 27–30]. This phase 1 clinical trial has provided (a) a better understanding of the impact of parameters affecting protective immunity and (b) a valuable repository of bio-samples and immune reagents to support future investigations of protective mechanisms. Based on the previously conducted trials [1, 12], we calculated that approximately 800–1200 infected bites would achieve 50% efficacy, and undertook immunization of the first cohort, indeed achieving our pre-trial goal (6 protected subjects/11 immunized subjects, 55% VE). However, when we repeated the same immunization schedule in a second cohort, we achieved 90% VE. To better understand this unexpected outcome, we explored the experimental and clinical details of both cohorts.

In both the 1999–2002 trial [12], and this current study, PfRAS appeared to be safe and well-tolerated in most subjects, with mild discomfort experienced during the mosquito immunization, focal local reactions, and generally mild systemic symptoms. In both studies, systemic AEs occurred at a lower frequency than local AEs. PfRAS by themselves appear to have no measurable reactogenicity as evidenced by the apparent similar rates of AEs and laboratory abnormalities comparing true- and mock-immunized volunteers (see further discussion in the S1 Appendix). Despite the good tolerability in most subjects, however, four (15%) experienced large local reactions to mosquito bites and had to be withdrawn. In general, these reactions appear to be caused by antigens in mosquito saliva since they occurred in both the true- and mock-immunized subjects. Large local reactions were also recorded in the prior NMRC trial and resulted in withdrawal, one occurring in a true-immunized subject and one in a mock-immunized subject [12]. In the 1999–2002 trial, the mock-immunized subject experienced immediate hypersensitivity with lymphangitic streaking in the affected arm; this was not seen in the current trial. In the 1999–2002 trial, [12] two subjects experienced unexpected systemic symptoms which began 16 hours after immunization, including fever, chills, muscle aches and headache. No such reactions were seen in the current trial.

As opposed to earlier published trials, in the current trial laboratory values were measured systematically on the day of immunization and 3 and 7 days post-immunization. There were no Grade 3 or Grade 4 laboratory abnormalities detected (Table 3). We were specifically interested in whether immunizations were associated with elevated liver function tests, given that irradiated sporozoites invade the liver. Notably, with regard to PfSPZ Vaccine (comprised of radiation-attenuated sporozoites) where doses are administered by direct venous inoculation (up to 2,700,000 sporozoites), there have been no significant elevations of hepatic enzymes linked to the vaccine [17, 26]. As detailed in the Results section, liver function test abnormalities in this current trial were predominantly Grade 1 in severity, with generally comparable findings in true- and mock-immunized subjects, indicating that immunization with PfRAS was not associated with hepatic laboratory findings. As RAS do not progress to a blood stage parasitemia, laboratory changes in hemoglobin, platelets, and leukocytes were not expected. There were no Grade 3 or Grade 4 hematologic abnormalities detected in either the true- or mock-immunized subjects in either cohort. The systematic laboratory testing in this trial supports the clinical findings that PfRAS by mosquito bite is generally safe and well tolerated.

Based on previous PfRAS trials [1, 12], we predicted that 800–1200 infected bites would achieve 50% VE, and we administered similar median numbers of infected bites at each immunization and similar cumulative total numbers of infected bites to both cohorts. While we attempted to follow identical protocols in both cohorts, significant differences occurred as a result of the difficulty in controlling the number of infectious mosquito bites from immunization to immunization. The first variable was that the mosquito infection rate was lower for the first cohort than the second cohort, and therefore the first cohort received a statistically significantly greater number of total bites (infected and uninfected) than the second cohort. One study in mice immunized by mosquito bite suggested that pre-exposure to uninfected bites reduced *P*. *yoelii* liver infection [31]. Thus, exposure of subjects in Cohort 1 to more uninfected mosquitoes may have reduced the entry of attenuated sporozoites into the liver leading to reduced immune responses. In addition, mosquito saliva increases levels of immunosuppressive IL-10 in the draining lymph nodes (dLN) [32], a major site of sporozoite antigenic priming, and increases the motility of regulatory T cells that might suppress activation of antigen presenting cells in the skin and dLN [33]. However, others have reported that hyperimmunization of mice to mosquito saliva did not result in any significant effect on sporozoite administration or infectivity [34]. The complexities of host-sporozoite interactions in the skin are still not fully elucidated [35].

A second variable was the statistically significantly greater number of infected bites in the first immunization in Cohort 2 compared to Cohort 1. A study in children showed that a fractional priming dose of inactivated polio vaccine induced significantly lower seroconversion rates and lower antibody titers [36]. Further analysis of the immune responses is necessary to determine whether a lower first dose affected subsequent activities of protective immune mechanisms.

Thirdly, the fifth dose was significantly reduced (three-fold) in Cohort 2 compared to Cohort 1. It has been suggested that infected mosquitoes deposit similar numbers of sporozoites per bite [37, 38] and thus, we propose that the lower numbers of bites in the fifth and final dose in Cohort 2 would have resulted in the injection of lower numbers of sporozoites than in the final dose in Cohort 1. We hypothesize that this “fractional dose” may be associated with higher efficacy.

The initial efficacy study of RTS,S vaccine used a fractional (one fifth) final (third) vaccine dose with delayed administration that was associated with greater efficacy against CHMI than 3-dose regimens without a delayed, fractional final dose [39, 40]. Recently, the fractional delayed dose regimen was re-tested resulting in similar high-level sterile protection [41]. It was suggested that the fractional dose may have increased somatic hypermutation in immunoglobulin genes resulting in improved avidity [41]. In the PfRAS study reported here, the interval between the fourth and fifth immunization was similar for both Cohorts (35 days), suggesting that the fractional dose itself may be associated with higher efficacy.

The final immunizing parameter that might have affected VE was the sporozoite gland scores during immunization that were significantly lower in Cohort 1 than Cohort 2 except during the first immunization. Studies in mice have suggested that there is no correlation between salivary gland load and numbers of sporozoites deposited during an infected bite [42–46]. In fact, 20% of heavily infected mosquitoes did not inject sporozoites, and other mosquitoes injected sporozoites on some days but not others suggesting that sporozoite injection is not related to salivary gland load [42]. Although there are no human data to definitively link salivary gland load and transmission efficiency in humans, it is possible this parameter affected protective efficacy.

Immune assays were conducted to analyze cellular IFN-γ and antibody responses, and in this study there were no differences by any measure between protected and non-protected study subjects. The details of the immunological results, including interesting associations between antigen-specific responses and protection and between cytokine dynamics and protection will be published separately.

We also examined whether immune responses were different in the two cohorts and therefore possibly related to differences in VE in each cohort. FluoroSpot IFN-γ, IL2 and IFN-γ+IL2 responses of Cohort 1 were significantly higher than Cohort 2 throughout immunization and especially after the first two immunizations, which did not correlate with higher protection in Cohort 2. Subsequent immunizations did not significantly boost responses in Cohort 1, but responses of Cohort 2 were significantly boosted after the third immunization and after the fractional fifth immunization. The rise in FluoroSpot IFN-γ, IL2 and IFN-γ+IL2 responses after the fifth dose in Cohort 2 may support the hypothesis that using a fractional dose could increase efficacy in Cohort 2. After the fractional dosing employed with RTS,S, boosting of antibody responses was seen [41]; whether or not this can be correlated with the boosting of FluoroSpot responses detected in our study is not clear. However, antibody responses to CSP (repeat and full length), but not to AMA1 nor IFA responses to sporozoites, were higher in Cohort 2 than Cohort 1 after the third and fifth immunizations, possibly suggesting a correlation between antibody responses to CSP and the higher protection in Cohort 2.

The legacy of PfRAS immunization is demonstrated in vaccines developed by Sanaria Inc before the IMRAS trial was initiated. Sanaria^®^ PfSPZ Vaccine is comprised of aseptic, purified, live (metabolically active), radiation-attenuated, cryopreserved *Plasmodium falciparum* (Pf) NF54 sporozoites administered by rapid direct venous inoculation (DVI) [47]. The initial trial utilizing intravenous administration demonstrated that five doses of 1.35x10^5^ PfSPZ of PfSPZ Vaccine induced sterile protection in 6/6 (100%) malaria-naïve subjects three weeks after the final immunization against homologous CHMI [17]. In another study [26], three doses of 4.5x10^5^ PfSPZ of PfSPZ Vaccine was shown to be similar in efficacy to five doses of 2.7x10^5^ PfSPZ of PfSPZ Vaccine, with the former protecting three 13/15 (87%) against homologous CHMI 3 weeks after the last dose and 8/14 (57%) against homologous CHMI 24 weeks after final immunization. Heterologous protection lasting up to 9 months has been demonstrated following CHMI with malaria clone 7G8 [26, 48] and against heterogeneous natural transmission for up to six months [49]. Alternative approaches use genetically attenuated sporozoites (Sanaria® PfSPZ-GA1)[23](or by the concurrent administration of an antimalarial drug after injecting non-irradiated, infectious PfSPZ (Sanaria® PfSPZ-CVac) [50, 51] In the latter case, the partner drug kills the parasites *in vivo*. Like immunization with RAS via mosquito bite, the protective immunity underlying these novel, whole SPZ-based vaccines is not well understood, since it is challenging to sample liver-resident effector CD8+ T cells. Thus, the results of IMRAS may not only promote antigen discovery and further subunit vaccine development, they may enhance our understanding of the high-level immunity induced by these promising whole SPZ vaccine candidates. IMRAS samples can be requested by application.

### Limitations of this study

The first limitation of this study is that the numbers of subjects in each cohort was limited by logistical constraints in producing PfRAS-infected mosquitoes. However, compared to previous PfRAS trials, this number allowed consistent immunization schedules and times to CHMI for each subject. The second limitation is that we only measured peripheral PBMC IFN-γ responses, whereas current evidence strongly suggests that liver-resident CD8+ T cells secreting IFN-γ are the main mediators of protection, and peripheral responses may not fully represent CD8+ T cell responses in the liver [52]. However, markers critical for migration of T cells to the liver could be studied using HLA tetramers [53, 54]. This further justifies the creation of a repository of samples from this trial for additional investigation by others.

## Supporting information

S1 Checklist CONSORT 2010 checklist of information to include when reporting a randomised trial. (DOC)

S1 Fig Association between number of infectious mosquito bites and time to CHMI with efficacy. All protected and non-protected subjects immunized with PfRAS in the 1989–1999 and 1999–2002 trials were included. For each subject, the total numbers of infectious bites were compared with time to CHMI (days). Protected and non-protected subjects from each trial are color-coded. Protected subjects generally received >800 bites and generally a time to CHMI <30 days. Based on these data, we hypothesized a range of infectious bites (800–1,000) and time to CHMI (23–25 days) to achieve 50% vaccine efficacy (black box).
(DOCX)

S2 Fig (PPTX)

S1 Appendix (DOCX)

S1 File (PDF)
