# Supplementary material for: IMRAS—A clinical trial of mosquito-bite immunization with live, radiation-attenuated P. falciparum sporozoites: Impact of immunization parameters on protective efficacy and generation of a repository of immunologic reagents
Source: PLoS One. 2020 Jun 17;15(6):e0233840. doi: 10.1371/journal.pone.0233840 (PMC7299375; doi:10.1371/journal.pone.0233840)
Supplement: S1 Fig — Association between number of infectious mosquito bites and time to CHMI with efficacy. All protected and non-protected subjects immunized with PfRAS in the 1989–1999 and 1999–2002 trials were included. For each subject, the total numbers of infectious bites were compared with time to CHMI (days). Protected and non-protected subjects from each trial are color-coded. Protected subjects generally received >800 bites and generally a time to CHMI <30 days. Based on these data, we hypothesized a range of infectious bites (800–1,000) and time to CHMI (23–25 days) to achieve 50% vaccine efficacy (black box). (DOCX) [file pone.0233840.s002.docx]

**S1Fig. Association between number of infectious mosquito bites and time to CHMI with efficacy**

All protected and non-protected subjects immunized with PfRAS in the 1989-1999 and 1999-2002 trials were included. For each subject, the total numbers of infectious bites were compared with time to CHMI (days). Protected and non-protected subjects from each trial are color-coded. Protected subjects generally received >800 bites and generally a time to CHMI <30 days. Based on these data, we hypothesized a range of infectious bites (800-1,000) and time to CHMI (23-25 days) to achieve 50% vaccine efficacy (black box).
