# Supplementary material for: IMRAS—A clinical trial of mosquito-bite immunization with live, radiation-attenuated P. falciparum sporozoites: Impact of immunization parameters on protective efficacy and generation of a repository of immunologic reagents
Source: PLoS One. 2020 Jun 17;15(6):e0233840. doi: 10.1371/journal.pone.0233840 (PMC7299375; doi:10.1371/journal.pone.0233840)
Supplement: S2 Fig — (PPTX) [file pone.0233840.s003.pptx]

## Slide 1
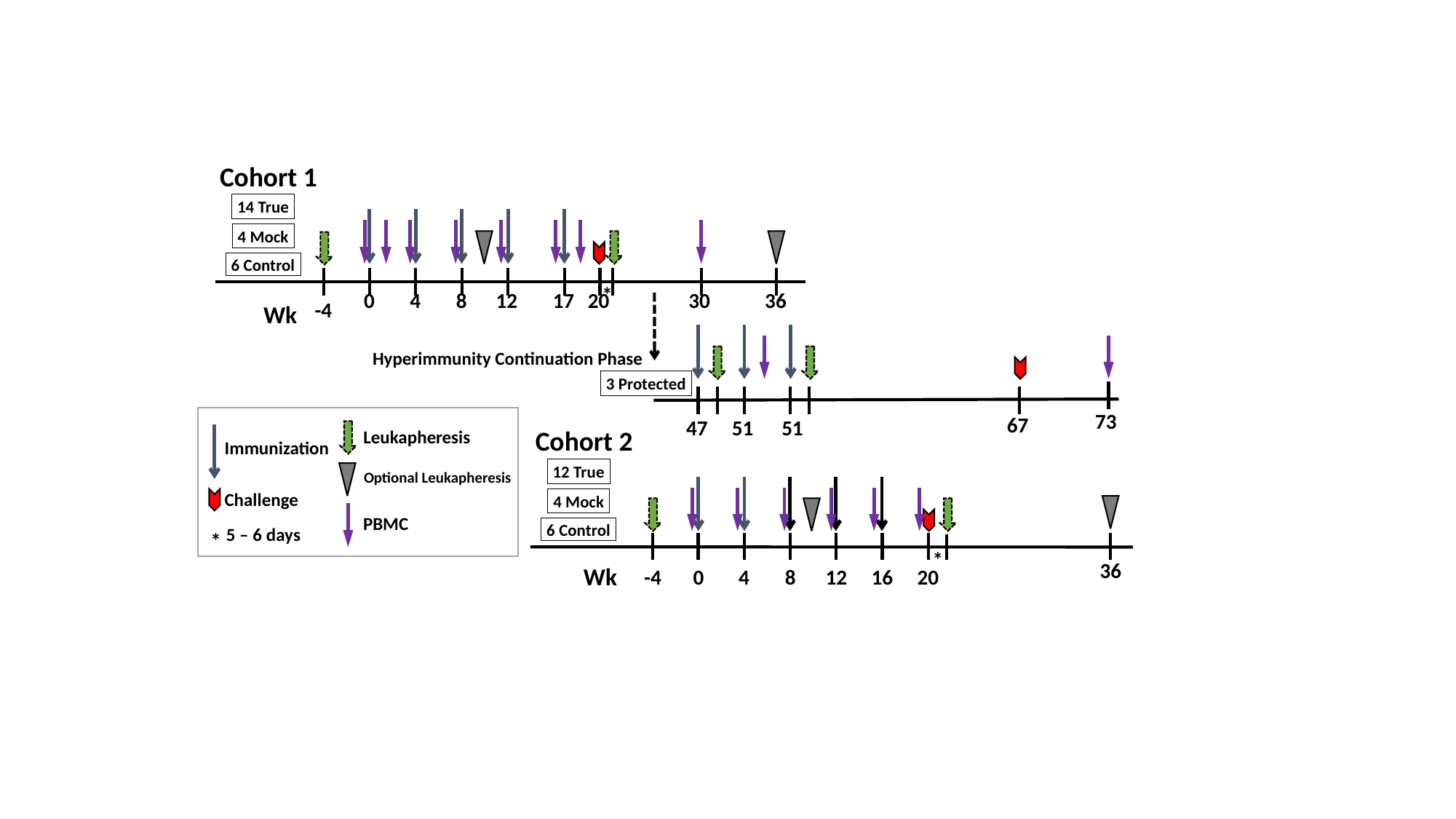

Cohort 1
14 True
4 Mock
6 Control
*
0
4
8
12
17
20
30
36
-4
Wk
Hyperimmunity Continuation Phase
3 Protected
73
67
51
47
51
Cohort 2
Leukapheresis
Immunization
12 True
Optional Leukapheresis
Challenge
4 Mock
PBMC
5 – 6 days
6 Control
*
*
36
Wk
-4
0
4
8
12
16
20
