# Supplementary material for: IMRAS—A clinical trial of mosquito-bite immunization with live, radiation-attenuated P. falciparum sporozoites: Impact of immunization parameters on protective efficacy and generation of a repository of immunologic reagents
Source: PLoS One. 2020 Jun 17;15(6):e0233840. doi: 10.1371/journal.pone.0233840 (PMC7299375; doi:10.1371/journal.pone.0233840)
Supplement: S1 Appendix — (DOCX) [file pone.0233840.s004.docx]

**Supplementary Appendix**

**IMRAS: A clinical trial of mosquito-bite immunization with live, radiation-attenuated *P. falciparum* sporozoites designed to assess the relationship between immunization parameters and protection and to generate a repository of reagents for identification of protective immune responses**

**Supplementary Results**

**Safety and Tolerability**

As described in the main text under Results: Study Flow, 4 subjects (2 true immunized and 2 mock immunized) developed large, local reactions with significant proximal extension that became generalized but confined to the skin. The clinical investigators obtained consultations from a specialist in allergy and immunology who advised that the reactions were likely due to an IgE-mediated allergic reaction most likely to mosquito antigens and not related to parasite antigens. All subjects were withdrawn from participation and did not receive further immunizations.

The specific details for each subject are as follows:

- **Subject IMRAS #63**: The subject, a 22-year-old female, was consented to the study on 30 April 2014 and randomized to the true-immunized group. On 10 June 2014, she received her first immunization without adverse reaction. On 8 July 2014, she received her second scheduled immunization consisting of 284 total irradiated mosquito bites, of which 227 were infected with *Plasmodium falciparum* parasites. Immediately following the first 5 minutes of mosquito feeding, it was noted that the subject had a vigorous local reaction with several wheals and mild swelling within the borders of the mosquito container. Approximately 10 minutes after the conclusion of immunization, the subject developed generalized pruritus and hives on her neck, head, and chin. The subject was noted to have normal vital signs, no respiratory distress or increased work of breathing, lungs clear to auscultation, and normal pharynx. Her local reaction consisted of large confluence of swelling inside the area of the bite sites with expansion of 1 cm swelling beyond the diameter of the container. The subject was treated with intramuscular diphenhydramine and after 2 hours her hives and generalized pruritus were resolved. She had mild residual pruritus on her head and her ears. She was discharged with a prescription for oral diphenhydramine and continued to take the medication over the next 24 hours. This allergic reaction was Grade 3 in severity, and the adverse event was considered definitely related to immunization.
- **Subject #17**: The subject, a 27-year-old male, was consented to the study on 18 February 2014 and randomized to the mock-immunization group. The first 2 immunizations occurred on 10 June 2014 and 8 July 2014 and were without adverse reaction. On 5 August 2014, the subject received his third scheduled immunization consisting of 226 uninfected, irradiated mosquito bites. Immediately following the first 5 minutes of mosquito feeding it was noted that the subject had a vigorous local response with coalescence of several wheals resulting in a solid area of mild swelling within the borders of the mosquito container. Approximately 10 minutes after the conclusion of the immunization, the subject developed diffuse pruritus and hives with papules and wheals on his head, neck, chest, and back. The subject was noted to have normal vital signs, no respiratory distress or increased work of breathing, lungs clear to auscultation, regular heart rate and rhythm, soft and non-tender abdomen, and normal pharynx. His local reaction consisted of a large confluence of swelling inside the area of the mosquito container. The subject was treated with intramuscular diphenhydramine and after 3 hours his hives and generalized pruritus were resolved. He had mild residual erythema on his head and neck. He was discharged with a prescription for oral diphenhydramine and continued to take the medication over the next 24 hours. This allergic reaction was Grade 3 in severity, and the adverse event was considered definitely related to immunization.
- **Subject #98:** The subject, a 37-year-old male, was consented to the study on 24 March 2015 and randomized to the true-immunized group. The first 2 immunizations occurred on 5 May 2015 and 2 Jun 2015 and were without adverse reaction. On 30 June 2015, the subject received his third scheduled immunization consisting of 251 total irradiated mosquito bites, of which 217 were infected with *Plasmodium falciparum* parasites. After the third immunization, he was noted to have a vigorous local response with moderate swelling and erythema (11 cm x 9.5 cm area of induration and 13 cm x 9.5 cm erythematous patch) at the immunization site. The subject also developed nonpruritic erythematous patches (2 cm x 2 cm) on the anterior neck, chest, and face. He had no generalized pruritus, no skin or soft tissue swelling of the face or neck, and no respiratory distress. His vital signs remained normal. He was treated with an antihistamine (diphenhydramine) after which the rash on his face, neck, and chest rapidly resolved. The large local reaction at the immunization site resolved within 24-48 hours of the procedure. The subject received no further immunization and remained in the study for safety participation only. The subject was provided counseling on the use of mosquito avoidance measures and was advised to continue antihistamine as needed. At follow-up, the subject had no further episode of rash.
- **Subject #100:** The subject, a 23-year-old male, was consented to the study on 24 February 2015 and randomized to the mock-immunization group. The first 2 immunizations occurred on 5 May 2015 and 2 June 2015 and were without adverse reaction. On 30 June 2015, the subject received his third mock-immunization consisting of 227 uninfected, irradiated mosquito bites. After immunization, the subject had a vigorous local response on the right arm with wheals and moderate swelling (10 cm x 8 cm) and erythema (11.5 cm x 15 cm). The subject also began to develop hives on the bilateral forearms and trunk. He had no generalized pruritus and his vital signs remained normal. The subject rapidly responded to an antihistamine (diphenhydramine). The large local reaction at the immunization site resolved within 24 hours of the immunization. The subject received no further immunizations and remained in the study for safety participation only. The subject was provided counseling on the use of mosquito avoidance measures and was advised to continue antihistamine as needed. At follow-up, the subject had no further episodes of rash or hives.

As described in the main text under Results: Study Flow, Cohort 2, Mock-immunization group, one subject suffered an unrelated serious AE. The details of the SAE are as follows:

- **Subject #126**, a 23‑year-old female, was consented to the study on 31 March 2015 and randomized to the mock-immunized group. On 5 May 2015 she received her first immunization without adverse reaction. The subject failed to show for her second scheduled immunization on 2 June 2015. The subject was reportedly ill and sought medical care in a local Emergency Department (ED) on the evening of 1 June 2015. In the ED, she was diagnosed with dizziness, hyperglycemia, recurrent pharyngitis, asthma exacerbation, and bilateral upper extremity cramping/numbness secondary to hyperventilation. She was discharged with instructions to follow up with her primary care physician. Her hyperglycemia was assessed to be induced by steroids, which were prescribed for the treatment of the subject’s asthma exacerbation the day prior to her ED presentation. Her steroid-induced hyperglycemia resolved after the steroid was stopped; her hyperglycemia resolved without medications. Her recurrent pharyngitis resolved after adenotonsillectomy on 8 June 2015. The asthma exacerbation suggested that her asthma may be chronic and not well-controlled. Additionally, she had a diagnosis of polycystic ovarian syndrome, which was not disclosed during her screening and, therefore, she no longer met the inclusion/exclusion criteria to remain in the study. The subject was withdrawn from the study on 5 August 2015, secondary to an Adverse Event, Unexpected, Grade 4 (Serious) of hyperglycemia and acute asthma exacerbation that occurred approximately 27 days after receiving mock-immunization 1, not related to the investigational product.

Unsolicited Adverse Events

Among true-immunized subjects, there were fewer unsolicited AEs (10) in Cohort 1 than in Cohort 2 (42), whereas unsolicited AEs after mock-immunizations were similar. The increased AEs in Cohort 2 were largely Grade 1, and were predominantly distributed among systemic AEs. All resolved rapidly without sequelae and were deemed not related to the investigational product. One mock-immunized subjected had an unrelated SAE 27 days after the first immunization (asthma exacerbation and hyperglycemia) (Subject #126, see Supplementary Appendix for details).

The relationship between solicited and unsolicited AEs and true- or mock-immunization are shown in Table S3. Of the 71 unsolicited AEs recorded, 10 events (14%) were deemed possibly, probably or definitely related to the investigational product

**Supplementary Tables**

**Table S1: Solicited adverse events by severity for subjects in both study cohorts safety population at each immunization**

|  | **Immunization** | | | | |
| --- | --- | --- | --- | --- | --- |
|  | **1** | **2** | **3** | **4** | **5** |
| **True-immunized** |  | | | | |
| Subjects receiving immunization | 25 | 23 | 22 | 21 | 21 |
| Total number of events | 16 | 13 | 12 | 3 | 5 |
| Grade 1 | 13 (81%) | 9 (69%) | 7 (58%) | 3 (100%) | 4 (80%) |
| Grade 2 | 3 (19%) | 0 | 3 (28%) | 0 | 1 (20%) |
| Grade 3 | 0 | 4 (31%) | 2 (17%) | 0 | 0 |
| Number of subjects with at least one event | 9 (36%) | 9 (39.1%) | 7 (32%) | 3 (14%) | 4 (19%) |
|  |  |  |  |  |  |
| **Mock-immunized** |  |  |  |  |  |
| Subjects receiving immunization | 8 | 6 | 7 | 4 | 5 |
| Total number of events | 2 | 4 | 7 | 0 | 6 |
| Grade 1 | 2 (100%) | 3 (75%) | 2 (29%) | 0 | 6 (100%) |
| Grade 2 | 0 | 0 | 3 (43%) | 0 | 0 |
| Grade 3 | 0 | 1 (25%) | 2 (29%) | 0 | 0 |
| Number of subjects with at least one event | 2 (25%) | 2 (33%) | 3 (43%) | 0 | 3 (60%) |

Summary is limited to subjects in either study cohort who received at least one immunization. Solicited adverse events for the immunizations which occurred as part of the hyper-immunization component of the trial are not included. Percentages are based on the number of summarized subjects who were included in the indicated intervention group.

**Table S2: Unsolicited adverse events occurring within 14 days of any immunization**

|  | Cohort 1 | | | | | | Cohort 2 | | | | | | |
| --- | --- | --- | --- | --- | --- | --- | --- | --- | --- | --- | --- | --- | --- |
|  | True n =13 | | | Mock n=4 | | | True n =12 | | | Mock n= 4 | | | |
|  | **Grade 1** | **Grade 2** | **Grade 3** | **Grade 1** | **Grade 2** | **Grade 3** | **Grade 1** | **Grade 2** | **Grade 3** | **Grade 1** | **Grade 2** | **Grade 3** | **Grade 4** |
| Acne | 0 | 0 | 0 | 0 | 0 | 0 | 0 | 1 (8 %) | 0 | 0 | 0 | 0 | 0 |
| Alanine aminotransferase | 0 | 0 | 0 | 0 | 0 | 0 | 0 | 1 (8 %) | 0 | 0 | 0 | 0 | 0 |
| Arthralgia | 0 | 0 | 0 | 0 | 0 | 0 | 1 (8 %) | 0 | 0 | 0 | 0 | 0 | 0 |
| Arthropod bite | 1 (8 %) | 0 | 0 | 0 | 0 | 0 | 0 | 0 | 0 | 0 | 0 | 0 | 0 |
| Asthma | 0 | 0 | 0 | 0 | 0 | 0 | 0 | 0 | 0 | 0 | 0 | 0 | 1 (25 %) |
| Autoimmune thyroiditis | 0 | 0 | 0 | 0 | 0 | 0 | 0 | 1 (8 %) | 0 | 0 | 0 | 0 | 0 |
| Axillary pain | 1 (8 %) | 0 | 0 | 0 | 0 | 0 | 0 | 0 | 0 | 0 | 0 | 0 | 0 |
| Back pain | 0 | 0 | 0 | 0 | 0 | 0 | 1 (8 %) | 0 | 0 | 0 | 0 | 0 | 0 |
| Benign bone neoplasm | 0 | 0 | 0 | 0 | 0 | 0 | 0 | 0 | 0 | 1 (25 %) | 0 | 0 | 0 |
| BP diastolic increased | 0 | 0 | 0 | 0 | 0 | 0 | 1 (8 %) | 0 | 0 | 0 | 0 | 0 |  |
| Chills | 0 | 0 | 0 | 1 (25 %) | 0 | 0 | 0 | 0 | 0 | 0 | 0 | 0 | 0 |
| Cough | 1 (8 %) | 0 | 0 | 0 | 0 | 0 | 2 (17 %) | 0 | 0 | 0 | 0 | 0 | 0 |
| Dehydration | 0 | 1 (8 %) | 0 | 0 | 0 | 0 | 0 | 0 | 0 | 0 | 0 | 0 | 0 |
| Depressive symptom | 0 | 0 | 0 | 0 | 0 | 0 | 1 (8 %) | 0 | 0 | 0 | 0 | 0 | 0 |
| Dermatitis contact | 0 | 0 | 0 | 0 | 0 | 0 | 1 (8 %) | 0 | 0 | 0 | 0 | 0 | 0 |
| Disturbance in attention | 0 | 0 | 0 | 0 | 0 | 0 | 1 (8 %) | 0 | 0 | 0 | 0 | 0 | 0 |
| Dizziness | 0 | 0 | 0 | 0 | 0 | 0 | 1 (8 %) | 0 | 0 | 0 | 0 | 0 | 0 |
| Dysgeusia | 1 (8 %) | 0 | 0 | 0 | 0 | 0 | 0 | 0 | 0 | 0 | 0 | 0 | 0 |
| Erythema | 0 | 0 | 0 | 0 | 0 | 0 | 0 | 0 | 0 | 1 (25 %) | 0 | 0 | 0 |
| Fatigue | 0 | 0 | 0 | 0 | 0 | 0 | 1 (8 %) | 0 | 0 | 0 | 0 | 0 | 0 |
| Headache | 0 | 0 | 0 | 1 (25 %) | 0 | 0 | 0 | 0 | 0 | 0 | 0 | 0 | 0 |
| Hepatic steatosis | 0 | 0 | 0 | 0 | 0 | 0 | 0 | 1 (8 %) | 0 | 0 | 0 | 0 | 0 |
| Hordeolum | 0 | 1 (8 %) | 0 | 0 | 0 | 0 | 0 | 0 | 0 | 0 | 0 | 0 | 0 |
| Hyperglycaemia | 0 | 0 | 0 | 0 | 0 | 0 | 0 | 0 | 0 | 0 | 0 | 0 | 1 (25 %) |
| Hyperlipidaemia | 0 | 0 | 0 | 0 | 0 | 0 | 0 | 1 (8 %) | 0 | 0 | 0 | 0 | 0 |
| Hypertension | 1 (8 %) | 0 | 0 | 0 | 1 (25 %) | 0 | 0 | 0 | 0 | 0 | 0 | 0 | 0 |
| Lymph node pain | 0 | 0 | 0 | 0 | 0 | 0 | 1 (8 %) | 0 | 0 | 0 | 0 | 0 | 0 |
| Lymphadenopathy | 0 | 0 | 0 | 0 | 0 | 0 | 1 (8 %) | 1 (8 %) | 0 | 0 | 0 | 0 | 0 |
| Muscle spasms | 0 | 1 (8 %) | 0 | 0 | 0 | 0 | 0 | 0 | 0 | 0 | 1 (25 %) | 0 | 0 |
| Muscle strain | 0 | 0 | 0 | 0 | 0 | 0 | 0 | 0 | 0 | 1 (25 %) | 0 | 0 | 0 |
| Musculoskeletal pain | 0 | 0 | 0 | 0 | 0 | 0 | 1 (8 %) | 0 | 0 | 0 | 0 | 0 | 0 |
| Myalgia | 0 | 0 | 0 | 0 | 0 | 0 | 1 (8 %) | 0 | 0 | 0 | 0 | 0 | 0 |
| Nasal congestion | 0 | 0 | 0 | 0 | 0 | 0 | 4 (33 %) | 0 | 0 | 0 | 0 | 0 | 0 |
| Nasopharyngitis | 0 | 0 | 0 | 0 | 0 | 0 | 2 (17 %) | 0 | 0 | 1 (25 %) | 0 | 0 | 0 |
| Nausea | 0 | 0 | 0 | 0 | 0 | 0 | 0 | 0 | 0 | 1 (25 %) | 0 | 0 | 0 |
| Oropharyngeal pain | 0 | 0 | 0 | 0 | 0 | 0 | 5 (42 %) | 0 | 0 | 0 | 0 | 0 | 0 |
| Pain | 0 | 0 | 0 | 1 (25 %) | 0 | 0 | 0 | 0 | 0 | 0 | 0 | 0 | 0 |
| Pain in extremity | 1 (8 %) | 0 | 0 | 1 (25 %) | 0 | 0 | 2 (17 %) | 0 | 0 | 0 | 0 | 0 | 0 |
| Paranasal sinus discomfort | 0 | 0 | 0 | 0 | 1 (25 %) | 0 | 0 | 0 | 0 | 0 | 0 | 0 | 0 |
| Pharyngitis | 0 | 0 | 0 | 0 | 0 | 0 | 0 | 0 | 0 | 0 | 1 (25 %) | 0 | 0 |
| Post-traumatic pain | 1 (8 %) | 0 | 0 | 0 | 0 | 0 | 0 | 0 | 0 | 0 | 0 | 0 | 0 |
| Pyrexia | 0 | 0 | 0 | 1 (25 %) | 0 | 0 | 0 | 0 | 0 | 0 | 0 | 0 | 0 |
| Rash erythematous | 0 | 0 | 0 | 0 | 0 | 0 | 0 | 0 | 1 (8 %) | 0 | 0 | 0 | 0 |
| Rhinorrhoea | 0 | 0 | 0 | 0 | 0 | 0 | 2 (17 %) | 0 | 0 | 0 | 0 | 0 | 0 |
| Stress | 0 | 0 | 0 | 0 | 0 | 0 | 1 (8 %) | 0 | 0 | 0 | 0 | 0 | 0 |
| Tenosynovitis | 0 | 0 | 0 | 0 | 0 | 0 | 1 (8 %) | 0 | 0 | 0 | 0 | 0 | 0 |
| Tympanic membrane perforation | 0 | 0 | 0 | 0 | 0 | 0 | 0 | 1 (8 %) | 0 | 0 | 0 | 0 | 0 |
| URI infection | 0 | 0 | 0 | 0 | 0 | 0 | 1 (8 %) | 0 | 0 | 0 | 0 | 0 | 0 |
| Vaccination site papule | 0 | 0 | 0 | 0 | 0 | 0 | 1 (8 %) | 0 | 0 | 0 | 0 | 0 | 0 |
| Vaccination site swelling | 0 | 0 | 0 | 1 (25 %) | 0 | 0 | 0 | 0 | 0 | 0 | 0 | 0 | 0 |
| Vomiting | 0 | 0 | 0 | 0 | 0 | 0 | 0 | 0 | 0 | 1 (25 %) | 0 | 0 | 0 |

Summary is limited to subjects who received at least one immunization. A subject who reported one or more such events was counted once for the most severe event. Unsolicited adverse events for the immunizations which occurred as part of the hyper-immunization component of the trial are not included.

**Table S3: Post-Immunization solicited adverse events by relationship to investigational product for subjects in both study cohorts safety population**

|  | **True-immunized**  n=25 | **Mock-immunized**  n=8 | **Total**  n=33 |
| --- | --- | --- | --- |
| **Solicited AEs** |  |  |  |
| Number of events | 49 | 19 | 68 |
| Not related | 2 (4%) | 1 (5%) | 3 (4%) |
| Unlikely | 4 (8%) | 0 | 4 (6%) |
| Possible | 4 (8%) | 0 | 4 (6%) |
| Probable | 3 (6%) | 0 | 3 (4%) |
| Definite | 36 (74%) | 18 (95%) | 54 (79%) |
| Number of subjects with at least one event |  |  |  |
| Not related | 0 | 0 | 0 |
| Unlikely | 2 (8%) | 0 | 2 (6%) |
| Possible | 1 (4%) | 0 | 1 (3%) |
| Probable | 0 | 0 | 0 |
| Definite | 13 (52%) | 7 (88%) | 20 (61%) |
|  |  |  |  |
| **Unsolicited AEs** |  |  |  |
| Number of events | 52 | 19 | 71 |
| Not related | 36 (69%) | 11 (58%) | 47 (66%) |
| Unlikely | 8 (15%) | 6 (32%) | 14 (20%) |
| Possible | 2 (4%) | 0 | 2 (3%) |
| Probable | 2 (4%) | 0 | 2 (3%) |
| Definite | 4 (8%) | 2 (11%) | 6 (9%) |
| Number of subjects with at least one event |  |  |  |
| Not related | 7 (28%) | 3 (38 %) | 10 (30%) |
| Unlikely | 4 (16%) | 2 (25%) | 6 (18%) |
| Possible | 2 (8%) | 0 | 2 (6%) |
| Probable | 1 (4%) | 0 | 1 (3%) |
| Definite | 4 (16%) | 2 (25%) | 6 (18%) |

Summary is limited to subjects in either study cohort who received at least one immunization. Solicited adverse events for the immunizations which occurred as part of the hyper-immunization component of the trial are not included. Percentages are based on the number of summarized subjects who were included in the indicated intervention group.

**Table S4: Cohort 1: Total bites and total infectious bites at each immunization**

| **TOTAL BITES** | **All subjects (n=11)** | **Imm1** | **Imm2** | **Imm3** | **Imm4** | **Imm5** | **Total** |
| --- | --- | --- | --- | --- | --- | --- | --- |
|  | **v2** | 215 | 321 | 273 | 230 | 293 | 1332 |
|  | **v5** | 265 | 243 | 367 | 286 | 243 | 1404 |
|  | **v21** | 208 | 169 | 315 | 345 | 286 | 1323 |
|  | **v33** | 200 | 268 | 250 | 331 | 266 | 1315 |
|  | **v39** | 187 | 266 | 323 | 234 | 351 | 1361 |
|  | **v45** | 178 | 114 | 295 | 326 | 290 | 1203 |
|  | **v57** | 203 | 298 | 374 | 371 | 328 | 1574 |
|  | **v60** | 240 | 236 | 251 | 261 | 223 | 1211 |
|  | **v61** | 272 | 248 | 306 | 396 | 288 | 1510 |
|  | **v71** | 207 | 215 | 259 | 177 | 253 | 1111 |
|  | **v78** | 205 | 302 | 190 | 287 | 279 | 1263 |
|  |  |  |  |  |  |  |  |
|  | **Protected subjects (n=6)** | **Imm1** | **Imm2** | **Imm3** | **Imm4** | **Imm5** | **Total** |
|  | **v2** | 215 | 321 | 273 | 230 | 293 | 1332 |
|  | **v39** | 187 | 266 | 323 | 234 | 351 | 1361 |
|  | **v45** | 178 | 114 | 295 | 326 | 290 | 1203 |
|  | **v57** | 203 | 298 | 374 | 371 | 328 | 1574 |
|  | **v60** | 240 | 236 | 251 | 261 | 223 | 1211 |
|  | **v78** | 205 | 302 | 190 | 287 | 279 | 1263 |
|  |  |  |  |  |  |  |  |
|  | **Non-protected**  **Subjects (n=5)** | **Imm1** | **Imm2** | **Imm3** | **Imm4** | **Imm5** | **Total** |
|  | **v5** | 265 | 243 | 367 | 286 | 243 | 1404 |
|  | **v21** | 208 | 169 | 315 | 345 | 286 | 1323 |
|  | **v33** | 200 | 268 | 250 | 331 | 266 | 1315 |
|  | **v61** | 272 | 248 | 306 | 396 | 288 | 1510 |
|  | **v71** | 207 | 215 | 259 | 177 | 253 | 1111 |
|  |  |  |  |  |  |  |  |
| **INFECTIOUS BITES** | **All subjects (n=11)** | **Imm1** | **Imm2** | **Imm3** | **Imm4** | **Imm5** | **Total** |
|  | **v2** | 187 | 278 | 118 | 169 | 272 | 1024 |
|  | **v5** | 228 | 178 | 294 | 181 | 227 | 1108 |
|  | **v21** | 158 | 107 | 231 | 218 | 248 | 962 |
|  | **v33** | 166 | 188 | 158 | 210 | 239 | 961 |
|  | **v39** | 180 | 222 | 269 | 187 | 269 | 1127 |
|  | **v45** | 148 | 83 | 215 | 290 | 271 | 1007 |
|  | **v57** | 168 | 247 | 249 | 309 | 262 | 1235 |
|  | **v60** | 175 | 156 | 201 | 209 | 186 | 927 |
|  | **v61** | 264 | 188 | 245 | 303 | 221 | 1221 |
|  | **v71** | 180 | 194 | 225 | 0 | 211 | 810 |
|  | **v78** | 150 | 171 | 152 | 239 | 205 | 917 |
|  |  |  |  |  |  |  |  |
|  | **Protected subjects (n=6)** | **Imm1** | **Imm2** | **Imm3** | **Imm4** | **Imm5** | **Total** |
|  | **v2** | 187 | 278 | 118 | 169 | 272 | 1024 |
|  | **v39** | 180 | 222 | 269 | 187 | 269 | 1127 |
|  | **v45** | 148 | 83 | 215 | 290 | 271 | 1007 |
|  | **v57** | 168 | 247 | 249 | 309 | 262 | 1235 |
|  | **v60** | 175 | 156 | 201 | 209 | 186 | 927 |
|  | **v78** | 150 | 171 | 152 | 239 | 205 | 917 |
|  |  |  |  |  |  |  |  |
|  | **Non-protected**  **Subjects (n=5)** | **Imm1** | **Imm2** | **Imm3** | **Imm4** | **Imm5** | **Total** |
|  | **v5** | 228 | 178 | 294 | 181 | 227 | 1108 |
|  | **v21** | 158 | 107 | 231 | 218 | 248 | 962 |
|  | **v33** | 166 | 188 | 158 | 210 | 239 | 961 |
|  | **v61** | 264 | 188 | 245 | 303 | 221 | 1221 |
|  | **v71** | 180 | 194 | 225 | 0 | 211 | 810 |

Subjects are shown as All subjects (n=11), Protected subjects (n=6) and Non-protected subjects (n=5). Total numbers of bite and total number of infectious bites delivered at each immunization (Imm). Numbers of infectious bites were total number of bites x infectivity rate of all mosquitoes used in that immunization.

**Table S5: Cohort 2: Total bites and total infectious bites at each immunization**

| **TOTAL BITES** | **All subjects (n=10)** | **Imm1** | **Imm2** | **Imm3** | **Imm4** | **Imm5** | **Total** |
| --- | --- | --- | --- | --- | --- | --- | --- |
|  | **v102** | 290 | 219 | 305 | 294 | 78 | 1186 |
|  | **v103** | 332 | 222 | 317 | 218 | 99 | 1188 |
|  | **v110** | 303 | 178 | 326 | 252 | 99 | 1158 |
|  | **v114** | 287 | 190 | 237 | 196 | 76 | 986 |
|  | **v120** | 270 | 321 | 317 | 256 | 79 | 1243 |
|  | **v127** | 283 | 302 | 113 | 292 | 79 | 1069 |
|  | **v130** | 336 | 324 | 327 | 256 | 90 | 1333 |
|  | **v131** | 253 | 228 | 277 | 184 | 85 | 1027 |
|  | **v133** | 290 | 347 | 266 | 234 | 61 | 1198 |
|  | **v134** | 253 | 206 | 236 | 235 | 46 | 976 |
|  |  |  |  |  |  |  |  |
|  | **Protected subjects (n=9)** | **Imm1** | **Imm2** | **Imm3** | **Imm4** | **Imm5** | **Total** |
|  | **v102** | 290 | 219 | 305 | 294 | 78 | 1186 |
|  | **v103** | 332 | 222 | 317 | 218 | 99 | 1188 |
|  | **v114** | 287 | 190 | 237 | 196 | 76 | 986 |
|  | **v120** | 270 | 321 | 317 | 256 | 79 | 1243 |
|  | **v127** | 283 | 302 | 113 | 292 | 79 | 1069 |
|  | **v130** | 336 | 324 | 327 | 256 | 90 | 1333 |
|  | **v131** | 253 | 228 | 277 | 184 | 85 | 1027 |
|  | **v133** | 290 | 347 | 266 | 234 | 61 | 1198 |
|  | **v134** | 253 | 206 | 236 | 235 | 46 | 976 |
|  |  |  |  |  |  |  |  |
|  | **Non-protected**  **subjects (n=1)** | **Imm1** | **Imm2** | **Imm3** | **Imm4** | **Imm5** | **Total** |
|  | **110** | 303 | 178 | 326 | 252 | 99 | 1158 |
|  |  |  |  |  |  |  |  |
| **INFECTIOUS BITES** | **All subjects (n=10)** | **Imm1** | **Imm2** | **Imm3** | **Imm4** | **Imm5** | **Total** |
|  | **v102** | 222 | 190 | 275 | 265 | 70 | 1022 |
|  | **v103** | 232 | 192 | 306 | 203 | 96 | 1029 |
|  | **v110** | 242 | 166 | 326 | 218 | 86 | 1038 |
|  | **v114** | 249 | 158 | 205 | 163 | 76 | 851 |
|  | **v120** | 207 | 310 | 296 | 239 | 68 | 1120 |
|  | **v127** | 217 | 262 | 94 | 263 | 79 | 915 |
|  | **v130** | 246 | 302 | 294 | 205 | 84 | 1131 |
|  | **v131** | 219 | 144 | 231 | 176 | 77 | 847 |
|  | **v133** | 242 | 312 | 266 | 195 | 51 | 1066 |
|  | **v134** | 194 | 172 | 220 | 212 | 41 | 839 |
|  |  |  |  |  |  |  |  |
|  | **Protected subjects (n=9)** | **Imm1** | **Imm2** | **Imm3** | **Imm4** | **Imm5** | **Total** |
|  | **v102** | 222 | 190 | 275 | 265 | 70 | 1022 |
|  | **v103** | 232 | 192 | 306 | 203 | 96 | 1029 |
|  | **v114** | 249 | 158 | 205 | 163 | 76 | 851 |
|  | **v120** | 207 | 310 | 296 | 239 | 68 | 1120 |
|  | **v127** | 217 | 262 | 94 | 263 | 79 | 915 |
|  | **v130** | 246 | 302 | 294 | 205 | 84 | 1131 |
|  | **v131** | 219 | 144 | 231 | 176 | 77 | 847 |
|  | **v133** | 242 | 312 | 266 | 195 | 51 | 1066 |
|  | **v134** | 194 | 172 | 220 | 212 | 41 | 839 |
|  |  |  |  |  |  |  |  |
|  | **Non-protected subjects (n-1)** | **Imm1** | **Imm2** | **Imm3** | **Imm4** | **Imm5** | **Total** |
|  | **v110** | 242 | 166 | 326 | 218 | 86 | 1038 |

Subjects are shown as All subjects (n=10), Protected subjects (n=9) and Non-protected subjects (n=1). Total numbers of bite and total number of infectious bites delivered at each immunization (Imm). Numbers of infectious bites were total number of bites x infectivity rate of all mosquitoes used in that immunization.

**Table S6: Cohort 1: Days of immunization and CHMI**

| **All subjects (n=11)** | **Imm1** | **Imm2** | **Imm3** | **Imm4** | **Imm5** | **CHMI** | **CHMI interval** |
| --- | --- | --- | --- | --- | --- | --- | --- |
| **v2** | 0 | 28 | 56 | 85 | 119 | 141 | 22 |
| **v5** | 0 | 28 | 56 | 85 | 119 | 141 | 22 |
| **v21** | 0 | 28 | 56 | 85 | 119 | 141 | 22 |
| **v33** | 0 | 28 | 56 | 85 | 119 | 141 | 22 |
| **v39** | 0 | 28 | 56 | 85 | 119 | 143 | 24 |
| **v45** | 0 | 28 | 56 | 85 | 119 | 143 | 24 |
| **v57** | 0 | 28 | 56 | 85 | 119 | 143 | 24 |
| **v60** | 0 | 28 | 56 | 85 | 119 | 143 | 24 |
| **v61** | 0 | 28 | 56 | 85 | 119 | 141 | 22 |
| **v71** | 0 | 28 | 56 | 85 | 119 | 143 | 24 |
| **v78** | 0 | 28 | 56 | 85 | 119 | 141 | 22 |
|  |  |  |  |  |  |  |  |
| **Protected subjects (n=6)** | **Imm1** | **Imm2** | **Imm3** | **Imm4** | **Imm5** | **CHMI** | **CHMI interval** |
| **v2** | 0 | 28 | 56 | 85 | 119 | 141 | 22 |
| **v39** | 0 | 28 | 56 | 85 | 119 | 143 | 24 |
| **v45** | 0 | 28 | 56 | 85 | 119 | 143 | 24 |
| **v57** | 0 | 28 | 56 | 85 | 119 | 143 | 24 |
| **v60** | 0 | 28 | 56 | 85 | 119 | 143 | 24 |
| **v78** | 0 | 28 | 56 | 85 | 119 | 141 | 22 |
|  |  |  |  |  |  |  |  |
| **Non-protected**  **Subjects (n=5)** | **Imm1** | **Imm2** | **Imm3** | **Imm4** | **Imm5** | **CHMI** | **CHMI interval** |
| **v5** | 0 | 28 | 56 | 85 | 119 | 141 | 22 |
| **v21** | 0 | 28 | 56 | 85 | 119 | 141 | 22 |
| **v33** | 0 | 28 | 56 | 85 | 119 | 141 | 22 |
| **v61** | 0 | 28 | 56 | 85 | 119 | 141 | 22 |
| **v71** | 0 | 28 | 56 | 85 | 119 | 143 | 24 |

Subjects were immunized five times at the days shown, and CHMI was received 22-24 days later.

**Table S7: Cohort 2: Days of immunization and CHMI**

| **All subjects (n=10)** | **Imm1** | **Imm2** | **Imm3** | **Imm4** | **Imm5** | **CHMI** | **CHMI interval** |
| --- | --- | --- | --- | --- | --- | --- | --- |
| **v102** | 0 | 28 | 56 | 84 | 119 | 143 | 24 |
| **v103** | 0 | 28 | 56 | 84 | 119 | 141 | 22 |
| **v110** | 0 | 28 | 56 | 84 | 119 | 143 | 24 |
| **v114** | 0 | 28 | 56 | 84 | 119 | 141 | 22 |
| **v120** | 0 | 28 | 56 | 84 | 119 | 141 | 22 |
| **v127** | 0 | 28 | 56 | 84 | 119 | 143 | 24 |
| **v130** | 0 | 38 | 56 | 84 | 119 | 141 | 22 |
| **v131** | 0 | 28 | 56 | 84 | 119 | 143 | 24 |
| **v133** | 0 | 38 | 56 | 84 | 119 | 141 | 22 |
| **v134** | 0 | 28 | 56 | 84 | 119 | 143 | 24 |
|  |  |  |  |  |  |  |  |
| **Protected subjects (n=9)** | **Imm1** | **Imm2** | **Imm3** | **Imm4** | **Imm5** | **CHMI** | **CHMI interval** |
| **v102** | 0 | 28 | 56 | 84 | 119 | 143 | 24 |
| **v103** | 0 | 28 | 56 | 84 | 119 | 141 | 22 |
| **v114** | 0 | 28 | 56 | 84 | 119 | 141 | 22 |
| **v120** | 0 | 28 | 56 | 84 | 119 | 141 | 22 |
| **v127** | 0 | 28 | 56 | 84 | 119 | 143 | 24 |
| **v130** | 0 | 38 | 56 | 84 | 119 | 141 | 22 |
| **v131** | 0 | 28 | 56 | 84 | 119 | 143 | 24 |
| **v133** | 0 | 38 | 56 | 84 | 119 | 141 | 22 |
| **v134** | 0 | 28 | 56 | 84 | 119 | 143 | 24 |
|  |  |  |  |  |  |  |  |
| **Non-protected**  **subjects (n=1)** | **Imm1** | **Imm2** | **Imm3** | **Imm4** | **Imm5** | **Total** | **CHMI interval** |
| **110** | 0 | 28 | 56 | 84 | 119 | 143 | 24 |

Subjects were immunized five times at the days shown, and CHMI was received 22-24 days later.

**Table S8: Cohort 1: Salivary gland scores during immunization and CHMI**

| **All subjects (n=11)** | **Imm1** | **Imm2** | **Imm3** | **Imm4** | **Imm5** | **Median** | **CHMI** |
| --- | --- | --- | --- | --- | --- | --- | --- |
| **v2** | 3.5 | 3.2 | 2.8 | 3.2 | 3.1 | 3.2 | 3.2 |
| **v5** | 3.2 | 3.7 | 2.9 | 2.9 | 3.3 | 3.2 | 3.8 |
| **v21** | 3.3 | 3.1 | 3.2 | 3.2 | 3.3 | 3.2 | 3.8 |
| **v33** | 3.6 | 3.4 | 2.9 | 3.2 | 3.2 | 3.2 | 3.6 |
| **v39** | 3.3 | 3.3 | 2.9 | 2.8 | 3.4 | 3.3 | 2.8 |
| **v45** | 3.2 | 3.3 | 2.7 | 3.3 | 3.0 | 3.3 | 3.6 |
| **v57** | 3.3 | 3.0 | 3.0 | 3.3 | 3.2 | 3.2 | 3.6 |
| **v60** | 3.1 | 3.5 | 3.1 | 3.1 | 3.1 | 3.1 | 3.8 |
| **v61** | 3.1 | 3.3 | 3.5 | 3.3 | 3.2 | 3.3 | 3.6 |
| **v71** | 3.3 | 3.1 | 3.0 |  | 3.2 | 3.2 | 3.6 |
| **v78** | 3.4 | 3.3 | 2.7 | 3.2 | 3.3 | 3.3 | 3.0 |
|  |  |  |  |  |  |  |  |
| **Protected subjects (n=6)** | **Imm1** | **Imm2** | **Imm3** | **Imm4** | **Imm5** | **Median** | **CHMI** |
| **v2** | 3.5 | 3.2 | 2.8 | 3.2 | 3.1 | 3.2 | 3.2 |
| **v39** | 3.3 | 3.3 | 2.9 | 2.8 | 3.4 | 3.3 | 2.8 |
| **v45** | 3.2 | 3.3 | 2.7 | 3.3 | 3.0 | 3.3 | 3.6 |
| **v57** | 3.3 | 3.0 | 3.0 | 3.3 | 3.2 | 3.2 | 3.6 |
| **v60** | 3.1 | 3.5 | 3.1 | 3.1 | 3.1 | 3.1 | 3.8 |
| **v78** | 3.4 | 3.3 | 2.7 | 3.2 | 3.3 | 3.3 | 3.0 |
|  |  |  |  |  |  |  |  |
| **Non-protected**  **Subjects (n=5)** | **Imm1** | **Imm2** | **Imm3** | **Imm4** | **Imm5** | **Median** | **CHMI** |
| **v5** | 3.5 | 3.2 | 2.8 | 3.2 | 3.1 | 3.2 | 3.2 |
| **v21** | 3.3 | 3.1 | 3.2 | 3.2 | 3.3 | 3.2 | 3.8 |
| **v33** | 3.6 | 3.4 | 2.9 | 3.2 | 3.2 | 3.2 | 3.6 |
| **v61** | 3.1 | 3.3 | 3.5 | 3.3 | 3.2 | 3.3 | 3.6 |
| **v71** | 3.3 | 3.1 | 3.0 |  | 3.2 | 3.2 | 3.6 |

Mean gland scores of infectious mosquitoes used at each immunization and CHMI.

**Table S9: Cohort 2: Salivary gland scores during immunization and CHMI**

| **All subjects (n=10)** | **Imm1** | **Imm2** | **Imm3** | **Imm4** | **Imm5** | **Median** | **CHMI** |
| --- | --- | --- | --- | --- | --- | --- | --- |
| **v102** | 2.9 | 3.4 | 3.8 | 3.5 | 3.8 | 3.5 | 3.2 |
| **v103** | 3.4 | 3.7 | 3.8 | 3.8 | 3.7 | 3.7 | 3.6 |
| **v110** | 3.5 | 3.8 | 3.9 | 3.7 | 3.7 | 3.7 | 3.6 |
| **v114** | 3.6 | 3.6 | 3.3 | 3.4 | 3.8 | 3.6 | 3.6 |
| **v120** | 3.2 | 3.6 | 3.5 | 3.5 | 3.4 | 3.5 | 3.8 |
| **v127** | 3.2 | 3.9 | 3.8 | 3.6 | 3.8 | 3.8 | 3.8 |
| **v130** | 3.0 | 3.8 | 3.7 | 3.6 | 3.7 | 3.7 | 3.4 |
| **v131** | 3.5 | 3.5 | 3.5 | 3.5 | 3.9 | 3.5 | 3.0 |
| **v133** | 3.4 | 3.6 | 3.4 | 3.6 | 3.6 | 3.6 | 3.4 |
| **v134** | 3.1 | 3.4 | 3.4 | 3.7 | 3.5 | 3.4 | 3.2 |
|  |  |  |  |  |  |  |  |
| **Protected subjects (n=9)** | **Imm1** | **Imm2** | **Imm3** | **Imm4** | **Imm5** |  | **CHMI** |
| **v102** | 2.9 | 3.4 | 3.8 | 3.5 | 3.8 | 3.5 | 3.2 |
| **v103** | 3.4 | 3.7 | 3.8 | 3.8 | 3.7 | 3.7 | 3.6 |
| **v114** | 3.5 | 3.8 | 3.9 | 3.7 | 3.7 | 3.7 | 3.6 |
| **v120** | 3.2 | 3.6 | 3.5 | 3.5 | 3.4 | 3.5 | 3.8 |
| **v127** | 3.2 | 3.9 | 3.8 | 3.6 | 3.8 | 3.8 | 3.8 |
| **v130** | 3.0 | 3.8 | 3.7 | 3.6 | 3.7 | 3.7 | 3.4 |
| **v131** | 3.5 | 3.5 | 3.5 | 3.5 | 3.9 | 3.5 | 3.0 |
| **v133** | 3.4 | 3.6 | 3.4 | 3.6 | 3.6 | 3.6 | 3.4 |
| **v134** | 3.1 | 3.4 | 3.4 | 3.7 | 3.5 | 3.4 | 3.2 |
|  |  |  |  |  |  |  |  |
| **Non-protected**  **subjects (n=1)** | **Imm1** | **Imm2** | **Imm3** | **Imm4** | **Imm5** | **Median** | **CHMI** |
| **110** | 3.5 | 3.8 | 3.9 | 3.7 | 3.7 | 3.7 | 3.6 |

Mean gland scores of infectious mosquitoes used at each immunization and CHMI.
