# Supplementary material for: IMRAS—A clinical trial of mosquito-bite immunization with live, radiation-attenuated P. falciparum sporozoites: Impact of immunization parameters on protective efficacy and generation of a repository of immunologic reagents
Source: PLoS One. 2020 Jun 17;15(6):e0233840. doi: 10.1371/journal.pone.0233840 (PMC7299375; doi:10.1371/journal.pone.0233840)
Supplement: S1 File — (PDF) [file pone.0233840.s005.pdf]

Phase 1 Trial with Challenge to Assess the Safety and Biomarkers of Protection in Malaria-naïve Adults of Immunization via Mosquito Bite with Radiation-Attenuated *Plasmodium falciparum* Sporozoites (IMRAS)

Sponsor
The Surgeon General, Department of the Army

Sponsor's Representative
Kenneth A. Bertram, MD, PhD
US Army Medical Research and Materiel Command (USAMRMC)
1430 Veterans Drive, Fort Detrick, MD 21702-5009
Telephone: 301-619-0317; DSN: 343-0317
Fax: 301-619-0197
E-mail: usarmy.detrick.medcom-usammda.mbx.usamrmc-regulatory-affairs@mail.mil

Research Monitor
Janine Danko, MD, MPH, CDR, MC, USN
Walter Reed Army National Military Medical Center (WRNMMC)
8901 Wisconsin Avenue
Bethesda, MD 20889
301-295-8279
Fax: 301-295-8025
Email: janine.r.danko.mil@mail.mil

Principal Investigator:
Eileen D. Franke Villasante, PhD
United States Military Malaria Vaccine Program (USMMVP)
Naval Medical Research Center (NMRC)
503 Robert Grant Avenue
Silver Spring, MD 20910
301-319-2076
Fax: 301-319-7545
Email: eileen.d.villasante.civ@mail.mil

Clinical Investigators:
United States Military Malaria Vaccine Program
Naval Medical Research Center
503 Robert Grant Avenue
Silver Spring, MD 20910
Judith E. Epstein, MD, CAPT, MC, USN
301-252-9026
E-mail: Judith.e.epstein.mil@mail.mil

Walter Reed Army Institute of Research
503 Robert Grant Avenue
Silver Spring, MD 20910
James Moon, MD, LTC, USA
301-319-9176
E-mail: james.e.moon.mil@mail.mil

Michele Spring, MD
301-319-9996
E-mail: michele.d.spring.ctr@mail.mil

Cardiologist (Consultant):

Todd Villines, MD, LTC, MC, USA
301-295-7839
Email: todd.c.villines.mil@health.mil

Research Investigators:

US Military Malaria Vaccine Program (USMMVP)
NMRC Component
Malaria Program
503 Robert Grant Avenue
Silver Spring, MD 20910

Keith Limbach, PhD
301-319-7657
Fax: 301-319-7545
Email: keith.j.limbach.ctr@mail.mil

Noelle Patterson, MS
301-319-7362
Fax: 301-319-7545
Email: noelle.b.patterson.ctr@mail.mil

Martha Sedegah, PhD
301-319-7586
Fax: 301-319-7545
Email: martha.sedegah.civ@mail.mil

Emily Smith, PhD
301-319-7560
Fax: 301-319-7545
Email: emily.c.smith40.ctr@mail.mil

Cathy Zou, PhD
301-319-3016
Fax: 301-319-7545
Email: xiaoyan.n.zou.ctr@mail.mil

Army Component

Urszula Krzych, PhD
301-319-9558
Fax: 301-319-9358
Email: urszula.krzych1.civ@mail.mil

Elke Bergmann-Leitner, PhD
301-319-9278
Fax: 301-319-9358
Email: elke.s.bergmann-leitner.civ@mail.mil

Christian Darko, PhD
301-319-9674
Fax: 301-319-9358
Email: christian.a.darko2.ctr@mail.mil

Jeffrey Teigler, PhD
301-319-9284
Fax: 301-319-9391
Email: jteigler@hivresearch.org

WRAIR Entomology

Megan Dowler
301-319-9162
Fax: 301-319-9290
Email: megan.g.dowler.civ@mail.mil

Seattle Children's Research Institute
307 Westlake Avenue N, Suite 500
Seattle, WA 98109-5219

Stefan Kappe, PhD
Professor and Malaria Program Director (Co-PI)
206-884-3180
Email: stefan.kappe@seattlechildrens.org

Nana Minkah, PhD
Nana.Minkah@seattlechildrens.org

Ken Stuart, PhD
President Emeritus/ Founder (Contact PI at SBM)
206-884-3227
Email: ken.stuart@seattlechildrens.org

Tracie Vongoedert
Manager Regulatory & Clinical Affairs
206-256-7326
Fax: 206-256-7229
Email: tracie.vongoedert@seattlebiomed.org

Uniformed Services University of the Health Sciences (USUHS)

V. Ann Stewart, DVM, PhD
301-295-3139
Email: astewart@usuhs.edu

CAMRIS International

Joao Aguiar, PhD
301-319-7580
Fax: 301-319-7545
Email: Ajanaus@gmail.com

Atreca, Inc

Daniel Emerling, PhD
VP of Research
510-333-5955
Email: d.emerling@atreca.com

Genocea Biosciences, Inc

Jean-Luc Bodmer, PhD
Director of Discovery
617-876-8191 ext. 8243
Email: jean-luc.bodmer@genocea.com

Fred Hutchinson Cancer Research Center

Stephen C. De Rosa, M.D.
206-667-1681
Fax: 206-667-6608
Email: sderosa@fredhutch.org

University of Washington

Marion Pepper, PhD
Associate Professor, Dept. of Immunology
206-221-8261
Fax: 206-616-4274
Email: mpepper@uw.edu

Lauren Rodda, PhD.
Postdoctoral Fellow
Department of Immunology
Email: lrodda@uw.edu

Clinical Trial Site

Naval Medical Research Center Clinical Trials Center (CTC)
8901 Wisconsin Avenue, Building 17, Suite 2B
Bethesda, MD 20889
301-295-4298

Immunizations and malaria challenge for the subjects enrolled and followed at NMRC CTC will take place at:
Walter Reed Army Institute of Research (WRAIR)
503 Robert Grant Avenue, Insectary (third floor)
Silver Spring, MD 20910

Site for post-challenge overnight stays:
Hotel in close proximity to NMRC CTC

Clinical Laboratories and Other Departments/Institutions Involved in the Trial

**Site Investigational Product Accountability:**	
**Clinical Laboratory:**	Walter Reed National Military Medical Center<br>Building 10<br>301-295-0250<br>Fax: 301-319-8293
**HLA Typing:**	C.W. Bill Young Department of Defense Marrow Donor Program<br>11333 Woodglen Drive, 3rd Floor<br>Rockville, Maryland 20852-3072<br>301-998-8900<br>Fax: 301-984-8856
**Contract Laboratory:**	Antigen Discovery Inc,<br>1 Technology Drive, Suite E309<br>Irvine, CA 92618<br>949-679-4068<br>Fax: 949-679-4069<br><br>BGI Americas Corporation<br>One Broadway, 3rd Floor<br>Cambridge, MA 02142<br>617-500-2741<br>Fax: 617-500-2742
**Research Laboratory:**	US Military Malaria Vaccine Program (USMMVP), NMRC<br>US Military Malaria Vaccine Program (USMMVP), WRAIR<br>Entomology Branch, WRAIR<br>Seattle Children', 307 Westlake Avenue N<br>Seattle, WA 98109-5219
**Project Management:**	
**Biostatistician:**	Meng Shi<br>Walter Reed Army Institute of Research<br>301-319-9763<br>E-mail: meng.shi.ctr@mail.mil

Quality Assurance:

Clinical Trial Site

Anatalio Reyes, BS, MS
Quality Assurance Manager
301-295-1095
Fax: 301-295-8025
Email: anatalio.e.reyes.ctr@mail.mil

WRAIR Insectary

Michael Hrycyk
Quality Assurance Scientist
301-319-2002
E-mail: michael.hrycyk.civ@mail.mil

Data Management:

Naval Medical Research Center Clinical Trials Center
8901 Wisconsin Avenue Building 17, Suite 2B
Bethesda, MD 20889
Contact Person: Mimi Wong, BS
301-319-4349
E-mail: mimi.a.wong.ctr@mail.mil

U. S. Army Medical Materiel Development Activity (USAMMDA)
Clinical Services Support Division
1430 Veterans Drive
Fort Detrick, MD 21702
Contact Person: Dixion Rwakasyaguri, MBA, BSISM
301-619-9127
Fax: 301-619-0197
Email: dixion.t.rwakasyaguri.civ@mail.mil

External Monitor:

U. S. Army Medical Materiel Development Activity (USAMMDA)
1430 Veterans Drive
Fort Detrick, MD 21702

Institutional Review Board(s):

Office of Research Administration
Naval Medical Research Center
503 Robert Grant Avenue
Silver Spring, MD 20910
301-319-7276
Fax: 301-319-7277
FWA00000152 IRB00001008

Human Research Protections Office
US Army Medical Research and Materiel Command
ATTN: MCMR-RPI
504 Scott Street
Fort Detrick, MD 20702-5012
Telephone: (301) 619-6240/DSN 343-6240
Fax: (301) 619-4165
Email: usarmy.detrick.medcom-usamrmc.other.hrpo@mail.mil

FWA A20000 IRB00007718

**Collaborating Institutions:**	Seattle Children's Research Institute<br>307 West Lake Avenue North, Suite 500<br>Seattle, WA 98109-5219<br>Contact Person: Ken Stuart, PhD
	Atreca, Inc<br>75 Shoreway Road,<br>San Carlos, CA 94070<br>408-810-6224<br>Contact Person: Paulette Dillon
	Bill and Melinda Gates Foundation<br>PO Box 23350<br>Seattle, WA 98102<br>206-709-3100<br>Contact Person: Janice Culpepper, PhD

Investigator’s Agreement

Phase 1 Trial with Challenge to Assess the Safety and Biomarkers of Protection in Malaria-naïve Adults of Immunization via Mosquito Bite with Radiation-Attenuated *Plasmodium falciparum* Sporozoites (IMRAS)

“I have read this protocol and agree to conduct the study as outlined herein in accordance with International Conference on Harmonization Good Clinical Practice Guideline and FDA, DoD, and United States Army Regulations.”

______________________________________ ______________________________

Eileen D. Franke Villasante, PhD
Principal Investigator
Naval Medical Research Center
Infectious Diseases Directorate
US Military Malaria Vaccine Program
Naval Medical Research Center
503 Robert Grant Avenue
Silver Spring, MD 20910
301-252-9026

Date

Procedures in Case of Emergency

Table 1: Emergency Contact Information

Role in Study	Name	Address and Telephone Number
Principal Investigator	Eileen D. Franke Villasante, PhD	Naval Medical Research Center<br>Infectious Diseases Directorate<br>US Military Malaria Vaccine Program<br>503 Robert Grant Avenue, Silver Spring, MD 20910<br><br>Mobile: 301-252-9026
Clinical Study Team	Yolanda Alcorta, MS, MAT<br>Santina Maiolatesi, BA<br>Anatalio Reyes, BSMT, SQSM<br>Mimi Wong, BS	Naval Medical Research Center<br>Clinical Trials Center<br>8901 Wisconsin Avenue, Building 17(b), Suite 2b, Bethesda, MD 20889<br>Telephone: 301-295-4298<br>Mobile: 301-233-9640
Study Coordinator/Protocol Nurse	Ivelese Guzman, LPN	
NMRC Regulatory		
Research Monitor	Janine Danko, MD, MPH<br>CDR, MC, USN	301-295-8279
USAMMDA Clinical Services Support Division	Director	USAMMDA<br>1430 Veterans Drive, Ft. Detrick, MD 21702-5009<br>301-619-1095
Sponsor’s Representative	Principal Assistant for Acquisition	USAMRMC<br>1430 Veterans Drive, Ft. Detrick, MD 21702-5009<br>301-619-0317<br>usarmy.detrick.medcom-usammda.mbx.usamrmc-regulatory-affairs@mail.mil
Institutional Review Board	NMRC Office of Research Administration	301- 319-7276
	ORP HRPO	301-619-6240

2. Synopsis

Name of Sponsor/Company:

The Surgeon General, Department of the Army

Name of Investigational Product(s):

Radiation-attenuated *Plasmodium falciparum* sporozoites administered by the bite of infected *Anopheles stephensi* mosquitoes

Name of Active Ingredient(s):

Radiation-attenuated *Plasmodium falciparum* sporozoites

Title of Study:

Phase 1 Trial with Challenge to Assess the Safety and Biomarkers of Protection in Malaria-naïve Adults of Immunization via Mosquito Bite with Radiation-Attenuated *Plasmodium falciparum* Sporozoites (IMRAS)

Study Center(s):

NMRC Clinical Trials Center

Principal Investigator:

Eileen D. Franke Villasante, PhD

**Studied Period (years):**	**Phase of development:**
Estimated date first subject enrolled: 2013<br>Estimated date last subject completed: 2016	1

Objectives:

Primary:

1. Assess safety and tolerability in malaria-naïve adults of immunization with radiation-attenuated *P falciparum* sporozoites (*Pf*RAS) administered via mosquito bites compared with mock immunization via noninfectious mosquito bites.
2. Induce protective immunity against controlled human malaria infection (CHMI) in approximately 50% of study subjects via immunization with *Pf*RAS.

Secondary:

a. Identify biomarkers of protection, including host response and antigenic targets, by comparing protected, nonprotected, and mock-immunized subjects.
b. Identify immune components of high-grade, durable protection in hyperimmunized subjects.

Methodology:

The clinical study design is an open-label safety and biomarkers of protection study in healthy malaria-naïve adults, who will receive bites from *Anopheles stephensi* mosquitoes either infected with *Pf*RAS (true-immunization) or noninfected (mock-immunization). Additionally, this study is a comprehensive, systems biology-based effort to identify and validate biomarkers of protection with *Pf*RAS immunization, comparing sterilely protected to nonprotected study subjects. The goal of the trial design is to achieve approximately 50% sterile protection in order to facilitate the identification of biomarkers and correlates of protection.

Following true-immunization or mock-immunization, study subjects and nonimmunized infectivity controls will receive a challenge via the bites of 5 *An stephensi* mosquitoes carrying infectious *P falciparum* sporozoites within a controlled clinical environment (controlled human malaria infection, CHMI) to determine the level of sterile protection.

To increase sample size and enable flexibility toward achieving the goal of approximately 50% sterile protection, there will be 2 cohorts; the first cohort will be initiated prior to the second. Each cohort will include true-immunized and mock-immunized study subjects and CHMI infectivity controls. Both cohorts will receive identical immunization regimens if protection in the first cohort is 40-60%; alternatively, the second cohort will receive more or fewer mosquito bites as part of the immunization regimens if protection in the first cohort is < 40% or > 60%, respectively.

Protected subjects from Cohort 1 will be offered the option to enroll in a continuation phase of the trial that will address secondary objective b (hyperimmunity). Subjects electing this option will receive 3 secondary

immunizations and a secondary challenge in conjunction with Cohort 2. This continuation phase will allow the exploration of the high-grade, durable immunity that is generated following primary challenge and boosting immunizations.

Cohort 1:

Twelve malaria-naïve adults will receive 5 doses of approximately 200 infectious bites (200-400 bites total) from *Pf*RAS-infected mosquitoes (true-immunization) and 4 additional subjects will receive the same from irradiated, uninfected mosquitoes (mock-immunization). The target dose is 960 infectious bites for the group mean. This should result in full sterile protective immunity in some true-immunized individuals. The target of 960 bites was selected because earlier studies (section 5.6.2) suggest that this is an approximate point of transition between nonprotection and protection. Following the fifth immunization, all immunized subjects (true-immunized and mock-immunized) plus 6 nonimmunized infectivity controls will undergo CHMI approximately 3 weeks after the fifth immunization session.

Trial Design

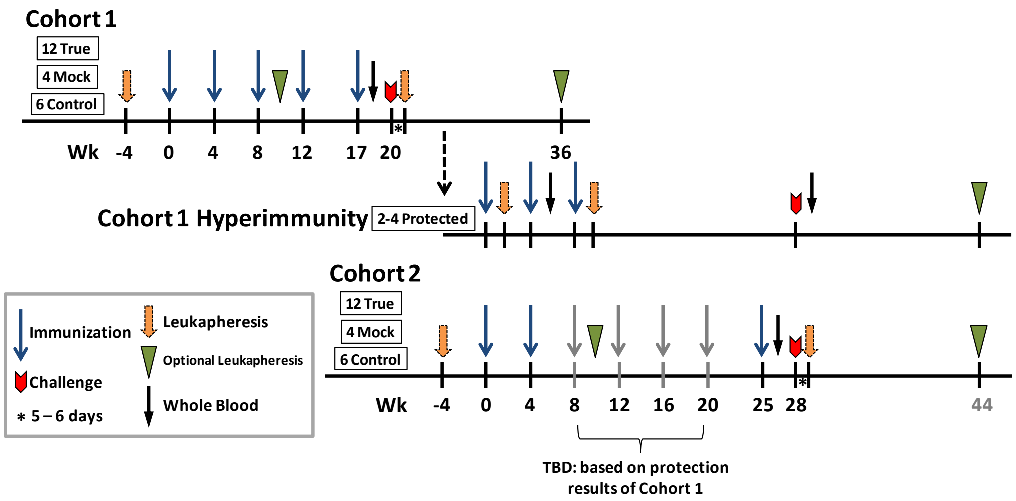

The interval between the penultimate and final immunization will be 5 weeks, rather than 4 weeks.

Cohort 2:

An adjustable dosing schedule for Cohort 2 will be used to balance out a skewed protection result in Cohort 1 should this occur (< 40% or > 60% protection), in order to achieve approximately 50% sterile protection averaged over the 2 cohorts. The first immunization for Cohort 2 will take place after the challenge results for Cohort 1 are known, allowing adjustment in the number of immunizations in Cohort 2.

Enrollment in Cohort 2 will mirror enrollment in Cohort 1: 12 malaria-naïve adults will receive doses of approximately 200 infectious bites from *Pf*RAS-infected mosquitoes (true-immunization), 4 subjects will receive the same from irradiated, uninfected mosquitoes (mock-immunization), and 6 subjects will be included as nonimmunized infectivity controls. Depending upon the proportion of subjects who are sterilely protected in Cohort 1, the Cohort 2 dosing regimen will be between 3 to 7 total immunizations.

Cohort 1 Hyperimmunity Sub-cohort (Continuation Phase):

Limited data from the literature indicate that both primary challenge as well as any subsequent boosting immunizations strengthen the protective immunity inducted by *Pf*RAS. In order to study this "second tier" of immunity, the trial includes an optional post-CHMI continuation phase, called Cohort 1 Hyperimmunity Sub-cohort. These research subjects will permit an examination of the immune response post-primary challenge, post-secondary immunizations, and post-secondary challenge. This approach will allow for the immunological profiling of high-grade, durable immunity, including its generation following primary challenge and during the course of 3 boosting immunizations. It will also allow a comparison of this durable immunity with the waning of immunity over time in study subjects who do not elect to enroll in the continuation phase.

We anticipate that 2-6 protected subjects from Cohort 1 will be willing to continue participation in this fashion, and will be assigned to the Cohort 1 Hyperimmunity Sub-cohort. This continuation phase sub-cohort will occur in conjunction with Cohort 2 in order to maximize resource utilization and to overcome logistical constraints. It is uncertain how long the interval between the conclusion of Cohort 1 and the initiation of Cohort 2 will be; however, it is estimated that it will be approximately 3 months. Therefore, Cohort 1 Hyperimmunity Sub-Cohort subjects will follow the prescribed sampling schedule, safety lab, and safety visit schedule for Cohort 1 until the initiation of Cohort 2. At that time, the continuation Sub-cohort will follow a unique sampling schedule, but will adhere to the Cohort 2 safety lab and safety visit schedule. Of note, the Hyperimmunity Sub-cohort will only receive the first 3 immunizations scheduled for Cohort 2 regardless of the final Cohort 2 dosing regimen. Following the third immunization (secondary immunization 3), the sub-cohort will be scheduled for a secondary challenge to correspond with the primary challenge of Cohort 2. Due to the adjustable dosing schedule for Cohort 2, the interval between the third (final) secondary immunization of the sub-cohort and the secondary challenge of this Sub-cohort may vary from 3 to 19 weeks.

Safety Monitoring:

Safety monitoring will occur to assess adverse events associated with immunization and CHMI. Historically, the primary source of adverse events associated with this type of immunization is reaction to mosquito salivary gland antigens. Although most individuals tolerate the bites well, some may need to be withdrawn from the study because of their reactions. Decisions about need to withdraw a subject will be based on the Principal Investigator's (PI's) clinical judgment in consultation with the research monitor.

Challenge Procedure:

The procedure for conducting CHMI requires 5 infectious mosquito bites. Beginning 7 days after the bites, subjects will be monitored daily for parasitemia by microscopic examination of thick blood smears, with immediate anti-malarial treatment upon detection of parasites in the blood. In the absence of sterile protection, most subjects will develop patent parasitemia by microscopy on days 9-14 (range 7 to 23).

Blood-stage malaria infection causes a predictable systemic illness characterized by headache, malaise, and fatigue, often accompanied by fever and chills. Study subjects are expected to recover rapidly and completely with no residual malaria infection.

Attenuation of Sporozoites:

Immunizations and CHMI will be conducted with the NF54 *P falciparum* strain. (Alternatively, the 3D7 strain may be used, if necessary.) Infected mosquitoes will be irradiated with 15,000 cGy. In our experience and within the world's literature (see section 5.6.2), there has never been a breakthrough *P falciparum* infection following a 15,000 cGy irradiation dose. Nevertheless, study subjects will be alerted to this risk and monitored for signs or symptoms of breakthrough parasitemia throughout the immunization phase of the trial in conjunction with routine post-immunization visits. However no blood smears will be collected prior to the CHMI unless clinically indicated based upon the judgment of the PI in consultation with the research monitor.

Leukapheresis Procedure Requirement:

This study requires a large amount of peripheral blood mononuclear cells (PBMCs) to identify biomarkers and correlates of protection. Leukapheresis will be performed to collect this large quantity of cells. The procedure is performed routinely by blood banks to obtain blood components from healthy subjects, in the same way that whole blood is donated. In this study, we are aiming for about 4 billion PBMCs harvested per procedure (about 2 hours on the apheresis machine).

Referring to the trial design found earlier, a large pre-challenge whole blood draw is shown as a black arrow and leukapheresis procedures as orange dotted arrows. The pre-immunization and post-challenge leukapheresis procedures are planned procedures in this study as these samples are necessary to address some of the objectives of the study. An optional leukapheresis procedure is shown as a green triangle. This procedure is optional in order to reduce participant fatigue and drop-out. Further, participation in this optional leukapheresis procedure will be limited to a maximum of 50% of the subjects. In the event that more than 50% of the study subjects would like to proceed with the optional leukapheresis procedures, the subjects that provided the greatest yield of cells will be selected for the optional procedures. Per the American Association of Blood Banks (AABB) 5.4.1A (3), healthy donors should be deferred for greater than or equal to 2 days after plasma-, platelet-, or leukapheresis. In order to maintain a very large safety factor, leukapheresis procedures for this trial will be separated by at least 2 weeks.

Many smaller volume whole blood draws (not shown in the figure) will be collected throughout the clinical trial in order to support the study objectives and monitor research subject safety. An additional optional leukapheresis procedure will be offered at approximately 4-6 months post-challenge. All study subjects (100%) may participate

in this procedure. Subjects participating in the Hyperimmunity Continuation Phase will undergo 2 additional leukapheresis procedures following the first and third secondary immunizations. The study event schedule details the blood volumes, timepoints, and reason for sample collection for all sample collections.

Staggered Challenge and Leukapheresis Procedures:

A staggered challenge and leukapheresis schedule will be followed within each cohort to overcome logistical limitations.

Estimated Number of Subjects Screened:

200

Maximum Number of Subjects Enrolled:

60 (enrollment is defined as a subject who has met all inclusion and exclusion criteria and who completes the first study-specific procedure (eg leukapheresis, immunization, CHMI); true- and mock-immunized subjects that fail to complete the planned pre-immunization leukapheresis may be enrolled into the study at the time of Immunization #1).

Number of Subjects (planned):

Planned enrollment goal of 24 true-immunized, 8 mock-immunized, 12 infectivity controls, and 16 alternates. The planned enrollment goals may be exceeded for each category secondary to the replacement of subjects with alternates (eg withdrawal of consent prior to Immunization #1).

Main Inclusion Criteria:

Subjects must meet all of the following criteria to participate in this study:

- Healthy adults (male or non-pregnant, non-breastfeeding female) 18-50 years of age (inclusive).
- Available and willing to participate for duration of study.
- Able and willing to provide written informed consent.
- Able to complete an Assessment of Understanding with a score of at least 70% correct.
- In good general health with no clinically significant health problems as established by medical history, physical exam and laboratory screening.
- Females of childbearing potential must have a negative pregnancy test at screening and agree to not become pregnant or breastfeed for the duration of the study. She must be willing to use a reliable form of contraception during the study.
    - Reliable forms of birth control include use of condoms, diaphragm or cervical cap, birth control pills, IUD or sperm killing products.
- Agree to refrain from blood donation (except as required in this study) for 3 years following *P falciparum* challenge.
- Agree not to travel to a malaria-endemic region during the study.
- Good peripheral venous access.

Main Exclusion Criteria:

Subject must not meet any of the following criteria in order to participate in this study:

- Positive Human Immunodeficiency Virus (HIV), Hepatitis B surface antigen (HBsAg), or Hepatitis C virus (HCV) serology.
- Positive sickle cell screening test, including evidence of sickle trait.
- Reactivity by CSP or AMA1 ELISpot assay or ELISA as determined by IMRAS Study Specific Procedure #204
- Anemia (below normal reference laboratory value of hemoglobin) on screening.
- Weight less than 110 pounds (this does not apply to infectivity controls as it is a weight cut-off for subjects undergoing leukapheresis procedure).
- Any history of malaria infection or travel to a malaria endemic region within 6 months prior to first immunization.
- History of long-term residence (> 5 years) in area known to have significant transmission of *P falciparum* [cumulative lifetime exposure].

- Use of systemic immunosuppressant pharmacotherapy for greater than 10 days within 60 days of scheduled first immunization (inhaled and topical steroids are allowed; short duration or tapered corticosteroid regimens of 10 days or less that have been discontinued prior to first immunization are allowed).
- Current significant medical condition (cardiovascular, hepatic, renal, pulmonary, or hematological) or evidence of any other serious underlying medical condition identified by medical history, physical examination, or laboratory examination (includes bleeding disorders).
- Plan for surgery between enrollment and day 28 post-challenge (minor procedures, elective corrective vision surgery, and dental procedures are allowed).
- Receipt of immunoglobulin and/or any blood products within 90 days of scheduled leukapheresis or immunization.
- Evidence of increased cardiovascular disease risk (defined as > 5%-10%, 5-year risk) as determined by the method of Gaziano (2008). Risk factors include sex, age (years), systolic blood pressure (mm Hg), smoking status, body mass index (BMI, kg/m^2), reported diabetes status, and blood pressure.
- An abnormal electrocardiogram (ECG), defined as one showing pathologic Q waves and significant ST-T wave changes; left ventricular hypertrophy; any non-sinus rhythm excluding isolated premature atrial contractions; right or left bundle branch block; or advanced (secondary or tertiary) A-V heart block.
- History of a splenectomy.
- History of any other illness or condition which, in the investigator’s judgment, may substantially increase the risk associated with the subject’s participation in the protocol or compromise the scientific objectives. This may include psychiatric disorders (such as personality disorders, anxiety disorders, or schizophrenia) or behavioral tendencies (including active alcohol or drug abuse) discovered during the screening process that in the opinion of the investigator would make compliance with the protocol difficult.
- History of anaphylactic or other severe response to mosquito bites (history of local hypersensitivity reactions is allowed).
- History of retinal disease, visual field changes, psoriasis, porphyria, or known allergy to the anti-malarial chloroquine phosphate, which will be used to treat subjects developing malaria after CHMI.
- Participation in any study involving any investigational vaccine or drug within 30 days prior to the screening visit, or plan to participate in another investigational vaccine/drug research during or within 1 month following participation in this study.
- Use or planned use of any drug with antimalarial activity that would coincide with immunization or challenge.
- Anticipated use of medications known to cause drug reactions with chloroquine or atovaquone-proguanil (Malarone) such as cimetidine, metoclopramide, antacids, and kaolin during the day 7 to 28 post-challenge period.
- Any other significant findings which, in the investigator’s judgment, may substantially increase the risk associated with the subject’s participation in the study or compromise the scientific objectives.

Investigational Product:

Radiation-attenuated *Plasmodium falciparum* sporozoites

Dosage:

Approximately 200 infectious bites (200-400 bites total) per immunization session

Mode of Administration:

Administered by the bite of infected *An stephensi* mosquitoes

Duration of Study:

The projected study period spans from the start of recruitment (estimated fall- 2013) through the final visit for Cohort 2. The projected study period spans approximately 120 weeks. Cohort 2 will be based upon a variable dosing regimen; therefore, it may range between 112 to 128 weeks.

Duration for Each Participant:

Cohort 1:

Each subject will actively participate for approximately 52 weeks (screening, immunization, pre- and post-immunization leukapheresis and follow-up).

Cohort 2:
Each subject will actively participate for approximately 52 weeks with a range between 46 to 60 weeks depending upon dosing regimen.

Cohort 1 Hyperimmunity Sub-cohort:

Each subject (up to a maximum of 6) who elects to participate in the continuation phase will actively participate for approximately 120 weeks (comprising the duration of Cohort 1 immunization and challenge, the interval between completion of Cohort 1 and initiation of Cohort 2, and the duration of Cohort 2 immunization and challenge).

Criteria for Evaluation:

Primary Endpoints:

1. Occurrence of solicited adverse events (AE) from administration of study immunization (*Pf*RAS) through 7 days post-dosing.
2. Occurrence of unsolicited AEs from administration of immunization through 14 days post-dosing.
3. Occurrence of laboratory AEs from administration of study immunization through 7 days post-dosing.
4. Occurrence of serious adverse events (SAE) from administration of immunization through the duration of the trial.
5. Occurrence of signs and symptoms related to malaria infection starting 7 days post CHMI (these will not be recorded as adverse events because they are expected as a result of malaria infection).
6. Development of parasitemia and time to parasitemia after malaria challenge.

Secondary Endpoints:

1. Collection and storage of PBMCs, serum, and whole blood for use in a comprehensive effort to identify and validate biomarkers of immunological protection associated with immunizing human subjects with *Pf*RAS, comparing read-outs between protected and non-protected subjects and between immunized and mock-immunized subjects.

Sample Size Selection:

The sample size of this study is limited by the complexity of the immunization and leukapheresis procedures. Twenty-two subjects per cohort (12 true-immunized, 4 mock-immunized, 6 infectivity controls), along with a maximum of 6 continuation phase subjects, is the limit achievable for immunizations, challenge, and leukapheresis procedures. This sample size is adequate to address the primary scientific requirements of the study.

3. Table of Contents, List of Tables, and List of Figures

Table of Contents

1. Title Page 1
2. Synopsis 10
3. Table of Contents, List of Tables, and List of Figures 16
4. List of Abbreviations and Definitions of Terms 21
5. Introduction 24
5.1. Need for a Malaria Vaccine 24
5.2. Military Relevance 24
5.3. Rationale for Study 24
5.4. Summary 27
5.5. Name and Description of the Investigational Product 28
5.6. Summary of Nonclinical and Clinical Trials 28
5.6.1. Nonclinical Studies with the Radiation Attenuated Sporozoite Vaccine 28
5.6.2. Clinical Studies 29
5.6.2.1. Safety Summary 29
5.6.2.2. Effectiveness Summary 31
5.6.2.3. Two-tiered Immunity: Effect of Primary Challenge and Booster Immunizations 34
5.7. Known and Potential Risks and Benefits to Human Subjects 36
5.7.1. Risks/Discomfort to Subjects and Precautions to Minimize Risk 36
5.7.1.1. Risks Associated with Immunization by Mosquito Bites 36
5.7.1.2. Risks Associated with CHMI 37
5.7.1.3. Risks Associated with Antimalarial Medications 39
5.7.1.4. Risks Associated with Leukapheresis 40
5.7.1.5. Pregnancy 40
5.7.1.6. Lactation 40
5.7.1.7. Risks Associated with Venipuncture/Blood Drawing 40
5.7.1.8. Allergic Reaction 41
5.7.1.9. Unknown Risks 41
5.7.2. Alternatives to This IND Product or Study 41
5.7.3. Intended Benefit for Subjects 41
5.7.4. Risks to the Study Personnel and the Environment 42
5.8. Route of Administration, Dosage Regimen, Treatment Period, and Justification 42
5.8.1. Administration 42
5.8.2. Dosage 42
5.8.3. Compliance Statement 43
5.9. Study Population 44
5.10. Study Site 44
6. Trial Objectives and Purpose 45
6.1. Primary Objective 45
6.2. Secondary Objectives 45
7. Trial Design 46
7.1. Study Endpoints 46
7.1.1. Primary Endpoints 46
7.1.2. Secondary Endpoints 46
7.2. Overall Study Design 46

7.2.1. Immunization 47
7.2.1.1. Cohort 1 47
7.2.1.2. Cohort 2 48
7.2.1.3. Cohort 1 Hyperimmunity Sub-Cohort 48
7.2.2. Challenge Procedure 49
7.2.3. Leukapheresis Procedure Requirement 49
7.3. Immunoassays and Biological Samples 50
7.3.1. Immunoassays 50
7.3.1.1. Global Immune Response Assays 56
7.3.1.2. Humoral Response Assays 57
7.3.1.3. Cell Mediated Immunity (CMI) Response Assays 62
7.3.2. Systems Biology Approach 67
7.3.3. Antigen Discovery 67
7.3.3.1. CMI Immunoscreening: NMRC 68
7.3.3.2. Serum Antibody Screen: NMRC 68
7.3.3.3. *Pf* Proteome Array: SCRI 69
7.3.3.4. Antigen Lead Acquisition System (ATLAS™): Genocea 69
7.3.3.5. Malaria-Specific Immune Receptor Sequencing 69
7.4. Measures Taken to Minimize/Avoid Bias 70
7.5. Investigational Product 71
7.5.1. Investigational Product Preparation 71
7.5.1.1. Mosquito Production 71
7.5.1.2. Sporozoite Production and Grading 71
7.5.1.3. Irradiation of Sporozoites 72
7.5.1.4. Immunization Procedure 72
7.5.1.5. Controlled Human Malaria Infection (CHMI or Challenge) . 73
7.6. Duration of Subject Participation 73
7.7. Dose-adjustment Criteria 73
7.7.1. Stopping Rules 73
7.7.2. Study Termination Criteria 74
7.8. Trial Treatment Randomization Codes 74
7.9. Identification of Data to be Recorded on the Case Report Forms 74

8. Selection and Withdrawal of Subjects 75
8.1. Recruitment of Subjects 75
8.1.1. Referral Fee 75
8.2. Informed Consent Process 75
8.3. Eligibility Screening 76
8.4. Subject Inclusion/Exclusion Criteria 76
8.4.1. Subject Inclusion Criteria 76
8.4.2. Subject Exclusion Criteria 77
8.5. Subject Withdrawal Criteria 78
8.5.1. When and How to Withdraw Subjects 79
8.5.2. Data Collected for Withdrawn Subjects 79
8.5.3. Withdrawal of Active Duty Military Subjects 79
8.5.4. Replacement of Subjects 80
8.5.5. Follow-up for Withdrawn Subjects 80
8.5.6. Subjects Who Miss Immunization Doses 80

9. Study Procedures 81
9.1. Study Visit Schedule and Follow-up Periods 81
9.1.1. Screening Visit 81

9.1.2. Pre-Leukapheresis Visit 83
9.1.2.1. Hemoglobin 83
9.1.2.2. Platelets 84
9.1.3. Leukapheresis Visit 84
9.1.4. Immunization Visit 85
9.1.5. Follow-Up Visits Post-Immunization 85
9.1.6. Challenge Visits 86
9.1.6.1. Management of Subjects Post-Challenge: 87
9.1.6.2. Post-Challenge Follow-Up Visits 87
9.1.6.3. Overnight Stays (Day 7-18) 88
9.1.6.4. Days 20, 22, 25, and 28 Post-challenge 89
9.1.6.5. Treatment of Malaria for Parasitemic Subjects 89
9.1.6.6. Long-term Follow-up Visits 90
9.2. Unscheduled Visit 91
9.3. Concomitant Medications 92
9.4. Procedures for Monitoring Subject Compliance 92

10. Leukapheresis Procedure 93
10.1. Leukapheresis Procedure Risks and Common Side Effects 93
10.2. Apheresis Rationale 94

11. Safety Assessment 95
11.1. Safety Monitoring 95
11.1.1. Study Safety Management 95
11.1.2. Research Monitor 95
11.1.3. USAMMDA Clinical Services Support Division 95
11.2. Specification of Safety Endpoints 96
11.3. IND Safety Reporting 96
11.3.1. Adverse Event 96
11.3.2. Solicited Adverse Event 96
11.3.3. Serious Adverse Event 96
11.3.4. Unexpected Adverse Event 97
11.3.5. Other Adverse Event 97
11.4. Relationship to Investigational Product 97
11.5. Recording Adverse Events 98
11.5.1. Methods/Timing for Assessing, Recording, and Analyzing Safety Endpoints 98
11.5.1.1. Following *Pf*RAS Immunization 98
11.5.1.2. Following CHMI 98
11.5.2. Duration of Follow-Up of Subjects after Adverse Events 98
11.5.3. Severity Assessment 99
11.6. Reporting Adverse Events 99
11.6.1. Reporting Serious and Unexpected Adverse Events 100
11.6.1.1. Reporting to the Sponsor 100
11.6.1.2. Reporting to the IRB 102
11.6.1.3. Reporting to ORP HRPO 102
11.6.2. Reporting Additional Immediately Reportable Events to the Sponsor's Representative and ORP HRPO 102
11.6.2.1. Pregnancy 102
11.6.2.2. AE-related Withdrawal of Consent 103
11.6.2.3. Pending Inspections/Issuance of Reports 103
11.6.3. IND Annual Report to the FDA 103

11.6.4. Periodic Safety Reporting 103
11.6.5. Final Study Report 103

12. Statistics 104
12.1. Description of Statistical Method 104
12.1.1. Safety Analyses 104
12.1.2. Effectiveness Analysis 105
12.1.3. Laboratory Data Analysis 105
12.2. Planned Enrollment and Reason for Sample Size 105
12.3. Interim Analysis and Stopping Rules 105
12.4. Accounting for Missing, Unused, and Spurious Data 105
12.5. Procedures for Reporting Deviations from the Original Statistical Plan 105
12.6. Blinding of Research Subjects and Study Personnel 105
12.7. Selection of Subjects to be Included in Analyses 106

13. Direct Access to Source Data/Documents 107
13.1. Study Photographs 107
13.2. Study Monitoring 107
13.3. Audits and Inspections 107
13.4. Institutional Review Board (IRB) 107

14. Quality Control and Quality Assurance 109

15. Ethics 110
15.1. Ethics Review 110
15.1.1. Review/Approval of Study Protocol 110
15.1.2. Protocol Modifications 110
15.1.3. Protocol Deviation Procedures 111
15.2. Ethical Conduct of the Study 111
15.2.1. Confidentiality 111
15.2.2. Compensation for Participation 112
15.2.3. Medical Care for Research-Related Injuries 113
15.3. Written Informed Consent 114

16. Data Handling and Recordkeeping 115
16.1. Inspection of Records 115
16.2. Retention of Records 115

17. Publication Policy 117

18. References 118

19. Appendices 122

Appendix A. Informed Consent Documents 123

Appendix B. Personnel Roles and Responsibilities 124

Appendix C. Study Event Schedule 125

Appendix D. Toxicity Grading Scales 126

List of Tables

Table 1: Emergency Contact Information 9

Table 2: Abbreviations 21

Table 3: Clinical Experience with *Pf*RAS Immunization by Exposure to Infected Mosquitoes 29

Table 4: Summary of Immunization and Challenge Studies at NMRC/WRAIR: 1989-1999 32

Table 5: Summary of Re-challenge of Irradiated *Pf* Sporozoite Volunteers Following Primary Challenge and Booster Immunization 35

Table 6: Summary of Re-Challenge of Irradiated *Pf* Sporozoite Volunteers without Booster Immunizations 36

Table 7: Options for Cohort 2 Immunization Sessions Based on Level of Protection in Cohort 1 ... 48

Table 8: Sample Requirements for Immunoassays and Antigen Discovery 51

Table 9: Scoring for Dissected Glands for Immunization and Challenge Procedures 72

Table 10: Study Contacts for Reporting Serious Adverse Events 101

Table 11: SAE Information to be Reported to the Sponsor’s Safety Office 101

Table 12: Compensation Plan 112

Table 13: Toxicity Grading Scale for Solicited Local Adverse Events 126

Table 14: Toxicity Grading Scale for Vital Signs 128

Table 15: Toxicity Grading Scale for Systemic Adverse Events 129

Table 16: Laboratory Value Adverse Event Grading Scale 131

List of Figures

Figure 1: IFA Sporozoite Titers of Protected vs Unprotected Subjects 33

Figure 2: ELISA Antibody Titers to r*Pf*CSP of Protected vs Unprotected Subjects 33

Figure 3: Comparison of Mean Interval (in Days) from Last Immunization to Challenge for Individuals Immunized 34

Figure 4: Study Design 47

4. List of Abbreviations and Definitions of Terms

The following abbreviations are used in this study protocol.

Table 2: Abbreviations

Abbreviation	Explanation
AABB	American Association of Blood Banks
ACD-A	Acid Citrate Dextrose
ADi	Antigen Discovery, Inc.
AE	Adverse Event
ALP	Alkaline Phosphatase
ALT	Alanine aminotransferase
AR	Army Regulation
AST	Aspartate aminotransferase
An stephensi	Anopheles stephensi
β-HCG	Beta-Human chorionic gonadotropin
BMGF	Bill & Melinda Gates Foundation
BUN	Blood Urea Nitrogen
CBC	Complete Blood Count
CFR	Code of Federal Regulations
cGy	Centi-Gray
CHMI	Controlled Human Malaria Infection = malaria challenge
CMI	Cell Mediated Immunity
CRF	Case Report Form
CSI	Committee for Samples and Immunoassays
CSP	Circumsporozoite protein
CSSD	Clinical Services Support Division
CTC	Clinical Trials Center
CTL	Cytotoxic T lymphocyte
DoD	Department of Defence
DOT	Directly observed treatment
ECG	Electrocardiogram
EDC	Electronic Data Capture
ELISA	Enzyme-Linked Immunosorbent Assay
ELISpot	Enzyme-Linked Immunospot Assay
FACS	Fluorescence-Activated Cell Sorter
FDA	Food and Drug Administration
FWA	Federal-Wide Assurance
GAP	Genetically Attenuated Parasite
GCP	Good Clinical Practice
HBsAg	Hepatitis B surface antigen
HCV	Hepatitis C Virus

Abbreviation	Explanation
HIPAA	Health Insurance Portability Accountability Act
HIV	Human Immunodeficiency Virus
HLA	Human leukocyte antigen
HRPO	Human Research Protection Office
HSPB	Human Subjects Protection Branch
ICH	International Conference on Harmonisation
IFA(T)	Indirect fluorescent antibody (test)
IFN-γ	Interferon gamma
IL	Interleukin
ILSDA	Inhibition of Liver Stage Development Assay
Imm	Immunization
IMRAS	Immunization via mosquito bite with radiation-attenuated sporozoites
IND	Investigational New Drug
INR	International Normalized Ratio
IRB(s)	Institutional Review Board(s)
ISTI	Inhibition of Sporozoite Traversal and Invasion
ITT	Intention To Treat
ITV	Intervention-Treatment-Vaccination
μg	Microgram
mL	Milliliter
NIH	National Institutes of Health
NMRC	Naval Medical Research Center
ORP	Office for Research Protections
PBMC(s)	Peripheral Blood Mononuclear Cell(s)
PEVA(s)	pre-erythrocytic stage vaccine antigen(s)
Pf, P falciparum	*Plasmodium falciparum*
*Pf*RAS	*Pf* Radiation-Attenuated Sporozoites
PI	Principal Investigator
Pk, P knowlesi	*Plasmodium knowlesi*
Py, P yoelii	*Plasmodium yoelii*
PSSB	Product Safety Surveillance Branch
PT	Prothrombin Time
PTT	Partial Thromboplastin Time
QA	Quality Assurance
QC	Quality Control
qRT-PCR	Quantitative Real-Time Polymerase Chain Reaction
RNA	Ribonucleic acid
SAE	Serious Adverse Event
SOP	Standard Operating Procedure
SSP	Study Specific Procedure

Abbreviation	Explanation
SSP2	Sporozoite Surface Protein 2
TAC	Therapeutic Apheresis Center
TBD	To Be Determined
TBN	To Be Named
TSG-DA	The Surgeon General-Department of the Army
UPIRTSO	Unanticipated problems involving risk to subjects or others
USAMMDA	US Army Medical Materiel Development Activity
USAMRAA	US Army Medical Research Acquisition Activity
USAMRMC	US Army Medical Research & Materiel Command
USMMVP	US Military Malaria Vaccine Program
USUHS	Uniformed Services University of the Health Sciences
WBC	White blood cell
WRAIR	Walter Reed Army Institute of Research
WRNMMC	Walter Reed National Military Medical Center

5. Introduction

5.1. Need for a Malaria Vaccine

Malaria represents a major public health problem worldwide, causing significant morbidity and mortality throughout endemic areas. The World Health Organization has reported that malaria was responsible for approximately 216 million clinical cases annually and 655,000 deaths in 2010, mostly in children living in sub-Saharan Africa (WHO-2011). Recent analyses suggest that the medical impact of malaria may be much higher than these figures would suggest (Murray et al-2012). The burden of malaria also extends to international tourists and to individuals residing in malaria-free regions of malaria-endemic countries who travel locally to areas where malaria is transmitted; these individuals are particularly at risk of developing severe disease, due to immunologically naïve status. Malaria also hinders economic development, exacting a toll on growth and productivity (Breman et al-2004; Gallup and Sachs-2001). Widespread and increasing drug and insecticide resistance, by the parasite and vector, respectively, highlight the importance of developing an effective malaria vaccine (Hill et al-2010).

5.2. Military Relevance

Malaria has had a significant impact on US military operations throughout our history. It was responsible for a greater loss of manpower than enemy fire in all conflicts occurring in tropical regions during the 20th century (Beadle and Hoffman-1993). Malaria continues to present a major challenge to force health protection during operations in any environment where malaria is endemic. This includes 108 countries spanning the tropical and subtropical regions of the world, including most of sub Saharan Africa and large regions of South Asia, Southeast Asia, Oceania, Central Asia, the Middle East, Central and South America, and the Caribbean. The US military is either currently deployed or has the potential to deploy on short notice to any of these regions, making malaria a leading infectious threat to mission success. In our malaria-naïve military population, an infection with malaria can severely degrade performance, result in missed duty, and may lead to prolonged hospitalization and, in some cases, death. The measures used to avoid malaria frequently compromise military performance and, given the difficulties of implementing control measures and chemoprophylaxis in combat, cannot be completely relied upon to prevent infection. Recent events in a number of endemic locations including Liberia in 2003 and 2009-10, Benin in 2009, and Haiti in 2010 underscore the DoD's critical need for a malaria vaccine for deployed military personnel (Armed Forces Health Surveillance Center-2010a, b; Centers for Disease Control Prevention-2010; Whitman et al-2010). Of the 5 malaria species infecting humans, *Plasmodium falciparum* has been prioritized by the US military for vaccine development because of its greater severity, with vaccines against *P vivax* a close second in priority.

5.3. Rationale for Study

In the early 1970s, several investigators reported that radiation-attenuated *P falciparum* sporozoites (*Pf*RAS), when delivered in sufficient numbers via mosquito bite to malaria-naïve human subjects, conferred sterile protection against malaria infection by inducing an immune response targeting the pre-erythrocytic stages of the parasite. Despite nearly 40 years of study, however, neither the immunological mechanism nor the antigens targeted have been precisely identified, although there is evidence from animal models that antibodies, CD4+ T cells and

especially CD8+ T cells contribute to radiation-attenuated sporozoite (RAS)-induced immunity. If a better understanding of the protection afforded by *Pf*RAS could be gained, it would strongly support the development of malaria vaccines, especially the identification of vaccine platforms inducing protective responses mimicking *Pf*RAS and the design of subunit vaccines incorporating protective antigens derived from the sporozoite and liver stages of the parasite that are targeted by *Pf*RAS-induced immunity.

The clinical trial described in this protocol aims to address this goal. Study subjects in this study, called "IMRAS" (immunization via mosquito bite with radiation-attenuated sporozoites), will be immunized with *Pf*RAS by receiving approximately 960 bites from infected, irradiated mosquitoes over several immunization sessions. This trial constitutes the cornerstone of a larger collaborative investigation to better characterize the protective mechanisms associated with several models of whole sporozoite immunization, including *Pf*RAS (this trial), infection-treatment vaccination or ITV (also called CPS, or chemoprophylaxis with sporozoites) (Roestenberg et al-2009; Roestenberg et al-2011), and immunization with genetically attenuated parasites (GAP) (Butler et al-2012; Matuschewski et al-2011). In this project advanced molecular tools will be used to identify the biological processes underlying whole sporozoite immunization, focusing on both innate and acquired immune responses, in a manner similar to the methods used in the related ITV and GAP projects.

Our hypothesis is that the best approach to understanding the protection induced by *Pf*RAS is to characterize, in the broadest and most unbiased manner possible, the complete host response, using the tools of systems biology. Starting with innate responses, we will assess the transcriptional profiles associated with early phases of *Pf*RAS immunization by collecting PBMCs from immunized subjects in kinetic studies and characterizing their evolving genomic signatures with software applications that will permit the association of response patterns with protection. We will likewise profile the full complement of serum and PBMC micro-RNAs, reasoning that the ability of these small molecules to critically regulate the innate and adaptive immune systems via their effects on mRNA will control the outcome of vaccine-induced responses. As another way to approach the immune response, we will assay soluble factors in plasma including 37 cytokines and chemokines using a variety of multiplexed assays. We will also examine immune cell phenotypes and functions, including the memory, maturation, and activation status of T and B lymphocytes and dendritic cells using multiparameter flow cytometry.

Complementing this systems biology approach, we will stimulate PBMCs with purified sporozoites, sporozoite extracts, and peptides derived from several representative pre-erythrocytic stage vaccine antigens (PEVAs). Immune responses to these antigens will also be studied without stimulation using tetramers and pentamers binding human leukocyte antigen (HLA)-matched epitopes for a subset of subjects, permitting cell sorting to enrich epitope-specific lymphocyte populations that can be characterized for activation markers and master regulators potentially correlating with protection. We will also study follicular helper T cells and antibody-secreting plasma cells due to the likely involvement of antibody in RAS-induced protection and test serum for its ability to inhibit sporozoite invasion and development in hepatocytes, as well as to promote parasite destruction through a cytotoxic T lymphocyte (CTL) killing assay. All these data will be integrated using powerful software applications, many of which have been developed by our collaborators, so that associations can be drawn with immunization, challenge, and protection.

To address these objectives, we will immunize two cohorts of 12 malaria-naïve subjects with *Pf*RAS via mosquito bites and sample their cells and sera at appropriate timepoints prior to, during, and after immunization. Subjects will undergo controlled human malaria infection (CHMI), also called malaria challenge, following immunization to ascertain their degree of protection. In order to maximize the power of the study to identify the correlates of protection, we have selected an immunization regimen for Cohort 1 that, based on prior clinical data, should protect about 50% of the study subjects. Because prior clinical data are limited, however, there is the possibility that regimen used for Cohort 1 will not achieve 50% protection. Cohort 2 will be immunized after Cohort 1 to allow an adjustment of the immunization regimen for Cohort 2 in the event that protection results from the first cohort are skewed (either too many or too few study subjects protected).

To measure the host response, sera and PBMCs will be collected at various timepoints to enable immunomonitoring. Most cells will be processed and cryopreserved for later use in the assays described, while a portion will be reserved for immediate use in fresh assays. To collect sufficient PBMCs, particularly for use in antigen discovery, subjects will be leukapheresed at key timepoints. To control for the effects of mosquito salivary gland antigens injected along with sporozoites during the mosquito feed immunizations, we will include 4 mock-immunized controls per Cohort, to be bitten by irradiated, uninfected mosquitoes and challenged in parallel with the true-immunized subjects and the infectivity controls. Comparing the immunological profiles of true- and mock-immunized subjects will help differentiate the effects of sporozoites and mosquito salivary gland antigens.

Limited prior data from clinical studies suggest that the high-grade protection induced by *Pf*RAS immunization as described above may be transient, and that it is not until after *Pf*RAS-immunized subjects have undergone primary malaria challenge that high-grade, durable immunity is generated. In order to study this “second tier” of immunity, the IMRAS trial includes an optional continuation phase for subjects in Cohort 1 who are protected upon the primary challenge. As a first data point for “second tier” immunity, immunological profiling (using all the assays employed during primary immunization and challenge) will be conducted following primary challenge of Cohort 1 for comparison with pre-challenge samples.

Those subjects who are protected on primary challenge and opt to participate in the continuation phase (up to a maximum of 6 subjects) will then be hyperimmunized by receiving 3 secondary (boosting) immunizations with *Pf*RAS via mosquito bite. These boosts will occur in conjunction with the first 3 primary immunizations of Cohort 2. These hyperimmunized individuals will be re-challenged at the time that Cohort 2 volunteers receive primary challenge. PBMCs and sera will be collected during and after boosting for ongoing immunological profiling, documenting the further development of “second tier” immunity. It is expected that all of the hyperimmunized study subjects will be protected on re-challenge. However, if any subjects are not protected, this information will aid in identifying the protective components of evolving anti-*Pf*RAS immunity.

Serum samples collected periodically from continuation phase volunteers will be used in passive immunization studies in humanized mice challenged with *P falciparum* to look for serum-transferrable protection. This objective is based on studies in murine models showing that hyperimmunization, in this case induced by repeated challenge, leads to serum-transferrable protection not present following primary challenge (Schmidt et al-2010). This serum-transferrable protection builds on the CD8+ T cell-based protection induced by primary

immunization. Limited data suggest that these antibodies may supplement cell-mediated anti-liver-stage immunity by targeting malaria antigens expressed on the surface of infected hepatocytes. For example, Rénia et al (1990) showed in a murine model that antibody to *Pf* heat-shock-like protein could lyse *Py*-infected liver nonparenchymal cells through an antibody-dependent cellular cytotoxicity mechanism.

Strengthening antibody responses to liver-stage antigens developing during hyperimmunization will be further examined by the periodic collection of plasmablasts for sequencing and bio-informatics analysis. Immunoglobulin repertoire profiles derived from the plasmablasts will be generated to define evolving clonal relationships as rising titers and affinity maturation occur following repeated immunizations and challenges. Transfection of paired heavy and light-chain immunoglobulin genes will be used to generate monoclonal antibodies for characterizing functional activity and for supporting the identification of target antigens.

Hyperimmunization of protected Cohort 1 study subjects should also improve the strength of T cell responses to diverse antigenic components of *Pf*RAS, increasing the sensitivity of cellular immunological screening for antigen discovery.

By request of the IMRAS project's funder, the Bill & Melinda Gates Foundation (BMGF), a special committee has been established called the Committee for Samples and Immunoassays (CSI) which was chartered to "optimize the collection, storage, distribution, testing and analysis of biological samples taken from the research subjects in the IMRAS trial, to enhance the likelihood of identifying biomarkers of protection and associated target antigens, thereby maximizing scientific benefit" (from CSI Terms of Reference). The CSI is composed of investigators from NMRC, BMGF, SCRI, WRAIR, and also outside experts from PATH-MVI. The CSI has been instrumental in reviewing planned immunological assays and in providing recommendations. This protocol reflects the outcome of these discussions. Of note, the BMGF has requested that 25% of the PBMCs be reserved for future use to be determined by the BMGF.

As an additional objective of IMRAS, if sufficient funding is provided by the BMGF, we will conduct limited immunoscreening of existing panels of candidate PEVA using the PBMCs from the clinical trial. The potential value of candidate antigens will be measured by the intensity of the interferon-gamma (IFN-γ) responses they induce in *Pf*RAS-immunized and protected subjects, by the number of protected subjects showing immunorecognition of the antigen, and by more frequent recognition by protected than non-protected subjects.

Immunization with *Pf*RAS has been previously done by NMRC and WRAIR, as detailed in Hoffman et al (Hoffman et al-2002). In addition to these published data, 10 additional subjects were immunized in 2000-2002, and the PBMCs collected from these subjects have been used successfully for antigen discovery (Doolan and Hoffman-1997, 2000). The supply of PBMCs from these 10 subjects is now depleted; however, interest in the human *Pf*RAS model as a way to support antigen discovery has grown. The proposed study will provide additional samples from *Pf*RAS-immunized subjects for this purpose.

5.4. Summary

In summary, we will immunize human subjects with *Pf*RAS, extensively characterize their responses, and correlate their responses with protection. This will enable the identification of protective immunological signatures and a prioritized list of candidate antigens involved in

*Pf*RAS-induced immunity. The biosystem signatures will be used to create validated biomarkers to use as immune correlates or surrogates. The information gained from this study should accelerate malaria vaccine development, since both the nature of pre-erythrocytic stage protective immunity as well as many of the targeted antigens will have been characterized. Biomarkers of protection will not only benefit vaccine development but also serve as indicators of vaccine take and thus support the deployment of vaccines in the field.

This project will rely upon the expertise of the NMRC and the WRAIR in *Pf*RAS immunization and malaria subunit vaccine development, the expertise of SCRI in systems biology, and the expertise of all 3 institutions in immunology, parasitology, antigen discovery, and whole sporozoite vaccines. The IMRAS clinical trial will be performed by NMRC, WRAIR, and USAMMDA (the latter acting as the sponsor). The trial will take place over a 3-year time period, with preparation for the clinical trial and subject recruitment during the first year; immunization, challenge, and follow-up during the second year and first part of the third year; and analyses of samples during the third year. Depending upon the success of the clinical trial, and the availability of funding from the BMGF, a protocol addendum may be submitted that would provide for additional follow-up boosting and re-challenge of research subjects during the third year.

5.5. Name and Description of the Investigational Product

This trial will be performed under a US Food and Drug Administration (FDA) Investigational New Drug (IND) application. The investigational product is radiation-attenuated *P falciparum* sporozoites administered by the bite of infected *An stephensi* mosquitoes. Immunizations and malaria sporozoite challenge will be performed with the NF54 *P falciparum* strains (or 3D7 strains if necessary). Mosquitoes used to immunize, whether infected with malaria or uninfected, will be irradiated with 15,000 cGy just prior to immunizations. This vaccine procedure has been studied before, but is now being performed under FDA regulatory oversight.

Refer to section7.5 for additional information.

5.6. Summary of Nonclinical and Clinical Trials

5.6.1. Nonclinical Studies with the Radiation Attenuated Sporozoite Vaccine

Malaria vaccine candidates have been extensively investigated over the past 4 decades with the first report in 1967 that mice immunized with irradiated sporozoites were protected against challenge with infectious sporozoites (Nussenzweig et al-1967). Since then, several studies have established that immunization with irradiated *P berghei*, *P chabaudi*, or *P yoelii* sporozoites protects against sporozoite challenge in rats and mice (Doolan and Hoffman-1997, 2000; Hoffman et al-1989; Nussenzweig et al-1967; Schofield et al-1987; Seguin et al-1994; Weiss et al-1988). Similarly, immunization with irradiated *P cynomolgi*, *P knowlesi*, and *P vivax* sporozoites protects or partially protects non-human primates (Collins and Contacos-1972; Gwadz et al-1979).

5.6.2. Clinical Studies

5.6.2.1. Safety Summary

Table 3 presents an overview of the published clinical studies along with the data from the most recent clinical trial conducted at NMRC during 2000-2004 (unpublished; DoD 30598). Several subjects participated in more than one of these studies and were challenged and re-challenged at various timepoints following the vaccine series of different durations.

Table 3: Clinical Experience with *Pf*RAS Immunization by Exposure to Infected Mosquitoes

Time frame (References)	Range of Radiation Dose (cGy)	Number of Subjects	Range of Immunizations per Subject	Range of Bites[a] per Subject	Breakthrough Infection[b] Infected/Total
1970s (Clyde-1990)	12,000	7	6–37	379–2206	4/7
1970s (Rieckmann-1990)	12,000	11	2-8	200–987	2/11
1980s through early 1990s (Edelman et al-1993; Herrington et al-1991)	17,000–27,000	5	7–20	625–1894	0/5
1989 through 1999 (Egan et al-1993; Hoffman et al-2002)	15,000	13	6–21	606–2290	0/13
2000-2004 (NMRC DoD 30598, unpublished)	15,000	27	5-6	1005-1561	0/27

[a] Cumulative number of mosquito-bites administered before a challenge experiment.
[b] Blood-stage *Pf* infection following vaccination, due to insufficient attenuation of the *Pf* parasite.

5.6.2.1.1. Breakthrough Blood-Stage *Pf* Infections

The *Pf*RAS immunization procedure has generally been well tolerated. A safety concern is that there could be breakthrough *P falciparum* infections in the immunized subjects, due to inadequate attenuation of the parasites. However, in our experience and in the world's literature, there has never been a breakthrough *P falciparum* infection with a 15,000 cGy irradiation dose (Clyde et al-1973a; Clyde et al-1973b; Rieckmann et al-1974; Rieckmann et al-1979). Also, a parenterally administered formulation of radiation-attenuated *P falciparum* sporozoites, called the *Pf*SPZ Vaccine, also subjected to 15,000 cGy, has been administered to 80 subjects by needle and syringe subcutaneously or intradermally (Epstein et al -2011) and to another 41 subjects intravenously; none of these subjects has experienced a breakthrough infection (unpublished data). Breakthrough infections can occur with lower radiation doses, however. In the studies by Clyde (1990), 4 of 7 subjects immunized by sporozoites irradiated with 12,000 cGy developed breakthrough infections. In the studies by Rieckmann (1990), 2 of 11 subjects immunized by sporozoites irradiated with 12,000 cGy developed breakthrough infections. For this reason, strict calibration and monitoring of the primary and back-up irradiators will be important components of the IMRAS trial.

5.6.2.1.2. Local Adverse Events

Subjects have generally noted mild discomfort and mild to moderate local reactogenicity similar to that experienced after being exposed to nonirradiated mosquitoes in the wild. After immunization, most subjects develop erythematous papules with surrounding erythema at the bite site, with or without local swelling, which coalesced into a confluent erythematous plaque that resolved within 24 to 72 hours. In most cases, these reactions have been limited to the area of skin to which the canister of feeding mosquitoes was applied. Two subjects, however, experienced more extensive reactogenicity at the site of the mosquito bites, developing edematous forearms that were termed “large local reactions”. The swelling did not extend above the elbow or below the wrist. One of these was experienced by a true-immunized subject, and one by a mock-immunized subject, indicating that the cause was likely the mosquito salivary gland antigens and not the malaria sporozoites. These reactions resolved uneventfully over 1 or 2 days without sequelae, and further immunizations of these 2 subjects were halted. One of these subjects (mock-immunized) had, during 2 previous immunization sessions, experienced a tracking erythematous streak likely corresponding to a lymph vessel extending from the immunization site up the arm to the neck, potentially heralding the large local reaction occurring on the third immunization.

5.6.2.1.3. Systemic Adverse Events

Systemic adverse events following immunization via mosquito bites have generally been mild. The earlier studies do not comment upon occurrence of systemic adverse events associated with immunization itself. Of a total of 27 subjects immunized at NMRC and WRAIR, 7 were noted to have mild malaise and headache during the 24 hours after immunization. In addition, in July of 2002, 2 subjects at NMRC had the onset of systemic symptoms approximately 16 hours after immunization.

The first subject experienced the onset of fever to 103°F, chills, sweats, mild neck ache, fatigue, and nausea, which improved within 2 hours and resolved uneventfully over the following 24 hours. All laboratory studies in this subject, including chemistries (eg, potassium, blood urea nitrogen (BUN), liver and renal function tests), and urinalysis were essentially within normal limits. Although the subject was able to go to work the morning after the immunization (6 hours after onset of symptoms), the AEs were classified as “severe” since at the time they occurred, the subject would have been unable to work.

The second subject experienced low-grade fever (99.8°F), malaise, myalgia, and nausea, all of which were classified as “mild” and resolved within 24 hours. Work-up by an allergist at the Walter Reed Army Medical Center, in consultation with allergists at National Institute of Allergy and Infectious Diseases and the National Naval Medical Center, indicated that the most likely diagnosis was a form of serum sickness, resulting from a concentration of pre-formed antibody and a concentration of foreign antigen (presumably mosquito salivary gland antigens) appropriate for the precipitation of antigen-antibody complexes in the blood. A review of preparation of the mosquitoes used for immunization did not reveal bacterial contaminants that might have been a source of bacteremia, which was considered unlikely due to the timing and rapid, spontaneous resolution of symptoms.

One of these 2 subjects with significant systemic reactions subsequently underwent further immunizations and challenge and did not re-experience systemic adverse events, nor did any of the other subjects from the study that subsequently underwent challenge.

Per the recommendation of CBER, a confirmed diagnosis of serum sickness and/or a delayed Type IV hypersensitivity reaction has been included as a stopping rule. Additionally, an IMRAS SSP has been developed to aid in the recognition, diagnosis, and treatment of any suspected cases of serum sickness. Moreover, COL Michael Nelson, Clinical Immunologist and Deputy Commander for Education, Training, and Research at WRNMMC, has agreed to act as a consultant if the need should arise.

5.6.2.1.4. Clinical Laboratory Data

The study charts for 22 subjects enrolled in DoD 30598 were available to document post-immunization laboratory abnormalities. The protocol design did not include systematic collection of post-immunization safety laboratory tests for research purposes; however, occasional clinical laboratory tests were collected during the study in accordance with the clinical judgment of the investigators to monitor safety on a case-by-case basis, and no significant laboratory abnormalities were identified.

5.6.2.2. Effectiveness Summary

The first human studies were reported in 1973 by Clyde and colleagues at the University of Maryland (Clyde-1975, 1990; Clyde et al-1973a, 1973b) and Rieckmann, Beaudoin, and colleagues at NMRC (1974, 1979). Mosquitoes with high-grade sporozoite infections were exposed to 12,000 to 27,000 cGy of irradiation and allowed to feed on malaria-naïve subjects. After several immunizations over the course of several months, accumulating a total of 200 to over 1,000 infected bites, subjects were challenged with non-irradiated mosquitoes harboring infectious sporozoites. Subjects immunized with greater than 950 bites from irradiated mosquitoes harboring either *P falciparum* or *P vivax* sporozoites were consistently protected against homologous challenge with non-irradiated sporozoites.

During the period of 1989-1999 investigators at NMRC, WRAIR, and the University of Maryland built on the foundation provided by Clyde, Rieckmann, and colleagues to further characterize sporozoite-induced protective immunity in humans. The studies conducted during this time period demonstrated that subjects who received $\geq$ 1,000 immunizing bites were protected, with all primary challenges of these subjects performed within 9 weeks of the last immunization session. The results of the study conducted by NMRC/WRAIR "Immunization of human subjects with *Plasmodium* sp. sporozoites" (1989-1994: HURRAO Log A-4839/WRAIR 229; 1995-1999 DoD 30518/WRAIR 363) are shown in Table 4.

In this study, several subjects were re-challenged at varying intervals and maintained protection. A total of 13 subjects were studied, 12 of whom were challenged (Table 4). The *P falciparum* NF54 strain and 3D7 clone of NF54 were used for immunization. All subjects receiving > 1,000 immunizing bites were protected against challenge with 2 exceptions: subject 15, who was immunized with 1,008 infectious bites and challenged 10 weeks post-immunization, and subject 4, who was protected in 5 post-immunization challenges but had lost immunity when challenged a sixth time at 5 years post-immunization. Two of the total 19 instances of protection (12 subjects challenged, some of whom received more than one post-immunization challenge)

were with heterologous strains, confirming earlier results. Of note, protection was demonstrated in 4 of these subjects despite long intervals between immunization and challenge (14, 14, 23, 36, 36, 41, and 42 weeks) (Hoffman et al-2002). In these cases, each study subject had received a primary challenge soon after immunization. Intervals longer than 10 weeks between immunization and primary challenge have not been studied.

Overall these studies demonstrated that immunization with *Pf*RAS produced potent, species-specific and strain-transcending protective immunity in humans against CHMI provided that:

1. Subjects received at least 1,000 immunizing bites.
2. Radiation dose ranged from 15,000 to 20,000 cGy.
3. Primary challenge occurred within 3 weeks and no later than 9 weeks.

Protection, measured by secondary challenge in subjects already undergoing primary challenge, appeared to be sustained, persisting as long as 42 weeks.

Table 4: Summary of Immunization and Challenge Studies at NMRC/WRAIR: 1989-1999

Subject ID	Study Period	Number of Immunization Sessions	Total Number of Immunizing Bites Prior to Last Challenge	Number of Challenges Per Subject	Protected Status Following Challenge/Total Challenges
1	6/89-5/90	8	606	1	0/1[a]
3	6/89-5/90	9	1,007	1	1/1
4	10/89-8/98	16	2,211	7	6/7[b]
5	10/89-8/98	21	2,927	5	5/5
10	11/90-5/94	16	1,872	3	3/3[c]
11	9/90-2/95	11	1,214	2	2/2
12	11/90-1/93	11	1,130	2	1/2[d]
15	2/93-2/95	10	1,008	1	0/1[e]
16	2/93-7/98	17	2,290	2	2/2
17	2/93-6/95	8	1,163	1	1/1
18	10/93-3/98	8	1,043	1	1/1
19	12/97-3/98	5	1,050	1	1/1
n = 12		n = 140		n = 27	

[a] Subject 1 was not protected when challenged after immunization with an accumulated total of 606 infectious bites.
[b] Subject 4 was not protected on fifth challenge which occurred almost 5 years after the 12th immunization, but was protected when re-challenged 2 weeks after a subsequent boost.
[c] Subject 10 was protected on second challenge at 36 weeks without boosting immunization.
[d] Subject 12 was not protected on second challenge at 36 weeks without boosting immunization.
[e] Subject 15 was not protected with a primary challenge at 10 weeks.

The most recent experience at NMRC is with protocol DoD 30598, 2000-2004, "Immunization of human subjects with *Plasmodium* sp. sporozoites". During this latest study (2000-2002), 10 subjects completed a course of 5-6 immunizations over 6-8 months receiving a total of 1,005-

1,561 (mean 1,245) irradiated infected mosquito bites and underwent sporozoite challenge 2-6 weeks later, along with 6 infectivity controls. In contrast to prior experience, only 5 out of the 10 were protected on initial challenge.

Although there was no sterile protection in 5 of the 10 immunized subjects, time to parasitemia was delayed in those subjects as compared to infectivity controls (13 days vs 10 days, p = 0.03) implying that there was a degree of protection, as manifested by decrease in liver-stage burden and subsequent release of fewer parasites into the blood, delaying the onset of parasitemia in the immunized group. The number of immunizing bites was similar in protected and unprotected irradiated sporozoite subjects (1,281 vs 1,208 bites), and overall there were no significant differences between protected and unprotected subjects when comparing other variables, although time between final immunization and challenge may have been a factor. Subjects who were protected demonstrated significantly increased peak indirect fluorescent antibody (IFA) titers and had higher Enzyme-Linked Immunosorbent Assay (ELISA) antibody titers against recombinant *Pf* circumsporozoite protein (r*Pf*CSP) compared to the unprotected subjects (Figure 1 and Figure 2).

Figure 1: IFA Sporozoite Titers of Protected vs Unprotected Subjects

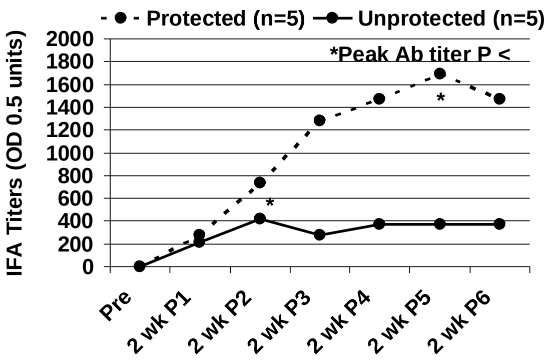

Figure 2: ELISA Antibody Titers to r*Pf*CSP of Protected vs Unprotected Subjects

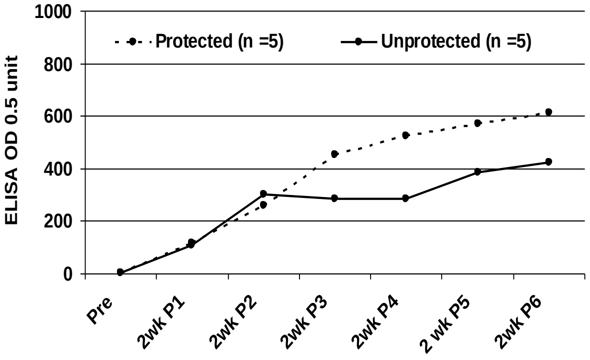

The results of this recent experience (5/10 protected) appear to differ from results obtained over the preceding 3 decades. Besides the possibility that these are true findings and that the protection provided by irradiated sporozoites really does vary between 50% and 90%, other hypotheses to explain these results have been developed:

1. Preliminary analysis suggests that the longer interval prior to CHMI may affect protection (Figure 3). It appears that subjects challenged > 30 days after last immunization were less likely to be protected.

2. The subjects in the 2000-2002 NMRC trial had leukapheresis prior to first immunization and challenge. It is possible that the leukapheresis procedure may have impacted the presence of immune cells in some individuals that may have been critical in the immune response to sporozoite challenge.

Of note, all parasitemic subjects experienced expected symptoms of malaria, received chloroquine as therapy, and had a rapid and uncomplicated recovery. One unprotected subject was re-immunized with 6 more immunizations (≥ 1,000 additional immunizing bites) and was protected on re-challenge with a homologous strain.

Figure 3: Comparison of Mean Interval (in Days) from Last Immunization to Challenge for Individuals Immunized

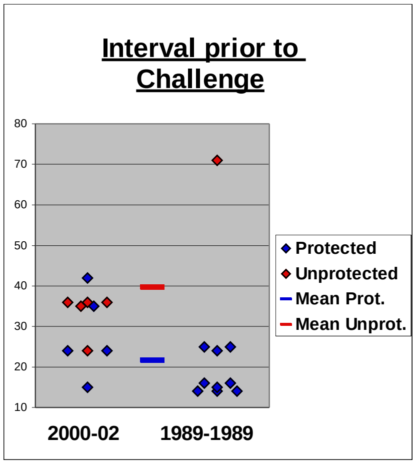

Means for ALL (1989-99 & 2000-02 combined) protected and unprotected shown in blue and red bars in center of graph.

5.6.2.3. Two-tiered Immunity: Effect of Primary Challenge and Booster Immunizations

Mouse models indicate that challenge of mice immunized by a variety of methods including RAS strengthens both humoral and cell mediated responses contributing to long-term sterile immunity. This "second tier" of immunity is superior in terms of both potency and duration to the immunity induced by primary immunization. In humans, there is evidence that booster immunizations following challenge may contribute to "second tier" immunity just as much as primary challenge. In prior human clinical studies, the majority of the re-challenges were preceded by one or more booster immunizations of *Pf*RAS as well as by the earlier primary challenge (Table 5), making it difficult to separate the effects of primary challenge and booster immunizations on long-term protection. Eight individuals underwent 16 re-challenges with between one and ten intervening boosts. Only one of these re-challenges resulted in patent parasitemia, and this individual was challenged with 90 infected mosquitoes, which is an unrealistic challenge that may have little relevance to this vaccine model. The 15 successful re-

challenges (meaning subjects were protected) occurred 1–39 weeks after the last immunization, underscoring the long-term nature of the immunity generated in immunized/challenged/boosted subjects. However, these data do not clearly distinguish whether it is primary challenge, secondary (booster) immunizations, or both that generate long-term, robust immunity.

Table 5: Summary of Re-challenge of Irradiated *Pf* Sporozoite Volunteers Following Primary Challenge and Booster Immunization

Subject ID	Reference	Cumulative No. Since Prior Challenge		Weeks Between Last Immunization and Challenge	Protection Status Following Challenge
		Immunizations	Immunizing bites		
4	Hoffman et al-2002	2	264	2	Protected
		2	291	9	Protected
		1	147	2	Protected
		3	463	6	Protected
5	Hoffman et al-2002	2	296	2	Protected
		2	288	8	Protected
		9	1342	6	Protected
10	Hoffman et al-2002	6	782	7	Protected
11	Hoffman et al-2002	1	121	23	Protected
16	Hoffman et al-2002	8	1163	5	Protected
GZ	Clyde et al-1973b	5	810	2	Protected
		1	120	1	Protected
		1	132	4	Protected
DFC	Clyde et al-1975	10	968	1	Protected
		3	400	12	Not protected[a]
8	Edelman et al-1993	1	199	39	Protected

[a] Volunteer was challenged with the bites of 90 infected mosquitoes.

5.6.2.3.1. Effect of Primary Challenge in the Absence of Booster Immunizations:

There are several volunteers who were re-challenged without intervening booster immunizations. A summary of these re-challenges, which occurred without intervening booster immunization, is described in Table 6. Eight individuals underwent 11 re-challenges 2–216 weeks after a previous challenge, of which 5 were protected with intervals of 2, 6, 32, 32, and 36 weeks. The other 3 subjects were not protected. It is thus not clear if repeated challenges have equivalent power to boost immunity as primary challenge followed by secondary (booster) immunizations. For example, there was 1 volunteer who underwent 3 re-challenges without any additional booster immunizations and was not protected for the last 2 challenges, which were performed after 9- and 8-week intervals.

Table 6: Summary of Re-Challenge of Irradiated *Pf* Sporozoite Volunteers without Booster Immunizations

Subject ID	Reference	Weeks Between Prior Challenge and Re-challenge	Protection Status Following Re-challenge
4	Hoffman et al-2002	32	Protected
		216	Not protected
5	Hoffman et al-2002	32	Protected
10	Hoffman et al-2002	36	Protected
12	Hoffman et al-2002	36	Not protected
GZ	Clyde et al-1973	2	Protected
LA	Rieckmann et al-1979	14	Not protected[a]
DS	Rieckmann et al-1979	6	Protected
		9	Not protected
		8	Not protected
WD	Rieckmann et al-1979	10	Not protected

[a] Volunteer was challenged with the bites of 45 infected mosquitoes.

In conclusion, data from humans suggest that secondary immunization (boosting) with *Pf*RAS is as important and potentially superior to re-challenge for inducing and maintaining long-term immunity in humans.

5.7. Known and Potential Risks and Benefits to Human Subjects

5.7.1. Risks/Discomfort to Subjects and Precautions to Minimize Risk

Described in the sections that follow are anticipated adverse reactions as well as potential but unlikely adverse reactions and a brief description of procedures to ameliorate risks and symptoms. All known risks and precautions described here are explained in detail in the informed consent (Appendix A).

5.7.1.1. Risks Associated with Immunization by Mosquito Bites

Subjects in the immunization groups (true and mock-immunization) will be immunized via the bites of irradiated mosquitoes. For each immunization session, the subject may receive 200-400 mosquito bites total. The risks associated with getting immunizing mosquito bites include the following:

- Local reactions. Local reactions including swelling, erythema, induration, and/or pruritus limited to the site where the mosquito-feeding container was placed, which can last 3-4 days. This occurs in the majority of subjects.
- Larger local allergic reaction after repeated exposures to mosquito bites. In the past, 2 out of 46 subjects have experienced large local reactions on the arm that received the mosquito bites (general swelling of the forearm extending beyond the margins of the container enclosing the mosquitoes), but these resolved with no issue. These 2 subjects were withdrawn from the study but have not subsequently had any reactions like this to mosquito bites from the wild.

- Systemic reactions. Although no immediate systemic allergic reactions have ever occurred, more delayed systemic reactions have occurred. In 2 instances, approximately 16 hours followed immunization, subjects experienced chills, fever, sweats, fatigue, muscle ache, and headache, which resolved completely within 24 hours.
- Blood-stage infection. If the irradiation is not complete, the subject could theoretically develop a blood-stage infection from the immunization process due to inadequate attenuation of the sporozoites. However, in over 10 years of conducting this procedure, this has not occurred with a 15,000 cGy irradiation dose. If the subject were to get malaria, he/she would be treated with 1 of the malaria drugs/regimens discussed in section 5.7.1.3.

5.7.1.2. Risks Associated with CHMI

Subjects (immunized and mock immunized) will be challenged with malaria in this study to assess the protective efficacy of the immunizations. Additionally, nonimmunized infectivity control subjects will be challenged. Therefore, some of the subjects are expected to develop malaria and to experience normal symptoms of malaria including the following:

- Fever, shaking chills, headache, dizziness, fatigue, muscle aches
- Nausea, vomiting, stomach cramps, diarrhea
- Mild anemia and moderate decrease in leukocytes and platelets

The clinical outcomes of subjects post-challenge are discussed in detail in the published literature (Epstein et al-2007; Roestenberg et al-2012).

Unfortunately, in malaria-endemic areas, the population is at risk for naturally acquired malaria. In cases where the diagnosis and treatment of malaria are delayed resulting in high levels of parasitemia, the most severe complications may develop. In that type of uncontrolled circumstance, malaria infection can lead to kidney, liver, or brain injury (seizures, coma) and death. Under the carefully controlled conditions of this study, the chance of such complications is unlikely and the risk of death from malaria infection is very small. There have been no cases with complications resulting in severe disability or death in study subjects undergoing controlled human malaria infection. Furthermore, no hospitalization has been required in past studies (for treatment due to failure to respond to an anti-malaria drug or for any other reason related to the study). Subjects are monitored closely and treatment is initiated immediately upon identification of parasitemia.

Of note, 3 cardiac events have been reported following CHMI in clinical trials in the Netherlands:

> In 2002, a 39-year-old male subject experienced a myocardial infarction during malaria treatment by chloroquine. The myocardial infarction occurred 2 days following detection of malaria parasites in a blood smear. The subject had cardiovascular risk factors (hypercholesterolemia and smoking) and the angiography revealed a stenosis of one of the coronary arteries (Verhage et al-2005). In retrospect, the diagnosis of parasitemia was debatable. Further, RT-PCR analysis of samples collected at the time of the cardiac event

were negative for malaria (the PCR analysis was conducted several months following the cardiac event).

In 2008, a 20-year-old healthy female subject developed retrosternal chest pain 2 days after treatment with artemether/lumefantrine for parasitemia. A diagnosis of acute coronary syndrome with limited myocardial necrosis of the inferior wall was based upon the pain, < 1 mm ST segment elevation ECG findings and cardiac enzyme analysis. A cardiac MRI was negative for evidence of atherosclerotic disease. The subject recovered quickly and her follow-up was uneventful. It is unclear if this subject's adverse event was the result of an experimental vaccine, malaria infection, antimalarial medication, or an unknown cause (Nieman et al-2009).

In 2014, a 23-year-old healthy male subject experienced acute myocarditis during malaria treatment by atovaquone/proguanil. The diagnosis of acute myocarditis was based on a single episode of retrosternal chest pain, kinetics of elevated plasma troponin T, minor repolarization disturbances detected by ECG, and focal areas of subepicardial and midwall delayed enhancement of the left ventricle with some odema and hypokinesia observed by cardiac MRI. Follow-up cardiac MRI at almost 5 months showed normal function of both ventricles and disappearance of oedema. The investigators could not establish a definitive aetiology for the myocarditis but the following possibilities were proposed: *P falciparum* infection, rhinovirus infection, unidentified pathogens, hyper-immunization (the subject received 6 travel vaccines between the last immunization and the CHMI), anti-malarial treatment, or a combination of these factors (van Meer et al-2014).

The NMRC CTC staff participated in the development of a WHO Consensus Document for the Standardization of Design and Conduct of *P falciparum* Sporozoite Challenge Trials (Laurens et al-2012). This document is utilized by the NMRC CTC to help ensure the safety of all subjects from the day of challenge until completion of all post-challenge follow-up.

The safe use of malaria challenge via mosquito bite in 118 subjects by NMRC through 1992 has been summarized (Church et al-1997). A more recent review corroborates the same level of safety with further challenges by mosquito bite conducted through 2007 (Epstein et al-2007). The safe use of mosquitoes for immunization purposes has also been summarized (Hoffman et al-2002).

We will be using *P falciparum* which has no hypnozoite stage and therefore has no relapsing effects once treated appropriately. The mosquitoes used in this study (for immunization and CHMI) are raised in a closed insectary colony at NMRC/WRAIR in Silver Spring, Maryland to minimize the risk of infection with any other blood disease. In the insectary, the mosquitoes used for immunization or for challenge are infected with malaria by feeding on malaria-infected human blood. To minimize the risk of disease transmission the following precautions are taken:

1. The blood is obtained from commercial sources and has been tested for syphilis, hepatitis B and C viruses, and the HIV virus.
2. The length of time from when the mosquitoes feed on the infected blood until they feed on the subject is 17-23 days. This decreases any chances of transmitting an infectious agent. Specifically, there has been no reported transmission of human viruses, including HIV or hepatitis, using this system. In addition, mosquitoes are known to digest hepatitis B surface virions within 2-3 days of ingestion.

Prior to the challenge, at least 2 emergency contact numbers will be confirmed and verified as authentic for each subject. The clinical study team will review each subject's adherence to the schedule and safety follow up to date. This review will be done in order to identify any likelihood that the subject may be unreliable or noncompliant with study visits post-challenge. A subject who has been non-compliant with prior study visits may be excluded from the challenge phase and followed for safety.

Subjects will be monitored closely, especially following the challenge with viable sporozoites. As soon as malaria infection is documented, the subject will be treated as described in section 9.1.6.5. Prompt treatment minimizes the risk of developing a serious complication due to the malaria infection.

Subjects will receive instructions not to travel outside of the Washington, D.C., metro area from the day of challenge to 8 weeks after challenge. If a subject must travel and is at risk of developing malaria, he/she will be presumptively treated for malaria infection and proper arrangements will be made to ensure adequate follow-up.

5.7.1.3. Risks Associated with Antimalarial Medications

The primary antimalarial will be chloroquine as the NF54 strain is highly sensitive to chloroquine. Subjects who cannot tolerate chloroquine will be given Malarone (atovaquone/proguanil), a generally well tolerated and highly effective treatment regimen. The study team will discuss these medications and their possible side effects in detail as part of the informed consent process and prior to initiation of treatment for subjects who are infected with malaria.

The following side effects are possible for chloroquine:

- Nausea, vomiting, abdominal pain, diarrhea, dizziness, sleep disturbances, photosensitivity
- Headache, blurred vision, tinnitus

Rarely:

- Pruritus, rash, exacerbation of psoriasis
- Changes on electrocardiogram, hypotension

The following side effects are possible for atovaquone/proguanil (Malarone):

- Nausea, vomiting, abdominal pain, loss of appetite, diarrhea
- Temporary elevation of liver function tests
- Headache

Rarely:

- Coughing
- Anemia
- Oral ulcers
- Fever, swelling, rash, hair loss

Other highly effective antimalarial treatment drugs are available, including artemisinin combination therapies such as Coartem (artemether/lumefantrine) which will serve as the third line antimalarial treatment.

5.7.1.4. Risks Associated with Leukapheresis

As with blood sampling, leukapheresis carries a minimal risk of minor discomfort and the possibility of minor bruising at the site of the needle puncture and, rarely, the possibility of infection at the needle puncture site. In addition, side effects include fatigue, nausea, dizziness, feeling cold, perioral paresthesias, paresthesias of the extremities, urticaria, muscle cramps, coagulation changes, and decreased blood pressure. These side effects are thought to be related to the citrate anti-coagulant used in the apheresis procedure which can result in temporary decreases in blood calcium level. The symptoms thus occur during the procedure, not afterwards. Serious side effects like seizures or abnormal heart beat are very rare. Reactions to citrate (used to thin blood during leukapheresis) are usually relieved by slowing the use of citrate and by administering oral calcium carbonate. Refer to section 10 for additional information.

Leukapheresis is performed on blood donors in routine fashion at blood donation centers. The apheresis tubing is a completely closed system and is single use, minimizing the possibility of blood contamination. When subjects experience the symptoms described above that are associated with citrate anticoagulation, the symptoms usually ameliorate by slowing the infusion rate, and/or administering oral or intravenous calcium. Generally there is no need to interrupt the apheresis procedure. Leukapheresis procedures will be performed at the NMRC CTC by professional apheresis nurses.

5.7.1.5. Pregnancy

Pregnant women will be excluded from this study. Study subjects should not become pregnant and should use a reliable form of contraception for the duration of the study.

5.7.1.6. Lactation

Breastfeeding females will be excluded from this study. Subjects should not breastfeed for the duration of the study.

5.7.1.7. Risks Associated with Venipuncture/Blood Drawing

Throughout the study, blood samples will be collected from the subject for safety and immunologic testing. There are risks associated with blood drawing that include:

- Discomfort, swelling or bruising around the vein
- Lightheadedness/fainting
- Anemia following repeated blood draws

Rarely:

- Infection at the blood-drawing site
- Clinically significant hematoma

Throughout this study, the amount of blood collected will be no more than 525 milliliters in any 8-week period (this follows the guidelines of the American Association of Blood Banks). During these blood draws, light snacks and refreshments may be available for subjects for nutritional support. Subjects will be screened for HIV, hepatitis B, and hepatitis C for both assessment of suitability of study participation as well as for protection of laboratory and health care personnel. Standard procedures will be followed for handling blood and body fluid specimens.

5.7.1.8. Allergic Reaction

There is always the risk of a serious, or even life-threatening, allergic reaction to mosquito bites or antimalarial drugs. Medical emergency equipment is located in the NMRC CTC and will be available during immunizations and challenges at the WRAIR/NMRC insectary. This equipment is available to handle emergencies, such as anaphylaxis, angioedema, bronchospasm, and laryngospasm. There will always be a physician trained in Advanced Cardiac Life Support on-site during these procedures and for at least 30 minutes following immunization and challenge.

Medication and equipment (“crash cart”) to treat possible but unlikely allergic reactions extending beyond the bite site will be available in the feeding/immunization area and a study doctor will be present to monitor the immunization process and the subjects for at least 30 minutes after the immunization. Topical steroids and antihistamines will be made available to treat pruritus. As stated earlier, in over 10 years of our studies, no subjects exposed to the bites of irradiated mosquitoes in our laboratory have had breakthrough blood-stage infections. However, study subjects will still be counseled at the time of each immunization about the signs and symptoms of malaria and given 24 hour/day contact information should any develop.

5.7.1.9. Unknown Risks

As with all clinical research, there is the remote possibility of risks that are unknown or that cannot be foreseen based on current information.

5.7.2. Alternatives to This IND Product or Study

This vaccine is not being developed for licensure and is being tested in order to identify novel antigens and immune correlates of protection to develop next generation malaria vaccines. Thus, there is no alternative to this product other than not participating in the study.

5.7.3. Intended Benefit for Subjects

There is no direct benefit to the research subjects. However, their participation will aid scientists in developing an effective malaria vaccine. While this immunization may protect subjects from malaria, it will be emphasized that they should not rely on this to protect them from exposures to malaria. If they are ever exposed to malaria in the future (in nature or as part of another study) they should assume that they are not protected from malaria and should take normal measures (eg, malaria prophylaxis) to prevent infection.

There may be potential for indirect benefit, because of the medical screening subjects receive. Participation is voluntary and subjects may withdraw at any time without penalty or loss of benefits to which the subject is otherwise entitled.

5.7.4. Risks to the Study Personnel and the Environment

The principal risk in the clinical setting is primarily in the handling of needles that may be contaminated. Adherence to Standard Operating Procedures (SOP) for working with infectious agents and universal precautions will reduce the risk of exposure for these individuals.

Biohazardous waste that is generated attendant to vaccination will be disposed of as stipulated by local, state, and federal regulations.

Since both immunization and CHMI are delivered via the bites of mosquitoes, there is the risk of release of mosquitoes into the environment that carry radiation-attenuated malaria or fully infectious malaria. However, all mosquitoes that will be used will be kept in secure containers inside the insectary. Since the institution's initiation of mosquito husbandry, no such release of mosquitoes has ever occurred. This storage of mosquitoes also limits the risk of study personnel being bitten by malaria-infected mosquitoes during challenge. If an exposure were to occur, standard SOPs would be followed, and the exposed person would be treated with chloroquine if a significant risk had been incurred.

The risk of accidentally transmitting malaria to a person in the community will be negligible; infected mosquitoes will be restricted to the insectary with only temporary excursions to rooms outside of the insectary (eg, for irradiation) in which case the mosquito transport containers will not be opened. All infections in subjects will be treated promptly before gametocytes can develop, thus eliminating the potential for human to mosquito transmission.

5.8. Route of Administration, Dosage Regimen, Treatment Period, and Justification

5.8.1. Administration

Immunization will be administered via bites from *Anopheles stephensi* mosquitoes either infected (true-immunization) or uninfected (mock-immunization) with *P falciparum* sporozoites.

5.8.2. Dosage

The immunization procedures (doses) will be administered at approximately 4- to 5-week intervals. Total bites per dose will range from 200 to 400 per subject in the true immunized group (infectious plus noninfectious bites), depending on the numbers of mosquitoes allowed to feed on each study subject, which will be adjusted according to the infection rate in the batch of mosquitoes, with a similar expectation for total numbers of noninfectious bites in the mock-immunized group. The total number of bites received by each study subject, both infectious and noninfectous, will be estimated after each immunization session by counting the number of fed mosquitoes (as determined by blood in the abdomen) and by measuring the infection rate (as determined by hand-dissection of a subsample of mosquito salivary glands).

A target mean value of 960 infectious bites for Cohort 1 will result in a spread in the actual number of bites for the subjects of plus/minus 150 bites. A characteristic of immunization with *Pf*RAS via mosquito bite is that one can control the number of mosquitoes placed on the arm, but not the number that bite. Therefore, as immunizations progress, even though the same number of mosquitoes is placed on each subject during a given immunization session, the actual number of bites that are totaled after each immunization for each volunteer (determined via examination of the mosquitoes post feeding) varies over a range. Adjustments in the number of mosquitoes used

for each immunization session will be made according to infection rates in each mosquito batch and according to the progress of the entire cohort toward the target mean number of infectious bites of 960.

As a result, we anticipate the following targets and ranges for ***infectious bites*** for true-immunized volunteers:

Cohort 1: 960 (5 × 192) plus/minus 150 = approximately 800-1,100 bites

Cohort 2, Option A: 576 (3 × 192) plus/minus 150 = approximately 425-725 bites

Cohort 2, Option B: 768 (4 × 192) plus/minus 150 = approximately 620-920 bites

Cohort 2, Option C: 960 (5 × 192) plus/minus 150 = approximately 800-1,100 bites

Cohort 2, Option D: 1,152 (6 × 192) plus/minus 150 = approximately 1,000-1,300 bites

Cohort 2, Option E: 1,344 (7 × 192) plus/minus 150 = approximately 1,200-1,500 bites

Cohort 1, Hyperimmunity Sub-cohort: 1,536 plus/minus 150 = approximately 1,380-1,680

The *total number of bites* received by true-immunized volunteers will be larger than these expected ranges for *infectious bites*, depending on the infection rates in the mosquitoes. In other words, the volunteers will be bitten not just by infectious mosquitoes, but also by noninfectious mosquitoes that are invariably present in any batch of infected mosquitoes. The total number of bites may influence outcome due to the immunological effects of mosquito salivary gland antigens, which are the primary source of reactogenicity associated with the delivery of *Pf*RAS via the vector (not the sporozoites themselves). For this reason, we intend that the total number of bites in the true-immunized volunteers (infectious plus non-infectious bites) should equal the total number of bites in the mock-immunized volunteers (all noninfectious). Since the same number of mosquitoes will be placed in the feeding cages for both true- and mock-immunized volunteers, this intention will be met only if malaria infection does not influence biting activity. For this reason, as immunization progresses, if differences in total number of bites start to emerge between true- and mock-immunized volunteers, the number of mosquitoes placed on the arms of mock-immunized volunteers will be adjusted so that the total numbers of bites in the 2 groups are relatively comparable by the end of the immunization regimen. The total number of immunizing bites in Cohort 2 will be adjusted depending upon the results of the challenge of Cohort 1.

The Cohort 1 hyperimmunity subjects will receive the first 3 immunizations in conjunction with Cohort 2; therefore, they will receive approximately 576 infectious bites in addition to the estimated 960 infectious bites received in Cohort 1.

5.8.3. Compliance Statement

The study will be conducted according to the protocol and in compliance with International Conference on Harmonization (ICH), Good Clinical Practice (GCP), Belmont Principles, and other applicable regulatory and Department of Defense (DoD) requirements. All identified study personnel will be trained to perform their roles and will carry out their responsibilities in accordance with ICH and GCP guideline and clinic site SOPs. Roles and responsibilities of study staff are presented in Appendix B.

5.9. Study Population

Subjects will be recruited under a protocol approved by the NMRC. For this study, healthy adult subjects age 18-50 years will be recruited from the local national capital area. Enrollment will be open to both military and civilian individuals.

It is estimated that approximately 200 subjects will be screened in order to enroll 60 eligible subjects, of which it is planned 32 subjects will be immunized (true- and mock-immunized), 12 subjects will be nonimmunized infectivity controls, and up to 16 alternates will be enrolled. The planned number of subjects in each category may be exceeded in the event that subjects must be replaced with alternates (eg replacement of immunized subject within the study schedule window immediately following Immunization 1 secondary to withdrawal of consent).

This approximate 4:1 ratio of screened to eligible subjects is based on our previous experience in recruiting for malaria vaccine trials in this study population. In this study, a subject is defined as being enrolled after he or she has met all eligibility criteria and has completed the initial study-specific procedure (pre-immunization leukapheresis, immunization, CHMI).

A 20-30% failure rate of leukapheresis procedures (the initial study-specific procedure for true- and mock-immunized groups) via peripheral intravenous access is expected due to inability to gain intravenous access with a large caliber steel needle, vasovagal responses, and inadequate return flow of blood products secondary to small veins. When a pre-immunization failure occurs, the subject may be brought back to attempt leukapheresis again. Additionally, a subject may be enrolled into the study as a true- or mock-immunized subject following a failed pre-immunization leukapheresis. In this case, enrollment occurs at the time of Immunization 1; however, it is preferable to enroll subjects that successfully complete pre-immunization leukapheresis in order to maximize the scientific objectives of the study.

5.10. Study Site

The study will be conducted at the NMRC CTC, building 17B at the WRNMMC in Bethesda Maryland. All visits other than immunization visits, challenge visits, and hotel stay will take place at the NMRC CTC. Immunization and challenge procedures will be conducted at the WRAIR Insectary, 503 Robert Grant Avenue, Silver Spring, Maryland. Site for post-challenge overnight stays for NMRC: Hotel in close proximity to NMRC CTC.

6. Trial Objectives and Purpose

6.1. Primary Objective

1. Assess safety and tolerability in malaria-naïve adults of immunization with radiation-attenuated *P falciparum* sporozoites (*Pf*RAS) administered via mosquito bites compared with mock immunization via noninfectious mosquito bites.
2. Induce protective immunity against controlled human malaria infection (CHMI) in approximately 50% of study subjects via immunization with PfRAS.

6.2. Secondary Objectives

The secondary objectives of this study are as follows:

1. Identify biomarkers of protection, including host response and antigenic targets, by comparing protected, nonprotected, and mock-immunized subjects.
2. Identify immune components of high-grade, durable protection in hyperimmunized subjects.

7. Trial Design

7.1. Study Endpoints

7.1.1. Primary Endpoints

The primary endpoints of this study are as follows:

1. Occurrence of solicited AEs from administration of study immunization (*Pf*RAS) through 7 days post-dosing.
2. Occurrence of unsolicited AEs from administration of immunization through 14 days post-dosing.
3. Occurrence of laboratory AEs from administration of study immunization through 7 days post-dosing.
4. Occurrence of SAEs from administration of immunization through the duration of the trial.
5. Occurrence of signs and symptoms related to malaria infection starting 7 days post-CHMI (these will not be recorded as adverse events because they are expected as a result of malaria infection).
6. Development of parasitemia and time to parasitemia after malaria challenge.

7.1.2. Secondary Endpoints

The secondary endpoints of this study are as follows:

1. Collection and storage of PBMCs, serum, and whole blood for use in a comprehensive effort to identify and validate biomarkers of immunological protection associated with immunizing human subjects with *Pf*RAS, comparing read-outs between protected and nonprotected subjects and between immunized and mock-immunized subjects.

7.2. Overall Study Design

The clinical study design is an open-label safety and biomarkers of protection study in healthy malaria-naïve adults, who will receive bites from *Anopheles stephensi* mosquitoes either infected with *Pf*RAS (true-immunization) or noninfected (mock-immunization). The study design in depicted in Figure 4. The goal of primary immunization is to achieve approximately 50% sterile protection in the true-immunization study subjects in order to facilitate the identification of biomarkers and correlates of protection, and the goal of secondary immunization in a subgroup of study subjects is to explore high-grade, durable protection (second-tier immunity). These study objectives will be achieved via a comprehensive, systems biology-based effort to identify and validate biomarkers of protection with *Pf*RAS immunization, comparing sterilely protected to non-protected study subjects.

Following true-immunization or mock-immunization, study subjects and non-immunized infectivity controls will receive a challenge via the bites of 5 *An stephensi* mosquitoes carrying infectious *P falciparum* sporozoites within a controlled clinical environment (CHMI) to determine the level of sterile protection.

To increase sample size and enable flexibility toward achieving the goal of approximately 50% sterile protection, there will be 2 cohorts; the first cohort will be initiated prior to the second. Each cohort will include true-immunized and mock-immunized study subjects and CHMI infectivity controls. Both cohorts will receive identical immunization regimens if protection in the first cohort is 40%-60%; alternatively, the second cohort will receive more or fewer mosquito bites, if protection in the first cohort is < 40% or > 60%, respectively.

A sub-cohort of protected subjects from Cohort 1 (6 subjects maximum) will be offered the optional opportunity to receive 3 secondary immunizations and a secondary challenge in conjunction with Cohort 2 in order to explore long duration, high grade immunity.

Figure 4: Study Design

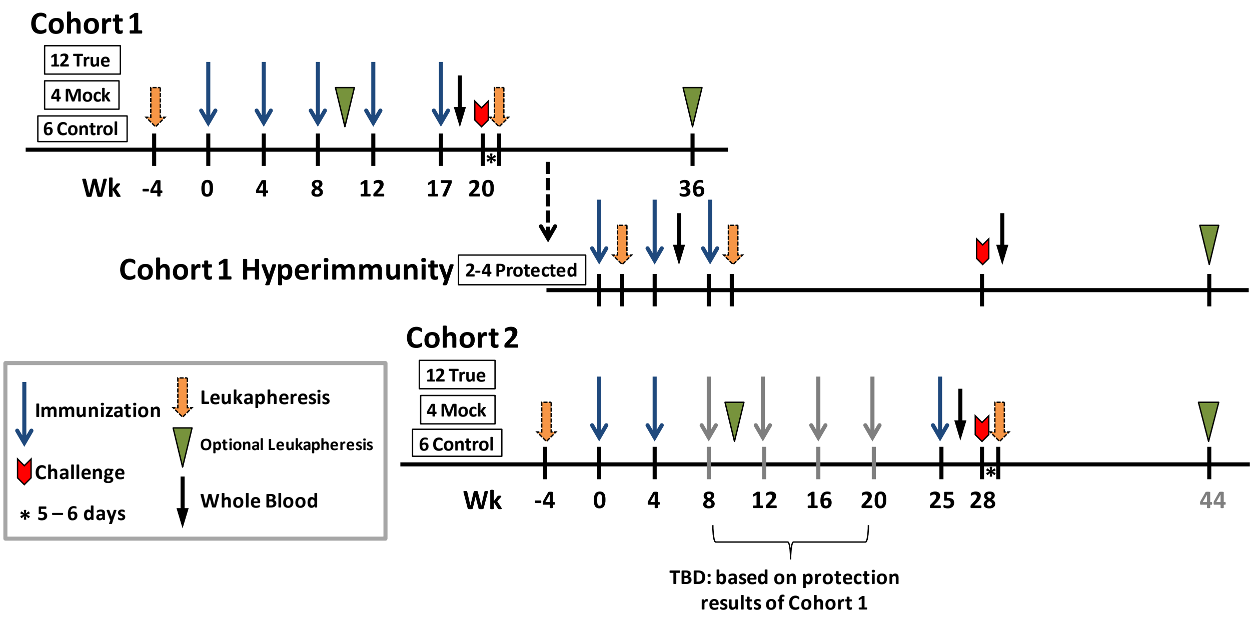

The interval between the penultimate and final immunization will be 5 weeks, rather than 4 weeks.

7.2.1. Immunization

7.2.1.1. Cohort 1

Cohort 1 will be initiated prior to start of Cohort 2. Twelve malaria-naïve adults will receive 5 doses of approximately 200 infectious bites (200-400 bites total) from *Pf*RAS-infected mosquitoes (true-immunization) and 4 additional subjects will receive the same from irradiated, uninfected mosquitoes (mock-immunization). The target dose is 960 infectious bites for the group mean. This should result in full sterile protective immunity in some true-immunized individuals. The target of 960 bites was selected because earlier studies (section 5.6.2) suggest that this is an approximate point of transition between nonprotection and protection. Following the fifth immunization, all immunized subjects (true-immunized and mock-immunized) plus 6 nonimmunized infectivity controls will undergo CHMI approximately 3 weeks after the fifth immunization session.

7.2.1.2. Cohort 2

As highlighted in section 7.2, an adjustable dosing schedule for Cohort 2 will be used to balance out a skewed protection result in Cohort 1 should this occur (< 40% or > 60% protection), to achieve ~ 50% sterile protection averaged over the 2 cohorts. The first immunization for Cohort 2 will take place after the challenge results for Cohort 1 are known, allowing adjustment in the number of immunizations in Cohort 2.

Subjects will receive 3 to 7 doses of approximately 200 infectious bites (200-400 bites total) from irradiated *P falciparum*-infected mosquitoes (true-immunization) and 4 additional subjects will receive the same from irradiated, uninfected mosquitoes (mock-immunization). Depending upon the proportion of subjects that have sterile protection in Cohort 1, Cohort 2 will be assigned to 1 of 5 options (Table 7).

Table 7: Options for Cohort 2 Immunization Sessions Based on Level of Protection in Cohort 1

Level of Sterile Protection (Cohort 1)	Number of Immunization Sessions (Cohort 2)
> 75%	3
> 60%-75%	4
40%-60%	5
25%-< 40%	6
< 25%	7

The adjustable dosing schedule illustrated in Table 7 is the planned method to achieve approximately 50% sterile protection averaged over the 2 cohorts; however, there is the potential for heavily skewed results from Cohort 1. In the event of heavily skewed results, trial design changes for Cohort 2 may also include extending the interval between last immunization and CHMI up to 6 weeks in case of high-grade protection in Cohort 1, extending the post-challenge leukapheresis up to 35 days in case of very poor protection in Cohort 1, and establishing 4 challenge days for Cohort 2 rather than 2 challenge days as described earlier to create a uniform timepoint (this would require 6 additional infectivity controls). Any of these trial design changes other than the adjustable dosing schedule will require the submission of a protocol amendment once the protection results are known following the Cohort 1 challenge.

7.2.1.3. Cohort 1 Hyperimmunity Sub-Cohort

This continuation phase sub-cohort will occur simultaneously with Cohort 2 in order to maximize resource utilization and to overcome logistical constraints. Due to the flexible design of Cohort 2, it is uncertain how long the interval between the conclusion of Cohort 1 and the initiation of Cohort 2 will be. It is estimated that it will be approximately 3 months; therefore, Cohort 1 Hyperimmunity Sub-Cohort subjects will follow the prescribed sampling schedule, safety lab, and safety visit schedule for Cohort 1 until the initiation of Cohort 2. At that time, the continuation sub-cohort will follow a unique sampling schedule but will adhere to the Cohort 2 safety lab and safety visit schedule. Of note, the Hyperimmunity Sub-cohort will only receive the first 3 immunizations of Cohort 2 regardless of the final Cohort 2 dosing regimen as determined

by Cohort 1 protection results. Following the third immunization (secondary immunization 3), the Sub-cohort will be scheduled for a secondary challenge to correspond with the primary challenge of Cohort 2. Due to the adjustable dosing schedule for Cohort 2, the interval between the third (final) secondary immunization of the sub-cohort and the secondary challenge of the Sub-cohort may vary from 3 to 19 weeks.

7.2.2. Challenge Procedure

Mosquitoes infected via membrane feeds prior to challenge and containing sporozoites in their salivary glands will be allowed to feed on the subjects. The mosquitoes will be dissected to confirm the presence of a blood meal and to determine the infectivity rate and the salivary gland score. Additional mosquitoes will be allowed to feed until there are 5 bites with the presence of a blood meal, with a minimum of 2+ salivary gland score, has been achieved. Beginning 7 days after the bites, subjects will be monitored daily for parasitemia by microscopic examination of thick blood smears, with immediate antimalarial treatment upon detection of parasites in the blood. In the absence of sterile protection, most subjects will develop patent parasitemia by microscopy on days 9-14 (range 7 to 23).

For scientific reasons, the interval between challenge and post-challenge leukapheresis should be 5-6 days. However, only 4 subjects can practically be apheresed on a given day. In order to accommodate these parameters, the challenge will be staggered over a 2 day period. Thus, about half of the immunized subjects will be challenged with 3 Infectivity Controls on 1 day and 2 days later, the remaining immunized subjects plus 3 Infectivity Controls will be challenged. The leukapheresis will then occur on Day 5 or Day 6 after challenge for each subject.

7.2.3. Leukapheresis Procedure Requirement

This study requires a large amount of peripheral blood mononuclear cells (PBMCs) to identify biomarkers and correlates of protection. Leukapheresis will be performed to collect this large quantity of cells. The procedure is performed routinely by blood banks in order to obtain blood components from healthy subjects, in the same way that whole blood is donated. In this study, we are aiming for about 4 billion PBMCs harvested per procedure (about 2 hours on the apheresis machine).

Referring to the trial design in Figure 4, a large pre-challenge whole blood draw is shown as a black solid arrow and leukapheresis procedures as orange dotted arrows. The pre-immunization and post-challenge leukapheresis procedures are planned procedures in this study as these samples are necessary to address some of the objectives of the study. An optional leukapheresis procedure is shown as a green triangle. This procedure has been deemed optional in order to reduce participant fatigue and drop-out. Further, participation in this optional leukapheresis procedure will be limited to a maximum of 50% of the subjects because of the possibility that leukapheresis at this intermediate timepoint might be deleterious to the induction of protective immune responses. In the event that a subject fails pre-immunization leukapheresis, it is possible to bring the subject back for a second attempt to complete the procedure. Additionally, a subject may be enrolled into the study as a true- or mock-immunized subject following a failed pre-immunization leukapheresis. In this case, enrollment occurs at the time of Immunization 1; however, it is preferable to enroll subjects that successfully complete pre-immunization leukapheresis in order to maximize the scientific objectives of the study.

Many smaller volume whole blood draws (not shown in Figure 4) will be collected throughout the clinical trial in order to support the study objectives and monitor research subject safety. An additional optional leukapheresis procedure will be offered at approximately 4–6 months post-challenge. All study subjects (100%) may participate in this procedure. The study event schedule (Appendix C) details the blood volumes, timepoints, and reason for sample collection for all sample collections.

7.3. Immunoassays and Biological Samples

The sample requirements and timepoints for the immunoassays and antigen discovery efforts are shown in Table 8. This table details the specific immunoassays, type and volume of samples, and the collection timepoints after each immunization and CHMI. Of note, the immunoassays listed may be performed in more than one location (ie, NMRC/WRAIR/SCRI designates that an immunoassay may be performed at any of the locations or any combination of the locations).

7.3.1. Immunoassays

Humoral and cellular immune responses against whole sporozoite and pre-erythrocytic stage antigens (including *Pf*CSP, *Pf*SSP2/TRAP, *Pf*Exp1, *Pf*LSA1, and *Pf*s16) have been demonstrated in individuals immunized with irradiated sporozoites (Doolan and Hoffman-1997; Egan et al-1993; Malik et al-1991; Wizel et al-1995a, 1995b). There is evidence that the protection induced by irradiated sporozoites is primarily mediated by T cell responses that recognize malarial antigens presented on infected hepatocytes (Rogers et al-1992). In particular, early studies highlighted CD8+ cytotoxic T cells (CTL) as a primary immune effector mechanism. However, antibodies and CD4+ responses have also been demonstrated against sporozoite as well as the 5 well-characterized pre-erythrocytic stage antigens (Doolan and Hoffman-1997, 2000; Egan et al-1993; Krzych et al-1995). Therefore, in order to screen and identify novel antigens associated with sterile protection, both humoral and cellular immune responses to these novel antigens will be assessed in immunized subjects.

Table 8: Sample Requirements for Immunoassays and Antigen Discovery

Assay		Sample	Time Points (D = Day Relative to Immunization, CHMI or Leukapheresis)
**Immunoassays**			
**Global**	Transcriptome (SCRI)	PaxGene<br>2.5 mL	Imm1 & 5: D0, 3hr[a], 6hr[a], 1, 3, 7, 14<br>Imm2 – 4: D0, 3, 7, 14<br>CHMI: D0, 1, 7<br>CHMI for Infectivity Controls: D0, 1, 7<br>Hyperimmunized Sub-cohort – Boost1: D0, 3, 7, 14; Boost3: D0, 3
	Bio-Plex® (SCRI)	Serum<br>4 mL	Imm1 & 5: D0, 6hr[a], 1, 3, 7, 14<br>Imm2 – 4: D0, 3, 7, 14<br>CHMI: D0, 1, 7<br>CHMI for Infectivity Controls: D0, 1, 7<br>Hyperimmunized Sub-cohort – Boost1: D0, 3, 7, 14; Boost3: D0, 7
**Humoral**	ELISA (NMRC/WRAIR)	Serum<br>4 mL[b]	Imm1: D0<br>All Imm: D14<br>CHMI: D0, 28, 56, 112<br>CHMI for Infectivity Controls: D0, 28, 56, 112<br>Hyperimmunized Sub-cohort – All Boosts: D0; Re-CHMI: D0, 28, 56, 112
	Luminex® (NMRC/WRAIR)	Serum<br>4 mL[b]	Imm1: D0<br>All Imm: D14<br>CHMI: D0, 28, 56, 112<br>CHMI for Infectivity Controls: D0, 28, 56, 112<br>Hyperimmunized Sub-cohort – All Boosts: D0; Re-CHMI: D0, 28, 56, 112
	Ab ILSDA (NMRC)	Serum<br>4 mL[b]	Imm1: D0<br>All Imm D14<br>CHMI: D0, 28, 56, 112<br>CHMI for Infectivity Controls: D0, 28, 56, 112<br>Hyperimmunized Sub-cohort – All Boosts: D0; Re-CHMI: D0, 28, 56, 112
	ISTI (SCRI)	Serum<br>4 mL[c]	Imm1 - 4: D0<br>Imm4: D28<br>CHMI: D0, 28, 56, 112<br>CHMI for Infectivity Controls: D0, 28, 56, 112<br>Hyperimmunized Sub-cohort – All Boosts: D0; Re-CHMI: D0, 28, 56, 112

Table 8: Sample Requirements for Immunoassays and Antigen Discovery (Continued)

Assay		Sample	Time Points (D = Day Following Immunization or CHMI)
**Humoral (cont.)**	Proteome Array	Serum<br>4 mL[c]	Imm 1 - 4: D0<br>Imm4: D28<br>CHMI: D0, 28, 56, 112<br>CHMI for Infectivity Controls: D0, 28, 56, 112<br>Hyperimmunized Sub-cohort – All Boosts: D0; Re-CHMI: D0, 28, 56, 112
	Plasmablast (BMGF)	PBMC 17 mL<br>68 mL<br>85 mL	Imm1: D7 (17 mL)<br>Imm2: D7 (17 mL)<br>Imm5: D7 (68 mL)<br>Hyperimmunized subjects – Boost1 & 3; D7 (leukapheresis); Boost 2 & Re-CHMI D7 (85 mL)
	Memory B cell ELISpot (NMRC/WRAIR/U. Washington)	PBMC	Pre-Imm Leukapheresis<br>Imm3: D14 Leukapheresis/PBMC<br>CHMI1: D5/6, 112 Leukapheresis/PBMC[d]<br>CHMI2:D3,D112 Leukapheresis/PBMC
	Antigen-specific Memory B cell Flow Cytometry (NMRC/WRAIR/U. Washington)	PBMC	Pre-Imm Leukapheresis<br>Imm1: D0, 3, 7, 14<br>Imm2: D7<br>Imm3: D0, 3, 5-9, 14 (PBMC/Leukapheresis)<br>Imm5: D0, 7, 14<br>CHMI1: D5/6 (Leukapheresis), 42, 56, 112 (PBMC/Leukapheresis)<br>Hyimm1: D0, 3, 7(PBMC/Leukapheresis),14<br>Hyimm2: D7,14<br>Hyimm3: D7 (Leukapheresis),D14<br>CHMI2: D0-3, 7-9, 42, 56, D112 (PBMC/Leukapheresis)
	Antigen-specific Memory B cell BCR and RNA sequencing (NMRC/U. Washington)	PBMC	Pre-Imm Leukapheresis<br>Imm1: D0, 3, 7, 14<br>Imm2: D7<br>Imm3: D0, 3, 5-9, 14 (PBMC/Leukapheresis)<br>Imm5: D0, 7, 14<br>CHMI1: D5/6 (Leukapheresis), 42, 56, 112 (PBMC/Leukapheresis)<br>Hyimm1: D0, 3, 7(PBMC/Leukapheresis),14<br>Hyimm2: D7,14<br>Hyimm3: D7 (Leukapheresis),D14<br>CHMI2: D0-3, 7-9, 42, 56, D112 (PBMC/Leukapheresis)
	Humanized Mice (SCRI)	Serum<br>20 mL	CHMI: D0<br>Hyperimmunized Sub-cohort –Re-CHMI: D0

	IFA (NMRC/WRAIR)	Serum<br>4 mL[b]	Imm1: D0<br>All Imm: D14<br>CHMI: D0, 28, 56, 112<br>CHMI for Infectivity Controls: D0, 28, 56, 112<br>Hyperimmunized Sub-cohort – All Boosts: D0; Re-CHMI: D0, 28, 56, 112
	Opsono-phagocytosis assay (WRAIR)	Serum<br>4 mL[b]	Imm1: D0<br>All Imm: D14<br>CHMI: D0, 28, 56, 112<br>CHMI for Infectivity Controls: D0, 28, 56, 112<br>Hyperimmunized Sub-cohort – All Boosts: D0; Re-CHMI: D0, 28, 56, 112
**CMI**	FluoroSpot (NMRC)	PBMC<br>17 mL	All Imm: D0<br>Imm1: D7<br>CHMI: D0, 42<br>Hyperimmunized Sub-cohort: All Boosts: D0; Re-CHMI: D0, 42<br>Leukapheresis Time Points will also be evaluated in addition to the PBMCs above:<br>Pre-Imm Leukapheresis<br>Imm3: D14 Leukapheresis<br>CHMI1: D5/6, 112 Leukapheresis<br>Boost 1: D7 Leukapheresis<br>Boost 3: D7 Leukapheresis<br>CHMI2: D112 Leukapheresis
	Ex vivo Flow Cytometry (SCRI)	PBMC<br>17 mL	Imm1 & 3: D0, 3, 7, 14<br>Imm5: D0, 7<br>Hyperimmunized Sub-cohort: Boost1: D0, 3, 7; Boost3 D7
	Functional Flow Cytometry (SCRI/NMRC)	PBMC<br>25.5 mL	Imm1: D0<br>Imm1, 3 & 5: D14<br>CHMI: D42, 56, 112<br>Hyperimmunized Sub-cohort: Boost1: D0; All Boosts: D14; Re-CHMI: D0, 42, 56, 112<br>Leukapheresis Time Points will also be evaluated in addition to the PBMCs above:<br>Pre-Imm Leukapheresis<br>Imm3: D14 Leukapheresis<br>CHMI1: D5/6, 112 Leukapheresis<br>Boost 1: D7 Leukapheresis<br>Boost 3: D7 Leukapheresis<br>CHMI2: D112 Leukapheresis

Table 8: Sample Requirements for Immunoassays and Antigen Discovery (Continued)

Assay		Sample	Time Points (D = Day Following Immunization or CHMI)
**CMI (cont._**	Memory Phenotype Flow Cytometry (NMRC/WRAIR/SCRI)	PBMC	Pre-Imm Leukapheresis<br>Imm3: D14 Leukapheresis<br>CHMI1: D5/6, 112 Leukapheresis<br>Boost 1: D7 Leukapheresis<br>Boost 3: D7 Leukapheresis<br>CHMI2: D112 Leukapheresis
	Transcriptional and epigenetic analysis of memory T cells (SCRI/U.Washington)	PBMC	Pre-Imm Leukapheresis<br>Imm1: D0, 3, 7, 14<br>Imm3: D0, 3, 7, 14 (Leukapheresis/PBMC)<br>Imm5: D0, 7, 14<br>CHMI1: D5/6 (Leukapheresis), 42, 56, 112 (Leukapheresis/PBMC)<br>Hyimm1: D0, 3, 7(Leukapheresis/PBMC),14<br>Hyimm2: D7,14<br>Hyimm3: D7 (Leukapheresis),D14<br>CHMI2: D3, 7, 42, 56, D112 Leukapheresis/PBMC
	Cytokine Profile Mesoscale (NMRC/WRAIR)	PBMC	Pre-Imm Leukapheresis<br>Imm3: D14 Leukapheresis<br>CHMI1: D5/6, 112 Leukapheresis<br>Boost 1: D7 Leukapheresis<br>Boost 3: D7 Leukapheresis<br>CHMI2: D112 Leukapheresis
	Cultured ELISpot and Flow Cytometry (NMRC/WRAIR)	PBMC	Pre-Imm Leukapheresis<br>Imm3: D14 Leukapheresis<br>CHMI1: D5/6, 112 Leukapheresis<br>Boost 1: D7 Leukapheresis<br>Boost 3: D7 Leukapheresis<br>CHMI2: D112 Leukapheresis
	Regulatory Phenotype Flow Cytometry (USUHS)	PBMC	Pre-Imm Leukapheresis<br>Imm3: D14 Leukapheresis<br>CHMI1: D5/6, 112 Leukapheresis<br>Boost 1: D7 Leukapheresis<br>Boost 3: D7 Leukapheresis<br>CHMI2: D112 Leukapheresis

**Antigen Discovery**			
	CMI Immunoscreening (NMRC)	PBMC	Pre-Imm Leukapheresis<br>Imm3: D14 Leukapheresis<br>CHMI1: D5/6, 112 Leukapheresis<br>Boost 1: D7 Leukapheresis<br>Boost 3: D7 Leukapheresis<br>CHMI2: D112 Leukapheresis
	Serum Antibody Screen (NMRC)	Serum/plasma	Imm1: D0<br>Imm5: D14<br>Hyperimmunized Sub-cohort: All Boosts: D0; Re-CHMI: D0, 28, 56, 112
	Protein Array (SCRI)	Serum	All Imm: D0<br>Imm4: D28<br>CHMI: D0, 28, 56, 112<br>CHMI for Infectivity Controls: D0, 28, 56, 112<br>Hyperimmunized Sub-cohort – All Boosts: D0; Re-CHMI: D0, 28, 56, 112
	ATLAS™ (Genocea)	PBMC	Leukapheresis

[a] Optional for subjects.
[b] Samples for ELISA, Luminex, IFA,Ab ILSDA and opsono-phagocytosis assays will come from one 4 mL blood draw.
[c] Samples for ISTI and Proteome Array will come from one 4 mL blood draw.
[d] Leukapheresis Timepoints: Pre-immunization, Immunization (Imm)3: D14 (optional), CHMI: D5 or 6, 4-6 months (optional).

7.3.1.1. Global Immune Response Assays

7.3.1.1.1. PBMC Transciptome Analysis: SCRI (2.5 mL)

Innate immune responses are sensitively reflected in the PBMC transcriptome shortly after vaccination. RNA will be stabilized and purified using PAXGene blood collection tubes and RNA kits. RNA will also be extracted from cryopreserved PBMCs for some timepoints. RNA sequences will be detected de novo on an Illumina HiSeq2000 instrument without the need for prior knowledge of the transcript sequences to be measured, and with a dynamic range spanning 5 orders of magnitude. Raw data will be deconvoluted into meaningful transcriptome profiles. Note: The 3 and 6 hr timepoints will be optional for subjects.

Timepoints for Transcriptome Analysis:

- Imm1 & 5: D0, 3hr, 6hr, 1, 3, 7, 14
- Imm2 – 4: D0, 3, 7, 14
- CHMI: D0, 1, 7
- CHMI for Infectivity Controls: D0, 1, 7
- Hyperimmunized Sub-cohort:
  - Boost1: D0, 3, 7, 14
  - Boost3: D0, 3

7.3.1.1.2. Serum Analyte Multiplex Assay (Bio-Plex®): SCRI(4 mL)

Serum proteins released by innate immune cells in response to primary immunization, as well as proteins secreted by adaptive immune cells induced by sequential boosting, will be measured by Bio-Plex® assay. A commercial multiplex bead-based assay designed to quantitate 39 cytokines/chemokines will be used to define the protein profiles in plasma after each immunization. Note: The 6 hr timepoint will be optional for subjects.

Timepoints for Bio-Plex®:

- Imm1 & 5: D0, 6hr, 1, 3, 7, 14
- Imm2 – 4: D0, 3, 7, 14
- CHMI: D0, 1, 7
- CHMI for Infectivity Controls: D0, 1, 7
- Hyperimmunized Sub-cohort:
  - Boost1: D0, 3, 7, 14
  - Boost3: D0, 7

7.3.1.2. Humoral Response Assays

7.3.1.2.1. Antibody ELISA: NMRC/WRAIR (4 mL)

Serum IgG antibodies specific for pre-erythrocytic antigens (CSP, SSP2/TRAP, AMA-1, LSA-1, and CelTOS) will be quantified by the International Reference Center for Malaria Serology Laboratory (WRAIR) using validated ELISA assays. In addition, proteins from 3 additional antigens that will be prioritized from antigen discovery programs will be prepared using the wheat germ cell-free protein expression system and used to detect serum IgG antibodies to novel pre-erythrocytic antigens.

Timepoints for ELISA:

- Imm1: D0
- All Imm: D14
- CHMI: D0, 28, 56, 112
- CHMI for Infectivity Controls: D0, 28, 56, 112
- Hyperimmunized Sub-cohort:
  - All Boosts: D0
  - Re-CHMI: D0, 28, 56, 112

7.3.1.2.2. Antibody Multiplex Assay (Luminex®): NMRC/WRAIR (4 mL)

Approximately 5 known and 10 hypothetical pre-erythrocytic vaccine candidate antigens will be coupled to Luminex beads, with a different bead spectral signature employed for each antigen, to allow for simultaneous determination, in a given subject's serum, of antibody levels against a large repertoire of representative pre-erythrocytic antigens. Secondary antibodies specific for human IgG1, IgG2, IgG3 and IgG4 will be used to determine sub-class distribution. The assays will also be repeated in the presence and absence of a chaotropic agent to assess the relative avidities of the pre-erythrocytic-specific antibodies.

Timepoints for Luminex®:

- Imm1: D0
- All Imm: D14
- CHMI: D0, 28, 56, 112
- CHMI for Infectivity Controls: D0, 28, 56, 112
- Hyperimmunized Sub-cohort:
  - All Boosts: D0
  - Re-CHMI: D0, 28, 56, 112

7.3.1.2.3. Antibody Inhibition of Liver-Stage Development Assay (ILSDA): NMRC (4 mL)

The inhibition of liver-stage development assay (ILSDA) will be used to test for functional anti-parasitic activity of antibodies targeting the malaria parasite during hepatocyte invasion and subsequent development within hepatocytes. Sera will be incubated with freshly dissected *Pf* sporozoites and subsequently added to cultures of cryopreserved primary human hepatocytes. Parasite 18S ribosomal RNA will be quantified 5 days after invasion.

Timepoints for Ab ILSDA

- Imm1: D0
- All Imm: D14
- CHMI: D0, 28, 56, 112
- CHMI for Infectivity Controls: D0, 28, 56, 112
- Hyperimmunized Sub-cohort:
  - All Boosts: D0
  - Re-CHMI: D0, 28, 56, 112

7.3.1.2.4. Antibody Inhibition of Sporozoite Traversal and Invasion Assay (ISTI): SCRI (4 mL)

A flow cytometric-based assay will be used to measure inhibition of sporozoite traversal and invasion (ISI). Dissected *Pf* sporozoites will be incubated with plasma then added to the human HCO4 hepatocyte cell line. Cell and sporozoites will be incubated for 90 min in the presence of dextran before being trypsinized, permeabilized, and stained with anti-CSP antibodies. The percentage of CSP-positive HCO4 cells and dextran-positive cells will be determined by flow cytometry.

Timepoints for ISTI

- Imm1 - 4: D0
- Imm4: D28
- CHMI: D0, 28, 56, 112
- CHMI for Infectivity Controls: D0, 28, 56, 112
- Hyperimmunized Sub-cohort:
  - All Boosts: D0
  - Re-CHMI: D0, 28, 56, 112

7.3.1.2.5. *Pf* Proteome Array: SCRI (4 mL)

Pf array including all pre-erythrocytic and erythrocytic stage proteins will be generated based on their known or selected expression in sporozoite or liver-stage parasites, based on published data in *PlasmoDB* and recent unpublished data from SCRI. Protein arrays will be printed onto nitrocellulose coated glass slides by Antigen Discovery Inc (ADi). The proteome chips will then

be used to assess plasma antibody responses before and after *Pf*RAS immunization and challenge.

Timepoints for Proteome Array:

- Imm1 - 4: D0
- Imm4: D28
- CHMI: D0, 28, 56, 112
- CHMI for Infectivity Controls: D0, 28, 56, 112
- Hyperimmunized Sub-cohort:
  - All Boosts: D0
  - Re-CHMI: D0, 28, 56, 112

7.3.1.2.6. Plasmablasts: BMGF (17 - 85 mL)

Single antigen-specific antibody-secreting cells will be isolated by fluorescence-activated cell sorting, followed by sequencing of immunoglobulin gene transcripts. Novel technology (Atreca, Inc., CA) will be used to produce recombinant monoclonal antibodies for evaluation in functional analyses (in vitro and in vivo) and antigen identification.

Timepoints for Plasmablasts:

- Imm1: D7 (17 mL blood)
- Imm2: D7 (17 mL blood)
- Imm5: D7 (68 mL blood)
- Hyperimmunized Sub-cohort:
  - Boost 1 & 3: D7 (leukapheresis samples)
  - Boost 2 & re-CHMI: D7 (85 mL blood)

7.3.1.2.7. Memory B cell Enzyme-Linked Immunospot Assay (ELISpot): NMRC/WRAIR/U. Washington

Memory B cells proposed to be important for long-term humoral immunity will be quantified in a B cell ELISpot assay. Cryopreserved PBMCs will be cultured in vitro for 5-6 days with a combination of mitogens to allow the memory B cells to proliferate and differentiate into antibody-secreting cells. Antigen-specific antibody-secreting cells will be quantified on ELISpot plates coated with malaria-specific antigens.

Timepoints for Memory B cell ELISpot:

- Pre-Imm Leukapheresis
- Imm3: D14 Leukapheresis
- CHMI1: D5/6, 112 Leukapheresis[e]
- CHMI2:D3,D112 Leukapheresis/PBMC

7.3.1.2.8. Antigen-Specific B cell Flow Cytometry: NMRC/WRAIR/U. Washington

Characterization of the differentiation stage of antigen-specific memory B cell responses will be performed on cryopreserved PBMCs obtained from the leukapheresis samples. B cells will be positively selected by magnetic bead separation and then stained with antigen-coated beads (CSP, AMA-1, LSA-1, CelTOS or SSP2/TRAP) in combination with various subset-specific markers (mIgM, mIgG, CD27, CD21, CD10, and CD20). B cell subsets will be identified from PBMC and leukapheresis samples and may be enriched with antibodies or tetramer/decoy and magnetic bead technique. The cells will then be stained with tetramers and/or B cell subset markers for flow cytometry.

Timepoints for Antigen-Specific B cell Flow Cytometry:

- Pre-imm (Leukapheresis)
- Imm1: D0, 3, 7, 14
- Imm2: D7
- Imm3: D0, 3, 5-9, 14 (PBMC/Leukapheresis)
- Imm5: D0, 7, 14
- CHMI1: D5/6 (Leukapheresis), 42, 56, 112 (PBMC/Leukapheresis)
- Hyimm1: D0, 3, 7(PBMC/Leukapheresis),14
- Hyimm2: D7,14
- Hyimm3: D7 (Leukapheresis), 14
- CHMI2: D0-3, 7-9, 42, 56, 112 PBMC/Leukapharesis

7.3.1.2.9. Antigen-Specific B cell BCR and RNA sequencing: U. Washington

CSP-specific B cells (all isotypes and differentiation states) will be identified from PBMC and leukapheresis samples and enriched with a tetramer/decoy and magnetic bead technique. The cells will then be stained with B cell subset markers and single-cell plate sorted. PCR and single-cell RNA sequencing of the CSP-specific B cells will yield transcriptomic data and BCR sequences for somatic hypermutation and repertoire analysis and will be expressed to form the antibodies for affinity testing.

Timepoints for Antigen-Specific B cell BCR and RNA sequencing:

- Pre-immunization (Leukapheresis)
- Imm1: D0, 3, 7, 14
- Imm2: D7
- Imm3: D0, 3, 5-9, 14 (PBMC/Leukapheresis)
- Imm5: D0, 7, 14
- CHMI1: D5/6 (Leukapheresis), 42, 56, 112 (PBMC/Leukapheresis)
- Hyimm1: D0, 3, 7 (PBMC/Leukapheresis), 14
- Hyimm2: D7,14
- Hyimm3: D7 (Leukapheresis), 14
- CHMI2: D0-3, 7-9, 42, 56, 112 (PBMC/Leukapharesis)

7.3.1.2.10. Humanized Mice: SCRI (20 mL)

Serum from hyperimmunized subjects will be tested for the ability to inhibit *Pf* development in liver chimeric mice (Vaughan et al-2012). The FRG huHep mouse model is a triple gene knockout (*Fah*$^{-/-}$/*Rag2*$^{-/-}$/*Il2rg*$^{-/-}$) engrafted with human hepatocytes.

Timepoints for Humanized Mice:

- CHMI: D0
- Hyperimmunized Sub-cohort:
  - re-CHMI: D0

7.3.1.2.11. Immunofluorescent antibody (IFA) assay: NMRC/WRAIR (4 mL)

The immunofluorescent antibody (IFA) assay will be used to measure *Pf* antigen-specific antibody titers. Serum will be incubated with air-dried and/or intact *Pf* sporozoites on glass slides and sporozoite-specific antibodies measured by immunofluorescence. The actual fluorescence will be measured quantitatively by image analysis software.

Timepoints for IFA:

- Imm1: D0
- All Imm: D14
- CHMI: D0, 28, 56, 112
- CHMI for Infectivity Controls: D0, 28, 56, 112
- Hyperimmunized Sub-cohort:
  - All Boosts: D0
  - Re-CHMI: D0, 28, 56, 112

7.3.1.2.12. Opsono-phagocytosis assay: WRAIR (4mL)

The opsono-phagocytosis assay will be used to measure the phagocytic activity mediated by *Pf* CSP-specific antibodies. Phagocytosis is assessed by measuring the uptake of CSP-coated beads by the human THP-1 monocytic cell line. Cells are analyzed by flow cytometry to determine the level of phagocytosis in the presence or absence of serum.

Timepoints for opsono-phagocytosis assay:

- Imm1: D0
- All Imm: D14
- CHMI: D0, 28, 56, 112
- CHMI for Infectivity Controls: D0, 28, 56, 112
- Hyperimmunized Sub-cohort:

– All Boosts: D0
– Re-CHMI: D0, 28, 56, 112

7.3.1.2.13 Proteomic serum evaluation: WRAIR (stored serum samples)

Serum will be utilized to establish a fingerprint of protective antibodies and identify key targets of the humoral immune response utilizing proteomics and mass spectrometry. Interrogation of the proteome will assist in deciphering profiles of antibodies in the protective and un-protective IMRAS sera. Further evaluation of these changes and antibody profiles will occur with the isolation of schizonts in HC04 human liver cells exposed to pooled protective/un-protective IMRAS sera. Analysis of infectivity rates, along with quantitative measures will be used to identify subset of proteins yielding changes in abundance across time points that relate to protection.

Time points for Proteomic analysis:

- Day 0 PreImm
- Day 14 Post Imm1
- Day 42 Post Imm2
- Day 70 Post Imm3
- Day 98 Post Imm4
- Day 133 Post Imm5
- Day 140 CHMI
- Post CHMI Day 28
- Post CHMI Day 56
- Post CHMI day 112

7.3.1.3. Cell Mediated Immunity (CMI) Response Assays

7.3.1.3.1. Cytokine FluoroSpot: NMRC (17 mL)

The FluoroSpot assay is based on ELISpot but utilizes fluorescent-based detection systems, enabling the detection of cells secreting either different cytokines, or combinations thereof, in the same cell. This assay will be used to simultaneously measure factors including but not limited to Th1 (IFN-γ, interleukin (IL)-2 and TNFα) and Th2 (IL-5 and IL-13) cytokines in response to 15-mer peptide pools spanning whole *Pf* antigens. Other antigenic stimulants that may be used include: APCs prepared thought the use of recombinant vectors expressing malaria antigens; recombinant proteins; whole *Pf* sporozoites; and defined HLA-restricted peptide pools. Both fresh and cryopreserved PBMCs will be assayed at various timepoints following *Pf*RAS immunization and challenge.

Timepoints for FluoroSpot:

- All Imm: D0; Imm1: D7; CHMI: D0, 42 (20 mL fresh assays – limited number of Ag)
- Leukapheresis samples (expanded number of Ag):

- Pre-Imm Leukapheresis
- Imm3: D14 Leukapheresis
- CHMI1: D5/6, 112 Leukapheresis[f]
- Boost 1: D7 Leukapheresis
- Boost 3: D7 Leukapheresis
- CHMI2: D112 Leukapheresis

- Hyperimmunized Sub-cohort:
  - All Boosts: D0
  - Re-CHMI: D0, 42

7.3.1.3.2. Ex vivo Flow Cytometry: SCRI (17 mL)

To identify unique patterns of T and B cell phenotypes with differential activation and memory status, PBMCs will be examined at early timepoints after immunization. T cells will be characterized for their activation status using the activation markers CD38, HLA-DR, Ki-67, Bcl-2, and CD57. A separate panel will be used to define memory populations using CD45RO, CCR7, CD27, and CD28. To identify antibody secreting cells and memory B cells, PBMCs will be examined using the markers CD19, CD20, CD27, CD38, and CD21. The phenotype of regulatory T cells, follicular helper T cells, and natural killer cells will also be studied at these timepoints.

Timepoints for Ex vivo Flow Cytometry:

- Imm1 & 3: D0, 3, 7, 14
- Imm5: D0, 7
- Hyperimmunized Sub-cohort:
  - Boost 1: D0, 3, 7
  - Boost 3: D7

7.3.1.3.3. Functional Flow Cytometry: SCRI (25.5 mL)/NMRC (Leukapheresis)

Intracellular cytokine staining, CFSE dye proliferation assays and multi-parameter flow cytometry will be used to measure the cytokine secretion patterns, proliferative, and cytotoxic capacity of *Pf*RAS specific CD4+ and CD8+ T cells in response to stimulation with parasite forms, including but not limited to whole *Pf* sporozoites. PBMCs will be isolated and cryopreserved 14 days after each immunization and stored until assayed.

Timepoints for Functional Flow Cytometry:

- Imm1: D0
- Imm1, 3 & 5: D14

- CHMI: D42, 56, 112
- Hyperimmunized Sub-cohort:
  - Boost 1: D0
  - All Boosts: D14
  - Re-CHMI: D0, 42, 56, 112
- Leukapheresis samples:
  - Pre-Imm Leukapheresis
  - Imm3: D14 Leukapheresis
  - CHMI1: D5/6, 112 Leukapheresis[g]
  - Boost 1: D7 Leukapheresis
  - Boost 3: D7 Leukapheresis
  - CHMI2: D112 Leukapheresis

7.3.1.3.4. Memory Phenotype Flow Cytometry: NMRC/WRAIR/SCRI

Detailed analysis of phenotypic, function and breadth of antigen-specific T cell responses will be performed on cryopreserved PBMCs obtained from the leukapheresis samples. Malaria specific T cells will be identified by parasite stimulation, including but not limited to whole P*f* sporozoites, and/or specific T cell epitopes will be detected by stimulating PBMCs with pools of overlapping 15-mer peptides to pre-erythrocytic antigens and pools stimulating a response by flow cytometry will be de-convoluted to identify the peptides expressing the stimulatory epitopes. Fluorescently labeled class I and class II tetramers expressing pre-erythrocytic epitopes will be used to identify and characterize ex vivo antigen-specific cells without the need for stimulation in vitro. Malaria antigen-specific tetramer+ T cells will be isolated using a high-speed Fluoresence-Activated Cell Sorter (FACS) Aria, and then the cells will be examined for their expression of the transcription factors Bcl-6, T-bet, Blimp-1, and Eomes by quantitative real-time PCR analyses. Only individuals with detectable responses by peptide stimulation will be selected for this extended analysis.

Timepoints for Memory Phenotype Flow Cytometry:

- Leukapheresis samples:
  - Pre-Imm Leukapheresis
  - Imm3: D14 Leukapheresis
  - CHMI1: D5/6, 112 Leukapheresis
  - Boost 1: D7 Leukapheresis
  - Boost 3: D7 Leukapheresis
  - CHMI2: D112 Leukapheresis

7.3.1.3.5. Transcriptional and epigenetic analysis of memory T cells: SCRI/U.Washington

Bulk, antigen experienced and/or antigen specific memory CD4 T cell subsets in circulation will be sorted into TRIzol and RNA isolated for RNA sequencing to identify cell-specific transcriptional signatures of durable memory after immunization. While rodent and non-human models of *Plasmodium* immunization indicate that memory CD8 T cells resident in the liver (T_{RM}s) are critical for protection, the inability to sample this tissue in healthy subjects limits the capacity to characterize liver-resident T cells in humans. However, recent evidence indicates that tissue-resident memory T cells can indeed be found in the blood after antigen re-exposure. Thus using markers of Plasmodium induced liver T_{RM}s, we will sort these previously antigen-experienced T cells into TRIzol, isolate DNA and RNA for transcriptional and epigenetic studies that will help unveil memory T cell correlates of durable immunity.

Timepoints for Transcriptional and epigenetic analysis:

- Pre-Imm Leukapheresis
- Imm1: D0, 3, 7, 14
- Imm3: D0, 3, 7, 14 (PBMC/Leukapheresis)
- Imm5: D0, 7, 14
- CHMI1: D5/6 (Leukapheresis), 42, 56, 112 (PBMC/Leukapheresis)
- Hyimm1: D0, 3, 7(PBMC/Leukapheresis),14
- Hyimm2: D7,14
- Hyimm3: D7 (Leukapheresis), D14
- CHMI2: D3, 7, 42, 56, D112 (PBMC/Leukapheresis)

7.3.1.3.6. Cytokine Profile Mesoscale: NMRC/WRAIR

The cytokine profile of antigen-specific T cell responses will be performed on cryopreserved PBMCs obtained from the leukapheresis samples. PBMC will be stimulated with peptide pools representing malaria antigens including but not limited to CSP, AMA-1, LSA-1, CelTOS and SSP2/TRAP as well as the CEF peptide pool as reference, SEB as positive control and negative control stimulation. Supernatants will be harvested 12 to 36 hr post stimulation and supernatants immediately tested by electro-chemiluminescence detection using the Mesoscale Diagnostics platform.

Timepoints for Cytokine Profile Mesoscale:

- Leukapheresis samples:
  - Pre-Imm Leukapheresis
  - Imm3: D14 Leukapheresis
  - CHMI1: D5/6, 112 Leukapheresis
  - Boost 1: D7 Leukapheresis
  - Boost 3: D7 Leukapheresis

- CHMI2: D112 Leukapheresis

7.3.1.3.7. Cultured ELISpot and Flow Cytometry: NMRC/WRAIR

Cultured ELISpot and flow cytometry will be used to assess the memory component of the immune response and T cell responses that may be present in the periphery at low frequency. Cryopreserved PBMC will be stimulated with peptides, control antigens, sporozoites and parasitized red blood cells for approximately 6-10 days. Cutured cells will be restimulated for 18 hours and IFN-γ detected by ELISpot. In addition, cultured cells will be stained for flow cytometry using several surface markers indicative of memory and terminal effector CD4 and CD8 T cell subsets in addition to cytokine secretion. These markers may include but are not limited to; CD3, CD4, CD8, CD45RO, CD62L, CD27, CD70,IL-2, IL-4, IFN-γ.

Timepoints for Cultured ELISpot and Flow Cytometry:

- Leukapheresis samples:
  - Pre-Imm Leukapheresis
  - Imm3: D14 Leukapheresis
  - CHMI1: D5/6, 112 Leukapheresis
  - Boost 1: D7 Leukapheresis
  - Boost 3: D7 Leukapheresis
  - CHMI2: D112 Leukapheresis

7.3.1.3.8. Regulatory Phenotype Flow Cytometry: USUHS

Analysis of sporozoite-specific regulatory T cell responses will be performed on cryopreserved PBMCs obtained from the leukapheresis samples. PBMCs will be stimulated overnight with *P falciparum* sporozites, using SEB as a positive control and sporozoite diluent as a negative control. Cells will be labelled with markers to identify $CD8^+$ and $CD4^+CD25^{hi}Foxp3^+$ Treg cells, as well as the more recently described $CD45RA^+Foxp3^{lo}$ naive and $CD45RA^-Foxp3^{hi}$ effector Treg subsets. Markers include: CTLA-4, a means of Treg suppressive function that has also been described on human $CCR7^+CTLA\text{-}4^+$ anergic cells; PD-1, a marker of T cell activation/exhaustion; and combinations of CD45RA, CCR7, and CD27 to define naïve, central memory and effector memory T cell subsets. Cytokines IL-10, IL-2, IFN-γ, will be sourced to the above identified T cell populations to determine how vaccination alters levels of antigen-specific cytokine expression and any correlation with protection.

Timepoints for Regulatory Phenotype Flow Cytometry:

- Leukapheresis samples:
  - Pre-Imm Leukapheresis
  - Imm3: D14 Leukapheresis
  - CHMI1: D5/6, 112 Leukapheresis
  - Boost 1: D7 Leukapheresis

- Boost 3: D7 Leukapheresis
- CHMI2: D112 Leukapheresis

7.3.1.3.9. MicroRNA: USUHS

Host microRNA (miRNA) are small single-stranded RNA molecules that control immune responses by post-translational regulation and influence immune cell development and differentiation. Analysis of microRNA released into exosomes by activated lymphocytes will be performed on cryopreserved PBMCs obtained from the leukapheresis samples. PBMCs will be stimulated for 24 hours with anti-CD3/anti-CD28 or malarial antigens, using unstimulated PBMCs as a negative control. Culture supernatants will be tested for changes in expression of select miRNAs which have been detected in response to vaccination, and/or shown to influence the adaptive immune response.

Timepoints for microRNA:

- Leukapheresis samples:
  - Pre-Imm Leukapheresis
  - Imm3: D14 Leukapheresis
  - CHMI1: D5/6, 112 Leukapheresis
  - Boost 1: D7 Leukapheresis
  - Boost 3: D7 Leukapheresis
  - CHMI2: D112 Leukapheresis
-

7.3.2. Systems Biology Approach

The immunological and transcriptional data generated from the assays described earlier will be integrated using systems biological approaches to characterize, at the transcriptional, proteomic and cellular levels, the networks and pathways activated by protective immunization regimens. It is hypothesized that a comparison of protected to non-protected subjects should make it possible to identify and validate specific immunological signatures that correlate with and predict protective efficacy.

7.3.3. Antigen Discovery

The malaria vaccine antigen pipeline is inadequate, with less than 0.2% of the *Pf* genome currently undergoing clinical testing. Most known antigens were identified ~20 years ago by screening libraries with anti-sera of through cloning of *Pf* orthologs of protective antigens from rodent malaria parasites. Moreover, with the exception of CSP, leading candidate antigens have failed to induce protection in clinical trials (LSA-1) or have induced marginally protective responses (SSP2/TRAP). Nevertheless, there is evidence that an effective malaria vaccine is feasible, based on the high-grade (> 90%) sterile immunity induced in humans or animals by immunization with *Pf*RAS (Hoffman et al-2002), GAP (Vaughan et al-2010), and native

sporozoites followed by chloroquine treatment (ITV) (Roestenberg et al-2009; 2011). This protective immunity appears to target multiple antigens, as evidenced by the identification of genetically restricted responses to pre-erythrocytic stage antigens other than CSP in *Pf*RAS-immunized subjects (Doolan et al-2003; Wizel et al-1995b) and by the fact that 100% protection can be induced by *Py*RAS in CSP-tolerized mice (Kumar et al-2006). Analysis of the cellular responses to rodent attenuated sporozoites emphasizes that a high-frequency of memory CD8+ T cells to a diversified pre-erythrocytic antigen set is required for sterile protection (Schmidt et al-2010). It should therefore be possible to identify these protective antigens, and to use them to formulate a protective pre-erythrocytic stage sub-unit vaccine.

7.3.3.1. CMI Immunoscreening: NMRC

Traditional methods will be used to identify pre-erythrocytic stage *Pf* antigens capable of recall responses from *Pf*RAS-immune CD4+ and CD8+ T cells. Cryopreserved PBMCs will be stimulated in vitro with malaria antigens including but not limited to (a) 45 *Pf* orthologs to *P yoelii* proteins previously identified to recall responses from *Py*RAS-immune mouse splenocytes and (b) 20 *Pf* antigens currently identified as promising by the PATH-MVI antigen discovery project. PBMCs will be stimulated in vitro with pools of peptides and subsequently analyzed by IFN-γ and IL-2 cultured ELISpot assay and flow cytometry for markers including but not limited to surface T cell phenotype and IL-2, TNF and IFN-γ production. These antigens will also be screened for their ability to stimulate T cells to secrete cytokines and chemokines using a 10 cytokine/chemokines multiplex Luminex assay and/or Cytokine Profile Mescoscale as described above in Section 7.3.1.3.6..

Timepoints for CMI Immunoscreening:

- Leukapheresis samples:
  - Pre-Imm Leukapheresis
  - Imm3: D14 Leukapheresis
  - CHMI1: D5/6, 112 Leukapheresis
  - Boost 1: D7 Leukapheresis
  - Boost 3: D7 Leukapheresis
  - CHMI2: D112 Leukapheresis

7.3.3.2. Serum Antibody Screen: NMRC

The same panel of 45 pre-erythrocytic *Pf* proteins will be screened by ELISA or Western blot for their reactivity to sera from *Pf*RAS-immunized subjects. Polyclonal sera will be induced in mice by protein immunization to determine sporozoite and liver stage surface expression as well as ability to inhibit sporozoite invasion (ISTI assay) and development (ILSDA).

Timepoints for Serum Antibody Screen:

- Imm1: D0
- Imm5: D14

- Hyperimmunized Sub-cohort:
  - All Boosts: D0
  - Re-CHMI: D0, 28, 56, 112

7.3.3.3. *Pf* Proteome Array: SCRI

As described in section 7.3.1.2.5 known and novel *Pf* pre-erythrocytic stage-specific proteome arrays will be used to assess plasma antibody responses before and after *Pf*RAS immunization and challenge. Multiple data points will be obtained with sera collected before and after immunizations or challenge for each antigen on the chip. This will enable statistical analyses for prioritization amongst antigens identified based on their immune reactivity, frequency, and magnitude of response.

7.3.3.4. Antigen Lead Acquisition System (ATLAS™): Genocea

The Antigen Lead Acquistion System (ATLAS™) T cell antigen discovery platform is a high throughput screening process that mimics the natural mammalian immune response to protein antigens, including antigen processing and presentation by APCs; T cell recognition of APC-displayed peptides; and immune activation using PBMCs from human donors with diverse HLA types. Genocea have built a full-length expressed protein library encompassing a significant fraction of the proteome of *Pf* (about 1,500 expressed proteins). In order to minimize the requirements for PBMC, the library is tailored to genes that have been associated with the pathogen's hepatic development stage, either through analysis of proteomic or RNA sequencing data. CD4 and CD8 T cell subsets will be purified from cryopreserved PBMC, expanded in vitro with purified autologous dendritic cells, and screened for immunoreactivity in the ATLAS™ system to identify antigenic targets for *Pf*RAS.

Timepoints for ATLAS™:

- Leukapheresis samples:
  - Pre-Imm Leukapheresis
  - Imm3: D14 Leukapheresis
  - CHMI1: D5/6, 112 Leukapheresis
  - Boost 1: D7 Leukapheresis
  - Boost 3: D7 Leukapheresis
  - CHMI2: D112 Leukapheresis

7.3.3.5. Malaria-Specific Immune Receptor Sequencing: NMRC

Identification of malaria antigen-specific immune cells will be identified by multiple approaches for the purpose of antigen receptor sequencing. Magnetic beads may be used to enrich B or T cell populations by negative selection. Peptide:HLA and/or protein tetramers representing malaria antigens of interest (including but not limited to PfCSP) will be used to identify and sort for antigen-specific T cells and/or B cells. PfCSP tetramer may be utilized to identify non-PfCSP-specific B cells, while protein tetramers for additional antigens may identify B cells

specific for novel proteins of interest. Influenza hemagglutinin (or another irrelevant protein) tetramers may be utilized as a negative control. Alternatively, PBMCs may be labeled with CFSE or a similar dye to facilitate identification of malaria-specific cells by parasite stimulation, including but not limited to whole *Pf* sporozoites, and identification of proliferating cells. Finally, use of plasmablasts enriched for reactivity against sporozoite antigens may facilitate isolation of parasite-specific B cells independent of targeted antigen, allowing selection of antigen-specific B cells based purely on parasite recognition and function, without requiring knowledge of the antigen target. Samples will be screened by staining to identify cellular subsets and analyzed by flow cytometry. Cells will be sorted and may be cultured to expand clonal populations, or stimulated with antigen to confirm antigen specificity by B cell ELISpot. Genomic sequence encoding T cell receptors (TCRs) and/or B cell receptors (BCRs) will be cloned and sequenced for further evaluation of interactions between immune cells and malaria antigens. Cell culture supernatants and/or purified antibodies may be used to identify sporozoite-reactive B cell clones by IFA, while functional assays including but not limited to ISTI and/or ILSDA may identify time points with inhibitory activity.

Timepoints for Malaria-Specific Immune Receptor Sequencing:

- Pre-immunization (Leukapheresis)
- Imm1: D0, 3, 7, 14
- Imm2: D7
- Imm3: D0, 3, 5-9, 14 (PBMC/Leukapheresis)
- Imm5: D0, 7, 14
- CHMI1: D5/6 (Leukapheresis), 42, 56, 112 (PBMC/Leukapheresis)
- Hyimm1: D0, 3, 7 (PBMC/Leukapheresis), 14
- Hyimm2: D7, 14
- Hyimm3: D7 (Leukapheresis), 14
- CHMI2: D0-3, 7-9, 42, 56, 112 (PBMC/Leukapheresis)

7.4. Measures Taken to Minimize/Avoid Bias

Immunized and mock-immunized subjects will receive bites from irradiated *An stephensi* mosquitoes either infected (true-immunization) or uninfected (mock-immunization) with *P falciparum* sporozoites. Including mock-immunized subjects should minimize any bias as a result of salivary antigens from the biting mosquitoes.

Block randomization will be used to allocate subjects to the true-immunized and mock-immunized groups.

7.5. Investigational Product

This study is classified as experimental medicine and does not target the development of a specific malaria vaccine product. The “product” studied here is radiation-attenuated *P falciparum* sporozoites administered by the bite of infected *An stephensi* mosquitoes

7.5.1. Investigational Product Preparation

7.5.1.1. Mosquito Production

Female *An stephensi* housed at the WRAIR/NMRC insectary will be used for immunizations and challenges. *An stephensi* mosquitoes can be maintained in the laboratory for up to 23 days after infection with Plasmodia parasites. *An stephensi* mosquitoes readily feed on humans and are able to effectively transmit malaria to subjects. *An stephensi* mosquitoes have been maintained at WRAIR and NMRC insectaries for several decades. The colony has been closed during this period, meaning no mosquitoes have been brought in from the outside. Anopheline mosquitoes are not known to transmit hepatitis viruses or HIV. Based on theoretical concerns of disease transmission to mosquito larva, a fish-food diet that does not contain any bovine products is used. For each immunization day (12 true-immunized subjects), approximately 4,000-6,000 mosquitoes will be infected by allowing them to feed through membranes on cultures of *P falciparum* containing a large proportion of gametocytes. Mosquitoes are kept in a secure insectary at 26ºC [78.8°F] + 5ºC with relative humidity at 75% ± 15%.

7.5.1.2. Sporozoite Production and Grading

To prepare infected mosquitoes, *P falciparum* asexual and sexual erythrocytic stage parasites are grown in normal human erythrocytes using standard culture medium containing 10%-15% normal human serum. All erythrocytes and serum are obtained from donors at low risk for both hepatitis and HIV infection and whose serum does not contain syphilis or HIV. This blood and serum for culture are purchased from a commercial source and each shipment carries a certificate of analysis certifying that the blood products were negative or non-reactive for these pathogens.

The *P falciparum* NF54 strain will be used for immunization and challenges. The 3D7 clone of NF54 may be used as a back-up in the unlikely event the NF54 strain stops functioning well. The NF54 strain of *P falciparum* is a human isolate which has never been passaged through nonhuman primates, is well adapted to culture, is a good producer of gametocytes that can infect mosquitoes; is susceptible to several currently available, licensed, antimalarial compounds, including chloroquine; and has previously been used by NMRC and WRAIR in our challenge model to successfully infect human subjects. No serious morbidity has been experienced by any subject challenged with any of these parasites under NMRC’s or WRAIR’s supervision.

All sporozoites, whether for immunization or challenge, will be administered to subjects via the bites of infected female *An stephensi* mosquitoes. Beginning approximately 14 days after membrane feeding, batches of 10 mosquitoes will be removed and dissected to look for sporozoites in their salivary glands. For immunizations and challenges, the dissected glands are scored as follows, by the number of sporozoites seen (Table 9).

Table 9: Scoring for Dissected Glands for Immunization and Challenge Procedures

Number of Salivary Sporozoites	Gland Score
1-10	1+
11-100	2+
101-1,000	3+
> 1,000	4+

7.5.1.3. Irradiation of Sporozoites

All infected mosquitoes used for immunization will be exposed to 15,000 rad (cGy) of gamma radiation using a ^{60}Co source (with a ^{137}Cs source used as back-up) prior to immunizing subjects. Based on published data and our own experience this dose is sufficient to attenuate the sporozoites, preventing the development of a patent blood-stage malaria infection, while permitting the development of a protective anti-hepatic stage immune response (Egan et al-1993; Herrington et al-1991).

7.5.1.4. Immunization Procedure

Immunization via mosquito bite is conducted through a container with a screened top. Per the applicable WRAIR Entomology SOPs and the NMRC CTC SSP 201, each container will hold 200-400 mosquitoes per container for this study. First, the container is placed in contact with the volar surface of the arm for 5 minutes. Second, a 2-minute break is given. Third, a second 5-minute feeding is conducted using the same mosquitoes on the same arm. The subject will lightly rest their forearm on the screened top and cover the arm with a towel to simulate dusk feeding conditions. The statistical likelihood is that subjects will actually be bitten by no more than 300 mosquitoes per session, about 50%-75% of which are infective. Subsequent to feeding, mosquitoes will be examined to determine the proportion having taken a blood meal, and a sample of these (approximately 30) will be dissected to determine percent infected and sporozoite gland score. Based on this procedure, subjects will receive approximately 200 immunizing bites (200-400 bites total) per session. In order to reach the target of 960 mean immunizing bites for Cohort 1, the number of mosquitoes placed in cartons for each immunization procedure will be appropriately varied to keep the group mean bite numbers on track (all subjects at each immunization session will receive the same number of mosquitoes).

In the event that the immunization procedure described above does not achieve the desired number of fed mosquitoes, a second carton of mosquitoes may be utilized. The second carton would be placed on the same location of the forearm as the first carton.

7.5.1.4.1. Immunization Safety Procedures

Subjects will be observed for at least 30 minutes after each immunization for evidence of immediate reactions to the mosquito bites. Subjects will be managed under standard of care practices in accordance with GCP guidelines throughout all phases of the study, including during the immunization period. The risk of a breakthrough infection from inadequately attenuated sporozoites is very unlikely; however, the study subjects will be alerted to this risk and monitored for signs or symptoms of breakthrough parasitemia throughout the immunization

phase of the trial. If a subject develops signs or symptoms consistent with malaria, a blood smear for microscopic examination may be collected post-immunization based upon investigator judgment. If the blood smear were found to be positive, the subject will be managed as outlined in the section 9.1.6.1, Management of Subjects Post-Challenge.

Study physicians will be available on the 24-hour emergency mobile phone for contact by study subjects. If subjects must travel post-immunization, arrangements will be made for coverage of questions, symptoms, etc.

Immunizations with large numbers of mosquitoes (eg, 200-400 actually taking a blood meal) will occur approximately every 4 to 5 weeks. In order to prevent hyper-sensitization to mosquito bites, no subject will receive more than 2,500 bites per 6-month period.

7.5.1.5. Controlled Human Malaria Infection (CHMI or Challenge)

CHMI will be conducted using 5 infected mosquito bites. Subjects will be monitored for 30 minutes immediately after challenge to ensure there are no immediate adverse reactions to challenge. Samples, including sera, plasma, and PBMCs will be taken prior to, during, and after challenge (delineated in the study events schedule) to be used to investigate the correlates of protection. Subjects will be monitored closely (section 5.7.1.2) during the challenge phase for symptoms of malaria and treated immediately upon first microscopic detection of parasitemia.

7.6. Duration of Subject Participation

The projected study period spans from the start of recruitment (estimated fall- 2013) through the final visit for Cohort 2. The projected study period spans approximately 120 weeks. Cohort 2 will be based upon a variable dosing regimen; therefore, it may range between 112 to 128 weeks.

Cohort 1: Each subject will actively participate for approximately 52 weeks (screening, immunization, pre- and post-immunization leukapheresis and follow-up).

Cohort 2: Each subject will actively participate for approximately 52 weeks with a range between 46 to 60 weeks depending upon dosing regimen.

Cohort 1 Hyperimmunity Sub-cohort: Each subject who elects to participate in the continuation phase will actively participate for approximately 120 weeks (comprising the duration of Cohort 1 immunization and challenge, the interval between completion of Cohort 1 and initiation of Cohort 2, and the duration of Cohort 2 immunization and challenge).

7.7. Dose-adjustment Criteria

7.7.1. Stopping Rules

If any of the events listed below occur, further true- and mock-immunizations will not be administered to subjects until a thorough review is completed. Immunization may resume with the concurrence of the research monitor, sponsor's representative, PI, NMRC Office of Research Administration (ORA), and the USAMRMC Human Research Protection Office (HRPO). The FDA CBER will be notified if any of the stopping rules are triggered. The IRB will be notified and will reserve the right to impose further safety restrictions:

1. An SAE in a subject thought to be possibly, probably, or definitely related to the *Pf*RAS immunization.

2. Any breakthrough infection following immunization.

3. Following immunization 1/3 or more subjects experience the same Grade 3 laboratory abnormality or the same Grade 3 systemic AE that is determined to be possibly, probably, or definitely related to the vaccine.

4. Any confirmed diagnosis of serum sickness or delayed Type IV hypersensitivity.

7.7.2. Study Termination Criteria

The principal investigator (PI), research monitor, sponsor's representative, the United States Army Medical Research and Materiel Command (USAMRMC) Office of Research Protections, Human Research Protection Office (ORP HRPO), or the FDA may stop or suspend this trial at any time.

7.8. Trial Treatment Randomization Codes

Trial treatment randomization codes are not applicable and will not be used in this protocol.

Once a subject signs the informed consent document, he/she will be assigned a subject identification number which will be formatted according to the corresponding study-specific procedure (SSP). Per the SSP, subjects will be assigned a number that will consist of the letters IMRAS followed by numbers (eg, 001, 002, etc.) in consecutive order. Numbers will not be reused.

Once the subject has been evaluated, met inclusion criteria and does not meet any exclusion criteria, he/she will be enrolled into the study. The allocation of subjects to true-immunization, mock-immunization, and challenge control categories will occur prior to the first immunization for true- and mock-immunized groups and prior to the CHMI for the infectivity control group. A stratification and block randomization method will be used for the allocation of true-immunized and mock-immunized subjects. Of note, a stratification of challenge control subjects will occur but they will not be block randomized. IMRAS Study Specific Procedure 205 delineates the process for the stratification and block randomization of subjects.

7.9. Identification of Data to be Recorded on the Case Report Forms

All data recorded at the time of visit will be considered source documentation. There will not be 2 separate source and Case Report Form (CRF) documents. Data will be recorded onto a hand-written form, which will serve as the source/CRF document. The electronic capture of study data will be transcribed from the source/CRF document. No data will be recorded directly in the electronic data capture (EDC) database system without prior written record. The transcribed data will be consistent with the source/CRF document or the discrepancies will be explained. The study and Data Management teams will prepare the source/CRFs, and the Data Management team will enter and Quality Control (QC) the data. For more information on data handling, refer to section 16.

8. Selection and Withdrawal of Subjects

8.1. Recruitment of Subjects

Adult men and women, civilian and active duty military subjects will be recruited from the general population in the Baltimore-Washington D.C. area, by use of advertisements in multiple media formats to include, but not limited to: informational flyers; large media formats to include newspaper, Metro, bus advertisements and social media (eg, Facebook, NMRC website, etc.); word of mouth and e-mail. All recruitment materials will be prepared and submitted for review and approval by the NMRC IRB prior to use. The large media formats require high-definition/high-quality images in order to maintain integrity and quality. For this reason, the IRB approved recruitment materials used in the large media formats may be used without the IRB approval stamp in order to maintain the high resolution quality necessary for these large media accounts. However, all of the information that appears on the IRB approval stamp will appear on all recruitment materials regardless of resolution. The information may appear in text format, but it must include all of the following information: NAVY MEDICINE HRPP; HRPP NMRC.2013.007; Approval Date; Expiration Date; and Verification by ORA Staff.

When a subject calls the CTC and discloses an interest in the study, the recruitment staff will discuss the trial from an IRB-approved script. If the subject is still interested, contact information will be obtained and an appointment for briefing and/or screening will be arranged. Active duty military subjects will require approval from their supervisor which will be documented using the Statement of Supervisor's Approval. As outlined in section 5.9, up to 200 subjects will be recruited in order to enroll 52 subjects in this study.

8.1.1. Referral Fee

A referral fee of $25 will be given to enrolled subjects who successfully recruit a subject. A subject will be considered a "successful recruit" if they meet all eligibility criteria and complete the entire screening visit. Each enrolled subject will be limited to four (4) successful recruits, for a maximum additional compensation of $100.

8.2. Informed Consent Process

All subjects will undergo an informed consent process consisting of a detailed informational presentation of the study given using IRB approved briefing slides. The briefing will be given either by an investigator or by means of a pre-recorded audio recording. This process will take place at NMRC CTC. Following the briefing given by a study investigator, the coordinator or designee will provide the subject an ample time to read the informed consent document (Appendix A). The study investigator and/or designee will answer all questions raised during the session. The subject will be asked to sign the consent. The subject will be allowed to take the consent document home to consider and discuss it with others and return to the CTC at a later time to sign it. After signing the consent, the subject will take an Assessment of Understanding Test. The test is administered to aid the study personnel in identifying gaps in understanding. Subjects must score at least 70% correct on the 10-question multiple-choice test. Any questions answered incorrectly will be explained to the subject, and the subject's questions will be answered. The subject will be given one additional opportunity to take the comprehension test. Any subject who, in the opinion of the study investigator, does not understand the study well

enough to consider their consent truly informed will be excluded. A passing score of 70% will be required to participate in the study.

No study procedures will occur before the potential subject gives informed consent. To minimize any possible coercion, supervisors or anyone in the chain of command or in a position of authority will not be present during the consenting process. However, they will be available to answer questions and provide additional information. If the subject has additional concerns, the research monitor will be available to answer medical questions and the NMRC Office of Research Administration is available to answer any human use related concerns.

Consent for Future Use: As part of the informed consent process, volunteers will be asked to provide permission for the future use of their specimens.

Consent for Base Access Screening: As part of the informed consent process, volunteers may be asked to provide personally identifiable information in order to gain access to Department of Defense (DoD) installations; individuals without a valid DoD identification card or a common access card will be asked to complete a vetting process through Naval Support Activity Bethesda and the Fort Detrick Forest Glen Annex. This process includes completing SECNAV Form 5512/1, which will then be submitted to the Pass and ID office of both installations. The vetting process will search databases that identify persons with a history of criminal activity and may result in denial of access to military installations. Study participants requesting access on these installations should be prepared to provide, at a minimum, at least one form of picture identification and the following personally identifiable information: full name, date of birth and social security number. Additional forms of identification or personally identifiable information may be required. Once vetted, participants will be required to resubmit forms every 90 days. In the event that base access requirements change, study subjects will be required to follow the new DoD requirements for base access.

8.3. Eligibility Screening

Subjects who have successfully completed the Assessment of Understanding and who have signed an informed consent form will provide a medical history and undergo a physical examination and routine laboratory screening tests. See screening section 9.1.1.

8.4. Subject Inclusion/Exclusion Criteria

8.4.1. Subject Inclusion Criteria

Subjects must meet all of the following criteria to be included in the study:

- Healthy adults (male or nonpregnant, nonbreastfeeding female) 18-50 years of age (inclusive).
- Available and willing to participate for duration of study.
- Able and willing to provide written informed consent.
- Able to complete an Assessment of Understanding with a score of at least 70% correct.
- In good general health with no clinically significant health problems as established by medical history, physical exam, and laboratory screening.

- Females of childbearing potential must have a negative pregnancy test at screening agree to not become pregnant or breastfeed for the duration of the study. She must be willing to use a reliable form of contraception during the study.
    - Reliable forms of birth control include use of condoms, diaphragm or cervical cap, birth control pills, IUD or sperm killing products.
- Agree to refrain from blood donation (except as required in this study) for 3 years following *P falciparum* challenge.
- Agree not to travel to a malaria endemic region during the study.
- Good peripheral venous access.

8.4.2. Subject Exclusion Criteria

Subjects meeting any of the following criteria will be excluded from the study:

- Positive HIV, HBsAg, or HCV serology.
- Positive sickle cell screening test, including evidence of sickle trait.
- Reactivity by CSP or AMA1 ELISpot assay or ELISA as determined by IMRAS Study Specific Procedure #204.
- Anemia (below normal reference laboratory value of hemoglobin) on screening.
- Weight less than 110 pounds (this does not apply to infectivity controls as it is a weight cut-off for subjects undergoing leukapheresis procedure)
- Any history of malaria infection or travel to a malaria endemic region within 6 months prior to first immunization.
- History of long-term residence (> 5 years) in area known to have significant transmission of *Pf* [cumulative lifetime exposure].
- Use of systemic immunosuppressant pharmacotherapy for greater than 10 days within 60 days of scheduled first immunization (inhaled and topical steroids are allowed; short duration or tapered corticosteroid regimens of 10 days or less that have been discontinued prior to first immunization are allowed).
- Current significant medical condition (cardiovascular, hepatic, renal, pulmonary, or hematological) or evidence of any other serious underlying medical condition identified by medical history, physical examination, or laboratory examination (includes bleeding disorders).
- Plan for surgery between enrollment and day 28 post-challenge (minor procedures, elective corrective vision surgery, and dental procedures are allowed).
- Receipt of immunoglobulin and/or any blood products within 90 days of scheduled leukapheresis or immunization.
- Has evidence of increased cardiovascular disease risk (defined as > 5%-10%, 5-year risk) as determined by the method of Gaziano (2008). Risk factors include sex, age

(years), systolic blood pressure (mm Hg), smoking status, body mass index (BMI, kg/m^2), reported diabetes status, and blood pressure.

- An abnormal electrocardiogram (ECG), defined as one showing pathologic Q waves and significant ST-T wave changes; left ventricular hypertrophy; any non-sinus rhythm excluding isolated premature atrial contractions; right or left bundle branch block; or advanced (secondary or tertiary) A-V heart block.
- History of a splenectomy.
- History of any other illness or condition that, in the investigator's judgment, may substantially increase the risk associated with the subject's participation in the protocol or compromise the scientific objectives. This may include psychiatric disorders (such as personality disorders, anxiety disorders, or schizophrenia) or behavioral tendencies (including active alcohol or drug abuse) discovered during the screening process that in the opinion of the investigator would make compliance with the protocol difficult.
- History of anaphylactic or severe response to mosquito bites, retinal or visual field changes, or known allergy to the antimalarial chloroquine phosphate, which will be used to treat subjects developing malaria after CHMI.
- Participation in any study involving any investigational vaccine or drug within 30 days prior to the screening visit, or plan to participate in another investigational vaccine/drug research during or within 1 month following participation in this study.
- Use or planned use of any drug with antimalarial activity that would coincide with immunization or challenge.
- History of psoriasis or porphyria, which may be exacerbated after treatment with chloroquine.
- Anticipated use of medications known to cause drug reactions with chloroquine or atovaquone-proguanil (Malarone) such as cimetidine, metoclopramide, antacids, and kaolin during the day 7 to 28 post-challenge period.
- Any other significant findings which, in the investigator's judgment, may substantially increase the risk associated with the subject's participation in the study or compromise the scientific objectives.

8.5. Subject Withdrawal Criteria

Any subject may withdraw consent at any time during the study without penalty. Counseling about the subject's health will be provided if he/she decides to discontinue participation in the study. Medical advice regarding what is in the best interest of the subject will be provided.

The PI may discontinue the subject's activity without the subject's consent if any of the following criteria is met:

- Failure to comply with study procedures.
- A subject's safety or health may be compromised by further participation.

- The scientific integrity of the study may be compromised by further participation.
- The subject engages in criminal activity or displays inappropriate behavior that may compromise the health, safety, or well-being of study staff or other research subjects.

8.5.1. When and How to Withdraw Subjects

If a subject withdraws, the investigator will make a reasonable effort to determine the reason for the withdrawal from the study and to complete termination procedures including safety follow-ups that are needed to assure the study subject's health (ie, the subject will be asked to complete the same safety visits as scheduled for the most recent applicable study events: leukapheresis, immunization, or challenge). Telephone calls, registered letters, email correspondence, and the use of social media tools are considered reasonable efforts. For subjects leaving the study, a targeted examination may be performed, if medically indicated and if permitted by the subject.

A subject may be withdrawn for an adverse event (AE) or serious adverse event (SAE) resulting in a safety concern or for noncompliance with protocol requirements. When a subject withdraws due to an AE or is withdrawn by the principal investigator due to an AE, the sponsor's safety office, the USAMRMC Clinical Services Support Division (CSSD), Product Safety Surveillance Branch (PSSB) must be notified within 72 hours (usarmy.detrick.medcom-usammda.mbx.sae-reporting@mail.mil). Investigators must follow specific policy at each institution regarding the timely reporting of AEs and SAEs to the NMRC IRB (section 11.6.1). In all cases, the PI will make a reasonable effort to complete study termination procedures.

Women who become pregnant during the study will be encouraged to seek obstetric care and will be asked to provide follow-up information at the conclusion of the pregnancy.

If a subject meets withdrawal conditions for a concomitant medication violation or noncompliance, this will be stated in the source document and the study termination forms.

Withdrawing from the study for any reason will not impact the subject's medical care.

8.5.2. Data Collected for Withdrawn Subjects

All data collected up to the time of withdrawal will be reported. The study forms will be completed, with the reason for withdrawal specified.

8.5.3. Withdrawal of Active Duty Military Subjects

In the event an active duty military subject is required to deploy or participate in temporary duty that prevents continuation of this study, participation in this clinical trial will not be allowed to impede those requirements. The member will simply be withdrawn from the study with provision for adequate safety follow-up and data collected up until that time will be used. Safety follow-up will be coordinated through the member's command and/or deployment unit and the NMRC clinical trials team. Depending on the timing of the deployment, the study subject may need empirical treatment with chloroquine (for example, if immediate deployment is required after receiving the mosquito bites associated with CHMI).

During the malaria challenge in this study, an active duty member may have their work performance temporarily affected if they contract malaria; however, this possibility will not affect their overall ability to deploy. All subjects are closely monitored and treated at the first

sign of parasitemia which only requires 48 hours of medical therapy. Recovery is uniformly rapid and complete.

8.5.4. Replacement of Subjects

At least 2 alternates will be recruited for each cohort and they will undergo pre-immunization leukapheresis. These alternates will be asked to be present on the day of first immunization. If an assigned subject does not present on the first day of immunization, elects to withdraw, or is found to have met an exclusion criteria, an alternate will be enrolled into the study. An alternate may also replace a subject who withdraws or is withdrawn from the study by the PI within a 7 day window post- first immunization only.

At least 2 alternates will be recruited for the challenge controls and will be asked to be present on the day of challenge. If an assigned subject does not present on the day of challenge, elects to withdraw, or is found to have met an exclusion criteria, an alternate will be enrolled.

Non-immunized alternates for Cohort 1 may elect to participate as Cohort 1 infectivity controls or elect to undergo re-screening and consideration for participation within Cohort 2 (if pre-immunization leukapheresis was successfully completed for Cohort 1, it does not have to be repeated for Cohort 2).

8.5.5. Follow-up for Withdrawn Subjects

If a subject withdraws, the investigator will make a reasonable effort to determine the reason for the subject’s withdrawal from the study and to complete termination procedures. Withdrawing from the study for any reason will not impact the subject’s medical care. Subjects who withdraw or are withdrawn will be followed, as allowed by the subject, through resolution of any on-going adverse events. Women who become pregnant during the active phase of the trial will be encouraged to seek obstetric care and will be asked to provide follow-up information at the conclusion of the pregnancy.

8.5.6. Subjects Who Miss Immunization Doses

Once immunization has began, study subjects will not be replaced. In order to maintain the scientific integrity of this study with a small sample size, subjects who remain eligible to participate in the study and miss up to two immunization dose(s) may be allowed to complete the immunization series and CHMI. Safety information collected from each subject who receives at least one dose of immunization can contribute to the general body of knowledge related to the safety of the study product. Moreover, clinical samples from subjects who receive at least two immunization would be analyzed for the presence of CD8 epitopes (personal communication, Dr. Martha Sedegah, Malaria Immunologist).

9. Study Procedures

9.1. Study Visit Schedule and Follow-up Periods

Subjects will actively participate for approximately 52 weeks (screening, leukapheresis, immunization, challenge, and follow-up). Evaluation of the study vaccine will include laboratory studies, medical history, and physical assessment by clinicians following immunization. The complete schedule of study visits, permitted windows for completing the visits, and evaluations performed at each visit is provided in a tabular format in Appendix C**.** There will be a specifically defined “window” for each study visit (allowable days before or after), although subjects will be encouraged to be present on the targeted study visit day. The blood volume drawn from each subject will not exceed 525 mL per 8-week period (per guidelines of the American Association of Blood Banks).

9.1.1. Screening Visit

Screening procedures, which will be conducted at the NMRC CTC, may occur during more than one visit. The screening visit(s) for subjects in the true-immunized and mock-immunized groups will be scheduled between 90 and 7 days prior to the receipt of the first immunization session where radiation-attenuated *Pf* sporozoites are administered by the bite of infected *An stephensi* mosquitoes. For infectivity controls, the screening visit will entail the same procedures and will be scheduled 90 to 3 days before CHMI. Fully informed written consent must be obtained from each subject prior to conducting any study procedure. Two consent forms will be signed: a consent form for study participation and a separate consent for HIV testing. Subjects may take the informed consent documents home for review or to discuss with family or friends. To assure understanding, a comprehension assessment will be administered. Once consent forms are signed, a unique identification number will be assigned to each study participant.

The following procedures will be carried out during the screening visit(s):

1. Slide Presentation of clinical trial design, risks, and study schedule.
2. Review of informed consent document and discussion with study investigator and or designee.
3. Signature of informed consent document and other research-related documents (HIV consent for non-active duty, Health Insurance Portability Accountability Act (HIPAA), registration). HIV consent is not required for active duty study subjects.
4. Assessment of Understanding (minimum of 70% required for participation and one repeat testing if necessary).
5. Vital Signs (temperature, pulse, blood pressure, and respiratory rate).
6. Height and weight measurements.
7. Medical History.
8. Analysis of 5-year cardiovascular risk: This will be performed based upon the method of Gaziano et al (2008). The risk factors will include sex, age, body mass index (BMI), blood pressure, history of diabetes mellitus, and history of smoking.

9. Physical examination: examination of general appearance, skin, neck (including thyroid), eyes, nose, throat, lungs, heart, abdomen, lymph nodes, extremities, and a basic neurological assessment.
10. ECG: A baseline 12 lead ECG will be obtained on all subjects as part of the screening process. The ECG(s) will be reviewed by a board-certified cardiologist; the results (normal, normal with variant, or abnormal) will be emailed back to the NMRC CTC study staff.
11. Laboratory screening: Standard clinical laboratory tests for the purpose of inclusion and exclusion of potential subjects and for safety monitoring will be performed at the clinical laboratory at WRNMMC. Laboratory studies may be conducted at other times during the course of the trial if the investigators judge it necessary for the safety of the participant. The following laboratories will be performed at screening:
    - Hematology: complete blood count (CBC) with differential
    - Serum chemistry: glucose, creatinine, alanine aminotransferase (ALT), aspartate aminotransferase (AST), total bilirubin, alkaline phosphatase, calcium, BUN
    - Coagulation profile: prothrombin time (PT), partial thromboplastin time (PTT); international normalized ratio (INR)
    - Sickle cell test (to exclude those with sickle cell trait)
    - Screening serologies: HBsAg, anti-HCV. Anti-HIV-1 (ELISA) will be performed and if positive, confirmed by Western blot (consent will be obtained prior to evaluation of HIV serology)
    - Urinalysis
    - G6PD (for clinical awareness; not an exclusion criteria)
    - Pregnancy test (Urine ß-HCG): pregnancy tests will be required of all female subjects regardless of age or reported sterilization, unless there is written proof of sterilization or the subject is menopausal defined as appropriate age with 1 year of amenorrhea. Urine pregnancy tests will be performed on site.

All screening laboratories will be considered expired if performed more than 90 days prior to immunization. In the event that the screening laboratories are expired, CBC with differential, glucose, creatinine, BUN, ALT, AST, alkaline phosphatase, total bilirubin, calcium, PT, PTT, INR, Anti-HIV-1, HBsAg, anti-HCV, and urine pregnancy test will be repeated (G6PD, sickle cell test and urinalysis will not be repeated) and results will be reviewed prior to the first leukapheresis procedure for those in the true-immunized and mock-immunized groups and prior to the challenge for the infectivity controls.

In addition to the clinical laboratory screening tests, research immunoassay screening will be conducted at NMRC to ensure that research subjects have not been previously exposed to malaria.

- CSP or AMA1 ELISpot assay and/or CSP or AMA1 ELISA

Subjects who meet all inclusion criteria and none of the exclusion criteria, sign the Informed Consent Document, and pass the Assessment of Understanding will be enrolled in the study. Subjects excluded from this study because of significant abnormalities will be managed initially by study clinicians and referred to outside care for evaluation as necessary. In the event of a positive HIV test, the subject will be referred for appropriate counseling and follow-up care. Notification of state and federal authorities, as required by law, will be the responsibility of the PI. For members of the military, notification of command will also be the responsibility of the PI as required by military regulations.

9.1.2. Pre-Leukapheresis Visit

Subjects will have a study visit within 7 days prior to the first leukapheresis procedure to confirm that they may proceed to their first leukapheresis. The following will occur:

- Study education provided: study schedule, contact card (who to contact and how to contact the research site)
- Medical history update and directed physical exam
- Vital signs (temperature, blood pressure, pulse, respiratory rate)
- HLA typing: A buccal swab will be collected for HLA typing. HLA typing is needed for the conduct of certain assays assessing antigen-specific, HLA-class restricted cellular responses. If any subjects are found to have HLA types associated with increased risk of an autoimmune disease, the subject may be referred for further counseling but would not be excluded from the study.
- Laboratories: CBC with differential

The PI or clinical investigator will review the results and will determine if the leukapheresis procedure can proceed. Results of the hemoglobin and platelet count must fall within specific limits as described subsequently. The subject will be notified of the lab result; if acceptable, the subject may be formally enrolled into the study at that time.

9.1.2.1. Hemoglobin

Two factors are important considerations for the safety of leukapheresis: hemoglobin levels and the hemodynamic challenge associated with temporarily removing blood to an extracorporeal site. Regarding the former, the impact on packed cell volume is minimal – leukapheresis is generally counted as removing the equivalent of 20 mL of whole blood. The hemodynamic challenge associated with extracorporeal circulation is also minimal in a healthy adult. To assure hemodynamic stability, subjects will be examined for postural hypotension prior to any leukapheresis procedure.

A normal hemoglobin value per reference laboratory (Walter Reed National Military Medical Center (WRNMMC)) will be used as a screening criterion for leukapheresis eligibility at the start of the study. This value is 12.8 g/dL for men and 11.5 g/dL for women. It is important that the study subject begin the trial with a normal hemoglobin, because hemoglobin levels tend to fall in association with the large volumes of blood taken for research purposes during a study. For this reason, once the study is initiated, lower levels of hemoglobin (eg, Grade 1 abnormality) will be acceptable to proceed with subsequent leukapheresis procedures. This value will be 12.5 g/dL for

men and 11.0 g/dL for women. These criteria have been reached following detailed discussion with our expert consultant, Dr. Cantilena from the National Institutes of Health (NIH) Therapeutic Apheresis Center and the grading criteria are consistent with the FDA’s Toxicity Grading Scale for Healthy Adult and Adolescent Volunteers Enrolled in Preventive Vaccine Clinical Trials. The precedent for apheresing research subjects below American Association of Blood Banks (AABB) guidelines (12.5 g/dL for both males and females) is well established. For example, HIV positive patients are often apheresed at the NIH with values as low as 9.0 g/dL. As stated previously, due to exposure to malaria, research subjects in this clinical trial will be ineligible for blood bank donations, including apheresis, for 3 years following CHMI.

9.1.2.2. Platelets

Subjects must have a normal platelet count per reference laboratory in order to proceed with leukapheresis. There will be no changes in the platelet cut-off for leukapheresis for subsequent leukapheresis procedures.

Due to the time-sensitive nature of the immune response and in recognition of the risks taken by research subjects who have received an experimental immunization with *Pf*RAS, every effort will be made to collect leukapheresis samples. The scientific contribution of the research subject is lost if the blood samples are not taken.

Therefore, if the hemoglobin or platelet values are below accepted criteria (below Grade 1 values for hemoglobin and below normal value for platelets), these labs may be repeated. A new safety laboratory sample will be collected in this situation for analysis. In the event that the repeated laboratory values are within acceptable criteria, the leukapheresis procedure may proceed as scheduled. In the event that the repeated laboratory values fall outside the acceptable range, the safety laboratories may be repeated every 12 hours per the judgment of the PI.

9.1.3. Leukapheresis Visit

Leukapheresis procedures will be performed at the CTC. Subjects will undergo leukapheresis before first immunization (all enrolled subjects), after the third immunization (optional, no more than 50% of immunized subjects), approximately 5-6 days post-challenge of immunized subjects and at approximately 4–6 months post-challenge (optional, no limit to how many immunized subjects), to collect large numbers of PBMCs.

Prior to the initiation of leukapheresis:

- The subject will be asked to sign a separate consent specifically for the leukapheresis procedure.
- The subject will be identified by name, date of birth, and study subject number.
- An interim medical history will be taken and a directed physical examination will be conducted.
- The results of the pre-leukapheresis CBC will be confirmed.
- Vital signs will be taken, deemed normal prior to initiation of the procedure, and subsequently checked or as needed for the duration of the procedure; this will include examination for postural hypotension.

- Results of pregnancy test (Urine ß-HCG) will be confirmed. Leukapheresis will not proceed unless a negative pregnancy test has been obtained for female subjects.

Based upon the information obtained from the preceding procedures, the PI or clinical investigators will determine whether the subject may proceed to leukapheresis. The procedure for leukapheresis can be found in section 10, including a discussion of citrate anti-coagulation and the temporary induction of hypocalcemia.

9.1.4. Immunization Visit

All immunizations will be administered according to the assigned cohort schedule. 12 subjects will be assigned to a "true"-immunized group and 4 subjects will be assigned to "mock"-immunized group. Immunization will be conducted at WRAIR insectary, Silver Spring, Maryland. Subjects will be transported by a designated NMRC staff to WRAIR insectary for the conduct of immunization. The procedure for immunization can be found in section 7.5.1.4.

The following will occur prior to each immunization:

- Vital signs will be taken.
- Medical history update and directed physical examination will be performed.
- Inclusion and exclusion criteria will be reviewed.
- Results of pregnancy test (Urine ß-HCG) will be confirmed. Immunization will not proceed unless a negative pregnancy test has been obtained on the day of immunization.
- The following laboratory tests will be collected:
  - Safety: CBC with differential, BUN, creatinine, ALT, AST, alkaline phosphatase, total bilirubin
  - Immunological Assays: Refer to Table 8 of Section 7.3.1

A post-immunization assessment will be completed at 30-60 minutes after each immunization. All subjects must be cleared by a study clinician to leave the study site following an immunization procedure. This will allow the study team to confirm that there are no immediate reactions in response to immunization and to provide immediate care if any reactions do occur. Symptomatic treatment, such as topical anti-inflammatory medication, will be provided as necessary.

Although breakthrough infections are not expected, subjects will be counseled about symptoms and signs of malaria and reminded how to contact study staff if needed at any time.

9.1.5. Follow-Up Visits Post-Immunization

Subjects will be asked to come to the CTC for post-immunization follow-up visits. At each visit, a study physician will evaluate the subjects. Vital signs including pulse, blood pressure, respiratory rate, and temperature will be noted. A directed physical examination will be conducted. Signs and symptoms will be solicited on scheduled visit days from subjects through Day 7 post-immunization and recorded by the investigators. Subjects will be asked to report on unsolicited adverse events through Day 14 post-immunization. Additionally, other clinical

concerns may prompt a study visit based on the judgment of a study clinician. Clinical laboratory assays and clinical evaluations will assess safety at 3 and 7 days after each immunization. At intervals throughout post-immunization, subjects will also have blood drawn for the following research assays (refer to Appendix C):

- Safety Laboratories (Days 3 and 7 after each immunization): CBC with differential, BUN, creatinine, AST, ALT, alkaline phosphatase, and total bilirubin
- Immunological Assays: Refer to Table 8 of Section 7.3.1.
- Blood smear for microscopic examination may be collected post-immunization based upon investigator judgment.

Optional Visits:

- **Post-immunizations 1 and 5:** Subjects will be asked to come back to the CTC 3-8 hours after their first and fifth immunizations. During this visit, blood will be collected for research assays.
- **Post-immunization 3. Leukapheresis procedure:** An optional visit to perform leukapheresis procedure will be done approximately 14 days after third immunization. This optional visit will be limited to a maximum of 50% of both the true-immunized and mock-immunized groups. For women of childbearing potential, leukapheresis may not proceed unless a negative pregnancy test has been obtained.

9.1.6. Challenge Visits

Challenge with viable, infectious sporozoites will be conducted at WRAIR Insectary, Silver Spring, Maryland. The interval between last immunization and challenge for a given subject is 21 days (± 7 days). For infectivity controls, Day 0 is defined as day of challenge. The cohort of study subjects will be split into 2 subcohorts, with each subcohort receiving a separate challenge separated by approximately 2 days from the challenge date for the other subcohort. If possible, each subcohort will be challenged by mosquitoes drawn from the same batches, with mosquitoes removed for the first challenge and then additional mosquitoes removed from the same cages two days later for the second challenge. These mosquitoes will have been infected with malaria via membrane feed on cultures of *P falciparum* 17 to 23 days prior to challenge.

Subjects will be asked NOT to wear cologne, perfume, or aftershave on the day of challenge or use scented soaps in the 24 hours prior to challenge as it may discourage mosquito feeding. Subjects will be transported by a designated NMRC staff to WRAIR insectary for the conduct of challenge.

The following will occur prior to CHMI:

- Vital signs will be taken.
- Medical history update and directed physical examination will be performed.
- Results of pregnancy test (Urine ß-HCG) will be confirmed. Immunization will not proceed unless a negative pregnancy test has been obtained.
- The following laboratory tests will be collected:

- Safety: CBC with differential, BUN, creatinine, ALT, AST, alkaline phosphatase, and total bilirubin
- Immunological Assays: Refer to Table 8 of Section 7.3.1

9.1.6.1. Management of Subjects Post-Challenge:

A post-challenge assessment will be completed at 30-60 minutes to assure that there are no systemic allergic reactions or other untoward events. Subjects with signs or symptoms related to the challenge will be managed appropriately by a clinical investigator. Symptomatic treatment, such as topical anti-inflammatory medication, will be provided as necessary. Subjects will be encouraged to contact study staff at any time if they experience symptoms secondary to the mosquito bites.

9.1.6.1.1. Instruction about Symptoms of Malaria

Before completing the challenge day, subjects will be instructed on the symptoms of malaria [fever (oral temperature of > 38ºC [100.4°F]), chills, rigors (shaking chills), sweats, headache, dizziness, malaise, fatigue, insomnia, joint pain, muscle pain, neck ache, nausea, vomiting, stomach/abdominal cramps, diarrhea]. Because there is good reason to assume that approximately 50% of the true-immunized subjects and all mock-immunized and infectivity control subjects will develop blood-stage malaria, it is critical that careful and systematic monitoring be provided. However, it is not possible for symptomatic blood stage malaria to develop prior to day 6 or 7 following challenge (day 6 has actually never been observed) since the parasites remain in the liver for at least 120 hours. In addition, subjects will be counseled to use methods that will reduce risk of exposure to mosquitoes beginning 5 days after challenge until 28 days post-challenge (if remaining without parasitemia) or until 2 consecutive daily negative smears after treatment for parasitemia. This counseling will be provided in order to minimize the risk that research subjects might transmit malaria to local Anopheles mosquito populations. However, individuals developing *P falciparum* malaria do not generate gametocytes until several days after the infection becomes patent, making transmission extremely unlikely even in the absence of precautions against mosquito bites.

9.1.6.1.2. Provision of Notification Card

Subjects will also be given a “Notification Card” and instructed to keep this card with them until they have either been fully treated for malaria or, in the event that they do not develop malaria, until the completion of the 28 day post-challenge follow-up period. In the event that a subject requires urgent medical care (for example, following a motor vehicle accident), this card will provide an alert to medical staff, that the subject has been challenged with mosquitoes infected with *P falciparum.* It will also include the date of challenge and a contact number for study staff.

9.1.6.2. Post-Challenge Follow-Up Visits

9.1.6.2.1. 1 Day Post-challenge

All subjects who received CHMI will be seen at the CTC 1 day after challenge. Vital signs, medical history update, and physical exam will be performed. They will be asked about adverse

events and concomitant medication taken since the day of challenge. Blood will be collected for research assays.

9.1.6.2.2. Post-challenge Leukapheresis Procedure

Approximately 5-6 days after challenge, leukapheresis will be performed. It is not planned that infectivity controls will be leukapheresed; however in the event that an infectivity control was leukapheresed as an alternate, a post-challenge leukapheresis may be performed. Clinical investigators will review vital signs, medical history, and laboratory results and perform a directed physical examination prior to leukapheresis procedure. For women of childbearing potential, leukapheresis may not proceed unless a negative pregnancy test has been obtained. No safety laboratory tests are planned on the leukapheresis day. The CBC for checking on the adequacy of hemoglobin and platelets will have been performed on day of challenge.

9.1.6.3. Overnight Stays (Day 7-18)

Pre-patent periods are generally within 9-14 days post challenge although can be as early as 6 days and as late as 21 days (Church et al, 1997; Epstein et al-2007). To facilitate daily close monitoring of clinical status and blood smears from Days 7 through 18 post-challenge, subjects who received challenge will be required to have up to 11 overnight stays. During this time, subjects will be free to go about their normal daily activities but must return to the hotel in the evenings. Each morning, subjects will be evaluated by the study team. Vital signs, medical history update, and directed physical exam will be performed. A study physician will be available for consultation 24 hours per day. During the history and physical examination by a study physician, subjects will be asked about signs and symptoms of malaria and a blood sample for malaria diagnosis will be collected. Signs and symptoms consistent with typical clinical malaria signs and symptoms (eg, fevers and chills) will be collected and documented as expected signs and symptoms of malaria rather than as AEs. Symptomatic study subjects may have blood smears collected as frequently as every 6-8 hours or at any time post-challenge based upon the clinical judgment of the investigator.

Microscopic blood smear examination will be performed in real time according to a consensus SOP developed a World Health Organization Committee developed for use in malaria clinical trials. Should the malaria blood smear be read as positive, the study subject will be called and asked to see a study investigator for treatment. Blood smear collection will end following documentation of cure, once 2 consecutive daily negative blood smears have been recorded, and if the PI or clinical investigator deems the subject clinically ready for discharge, study subjects can check out of the hotel, and will be asked to return to the CTC on Day 28 post-challenge. At the time each blood smear is collected, a separate blood sample will be collected and stored for retrospective PCR analysis.

In addition, the following laboratories will be collected:

- **Safety Laboratories:** CBC with differential, BUN, creatinine, AST ALT, alkaline phosphatase, and total bilirubin will be collected on:
  - At the onset of parasitemia/ or with the following morning sample
  - Approximately 72 hours after onset of parasitemia

- 28 days post-challenge
- Clinical laboratories may be drawn at any other timepoints post-challenge when deemed clinically indicated by the investigators

- **Immunological Assays**: Refer to Table 8 of Section 7.3.1

9.1.6.4. Days 20, 22, 25, and 28 Post-challenge

An immunized individual who does not have complete protection may have a prolongation of the pre-patent period post challenge. Although this has not previously been observed, in theory it could be longer than 21 days. For this reason, immunized subjects who are still negative 18 days post-challenge will be followed on Days 20, 22, 25 and 28 after challenge. However, subjects will be permitted to leave the hotel on day 18 and will be seen at the CTC on Days 20, 22, 25 and 28. During these visits, the following will occur:

- Vital signs, medical history update, and directed physical exam
- Blood smear and PCR sample
- CBC with differential, BUN, ALT, AST, alkaline phosphatase, total bilirubin, and creatinine (these hematology and chemistry tests only on Day 28 post-challenge or if clinically indicated)

9.1.6.5. Treatment of Malaria for Parasitemic Subjects

Subjects who develop blood-stage malaria will be treated with a standard dose of chloroquine (a total of 1,500 mg chloroquine base given orally in divided doses: 600 mg initially, followed by 300 mg given approximately 6, 24, and 48 hours later). This will be done by directly observed treatment (DOT), meaning that a study team member, either physician or nurse, will witness the swallowing of the chloroquine dose. In the case that a subject is allergic to, or unable to tolerate chloroquine, he/she will be treated with Malarone (250 mg atovaquone/100 mg proguanil tablets) 4 tablets once per day for 3 days as a second line therapy, or other appropriate antimalarial drugs or drug combinations such as Coartem (artemether/lumefantrine) as a third line therapy. There are additional highly effective treatments available as well, including mefloquine or quinine/doxycycline. Subjects will be reminded if the potential side effects of the antimalarial treatment. All subjects exposed to malaria will be unable to donate blood for 3 years.

On development of symptoms, provided there are no contraindications, subjects may be given acetaminophen (325 to 650 mg every 4 to 6 hours or 1,000 mg every 6 to 8 hours) or ibuprofen (400 mg orally every 6 hours as needed). In case of vomiting, particularly if it interferes with the administration of antimalarial medication, subjects will be treated with prochlorperazine. Antimalarial medication administration will be repeated once if vomiting occurs within 60 minutes of initial administration. If vomiting occurs twice, an alternative antimalarial treatment will be selected (see above).

9.1.6.6. Long-term Follow-up Visits

9.1.6.6.1. Day 42 Post-challenge

Vital signs including pulse, blood pressure, respiratory rate, and temperature will be noted. A directed physical examination will be conducted. Blood for research assays will be collected. (Refer to Table 8 of Section 7.3.1.)

9.1.6.6.2. 2 Months Post-challenge

All subjects will be seen at the CTC on Day 56 after challenge. Vital signs including pulse, blood pressure, respiratory rate, and temperature will be noted. A directed physical examination will be conducted. Any AEs or SAEs that are unresolved at that time will be continued to be followed until resolution, or, if a chronic condition has developed, until it has stabilized. The following laboratories will be collected:

- **Safety laboratories:** CBC with differential, BUN, creatinine, ALT, AST, alkaline phosphatase, and total bilirubin
- **Immunology assays**: Refer to Table 8 in section 7.3.1

9.1.6.6.3. 4-6 Months Post-challenge (Final Visit)

Subjects will have a medical history update and direct physical exam performed. They will be asked about adverse events and concomitant medications. An optional leukapheresis will be offered to all true-immunized and mock-immunized subjects. Leukapheresis will not be performed on subjects who are infectivity controls. All procedures and criteria prior to leukapheresis procedure will be followed as described in section 9.1.2. The following laboratories will be collected:

- **Safety laboratories:** CBC with differential, BUN, creatinine, ALT, AST, alkaline phosphatase, and total bilirubin
- **Immunology assays**: Refer to Table 8 in section 7.3.1

All subjects will be instructed to contact the study team any time, within a year post-challenge should they experience fever. If subjects leave the study, prior to the passage of 1 year post-challenge and move outside of the Washington, DC area, they will be instructed to inform their medical care providers that they have been exposed to malaria and must have a blood smear checked whenever they develop a fever. In addition, they should request that their medical care providers contact the study physicians for advice concerning the diagnosis and treatment of malaria infection. If they leave the study prior to the passage of 1 year post-challenge but remain in the area, they will be instructed to contact the study team whenever they have a fever within 1 year after a challenge so that a blood smear can be checked and be treated as needed. These instructions are precautionary. In fact, there has never been a recrudescence of the NF54 or 3D7 strains of *P falciparum* malaria following CHMI at the WRAIR insectary once the study subject has received primary treatment with chloroquine.

9.1.6.6.4. 6-month and 12-month Phone Call Post-Challenge

Subjects will be contacted by the NMRC Staff at approximately 6 months and 12 months after the final challenge (CHMI). For the Hyperimmunized Sub-Cohort, these phone calls will also occur after the secondary challenge.

9.1.6.6.5. Cohort 1 Hyperimmunized Sub-cohort (Continuation Phase)

After the protection status of Cohort 1 is known, an optional hyperimmunization continuation phase will be offered to protected subjects. A maximum of 6 protected subjects from Cohort 1 will comprise the Cohort 1 Hyperimmunization Sub-cohort. It is estimated that the interval between the challenge of Cohort 1 and the initiation of Cohort 2 will be approximately 3 months; however, it is possible that this interval may be longer. Therefore, until the initiation of Cohort 2, the Cohort 1 Hyperimmunization Sub-cohort will follow the study schedule for Cohort 1.

Upon the initiation of Cohort 2, the continuation phase subjects will begin following their own study schedule. Of note, the immunizations, safety laboratories, and follow-up visits will coincide with Cohort 2's first 3 immunizations and study schedule. Additionally, leukapheresis procedures will be performed following the first and third secondary immunizations.

The secondary challenge for Cohort 1 Hyperimmunzation Sub-cohort will coincide with the primary challenge for Cohort 2. As Cohort 2 is designed with an adjustable dosing regimen, the interval between the third secondary immunization and the secondary challenge may be 3-19 weeks.

- **Safety Laboratories (Days 3 and 7 after each secondary immunization):** CBC with differential, BUN, creatinine, ALT, AST, alkaline phosphatase, and total bilirubin
- **Safety Laboratories post secondary CHMI:** CBC with differential, BUN, creatinine, ALT, AST, alkaline phosphatase, and total bilirubin will be collected on:
  - At the onset of parasitemia
  - Approximately 72 hours after onset of parasitemia
  - 28 days post-challenge
  - Clinical laboratories may be drawn at any other timepoints post-challenge when deemed clinically indicated by the investigators
- **Immunology assays**: Refer to Table 8 in section 7.3.1

9.2. Unscheduled Visit

Unscheduled visits, requested by the subject or the study physician, will prompt a medical history update, physical examination, vital signs, clinically indicated laboratory tests, documentation of any AEs, and/or any other medically indicated diagnostic or therapeutic procedures.

9.3. Concomitant Medications

This protocol places no restrictions on the use of concomitant medications with the exception of the immunomodulators as detailed in the exclusion criteria, drugs with anti-malarial properties (eg, doxycycline, clindamycin, azithromycin) and drugs that might interfere with anti-malarials during the treatment phase post-challenge in those subjects who become parasitemic. Any pre-existing conditions that require routine or intermittent medications should be discussed at the time of screening and a study physician will determine if participation is safe and will not interfere with any data being collected. If participation is permitted, the concomitant medication(s) will be recorded.

Any new medications required during the course of participation in the study must be discussed with the study team to ensure both safety of the subject and integrity of the data being collected. Information regarding all over-the-counter and/or prescription medications taken will be solicited and recorded at each scheduled study visit.

The investigator will recommend medication for symptomatic relief, if necessary. This will not affect the data collected from subjects who take medication during the trial.

9.4. Procedures for Monitoring Subject Compliance

All immunizations, leukapheresis procedures, and challenge will be conducted under the direct supervision of the investigational staff. Subject failure to attend scheduled study visits (within specified windows) will be recorded as protocol deviations.

If a subject fails to attend a scheduled study visit, the PI or his or her designee will make a reasonable effort to determine the reason for the attendance failure. Telephone calls, registered letters, email correspondence, and the use of social media tools are considered reasonable efforts. If a study subject is deemed to be unreliable for future study visits, he or she may be withdrawn from the study based upon the judgment of the PI. For subjects being withdrawn from the study, a targeted examination may be performed, if medically indicated and if permitted by the subject.

10. Leukapheresis Procedure

Several PBMC collections will be performed via leukapheresis, a standard procedure for the collection of blood products. It will be performed on site at the NMRC CTC and will be supervised, staffed, and performed by trained personnel from Baltimore RH Typing Laboratory. Baltimore RH Typing Laboratory is a medical apheresis service that has provided apheresis services for the last 21 years in the Pennsylvania, Maryland, Virginia, and Washington, DC areas. The company is staffed with 3 full-time and 2 per diem Registered Nurses. The Medical Director is Cathy Conry-Cantilena, MD. She is concurrently the Medical Director and Senior Staff Physician of the Therapeutic Apheresis Center (TAC), NIH Department of Transfusion Medicine. The TAC serves 14 hospitals in the region and has performed procedures on over 2,900 patients and/or subjects. The actual procedure will be performed at the NMRC CTC by the Baltimore RH Typing Laboratory staff, given their long-standing expertise in this procedure. The nurses performing the procedure will be required to undergo license verification by WRNMMC. NMRC CTC staff, including the PI or a clinical investigator who is a qualified clinician, will be on site during every procedure.

All leukapheresis procedures will be performed using a 2-armed approach via an apheresis device such as the Cobe Spectra – blood exiting via 1 arm (needle inserted into antecubital vein) and returning via the other. Subjects will be specifically consented for the leukapheresis process. The volume processed will be 2 blood volumes, approximately 10-12 liters. In most subjects this will result in collection of a volume of 200-300 mL of plasma mixed with citrate and 1×10^9 WBCs per liter processed with a target PBMC total content in the bag of $> 7.5 \times 10^8$. The lost plasma volume is replaced with normal saline solution. There is a net loss of packed hemoglobin equivalent to approximately 20 mL of whole blood. Anticoagulation will be accomplished using acid citrate dextrose (ACD-A). Subjects will be monitored for signs or symptoms consistent with hypocalcemia secondary to the use of ACD-A (see section 10.1). Hypocalcemia during leukapheresis may be controlled by slowing the flow rate and/or by the administration of oral calcium, detailed in SOP 416 entitled “Apheresis”.

Upon completion of leukapheresis procedure, the collection bag will be labeled with the subject’s ID number and given directly to designated study personnel. Typically, 2 to 4 hours are required to process 10-12 liters of blood. The final amount of PBMCs collected will depend on each subject’s unique physiology and the length of the leukapheresis procedure. The number of total PBMCs collected is a small fraction of the total number of whole-body WBCs and they are quickly replaced from the bone marrow and other internal organs. The removal of these peripheral blood WBCs has not been reported to have negative impacts on health (Sandler and Nusbacher-1982).

10.1. Leukapheresis Procedure Risks and Common Side Effects

As with blood sampling, leukapheresis carries a risk of minor discomfort, minor bruising at the site of the needle puncture, and rarely the possibility of infection at the needle puncture site. Side effects of this procedure include: fatigue, nausea, dizziness, decreased blood pressure, feeling cold, perioral paresthesias, phalangeal paresthesias, urticaria, muscle cramps, and coagulation changes. These latter side effects thought to be related to the citrate anti-coagulant used in the apheresis procedure. The anticoagulant chelates calcium ions and can routinely lead to symptomatic hypocalcemia (Weinstein-2011).

A typical leukapheresis procedure will remove approximately 20 mL of whole blood as all blood components other than peripheral leukocytes are returned to the subject. The value of 20 mL per normal leukapheresis procedure will be incorporated into the maximum allowable withdrawal limit of 525 mL of whole blood every 8 weeks.

In the unexpected event of an apheresis device malfunction, a maximum amount of whole blood withdrawn from the subject would be approximately 284 mL (approximately 1 pint). If this occurs, the study subject's hemoglobin will be measured the next day and a specific plan for limiting upcoming trial-related blood drawing for that individual will be determined in consultation with the research monitor and after review with the study subject. This is necessary because a loss of 284 mL would likely place the study subject's total blood loss above the 525 mL limit per 8-week period (depending on which leukapheresis procedure was involved). However, device failure (such as would result from a power outage) is a very unusual event.

10.2. Apheresis Rationale

Leukapheresis is necessary to collect a sufficient supply of PBMCs in order to meet the study objectives; however, the large quantity of leukocytes removed could potentially affect the malaria-specific immune response and thus the level of protection. This is especially true if leukapheresis were performed at the time of challenge, the timepoint that best reflects the immune response encountered by the malaria parasites following their administration during CHMI. This concern is based on the unpublished clinical trial of *Pf*RAS immunization conducted at NMRC in 2000-2002 in which all 10 challenged subjects underwent leukapheresis prior to challenge; there is a possibility that this leukapheresis depleted the immune response at a critical timepoint, resulting in only 50% protection compared to the 93% seen in earlier studies. In other words, although leukapheresis does not significantly affect the white blood cell count, since removed cells are rapidly replenished, the cells removed could potentially be a large proportion of the malaria-specific lymphocytes, while the replacement cells would not have this specificity.

To avoid the potential of affecting the immune response prior to challenge, leukapheresis will be performed 5 to 6 days after challenge, at which point immune-targeting of developing liver-stage parasites should have already occurred. Presumably, leukapheresis can then be performed without the risk of affecting challenge results. However, it is possible, even likely, that the immune profile measured days 5 to 6 post-challenge will already have changed from the immune profile at the time of challenge. For this reason, we will also take a large whole blood sample prior to challenge (black arrow, Figure 4). While this one pre-challenge sample will remove so few lymphocytes that there is scant possibility that it could affect protection, it will accurately represent the state of the host immune responses at the time of challenge (as measured in the periphery). These very limited PBMCs will be used to confirm major findings from the more extensive assessments that will be performed on the large apheresis samples collected 5 to 6 days post challenge. Assessments not requiring a large quantity of blood can be performed directly on the pre-challenge sample without waiting for the results on leukapheresis samples.

11. Safety Assessment

11.1. Safety Monitoring

Safety monitoring will occur to assess adverse events associated with immunization and CHMI. The primary source of adverse events associated with immunization is reaction to mosquito salivary gland antigens. Most individuals tolerate the bites well, but some may need to be withdrawn from the study because of reactions. Decisions about need to withdraw a subject will be based on the PI’s clinical judgment.

11.1.1. Study Safety Management

The research monitor and PI will review any safety concerns. In his/her official capacity, the research monitor will utilize consultant physicians as necessary throughout the study.

11.1.2. Research Monitor

The research monitor will function as an independent safety advocate for subjects per DoD Directive 3216.02. An independent research monitor is required to review all unanticipated problems involving risk to subjects or others including SAEs occurring during the execution of the protocol and provide an unbiased written report of the event. The research monitor should comment on the outcomes of the event or problem and, in the case of a SAE, comment on the relationship to participation in the study. The research monitor should also indicate whether he/she concurs with the details of the report provided by the study investigator. Reports for events determined by either the investigator or research monitor to be possibly, probably or definitely related to participation and reports of all SAEs should be promptly forwarded to the IRB, ORP HRPO, and USAMRMC CSSD PSSB (sponsor safety office).

Additionally, the research monitor may perform oversight functions (eg, observe recruitment, enrollment procedures, and the consent process for individuals; oversee study interventions and interactions; review monitoring plans and unanticipated problems involving risk to subjects or others (UPIRTSO) reports; and oversee data matching, data collection, and analysis) and report their observations and findings to the IRB or a designated official.

The research monitor may discuss the research protocol with the investigators, interview human subjects, and consult with others outside of the study about the research. The research monitor shall have authority to stop a research protocol in progress, remove individual human subjects from a research protocol, and take whatever steps are necessary to protect the safety and well-being of human subjects until the IRB can assess the monitor’s report. Research monitors shall have the responsibility to promptly report their observations and findings to the IRB or other designated official.

11.1.3. USAMMDA Clinical Services Support Division

Clinical Services Support Division is responsible for coordinating and integrating the review of safety data regarding products sponsored by The Surgeon General-Department of the Army (TSG-DA). The PSSB reviews each SAE report for medical consistency, accuracy, and completeness and follows each event until it is satisfactorily resolved. The Director, CSSD has overall responsibility for assuring the functioning of the Data Review Committee.

11.2. Specification of Safety Endpoints

See section 7.1.

11.3. IND Safety Reporting

The following terms, as defined by 21 CFR 312.32, apply to IND safety reporting.

11.3.1. Adverse Event

Adverse event means any untoward medical occurrence associated with the use of a drug in humans, whether or not considered drug related.

Suspected adverse reaction means any adverse event for which there is a reasonable possibility that the drug caused the adverse event. For the purposes of IND safety reporting, “reasonable possibility” means there is evidence to suggest a causal relationship between the drug and the adverse event. Suspected adverse reaction implies a lesser degree of certainty about causality than adverse reaction, which means any adverse event caused by a drug.

11.3.2. Solicited Adverse Event

A solicited AE is a predetermined event, identified in the Investigator’s Brochure and by previous experience with the investigational product or procedures, which may reflect safety concerns related to the investigational product or procedures. The solicited AEs for this study include:

> Solicited Local Adverse Events: pain, tenderness, erythema, swelling, induration, localized pruritus, lymphatic streaking, axillary adenopathy.
>
> Solicited Systemic Adverse Events: Fever (oral temperature ≥ 100.4 F), chills, headache, fatigue, nausea, vomiting, diarrhea, malaise, myalgia, arthralgia, systemic allergic type reactions (systemic pruritus, localized urticaria, systemic urticaria or angioedema, and anaphylaxis).

See Appendix D for Intensity Grading Scale for Solicited AEs.

11.3.3. Serious Adverse Event

An adverse event or suspected adverse reaction is considered “serious” if, in the view of either the investigator or sponsor, it results in any of the following outcomes:

- Death
- Life-threatening adverse event
- Inpatient hospitalization or prolongation of existing hospitalization
- Persistent or significant incapacity or substantial disruption of the ability to conduct normal life functions
- Congenital anomaly/birth defect

An adverse event or suspected adverse reaction is considered “life-threatening” if, in the view of either the investigator or sponsor, its occurrence places the patient or subject at immediate risk of death. It does not include an adverse event or suspected adverse reaction that, had it occurred in a more severe form, might have caused death.

Important medical events that may not result in death, be life-threatening, or require hospitalization may be considered serious when, based upon appropriate medical judgment, they may jeopardize the patient or subject and may require medical or surgical intervention to prevent one of the outcomes listed in this definition. Examples of such medical events include allergic bronchospasm requiring intensive treatment in an emergency room or at home, blood dyscrasias or convulsions that do not result in inpatient hospitalization, or the development of drug dependency or drug abuse.

11.3.4. Unexpected Adverse Event

An adverse event or suspected adverse reaction is considered “unexpected” if it is not listed in the investigator’s brochure or is not listed at the specificity or severity that has been observed; or is not consistent with the risk information described in the clinical protocol. For example, under this definition, hepatic necrosis would be unexpected (by virtue of greater severity) if the investigator’s brochure referred only to elevated hepatic enzymes or hepatitis. Similarly, cerebral thromboembolism and cerebral vasculitis would be unexpected (by virtue of greater specificity) if the investigator’s brochure listed only cerebral vascular accidents. “Unexpected,” as used in this definition, also refers to adverse events or suspected adverse reactions that are mentioned in the investigator’s brochure as occurring with a class of drugs or as anticipated from the pharmacological properties of the drug, but are not specifically mentioned as occurring with the particular drug under investigation.

11.3.5. Other Adverse Event

Other adverse events, referred to in this protocol as unsolicited AEs, will be identified by investigators during the evaluation of safety data. After reviewing solicited AEs, the subject will be asked a non-leading question such as: “Have you experienced any other symptoms” or "Have you noticed any changes to your health or felt different in any way since receiving the vaccine or since the previous visit?"

Significant adverse events of particular clinical importance, other than SAEs and those AEs leading to discontinuation of the subject from the study, will be classified as other adverse events. For each, a narrative may be written and included in the clinical study report.

11.4. Relationship to Investigational Product

The investigator must assign a relationship of each AE to the receipt of the investigational product. The investigator will use clinical judgment in conjunction with the assessment of a plausible biologic mechanism, a temporal relationship between the onset of the event in relation to receipt of the investigational product, and identification of possible alternate etiologies including underlying disease, concurrent illness or concomitant medications. The following guidelines should be used by investigators to assess the relationship of an AE to study product administration. ONLY A PHYSICIAN CAN MAKE THIS DETERMINATION.

Not related: No relationship to study product. Applies to those events for which definitive evidence exists that there is an alternate etiology.

Unlikely: Likely unrelated to the investigational product. Likely to be related to factors other than study product, but cannot be ruled out with certainty.

Possible: An association between the event and the administration of investigational product cannot be ruled out. There is a reasonable temporal association, but there is also an alternative etiology such as the subject's clinical status or underlying factors including other therapy that appears to be a more likely explanation.

Probable: There is a reasonable degree of certainty that a relationship to the investigational product exists, including a reasonable temporal association. However, there are alternative etiologies related to the subject's clinical state or factors including other therapy that could possibly also explain the findings, even though the investigational product is a more likely cause.

Definite: An association exists between the receipt of investigational product and the event. An association to other factors has been ruled out.

11.5. Recording Adverse Events

11.5.1. Methods/Timing for Assessing, Recording, and Analyzing Safety Endpoints

AEs will be documented in the source records/CRFs and recorded in the EDC System using accepted medical terms and/or the diagnoses that accurately characterize the event. When a diagnosis is known, the AE term recorded in the database will be the diagnosis rather than a constellation of symptoms. The investigator will assess all AEs for seriousness, relationship to investigational product, severity, and other possible etiologies. When an event has not resolved by study closure, it will be documented on the AE datasheet as "ongoing".

11.5.1.1. Following *Pf*RAS Immunization

Solicited AEs will be assessed through day 7 after immunization; unsolicited AEs will be assessed through day 14; occurrence of laboratory abnormalities will be assessed through day 7 after immunization. SAEs will be recorded from first immunization to the end of the trial.

11.5.1.2. Following CHMI

Unsolicited signs and symptoms will be collected through Day 7 post-challenge. Solicited local signs and symptoms will be collected through Day 7. Post-challenge and solicited systemic signs and symptoms will be collected through Day 6.

Starting at Day 7 post-challenge, subjects will be monitored for signs and symptoms consistent with malaria infection. These will be documented but will not be categorized as adverse events related to immunization. Safety laboratories will be collected on day of challenge, at the time of first detection of parasitemia, approximately 72 hours later, and on Day 28 post-challenge.

11.5.2. Duration of Follow-Up of Subjects after Adverse Events

Investigators are required to follow AEs and SAEs to resolution, even if this extends beyond the prescribed reporting period. Resolution is the return to baseline status or stabilization of the

condition with the probability that it will become chronic. The SAE outcomes will be reported to the sponsor's representative using the Serious Adverse Event Report Form.

Investigators are not obligated to actively seek SAEs in former subjects; however, if an SAE, considered to be related to the investigational product is brought to the attention of the investigator at any time following completion of the study, the event will be reported to the sponsor's safety office as defined in section 11.6.1.1.

11.5.3. Severity Assessment

All AEs will be assessed for severity by the investigator. Inherent in this assessment is the medical and clinical consideration of all information surrounding the event including any medical intervention required. Each event will be assigned one of the following categories: mild, moderate, severe, or life-threatening. Refer to the grading scale in Appendix D for further guidance in the assignment of severity. The criteria below may be used for any symptom not included in the grading scale. Any grade 4 (life-threatening) AE must be reported as an SAE.

The CRF for AEs will reflect only the highest severity for continuous days an event occurred.

**Mild**	Grade 1	Does not interfere with routine activities<br>Minimal level of discomfort
**Moderate**	Grade 2	Interferes with routine activities<br>Moderate level of discomfort
**Severe**	Grade 3	Unable to perform routine activities<br>Significant level of discomfort
**Potentially life-threatening**	Grade 4	Hospitalization for potentially life-threatening event

FDA guidelines for toxicity will be followed; however, if a subject is evaluated in an emergency room for non-life-threatening illness or symptoms (ie, visits emergency department on weekend for mild problems because the physician's office is closed), the information from that visit will be reviewed and severity of the adverse event will be assessed according to the subject's clinical signs and symptoms.

As defined by the ICH guideline for GCP, the term "severe" is often used to describe intensity (severity) of a specific event (as in mild, moderate, or severe myocardial infarction); the event itself however, may be of relatively minor medical significance (such as severe headache). This is not the same as "serious", which is based on subject/event outcome or action criteria usually associated with events that pose a threat to a subject's life or functioning. Seriousness (not severity) serves as a guide for defining regulatory reporting obligations.

Solicited AEs, both local and systemic; vital signs; and laboratory abnormalities specific for this study along with the intensity grading scales to be used to grade AEs are listed in Appendix D.

11.6. Reporting Adverse Events

The PI will report all AEs to the sponsor's safety office (USAMRMC CSSD PSSB) and the local IRB in the appropriate safety, annual, and/or final reports. After appropriate data cleaning and query resolution between the clinical site, sponsor's clinical monitor, and clinical data manager, SAEs from the clinical database will be reconciled with the sponsor's SAE database. SAEs and

AEs for inclusion in annual and final reports to the FDA will be provided from the clinical database by the clinical data manager in the CSSD.

11.6.1. Reporting Serious and Unexpected Adverse Events

SAEs will be reported immediately to the research monitor and to the NMRC IRB and HSRRB (within 24 hours). The following information will be provided via e-mail or telephone (refer to Table 11):

a. NMRC IRB protocol number, HRPO Log number, investigational product, investigator name and contact number
b. Subject identification number, initials
c. SAE, onset date, date of investigational product administration, severity, relationship, and subject's current status
d. Concomitant Medication CRF or a list of concomitant medications
e. Medical record progress notes including pertinent laboratory/diagnostic test results
f. e-mail: usarmy.detrick.medcom-usammda.mbx.sae-reporting@mail.mil

When submitting SAE reports via e-mail, the subject line will read as follows:

SAFETY REPORT – Protocol Log # A-____, Subject# ____, Event term

Notification of Additional Immediately Reportable Events

The investigator must report the following events *immediately* **(within 24 hours of identification)** by e-mail or fax to the NMRC IRB:

a. Any withdrawal of consent during the study due to study procedure related-adverse events.
b. Pregnancy or intent to become pregnant*

   *Subjects who become pregnant any time after the first immunization will be followed for duration of the pregnancy to ensure the safety of the mother and child. A pregnancy should be followed to term for outcome, including date of delivery, health status of the mother and child including the child's gender, height, and weight. Complications and or abnormalities should be reported including any premature terminations. A pregnancy is reported as an AE or SAE only when there is suspicion that the investigational product may have interfered with the effectiveness of contraception or there was a serious complication in the pregnancy including a spontaneous abortion or an elective termination for medical rationale.

11.6.1.1. Reporting to the Sponsor

All unexpected AEs and all SAEs must be reported promptly (within 72 hours) to the sponsor's representative as per 21 CFR 312.64, whether or not the event is considered related to study product. All notifications will be provided to the sponsor's safety office, the CSSD PSSB, USAMMDA, and USAMRMC. Further, the investigator should comply with relevant study site SOPs on reporting SAEs.

The information that the investigator will provide to the USAMRMC CSSD PSSB is specified in Table 11. The Sponsor's representative may request additional information for purposes of the study.

Table 10: Study Contacts for Reporting Serious Adverse Events

**Sponsor's Safety Office**	U.S. Army Medical Research & Materiel Command<br>ATTN: MCMR-UMR<br>1430 Veterans Drive<br>Fort Detrick, MD 21702-5009<br>Fax: 301-619-0197<br>Telephone: 301-619-0317<br>Email: **usarmy.detrick.medcom-usammda.mbx.sae-reporting@mail.mil**
**Naval Medical Research Center Office of Research Administration Institutional Review Board**	NMRC Office of Research Administration<br>503 Robert Grant Avenue<br>Silver Spring, MD 20910<br>Telephone: 301-319-7276<br>Fax: 301-319-7277
**USAMRMC Office of Research Protections**<br>**Human Research Protection Office**	US Army Medical Research and Materiel Command,<br>ATTN: MCMR-RPH<br>504 Scott Street<br>Fort Detrick, Maryland 21702-5012<br>Fax: 301-619-7803<br>Telephone: 301-619-7550<br>Email: usarmy.detrick.medcom-usamrmc.other.hrpo@mail.mil
**Research Monitor**	Janine Danko, MD, MPH, CDR, MC, USN<br>Walter Reed Army National Military Medical Center (WRNMMC)<br>8901 Wisconsin Avenue<br>Bethesda, MD 20889<br>301-295-8279<br>Fax: 301-295-8025<br>Email: **janine.r.danko.mil@mail.mil**

Table 11: SAE Information to be Reported to the Sponsor's Safety Office

Notification Method	Information to be Provided
**Email or Telephone (within 72 hours)**	IND Number: TBD<br>Sponsor Study Number: S-12-22<br>Investigational Product Short Name: Radiation-attenuated *Plasmodium falciparum* sporozoites<br>PI: Eileen D. Franke Villasante, PhD<br>PI: Contact Number: 301-319-2076<br><br>Subject identification number<br>SAE, onset date, date of investigational product administration, severity, relationship, and subject's current status
**AND**<br>**Email or Fax**	Cover sheet or letter<br>Adverse event CRF<br>SAE CRF<br>Concomitant medication CRF or a list of concomitant medications<br>Medical record progress notes including pertinent laboratory/diagnostic test results
NOTE: When submitting SAE reports via email, the subject line of each email notification will read as follows: **SAFETY REPORT – IND # _____, Sponsor Study #_____, Subject# _____, Event term: _____**	

In order to comply with regulations mandating sponsor notification of specified SAEs to the FDA within 7 calendar days, investigators must submit additional information as soon as it is available. The sponsor's representative will report unexpected SAEs associated with the use of the investigational product to the FDA as specified at 21 CFR 312.32 (c).

Investigators must follow all relevant regulatory requirements as well as specific policy at each institution regarding the timely reporting of SAEs to the local IRB, research monitor, and the USAMRMC ORP HRPO.

Reporting to the sponsor's representative does not fulfill the investigator's duty to report all unanticipated problems involving risk to human subjects or others to the IRB. The PI will notify the NMRC IRB, the WRAIR Human Research Protection Branch, the ORP HRPO, and the research monitor.

11.6.1.2. Reporting to the IRB

Unanticipated problems involving significant risk to subjects or others, SAEs related to participation in the study, and all subject deaths should be reported by phone, email, or fax to the NMRC Institutional Review Board (IRB) and the WRAIR HSPB within 24 hours of occurrence.

Investigators are required to forward safety information provided by the sponsor's representative to the IRB.

11.6.1.3. Reporting to ORP HRPO

Unanticipated problems involving significant risk to subjects or others, SAEs related to participation in the study, and all subject deaths related to participation in the study should be promptly reported by telephone, email, or fax to the US Army Medical Research and Materiel Command Office of Research Protections, Human Research Protection Office. A complete written report should follow the initial notification.

11.6.2. Reporting Additional Immediately Reportable Events to the Sponsor's Representative and ORP HRPO

11.6.2.1. Pregnancy

Each pregnancy must be reported immediately (**within 72 hours of identification**) by email or fax to the sponsor's safety office (CSSD PSSB) and the ORP HRPO. Subjects who become pregnant after Day 0 will be followed to term, and the following information will be gathered for outcome, date of delivery, health status of the mother and child including the child's gender, height and weight.

Complications and/or abnormalities should be reported including any premature terminations. A pregnancy is reported as an AE or SAE only when there is suspicion that the investigational product may have interfered with the effectiveness of contraception or there was a serious complication in the pregnancy including a spontaneous abortion or an elective termination for medical rationale.

11.6.2.2. AE-related Withdrawal of Consent

Any AE-related withdrawal of consent during the study must be reported *immediately* **(within 72 hours of identification)** by email or fax to the sponsor's representative and the ORP HRPO.

11.6.2.3. Pending Inspections/Issuance of Reports

The knowledge of any pending compliance inspection/visit by the FDA, Office for Human Research Protections (Department of Health and Human Services), or other government agency concerning clinical investigation or research, the issuance of Inspection Reports, FDA Form 483, warning letters, or actions taken by any regulatory agency including legal or medical actions and any instances of serious or continuing noncompliance with the regulations or requirements will be reported immediately to USAMRMC ORP HRPO and the sponsor's representative.

11.6.3. IND Annual Report to the FDA

The PI will be responsible for the preparation of a detailed annual synopsis of clinical activity, including adverse events, for submission to the sponsor's representative (USAMMDA). Each annual report will summarize IND activity for 1 year beginning approximately 3 months before the IND FDA anniversary date. The sponsor's representative will notify the PI of the due date with sufficient time for the PI to assemble the required information.

11.6.4. Periodic Safety Reporting

The PI will report all AEs to the sponsor's representative (USAMRMC Division of Regulated Activities and Compliance) and the local IRB in the appropriate safety, annual, and/or final reports. The study site will provide personal computer (PC) transfer files to the sponsor's representative for preparation of annual and final reports to the FDA.

11.6.5. Final Study Report

A final study report will be prepared in accordance with "Guidance for Industry: Submission of Abbreviated Reports and Synopses in Support of Marketing Applications" and ICH E3 Guideline "Structure and Content of Clinical Study Reports" and provided to the sponsor's representative for review and approval. The sponsor's representative will use this report to prepare the final clinical study report for submission to the FDA.

The principal investigator will report all AEs to the sponsor's safety office (USAMRMC CSSD PSSB) and the local IRB in the appropriate safety, annual, and/or final reports. After appropriate data cleaning and query resolution between the clinical site, sponsor's clinical monitor, and clinical data manager, SAEs from the clinical database will be reconciled with the sponsor's SAE database. SAEs and AEs for inclusion in annual and final reports to the FDA will be provided from the clinical database by the clinical data manager in the CSSD.

12. Statistics

12.1. Description of Statistical Method

All analyses will be performed based on a statistical analysis plan (SAP) agreed to by all parties prior to closing the database (final database lock). The SAP is a document separate from the protocol. This is a small Phase 1 study, so it is largely descriptive and is intended to provide initial safety, reactogenicity, immunogenicity, and efficacy of the candidate malaria vaccine. The groups are not large enough to support inter-group comparisons unless there are very large differences between groups. Demographic characteristics for each study cohort will be tabulated

12.1.1. Safety Analyses

Safety analysis will include data collected from all immunized subjects and infectivity controls. Adverse event data will be listed individually (including intensity and relatedness to investigational product) and categorized as local or systemic and as solicited or unsolicited AEs. Serious and/or unexpected AEs will also be discussed on a case-by-case basis. For the tabulation of AEs only the highest intensity of a specific AE will be recorded. The frequency and percentage will be summarized in by cohort in a table(s).

All adverse experiences will be described on the CRFs using standard medical terminology. The investigator will evaluate all adverse experiences as to their severity and relationship to the immunizations or challenge, and will report outcome and action taken, if any.

At each visit /assessment, all adverse events either observed or reported by the subject spontaneously or in response to a direct question will be evaluated. The nature of each event, date and time (where appropriate and if accurately recalled) of onset, outcome, maximum intensity and relationship to immunization will be established and details of corrective action (if any) will be documented. Duration will be calculated arbitrarily as the number of 24 hour periods bridged by the adverse event, with the cut-off between days being 24:00.

Adverse events already documented in the CRF (ie, at a previous assessment) and designated as “ongoing” will be reviewed at subsequent visits.

If these ongoing events have resolved, the documentation in the CRF will be completed. If an adverse event increases in frequency or intensity during a study period, a new record of the event will be started. Adverse experience data will be listed individually and summarized as local or systemic in nature. Serious and/or unexpected AEs will be discussed on a case-by-case basis. For the tabulation of AEs, a given AE for a particular study subject will be counted only once even if it occurs several times, and for tabulating the number of subjects experiencing AE’s, each subject will be counted only once. For example, a subject reporting nausea and diarrhea will be reported as one subject experiencing AEs, and the symptoms will be listed as two AEs, even if nausea and/or vomiting occurred more than one time during the period of observation.

Preliminary analyses of the safety data may be performed prior to the closing of the database or final database lock as specified in the statistical analysis plan after a database freeze. The database freeze shall be conducted in consultation with appropriate representatives at the USAMMDA. The preliminary analyses will facilitate NMRC’s timely preparation and writing of the results and conclusions for the final study report and subsequent manuscript.

12.1.2. Effectiveness Analysis

Protection will be defined by lack of parasitemia during the duration of the study. Frequency and percentage of protected subject will be summarized by cohort. Time to Parasitemia will be graphed by Kaplan Meier Curve.

12.1.3. Laboratory Data Analysis

Abnormal hematology and serum chemistry laboratory results will be tabulated by subject and by specific laboratory parameter. This will include day of onset, day of resolution, and intensity. These tables will be reviewed by the PI to evaluate whether any significant trends in laboratory values occurred.

12.2. Planned Enrollment and Reason for Sample Size

The target enrollment is 60 subjects: 24 for the immunized group, 8 mock-immunized, 12 infectivity controls, 16 alternates. This sample size is consistent with other Phase 1 studies at NMRC/WRAIR.

The hyperimmunization phase will enroll only 2 to 6 research subjects. This number cannot be higher due to logistic limitations during the immunization of Cohort 2: no more that 6 research subjects can be added to the immunizations without exceeding the insectary's capacity. Even with this very small sample size, the data obtained from these research subjects will be extremely valuable. Any findings with regard to immune measures will help to generate hypotheses for future testing with larger sample sizes. In addition, since the plasmablasts isolated from these research subjects will be used to generate human monoclonal antibodies, even the discovery of a single protective monoclonal antibody would be a highly significant finding.

12.3. Interim Analysis and Stopping Rules

No interim analysis is indicated for this trial. Please refer to section 7.7.1 for study stopping rules. However, there will be a meeting of investigators once challenge results are known for Cohort 1 to determine the immunization and challenge regimen to be applied to Cohort 2.

12.4. Accounting for Missing, Unused, and Spurious Data

Safety data from all immunized subjects will be included in analysis. Nonanalyzable data will be documented.

12.5. Procedures for Reporting Deviations from the Original Statistical Plan

Any deviation(s) from the original statistical plan as indicated in the protocol will be described in an amendment to the protocol and the Statistical Analysis Plan.

12.6. Blinding of Research Subjects and Study Personnel

Research subjects in the immunization group will be blinded to their status as mock-immunized or true-immunized subjects. Infectivity control subjects will not be blinded as they are recruited specifically as infectivity control subjects. NMRC CTC staff and WRAIR Entomology staff will be aware of each subject's group. Immunologist, microscopists, and other study personnel

receiving biological specimens will be blinded to immunization status and/or protection status unless there is a valid scientific or safety reason for unblinding of samples.

12.7. Selection of Subjects to be Included in Analyses

Data collected from all immunized subjects will be analyzed for safety. Efficacy (protection or lack thereof) will focus on those subjects completing all immunization sessions and challenge per protocol (per protocol analysis). An intention to treat analysis (ITT) is not an important outcome in this study. Correlates of immunity will be analyzed only for study subjects completing all immunization sessions and challenge (per protocol analysis).

13. Direct Access to Source Data/Documents

Subjects will be identified on source documents/CRFs and database records by a unique subject identification number. No personal identifier will be used in any publication or communication used to support this research study. The subject identification number will be used if it becomes necessary to identify data specific to a single subject. Representatives of USAMRMC, the sponsor's representative, the local IRB, and the FDA are eligible to review medical and research records related to this study as a part of their responsibility to protect human subjects in clinical research. Personal identifiers will be removed from photocopied medical and research records.

13.1. Study Photographs

Photographs may be taken of research subjects during the IMRAS trial in order to document procedures, clinical signs for documentation/diagnosis/treatment, or for future educational purposes. Photographs will be linked to subject's unique identification number and no personal identifier will be used in publication or communication used to support this research study.

13.2. Study Monitoring

Study monitoring will be the responsibility of the USAMMDA CSSD. Upon successful approval of the protocol and establishment of the regulatory file, an external clinical monitor appointed by USAMMDA will establish a clinical monitoring plan. To ensure that the investigator and the study staff understand and accept their defined responsibilities, the external clinical monitor will maintain regular correspondence with the site and may be present during the course of the study to verify the acceptability of the facilities, compliance with the investigational plan and relevant regulations, and the maintenance of complete records.

Monitoring visits by a sponsor's representative-designated external clinical monitor will be scheduled to take place at the initiation of the study, during the study at appropriate intervals, and after the last subject has completed the study. A report of monitoring observations will be provided to the PI (for corrective actions) and to the USAMRMC Division of Regulated Activities and Compliance.

13.3. Audits and Inspections

Authorized representatives of the sponsor, the FDA, or the Institutional Review Board may visit the site to perform audits or inspections, including source data verification. The purpose of the audit or inspection is to systematically and independently examine all study-related activities and documents to determine whether these activities were conducted, and data were recorded, analyzed, and accurately reported according to the protocol, ICH, GCP, and any applicable regulatory requirements.

The investigator should contact the IRB, sponsor's representative, and ORP HRPO immediately if contacted by a regulatory agency about an inspection.

13.4. Institutional Review Board (IRB)

The PI must obtain NMRC IRB approval for the study. Initial IRB approval, and all materials approved by the IRB for this study, including the subject consent form and recruitment materials, must be maintained by the investigator and made available for inspection. The PI will be

responsible for preparing and submitting continuing review reports per institution and IRB policies. The PI or a designee will submit the approved continuing review reports and the local IRB approval notifications to HRPO as soon as the documents are available. The PI or a designee will transmit the approved final study report and the local IRB approval notification to the USAMRMC ORP HRPO as soon as the documents are available. IRB approvals and reports, continuing review reports and supporting documents will also be submitted to WRAIR HSPB for acknowledgment via usarmy.detrick.medcom-wrair.mbx.hspb@mail.mil.

14. Quality Control and Quality Assurance

Internal Quality Control and Quality Assurance will be conducted by the NMRC CTC's Quality Assurance (QA) staff to ensure regulatory compliance, data integrity, and adherence to NMRC CTC's SOPs.

15. Ethics

15.1. Ethics Review

The study is based on adequately performed clinical and laboratory evaluations; the study will be conducted under a protocol reviewed by the NMRC IRB and the WRAIR DHSP. The study is to be conducted by scientifically and medically qualified persons; the benefits of the study are in proportion to the risks; the rights and welfare of the subjects will be respected; the physicians conducting the study will ensure the study does not entail undue hazards to the research subjects; the results to be reported will be accurate; subjects will give their informed consent and will be competent to do so and not under duress; and all study staff will comply with the ethical principles in 21 CFR Part 50 and the Belmont Principles.

15.1.1. Review/Approval of Study Protocol

Before a clinical study can be initiated, in addition to IRB review (see section 13.4), the study protocol and other required documents will be submitted to the following departments in the order listed for review and/or approval, with the final review by the FDA:

- Sponsor’s Representative Team (Division of Regulated Activities and Compliance, USAMMDA)
- NMRC ORA
- WRAIR Human Subjects Protection Branch (WRAIR HSPB)
- Commander, NMRC
- ORP HRPO
- Sponsor’s Representative (acting for The Surgeon General-Department of the Army)
- USAMRMC Commanding General, if applicable

Enrollment in this protocol may not begin until all approvals have been obtained and the formal authorization letter is received by the PI from the sponsor’s representative.

15.1.2. Protocol Modifications

All modifications to the protocol and supporting documents (informed consent, study-specific procedures, recruitment materials, etc.) must be reviewed and approved prior to implementation. Any protocol amendment will be agreed upon and approved by the sponsor’s representative prior to submission to the local IRB and prior to implementation of said change or modification. The informed consent document must be revised to concur with any amendment as appropriate and must be reviewed and approved with the amendment.

Any subject already enrolled in the study will be informed about the revision and asked to sign the revised informed consent document if the modification directly affects the individual’s participation in the study. A copy of the revised, signed, and dated informed consent document will be given to the subject. All original versions of the informed consent document will be retained in the protocol regulatory file, and a copy will be retained in the clinic medical record.

Any modification that could potentially increase risk to subjects must be submitted to the ORP HRPO for approval prior to implementation. Documentation that the local IRB reviewed and approved the modifications also will be submitted. All other amendments will also be submitted to the ORP HRPO for inclusion in the HRPO study file. Additionally, the WRAIR Commander approval authorization will be required for all protocol modifications and protocol amendments.

15.1.3. Protocol Deviation Procedures

All subject-specific deviations from the protocol (ie, failure to return for follow-up visits or blood collection within the time indicated in the protocol) are to be documented. The PI or designee will be responsible for identifying and reporting all deviations, which are defined as isolated occurrences involving a procedure that did not follow the study protocol or study-specific procedure. Deviations will be reported annually in the continuing review report to the NMRC IRB and ORP HRPO, and if appropriate, in the final study report. Action taken in response to the deviation and the impact of the deviation will be assessed by the PI or clinical investigator and recorded as significant or nonsignificant.

If a protocol deviation jeopardizes the safety or rights of a subject or scientific integrity of the study, the deviation will be reported immediately to the sponsor’s representative, NMRC IRB, and the ORP HRPO.

15.2. Ethical Conduct of the Study

This study will be conducted in accordance with 21 CFR Part 50 and the Belmont Principles of respect for persons, beneficence, and justice. The procedures set out in this study are designed to ensure that the sponsor’s representative and all study personnel abide by the principles of the ICH GCP Guideline and the CFR. The PI confirms this by signing this study protocol and FDA Form 1572.

15.2.1. Confidentiality

HIPAA requires that researchers obtain the subject’s permission (HIPAA Authorization) to use and disclose health information about the subject that is either created by or used in connection with this research. The information includes the entire research record and supporting information from the subject’s medical records, results of laboratory tests, and both clinical and research observations made during the individual’s participation in the research.

In this research, the subject’s health information will be collected and used to conduct the study; to monitor the subject’s health status; to measure effects of the study product; to determine research results, and possibly to develop new tests, procedures, and commercial products. Health information is used to report results of research to the sponsor’s representative and Federal regulators and may be reviewed during study audits for compliance with study plans, regulations, and research policies. After the study ends, each subject has the right to see and receive a copy of his/her information.

Representatives of the TSG-DA as the IND sponsor, USAMMDA as the sponsor’s representative, the HQ USAMRMC IRB, the DoD, and the FDA are eligible to photocopy and review records related to this protocol as a part of their responsibility to protect the participants of this treatment protocol. No personal identifier will be used in any publication or

communication used to support this research study. The subject's identification number will be used in the event it becomes necessary to identify data specific to a single subject.

15.2.2. Compensation for Participation

In general, compensation for completing the screening process will be $75 for all subjects. **For civilian subjects:** Compensation for study visits not involving leukapheresis or immunization will be $75 per visit for civilian subjects. Compensation for leukapheresis procedure and challenge study visits is $250 and compensation for immunization study visits is $200. **For subjects who are on active duty or federal employees:** compensation is limited to $50 unless the subject is on approved leave or on an off-duty status as determined by the service member's supervisor (in these cases the subject who is on active duty or a federal employee will be eligible for the same compensation as civilian subjects).

Compensation will occur at the time of each designated visit. Compensation will be provided only for completed study procedures designated for compensatory payment. Total amount of compensation may vary depending upon group (for example, Cohort 2 may receive 7 doses plus challenge). Compensation will also be provided for necessary unscheduled visit requiring blood draw ($75). Compensation will also vary depending upon when/if subjects become parasitemic. Subjects that become parasitemic will be given $75 per dose of anti-malarial medication to encourage strict adherence to the treatment regimen. Infectivity Controls will be paid only for the days during which they participate (screening, challenge, and all challenge follow-up). Subjects who serve as alternates, but who are not immunized/challenged will be paid for the screening visit and for the day when they serve as an alternate ($100.00 for the latter).

A detailed compensation plan is provided in Table 12.

Table 12: Compensation Plan

Activities	Number of Visits	Compensation per Visit ($)	Total Compensation ($)
Screening	1	75	75
Pre-immunization leukapheresis	1	250	250
Immunization	5	200	1,000
Follow-up visits	24	75	1,800
Post-immunization leukapheresis	1	250	250
Challenge	1	250	250
Post-CHMI leukapheresis	2	250	500
Overnight Stay	12	75	900
Anti-malaria medication	4	75	300
Blood Smear Follow-up	3	75	225
Follow-Up	3	75	225
Bonus for Completion	1	250	250
Total Cohort 1[a, d]	54		5,875
Total Cohort 2[b, d]	54		6,575
Total Cohort 1 Sub-cohort[c, d]	32		3,775

Activities	Number of Visits	Compensation per Visit ($)	Total Compensation ($)
Total Infectivity Controls [d]	12		2,500

[a] Maximum planned amount for any subject in Cohort 1 who completes all visits.
[b] Maximum planned amount and number of visits for Cohort 2 will vary depending upon adjustable dosing regimen.
[c] Maximum planned amount for Cohort 1 Hyperimmunation Sub-cohort who completes all visits in addition to Cohort 1 compensation and visits.
[d] Total compensation is approximate as total number and type of visits will be unique for each subject. Amounts of compensation may exceed the planned maximum amounts listed secondary to compensated unplanned visits (eg evaluation of adverse events; second attempt to complete leukapheresis procedures in the event of failure; a subject that becomes parasitemic will have fewer overnight hotel stays but will receive compensation for anti-malarial medical dosing, etc.).

15.2.3. Medical Care for Research-Related Injuries

All nonexempt research involving human subjects shall, at a minimum, meet the requirement of 32 CFR 219.116(a)(6).

If a subject is injured because of participation in this research and is a DoD healthcare beneficiary (eg, active duty in the military, military spouse, or dependent), the subject is entitled to medical care for that injury within the DoD healthcare system, as long as the subject remains a DoD healthcare beneficiary. This care includes, but is not limited to, free medical care at military hospitals or clinics.

If a subject is injured because of participation in this research and is not a DoD healthcare beneficiary, the subject is entitled to free medical care for that injury at an military hospital or clinic. It cannot be determined in advance which military hospital or clinic will provide care. If the subject receives care for research-related injuries outside of a military hospital or clinic, the subject or the subject’s insurance will be responsible for medical expenses

For all subjects: Transportation will not be provided from domicile to study location, Military hospitals, clinics, or the CTC; however, transportation between the CTC and the WRAIR/NMRC insectary will be provided on days of immunization and challenge. No reimbursement is available if the subject incurs medical expenses to treat research-related injuries. No compensation is available for research-related injuries or lost time from work. The subject is not waiving any legal rights. The subject should contact the PI if the subject believes he or she has sustained a research-related injury. The subject should contact the PI for any questions.

Military members retain the right to pursue military disability benefits, and Federal civilian employees retain the right to pursue relief through established workers compensation processes, but neither military disability benefits nor workers compensation benefits are guaranteed. The right of other parties to seek redress against the United States Government is limited to that set forth by existing agency regulations and the Federal Tort Claims Act. The subject should understand that this does not constitute a waiver or release of legal rights. This issue is addressed in the informed consent and will be discussed with the subject by the investigator or designee before the subject signs the informed consent to participate in the study. Requests for other benefits, such as compensation for lost time from work, are processed independently of this protocol.

15.3. Written Informed Consent

The informed consent process and document will be reviewed and approved by the IRB and sponsor's representative prior to initiation of the study. The consent document contains a full explanation of the possible risks, advantages, and alternate treatment options, and availability of treatment in the case of injury, in accordance with 21 CFR 50. The consent document indicates that by signature, the subject permits access to relevant medical records by the sponsor's representative and by representatives of the FDA.

The sponsor's representative will submit a copy of the initial IRB- and sponsor's representative-approved consent form to the FDA and will maintain copies of revised consent documents that have been reviewed and approved by the NMRC IRB. A written informed consent document, in compliance with 21 CFR Part 50, 32 CFR Part 219, the Belmont Principles, and HIPAA Authorization will be signed by the subject before any study-related procedures are initiated for that subject. This consent document must be retained by the investigator as part of the study records. The investigators or their designees will present the protocol in lay terms to individual subjects. Questions on the purpose of the protocol, protocol procedures, and risks to the subjects will then be solicited. Any question that cannot be answered will be referred to the PI. No subject should grant consent until he or she has had ample time to review the informed consent document and questions have been answered to his/her satisfaction.

The subject should understand that the study product is an investigational product and is not licensed by the FDA for commercial use, but is permitted to be used in this clinical research. Informed consent includes the principle that it is critical the subject be informed about the principal potential risks and benefits. This information will allow the subject to make a personal risk versus benefit decision and understand the following:

- Participation is entirely voluntary.
- Subjects may withdraw from participation at any time.
- Refusal to participate involves no penalty.
- The individual is free to ask any questions that will allow him/her to understand the nature of the protocol.
- A description of this clinical trial will be available on http://www.ClinicalTrials.gov, as required by US law.

Should the protocol be modified, the subject consent document must be revised to reflect the changes to the protocol. If a previously enrolled subject is directly affected by the change, the subject will receive a copy of the revised informed consent document. The approved revision will be read, signed, and dated by the subject.

16. Data Handling and Recordkeeping

A detailed data management plan will be written and approved by the study team and the PI prior to study start. All updates to the data management plan must be approved before study close-out and database lock.

The primary source document for this study will be the subject's record. The source documents will be retained at the site.

For this study, an EDC database system will be used for the electronic collection of the hardcopy source documents and CRF study data. The EDC database system will be designed based on the protocol requirements, and the approved layouts and specifications in accordance with 21 CFR Part 11. The database interface (data entry screens) layouts and specifications define and identify the applicable source data that will be collected and captured into the EDC database system. The applicable source data will be entered by the site designee in the EDC database system. The investigator is responsible for the accuracy of the data transcribed on the EDC database. Data monitoring and management will be performed in the EDC database system by the NMRC CTC data specialist and QA staff and the sponsor’s representative.

16.1. Inspection of Records

The sponsor’s representative or designee will be allowed to conduct site visits to the investigation facilities for the purpose of monitoring any aspect of the study. The investigator agrees to allow the monitor to inspect the IND storage area, IND product stocks, accountability records, subject charts, study source documents, and other records relative to study conduct.

Subjects’ health information is used to report results of research to the sponsor’s representative and Federal regulators and may be reviewed during study audits for compliance with study plans, regulations, and research policies. The consent document indicates that by signature, the subject permits access to relevant medical records by the sponsor’s representative and by representatives of the FDA. Upon a subject’s termination from the trial, completed source/CRFs will be ready and available for on-site review by the sponsor’s representative or the designated representative within 14 days of receipt of study data.

16.2. Retention of Records

The PI must maintain all documentation relating to the study for a period of 2 years after the last marketing application approval, or if not approved for 2 years following the discontinuance of the investigational product for investigation. If it becomes necessary for the sponsor’s representative or designee or the FDA to review any documentation relating to the study, the investigator must permit access to such records. Completed, monitored EDC data will be stored on a secure network drive with password controlled access.

The PI will be responsible for retaining sufficient information about each subject, i.e., name, address, telephone number, Social Security Number, and subject identifier in the study, so that the sponsor’s representative, the local IRB, the FDA, employees of USAMRMC, or other regulatory authorities may have access to this information should the need arise.

It is the policy of the USAMRMC that data sheets are to be completed for all subjects participating in research (Form 60-R, Subject Registry Data Sheet). The data sheets will be entered into this Command’s Subject Registry Database. The information to be entered into this

confidential data base includes the subject’s name, address, and Social Security Number; study title; and dates of participation. The intent of this data base is twofold: first, to readily answer questions concerning an individual’s participation in research sponsored by USAMRMC; and second, to ensure that USAMRMC can exercise its obligation to ensure research subjects are adequately warned (duty to warn) of risks and to provide new information as it becomes available. The information will be stored at USAMRMC for a minimum of 75 years. The Subject Registry Database is a separate entity and is not linked to the study database.

17. Publication Policy

All data collected during this study will be used to support this IND. All data may be published in the open medical or military literature with the identity of the subjects protected. Anyone desiring to publish or present data obtained during the conduct of the study will conform to NMRC Command policies and publication review clearance procedures. Publications must also be forwarded for review to the Commander, USAMMDA or designee and usarmy.detrick.medcom-usamrmc.list.clearances@mail.mil prior to submission.

18. References

Armed Forces Health Surveillance Center. Case report: fatal outcome of falciparum malaria acquired in Liberia, U.S. Navy member. MSMR.2010a;17.

Armed Forces Health Surveillance Center. Outbreak report: malaria in a U.S. Marine reserve unit deployed to Benin. MSMR. 2010b:17.

Beadle C, Hoffman SL. History of malaria in the United States Naval Forces at war: World War I through the Vietnam conflict. Clin Infect Dis. 1993;16,320-9.

Breman JG, Alilio MS, Mills A. Conquering the intolerable burden of malaria: what's new, what's needed: a summary. Am J Trop Med Hyg. 2004;71:1-15.

Butler NS, Vaughan AM, Harty JT, Kappe SH. Whole parasite vaccination approaches for prevention of malaria infection. Trends Immun. 2012;33:247-54.

Centers for Disease Control Prevention. Malaria acquired in Haiti - 2010. MMWR Morb Mortal Wkly Rep 2010;59:217-9.

Church LW, Le TP, Bryan JP, Gordon DM, Edelman R, Fries L, et al. Clinical manifestations of Plasmodium falciparum malaria experimentally induced by mosquito challenge. J Inf Dis. 1997;175:915-20.

Clyde DF. Immunization of man against falciparum and vivax malaria by use of attenuated sporozoites. Am J Torp Med Hyg. 1975;24:397-401.

Clyde DF. Immunity to falciparum and vivax malaria induced by irradiated sporozoites: a review of the University of Maryland studies, 1971-75. Bull World Health Organ. 1990;68 Suppl:9-12.

Clyde DF, McCarthy VC, Miller RM, Hornick RB. Specificity of protection of man immunized against sporozoite-induced falciparum malaria. Am J Med Sci. 1973a;266:398-403.

Clyde DF, Most H, McCarthy VC, Vanderberg JP. Immunization of man against sporozite-induced falciparum malaria. Am J Med Sci. 1973b;266:169-77.

Collins WE, Contacos PG. Immunization of monkeys against Plasmodium cynomolgi by X-irradiated sporozoites. Nat New Biol. 1972;236:176-7.

Doolan DL, Hoffman SL. Pre-erythrocytic-stage immune effector mechanisms in Plasmodium spp. infections. Philos Trans R Soc Lond B Biol Sci. 1997;352:1361-7.

Doolan DL, Hoffman SL. The complexity of protective immunity against liver-stage malaria. J Immun. 2000;165:1453-62.

Doolan DL, Southwood S, Freilich DA, Sidney J, Graber NL, Shatney L, et al. Identification of Plasmodium falciparum antigens by antigenic analysis of genomic and proteomic data. Proc Natl Acad Sci USA. 2003;100:9952-7.

Edelman R, Hoffman SL, Davis JR, Beier M, Sztein MB, Losonsky G, et al. Long-term persistence of sterile immunity in a volunteer immunized with X-irradiated Plasmodium falciparum sporozoites. J Infect Dis. 1993;168:1066-70.

Egan JE, Hoffman SL, Haynes JD, Sadoff JC, Schneider I, Grau GE, et al. Humoral immune responses in volunteers immunized with irradiated Plasmodium falciparum sporozoites. Am J Trop Med Hyg. 1993;49:166-73.

Epstein JE, Rao S, Williams F, Freilich D, Luke T, Sedegah M, et al. Safety and clinical outcome of experimental challenge of human volunteers with Plasmodium falciparum-infected mosquitoes: an update. J Infect Dis. 2007;196:145-54.

Gallup JL, Sachs JD. The economic burden of malaria. Am J Trop Med Hyg. 2010;64:85-96.

Gaziano TA, Young CR, Fitzmaurice G, Atwood S, Gaziano JM. Laboratory-based versus non-laboratory-based method for assessment of cardiovascular disease risk: the NHANES I Follow-up Study cohort. Lancet. 2008;371:923-31.

Gwadz RW, Cochrane AH, Nussenzweig V, Nussenzweig RS. Preliminary studies on vaccination of rhesus monkeys with irradiated sporozoites of Plasmodium knowlesi and characterization of surface antigens of these parasites. Bull World Health Organ. 1979;57 Suppl 1:165-73.

Herrington D, Davis J, Nardin E, Beier M, Cortese J, Eddy H, et al. Successful immunization of humans with irradiated malaria sporozoites: humoral and cellular responses of the protected individuals. Am J Trop Med Hyg. 1991;45:539-47.

Hill, A.V., Reyes-Sandoval, A., O'Hara, G., Ewer, K., Lawrie, A., Goodman, A., Nicosia, A., Folgori, A., Colloca, S., Cortese, R., et al. (2010). Prime-boost vectored malaria vaccines: progress and prospects. Human vaccines 6, 78-83.

Hoffman SL, Goh LM, Luke TC, Schneider I, Le TP, Doolan DL, et al. Protection of humans against malaria by immunization with radiation-attenuated Plasmodium falciparum sporozoites. J Infect Dis. 2002;185:1155-64.

Hoffman SL, Isenbarger D, Long GW, Sedegah M, Szarfman A, Waters L, et al. Sporozoite vaccine induces genetically restricted T cell elimination of malaria from hepatocytes. Science. 1989; 244:1078-81.

Krzych U, Lyon JA, Jareed T, Schneider I, Hollingdale MR, Gordon DM, Ballou WR. T lymphocytes from volunteers immunized with irradiated Plasmodium falciparum sporozoites recognize liver and blood stage malaria antigens. J Immun 1996;155:4072-7.

Kumar KA, Sano G, Boscardin S, Nussenzweig RS, Nussenzweig MC, Zavala F, Nussenzweig V. The circumsporozoite protein is an immunodominant protective antigen in irradiated sporozoites. Nature. 2006;444:937-40.

Laurens MB, Duncan CJ, Epstein JE, Hill AV, Komisar JL, Lyke KE, et al. A consultation on the optimization of controlled human malaria infection by mosquito bite for evaluation of candidate malaria vaccines. Vaccine. 2012;30:5302-4.

Malik A, Egan JE, Houghten RA, Sadoff JC, Hoffman SL. Human cytotoxic T lymphocytes against the Plasmodium falciparum circumsporozoite protein. Proc Ntl Acad Sci USA. 1991;88:3300-4.

Matuschewski K, Hafalla JC, Borrmann S, Friesen J. Arrested Plasmodium liver stages as experimental anti-malaria vaccines. Hum Vacc. 2011;7 Suppl:16-21.

Murray CJ, Rosenfeld LC, Lim SS, Andrews KG, Foreman KJ, Haring D, Fullman N, Naghavi M, Lozano R, Lopez AD. Global malaria mortality between 1980 and 2010: a systematic analysis. Lancet. 2012;379:413-31.

Nieman AE, de Mast Q, Roestenberg M, Wiersma J, Pop G, Stalenhoef A, et al. Cardiac complication after experimental human malaria infection: a case report. Malaria J. 2009;8:277.

Nussenzweig RS, Vanderberg J, Most H, Orton C. Protective immunity produced by the injection of x-irradiated sporozoites of plasmodium berghei. Nature. 1967;216:160-2.

Rénia L, Mattei D, Goma J, Pied S, Dubois P, Miltgen F, Nüssler A, , et al. A malaria heat-shock-like determinant expressed on the infected hepatocyte surface is the target of antibody-dependent cell-mediated cytotoxic mechanisms by nonparenchymal liver cells. Eur J Immunol. 1990;20(7):1445-9.

Rieckmann KH. Human immunization with attenuated sporozoites. Bull World Health Organ. 1990;68 Suppl:13-6.

Rieckmann KH, Beaudoin RL, Cassells JS, Sell KW. Use of attenuated sporozoites in the immunization of human volunteers against falciparum malaria. Bull World Health Org. 1973;57 Suppl 1:261-5.

Rieckmann KH, Carson PE, Beaudoin RL, Cassells JS, Sell KW. Letter: sporozoite induced immunity in man against an Ethiopian strain of Plasmodium falciparum. Trans R Soc Trop Med Hyg.1974;68:258-9.

Roestenberg M, McCall M, Hopman J, Wiersma J, Luty AJ, van Gemert GJ, et al. Protection against a malaria challenge by sporozoite inoculation. N Engl J Med. 2009;361:468-77.

Roestenberg M, O'Hara GA, Duncan CJ, Epstein JE, Edwards NJ, Scholzen A, et al. Comparison of clinical and parasitological data from controlled human malaria infection trials. PloS one. 2012;7:e38434.

Roestenberg M, Teirlinck AC, McCall MB, Teelen K, Makamdop KN, Wiersma J, et al. Long-term protection against malaria after experimental sporozoite inoculation: an open-label follow-up study. Lancet. 2011;377:1770-6.

Rogers WO, Malik A, Mellouk S, Nakamura K, Rogers MD, Szarfman A, et al. Characterization of Plasmodium falciparum sporozoite surface protein 2. Proc Ntl Acad Sci USA. 1992;89:9176-80.

Sandler SG, Nusbacher J. Health risk of leukapheresis donors. Haematologia.1982;15:57-69.

Schmidt NW, Butler NS, Badovinac VP, Harty JT. Extreme CD8 T cell requirements for anti-malarial liver-stage immunity following immunization with radiation attenuated sporozoites. PLoS Pathogens. 2010;6:e1000998.

Schofield L, Villaquiran J, Ferreira A, Schellekens H, Nussenzweig R, Nussenzweig V. Gamma interferon, CD8+ T cells and antibodies required for immunity to malaria sporozoites. Nature. 1987;330: 664-6.

Seguin MC, Klotz FW, Schneider I, Weir JP, Goodbary M, Slayter M, et al. Induction of nitric oxide synthase protects against malaria in mice exposed to irradiated Plasmodium berghei infected mosquitoes: involvement of interferon gamma and CD8+ T cells. J Exp Med. 1994;180:353-8.

van Meer MPA, Bastiaens GJH, Boulaksil M, de Mast Q, Gunasekera A, et al. Idiopathic acute myocarditis during treatment for controlled human malaria infection: a case report. Malar J. 2014;13:38.

Vaughan AM, Wang R, Kappe SH. Genetically engineered, attenuated whole-cell vaccine approaches for malaria. Hum Vac. 2010;6:107-13.

Vaughan AM, Mickolajczak SA, Wilson EM, Grompe M, Kaushansky A, Camargo N, et al. Complete Plasmodium falciparum liver-stage development in liver-chimeric mice. J Clin Invest. 2012;122(10):3618-28.

Verhage DF, Telgt DS, Bousema JT, Hermsen CC, van Gemert GJ, van der Meer JW, Sauerwein RW. Clinical outcome of experimental human malaria induced by Plasmodium falciparum-infected mosquitoes. Netherlands J Med. 2005;63:52-8.

Weinstein R. Hypocalcemic toxicity and atypical reactions in therapeutic plasma exchange. J Clin Apheresis. 2001;16:210-1.

Weiss WR, Sedegah M, Beaudoin RL, Miller LH, Good MF. CD8+ T cells (cytotoxic/suppressors) are required for protection in mice immunized with malaria sporozoites. Proc Natl Acad Sci USA. 1988;85:573-6.

Whitman TJ, Coyne PE, Magill AJ, Blazes DL, Green MD, Milhous WK, et al. An outbreak of Plasmodium falciparum malaria in U.S. Marines deployed to Liberia. AM J Trop Med Hyg. 2010;83:258-65.

Wizel B, Houghten R, Church P, Tine JA, Lanar DE, Gordon DM, et al. HLA-A2-restricted cytotoxic T lymphocyte responses to multiple Plasmodium falciparum sporozoite surface protein 2 epitopes in sporozoite-immunized volunteers. J Immun. 1995a;155:766-75.

Wizel B, Houghten RA, Parker KC, Coligan JE, Church P, Gordon DM, et al. Irradiated sporozoite vaccine induces HLA-B8-restricted cytotoxic T lymphocyte responses against two overlapping epitopes of the Plasmodium falciparum sporozoite surface protein 2. J Exp Med. 1995b;182:1435-45.

World Health Organization (2011). World Malaria Report.

19. Appendices

Appendix A. Informed Consent Documents

See Attached Documents

Appendix B. Personnel Roles and Responsibilities

NAME	POSITION	RESPONSIBILITY*
Eileen D. Franke Villasante, PhD	Principal Investigator	1, 9, 10, 15, 16
CAPT Judith Epstein	Clinical Investigator	1, 2, 3, 4, 6, 8, 9, 10, 16
LTC James Moon	Clinical Investigator	1, 2, 3, 4, 6, 8, 9, 10, 16
Michele Spring, MD	Clinical Investigator	1, 2, 3, 4, 6, 8, 9, 10, 16
Maria Sharina Reyes	Clinical Project Manager	1, 4, 5, 6, 8, 9, 10, 13
Joanne Lumsden	Scientific Project Manager	6, 11, 13, 15
Martha Sedegah	Research Associate	11
Yolanda Alcorta	Clinical Research Coordinator	1, 6, 8, 10, 13, 14
Anatalio Reyes	Quality Assurance Manager	4, 6, 9, 10, 13
Santina Maiolatesi	Project Manager/Regulatory	1, 6, 14
Glenna Banania	Clinical Research Nurse	1, 2, 4, 5, 6, 8, 9, 10, 14
Ivelese Padilla	Clinical Research Nurse	1, 2, 4, 5, 6, 8, 9, 10, 14
HM3 Ashley Lindstrom	Clinical Research Assistant	4, 6, 8, 10, 13
CDR Janine Danko	Research Monitor	3, 9, 12, 16
LTC Todd Villines	Cardiologist	9, 16

*Responsibility key:

1. Informed Consent Process
2. Administration of Study Product
3. Medical History / Physical Exam
4. Phlebotomy
5. Pregnancy Testing
6. Data Entry
7. Mix Study Product
8. Vital Signs
9. Review AEs
10. Source/CRFs
11. Research Assay
12. Research monitor
13. Quality Control & Assurance
14. Recruitment
15. Statistics
16. Review EKGs

Appendix C. Study Event Schedule

See Attached Documents

Appendix D. Toxicity Grading Scales

Table 13: Toxicity Grading Scale for Solicited Local Adverse Events

Local Reaction to Vaccine[a,b]	Mild (Grade 1)	Moderate (Grade 2)	Severe (Grade 3)	Potentially Life threatening (Grade 4)
Pain (as measured beyond the diameter of the mosquito container)	Does not interfere with activity	Repeated use of non-narcotic pain reliever >24 hours or interference with activity	Any use of narcotic pain reliever; prevents daily activity; urgent medical evaluation not requiring hospitalization (including Emergency Department (ED) visits)	Hospitalization for potentially life threatening event
Swelling (as measured beyond the diameter of the mosquito container)	2.5 – 5cm and does not interfere with activity	5.1 – 10 cm or interferes with activity	>10 cm or prevents daily activity; urgent medical evaluation not requiring hospitalization (including ED visits)	Necrosis or exfoliative dermatitis; Hospitalization for potentially life threatening event
Erythema (as measured beyond the diameter of the mosquito container)	2.5 – 5cm	5.1 – 10 cm	>10 cm; urgent medical evaluation not requiring hospitalization (including ED visits)	Necrosis or exfoliative dermatitis; Hospitalization for potentially life threatening event
Tenderness (as measured beyond the diameter of the mosquito container)	Mild discomfort to touch	Discomfort with movement	Significant discomfort that prevents daily activity; urgent medical evaluation not requiring hospitalization (including ED visits)	Hospitalization for potentially life threatening event
Induration (as measured beyond the diameter of the mosquito container)	2.5 – 5cm and does not interfere with activity	5.1 – 10 cm or interferes with activity	>10 cm or prevents daily activity; urgent medical evaluation not requiring hospitalization (including ED visits)	Necrosis or exfoliative dermatitis; Hospitalization for potentially life threatening event

Table 13: Toxicity Grading Scale for Solicited Local Adverse Events (Continued)

Local Reaction to Vaccine[a,b]	Mild (Grade 1)	Moderate (Grade 2)	Severe (Grade 3)	Potentially Life threatening (Grade 4)
Localized Pruritis (as measured beyond the diameter of the mosquito container)	Mild and localized to bite sites; relieved spontaneously or by local measures	Intense or more widespread than bite sites; relieved spontaneously or by systemic measures	Intense and widespread; poorly controlled despite systemic measures; urgent medical evaluation not requiring hospitalization (including ED visits)	Hospitalization for potentially life threatening event
Lymphatic streaking	Lymphatic erythematous streaking below elbow	Lymphatic erythematous streaking above elbow	Mild lymphedema; non function limiting; urgent medical evaluation not requiring hospitalization (including ED visits)	Moderate or greater lymphedema; function limiting; Hospitalization for potentially life threatening event
Axillary adenopathy	Ipsilateral axillary non tender adenopathy; does not interfere with activity	Ipsilateral tender axillary adenopathy; does not interfere with activity	Ipsilateral tender axillary adenopathy that interferes with activity; urgent medical evaluation not requiring hospitalization (including ED visits)	Hospitalization for potentially life threatening event

[a] All AEs will have an assessment of causality completed/ Per the clinical investigator’s judgment, each AE will be categorized for causality as: not related, unlikely, possibly, probably, definitely related.

[b] Per the clinical investigator’s judgment, each AE will be categorized for outcomes as: recovered, recovered with sequelae, ongoing at study conclusion, deceased, or unknown.

Table 14: Toxicity Grading Scale for Vital Signs

Vital Signs[a,b,c]	Mild (Grade 1)	Moderate (Grade 2)	Severe (Grade 3	Potentially Life threatening (Grade 4)
Fever (°F, Oral)	100.4 - 101.1	101.2 - 103.0	103.1 – 104; urgent medical evaluation not requiring hospitalization (including ED visits)	> 104 or hospitalization for hyperthermia
Tachycardia – beats per minute	101 - 120	121 - 130	> 130; urgent medical evaluation not requiring hospitalization (including ED visits)	hospitalization for arrhythmia
Bradycardia – beats per minute	45 - 54	35 - 44	< 35; urgent medical evaluation not requiring hospitalization (including ED visits)	hospitalization for arrhythmia
Hypertension (systolic) – mm Hg	141 - 150	151 - 159	> 160; urgent medical evaluation not requiring hospitalization (including ED visits)	hospitalization for malignant hypertension
Hypertension (diastolic) – mm Hg	91 - 100	101 - 110	> 110; urgent medical evaluation not requiring hospitalization (including ED visits)	hospitalization for malignant hypertension
Hypotension (systolic) – mm Hg	85 - 89	80 - 84	< 80; urgent medical evaluation not requiring hospitalization (including ED visits)	hospitalization for hypotensive shock
Respiratory Rate – breaths per minute	21-25	26 - 30	> 30; urgent medical evaluation not requiring hospitalization (including ED visits)	hospitalization for tachypnea or intubation

[a] Use clinical judgment when characterizing vital signs as adverse events among healthy subject populations; For example, conditioned athletes who may have resting heart rates of 40 beats per minute are normal and not classified as an adverse event. Other examples may include transient vasovagal hypotension, hypertension with explainable pathophysiologic cause such as poor medication compliance, and elevated/decreased oral temperature secondary to food or liquid intake.

[b] All vital signs abnormalities that are clinically determined to be AEs will have an assessment of causality completed. Per the clinical investigator’s judgment , each AE will be categorized for causality as: not related, unlikely, possibly, probably, definitely not related.

[c] Per the clinical investigator’s judgment, each AE will be categorized for outcomes as: recovered, recovered with sequelae, ongoing at study conclusion, deceased, or unknown.

Table 15: Toxicity Grading Scale for Systemic Adverse Events

Systemic (General)	Mild (Grade 1)	Moderate (Grade 2)	Severe (Grade 3	Potentially Life threatening (Grade 4)
Chills	No interference with activity or 1-2 episodes in 24 hours	Some interference with activity or > 2 episodes in 24 hours	Significant; prevents daily activity; urgent medical evaluation not requiring hospitalization (including ED visits)	Hospitalization for potentially life threatening event
Headache	No interference with activity	Repeated use of non-narcotic pain reliever > 24 hours or some interference with activity	Significant; any use of narcotic pain reliever or prevents daily activity; urgent medical evaluation not requiring hospitalization (including ED visits)	Hospitalization for potentially life threatening event
Vomiting	No interference with activity or 1-2 episodes in 24hours	Some interference with activity or > 2 episodes in 24 hours	Prevents daily activity; requires IV hydration; urgent medical evaluation not requiring hospitalization (including ED visits)	Hospitalization for potentially life threatening event
Nausea	No interference with activity or 1-2 episodes in 24hours	Some interference with activity or > 2 episodes in 24 hours	Prevents daily activity; requires IV hydration; urgent medical evaluation not requiring hospitalization (including ED visits)	Hospitalization for potentially life threatening event
Diarrhea	2-3 loose stools or < 400 grams in 24 hours	4-5 stools or 400-800 gms in 24 hours	6 or more watery stools or > 800 gms in 24 hours or requires IV hydration; urgent medical evaluation not requiring hospitalization (including ED visits)	Hospitalization for potentially life threatening event
Malaise	No interference with activity	Some interference with activity	Significant; prevents daily activity; urgent medical evaluation not requiring hospitalization (including ED visits)	Hospitalization for potentially life threatening event
Fatigue	No interference with activity	Some interference with activity	Significant; prevents daily activity; urgent medical evaluation not requiring hospitalization (including ED visits)	Hospitalization for potentially life threatening event
Myalgia	No interference with activity	Repeated use of non-narcotic pain reliever > 24 hours or some interference with activity	Significant; any use of narcotic pain reliever or prevents daily activity; urgent medical evaluation not requiring hospitalization (including ED visits)	Hospitalization for potentially life threatening event

Table 15: Toxicity Grading Scale for Systemic Adverse Events (Continued)

Systemic (General)	Mild (Grade 1)	Moderate (Grade 2)	Severe (Grade 3	Potentially Life threatening (Grade 4)
Arthralgia	No interference with activity	Repeated use of non-narcotic pain reliever > 24 hours or some interference with activity	Significant; any use of narcotic pain reliever or prevents daily activity; urgent medical evaluation not requiring hospitalization (including ED visits)	Hospitalization for potentially life threatening event
Systemic Allergic Type Reaction	Systemic pruritis	Localized urticaria	Systemic urticaria or angioedema; urgent medical evaluation not requiring hospitalization (including ED visits)	Hospitalization for potentially life threatening event

Table 16: Laboratory Value Adverse Event Grading Scale

Adverse Event Grade	Laboratory Values
WBC (Males & Females) Increase/Normal	Normal (3,600-10,600 cell/mm)^3
Grade 1	10,601– 15,000 cell/mm^3
Grade 2	15,001 – 20,000 cell/mm^3
Grade 3	20,001 – 25,000 cell/mm^3
Grade 4	> 25,000 cell/mm^3
WBC (Males & Females) Decrease/Normal	Normal (3,600-10,600 cell/mm^3)
Grade 1	3,599 -2,500 cell/mm^3
Grade 2	2,499 -1,500 cell/mm^3
Grade 3	1,499 -1,000 cell/mm^3
Grade 4	< 1,000 cell/mm^3
Lymphocyte (Males & Females) Decrease/Normal	Normal (1,000-3,100 cell/mm^3)
Grade 1	999-750 cell/mm^3
Grade 2	749 -500 cell/mm^3
Grade 3	499-250 cell/mm^3
Grade 4	< 250 cell/mm^3
Neutrophil (Males & Females) Decrease/Normal	Normal (1,800 -7,500 cell/mm^3)
Grade 1	1,799 -1,500 cell/mm^3
Grade 2	1,499 -1,000 cell/mm^3
Grade 3	999-500 cell/mm^3
Grade 4	< 500 cell/mm^3
Eosinophil (Males & Females) Increase/Normal	Normal (0-500 cell/mm^3)
Grade 1	501 – 1,500 cell/mm^3
Grade 2	1,501 – 2,500 cell/mm^3
Grade 3	2, 500 - 5,000 cell/mm^3
Grade 4	> 5,000 cell/mm^3
Hemoglobin (Males) Decrease /Normal	Normal (12.8 – 17.7 gm/dL)
Grade 1	12.7 – 12.5 gm/dL
Grade 2	12.4 – 10.5 gm/dL
Grade 3	10.4 – 8.5 gm/dL
Grade 4	< 8.5 gm/dL

Table 16: Laboratory Value Adverse Event Grading Scale (Continued)

Adverse Event Grade	Laboratory Values
Hemoglobin (Females) Decrease/Normal<br>Grade 1<br>Grade 2<br>Grade 3<br>Grade 4	Normal (11.5 – 15.4 gm/dL)<br>11.4– 11.0 gm/dL<br>10.9 – 9.5 gm/dL<br>9.4– 8.0 gm/dL<br>< 8.0 gm/dL
Platelets (Males & Females) Decrease/Normal<br>Grade 1<br>Grade 2<br>Grade 3<br>Grade 4	Normal (162,000 -427,000 cell/mm^3)<br>161,999-125,000 cell/mm^3<br>124,999-100,000 cell/mm^3<br>99,999-25,000 cell/mm^3<br>< 25,000 cell/mm^3
Creatinine (Males) Increase/Normal<br>Grade 1<br>Grade 2<br>Grade 3<br>Grade 4	Normal (0.7 – 1.29 mg/dL)<br>1.3 - 1.6 mg/dL<br>1.7 - 2.0 mg/dL<br>2.1 – 2.5 mg/dL<br>> 2.5 or requires dialysis
Creatinine (Females) Increase/Normal<br>Grade 1<br>Grade 2<br>Grade 3<br>Grade 4	Normal (0.5 – 0.99mg/dL)<br>1.0 - 1.6 mg/dL<br>1.7 - 2.0 mg/dL<br>2.1 – 2.5 mg/dL<br>> 2.5 or requires dialysis
AST (Males) Increase/Normal<br>Grade 1<br>Grade 2<br>Grade 3<br>Grade 4	Normal (0-40 U/L)<br>1.1 - 2.5 times upper limit of normal (ULN)<br>2.6 – 5.0 times ULN<br>5.1 - 10 times ULN<br>> 10 times ULN
AST (Females) Increase/Normal<br>Grade 1<br>Grade 2<br>Grade 3<br>Grade 4	Normal (0-32 U/L)<br>1.1 - 2.5 times upper limit of normal (ULN)<br>2.6 – 5.0 times ULN<br>5.1 - 10 times ULN<br>> 10 times ULN

Table 16: Laboratory Value Adverse Event Grading Scale (Continued)

Adverse Event Grade	Laboratory Values
ALT (Males) Increase/Normal<br>Grade 1<br>Grade 2<br>Grade 3<br>Grade 4	Normal (0-41 U/L)<br>1.1 - 2.5 times upper limit of normal (ULN)<br>2.6 – 5.0 times ULN<br>- 10 times ULN<br>> 10 times ULN
ALT (Females) Increase/Normal<br>Grade 1<br>Grade 2<br>Grade 3<br>Grade 4	Normal (0-33 U/L)<br>1.1 - 2.5 times upper limit of normal (ULN)<br>2.6 – 5.0 times ULN<br>- 10 times ULN<br>> 10 times ULN
Blood Urea Nitrogen (Males & Females)<br>Grade 1<br>Grade 2<br>Grade 3<br>Grade 4	Normal (6-20 mg/dL)<br>21-26<br>27-31<br>> 31<br>Requires dialysis
Alkaline Phosphatase (Males & Females) Increase/Normal<br>Grade 1<br>Grade 2<br>Grade 3<br>Grade 4	Normal (40-129 U/L)<br>1.1 - 2.0 times upper limit of normal (ULN)<br>2.1 - 3.0 times ULN<br>3.1 - 10 times ULN<br>> 10 times ULN
Total Bilirubin (Males & Females) Increase/Normal<br>Grade 1<br>Grade 2<br>Grade 3<br>Grade 4	Normal (0.0–1.0 mg/dL)<br>1.1 - 1.5 times upper limit of normal (ULN)<br>1.6 – 2.0 times ULN<br>2.1 - 3.0 times ULN<br>> 3 times ULN

All AEs will have an assessment of causality completed/ Per the clinical investigator's judgment, each AE will be categorized for causality as: not related, unlikely, possibly, probably, definitely related.
Per the clinical investigator's judgment, each AE will be categorized for outcomes as: recovered, recovered with sequelae, ongoing at study conclusion, deceased, or unknown.
